# Holophyllane A: A Triterpenoid Possessing an Unprecedented B-nor-3,4-*seco*-17,14-*friedo*-lanostane Architecture from *Abies holophylla*

**DOI:** 10.1038/srep43646

**Published:** 2017-03-02

**Authors:** Chung Sub Kim, Joonseok Oh, Lalita Subedi, Sun Yeou Kim, Sang Un Choi, Kang Ro Lee

**Affiliations:** 1 Natural Products Laboratory, School of Pharmacy, Sungkyunkwan University, Suwon 16419, Republic of Korea; 2 Department of Chemistry, Yale University, New Haven, Connecticut 06520, United States; 3 Chemical Biology Institute, Yale University, West Haven, Connecticut 06516, United States; 4 Gachon Institute of Pharmaceutical Science, Gachon University, Incheon 21936, Republic of Korea; 5 College of Pharmacy, Gachon University, #191, Hambakmoero, Yeonsu-gu, Incheon 21936, Republic of Korea; 6 Korea Research Institute of Chemical Technology, Daejeon 34114, Republic of Korea

## Abstract

A novel triterpenoid, holophyllane A (**1**), featuring a B-nor-3,4-*seco*-17,14-*friedo*-lanostane, along with its putative precursor, compound **2** were isolated from the methanol extract of the trunks of *Abies holophylla*. The 2D structure and relative configuration of **1** were initially determined via analysis of 1D and 2D NMR spectroscopic data and the assignment was confirmed by quantum mechanics-based NMR chemical shift calculations. The absolute configuration was established by comparison of the experimental and simulated ECD data generated at different theory levels. Compounds **1** and **2** exhibited moderate to weak cytotoxicity and significant inhibitory activity against nitric oxide (NO) production.

*Abies* species (Pinaceae) have been used as traditional medicines for the remedy of vascular and pulmonary diseases, indigestion, rheumatic diseases, and stomachache. Phytochemical studies revealed that *Abies* species are rich sources of diverse secondary metabolites such as terpenoids, lignans, flavonoids, phenols, and steroids^1^,^2^,^3^. Some of these bioactive molecules exhibited antitumor, antibacterial, antifungal, and anti-inflammatory activities^1^,^2^,^3^. *Abies holophylla* Maxim. is widely distributed in Korea, mainland China, and Russia, and may be considered as a prominent source of diverse bioactive compounds^2^,^4^, given our previous investigations elaborating the isolation of two rearranged abietane-type diterpenoids, lignans, and other relatively rare diterpenoids^3^,^5^,^6^.

In a continuing endeavor to discover bioactive secondary metabolites possessing novel architectures from *A. holophylla*, we delineated the structural elucidation of a rearranged lanostane-type triterpenoid, namely holophyllane A (**1**), along with its putative biosynthetic precursor **2** (Fig. 1). The 2D structure of **1** was established utilizing 1D and 2D NMR spectroscopic data, and this initial assignment was confirmed by comparison of experimental and computed chemical shift values at different quantum mechanical (QM) theory levels. The absolute configuration was assigned via experimental and calculated ECD data. The cytotoxicity and inhibitory potential of compounds **1** and **2** against NO production, a signaling molecule in the pathogenesis of inflammation^7^, were also evaluated to validate if this new scaffold may be developed into relevant drug prototypes.

## Results and Discussion

**Figure 2:**
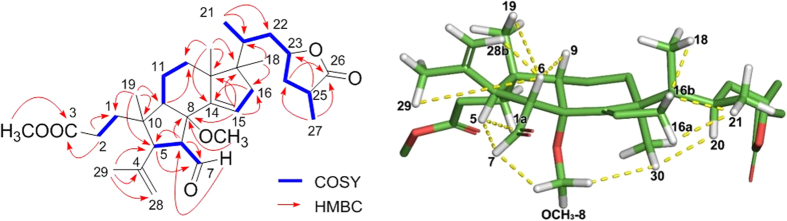
COSY (blue bolds) and HMBC (red arrows) (left) and NOESY (yellow dashed) correlations (right) of 1. The 3D structure was minimized at the B3LYP/6–31 + G(d,p) level in the polarizable continuum solvation model (PCM) (CHCl_3_).

Holophyllane A (**1**) was isolated as a colorless gum. The molecular formula was established as C_32_H_48_O_6_ based on the sodium-adduct HRFABMS ion (*m/z* 551.3347 [M + Na]^+^, calcd for C_32_H_48_O_6_Na *m/z* 551.3349) and ^13^C NMR data, indicating nine indices of hydrogen deficiency. The ^1^H NMR spectrum of **1** (Table 1) showed the presence of a formyl proton [*δ*_H_ 9.76 (1 H, d, *J* = 4.3 Hz)], three olefinic protons [*δ*_H_ 5.86 (1 H, dd, *J* = 3.4, 1.6 Hz), 4.95 (1 H, m), and 4.73 (1 H, brs)], an oxygenated methine proton [*δ*_H_ 4.63 (1 H, m)], two methoxy groups [*δ*_H_ 3.69 (3 H, s) and 3.01 (3 H, s)], and six methyl functionalities. The ^13^C NMR and DEPT spectra of **1** revealed a total of 32 carbon resonances including one formyl (*δ*_C_ 203.8), two ester-like carbonyl (*δ*_C_ 179.8 and 174.9), four olefinic (*δ*_C_ 146.4, 143.5, 126.2 and 114.8) and two oxygenated (*δ*_C_ 86.3 and 76.4) carbons. One formyl and two carbonyl groups and two double bonds accounted for five indices of hydrogen deficiency, implying the presence of four ring systems in the molecule. These spectroscopic data were similar to those of (23*R*,25*R*)-3,4-*seco*-17,14-*friedo*-9*βH*-lanosta-4(28),6,8-(14)-trien-26,23-olid-3-oic acid from *A. sachalinensis*, indicating that compound **1** is a 3,4-*seco*-17,14-*friedo*-lanostane-type triterpenoid^8^. The major difference is the presence of an olefinic functionality at C-14 and C-15 in **1** [*δ*_H_ 5.86 (1 H, dd, *J* = 3.4, 1.6 Hz)], which was validated based upon the COSY correlation of H-15/H-16 and HMBC correlations of H-15/C-8, C-13, C-16, and C-17 (Fig. 2). The cyclopentane-based B-ring was identified based on the HMBC correlations of H-19/C-9, C-10, and C-5, and H-6/C-5 and C-8, and COSY correlation of H-5/H-6 (Fig. 2). Such a B-nor-3,4-*seco*-17,14-*friedo*-lanostane scaffold is unprecedented among diverse triterpenoids. The COSY correlation of H-6/H-7 and HMBC correlation of H-5 and H-6/C-7 and H-7/C-6 corroborated that the formyl group was positioned at C-6 (Fig. 2). The location of the methoxy group was confirmed by the HMBC correlation of OCH_3_-8/C-8 (Fig. 2).

**Figure 3:**
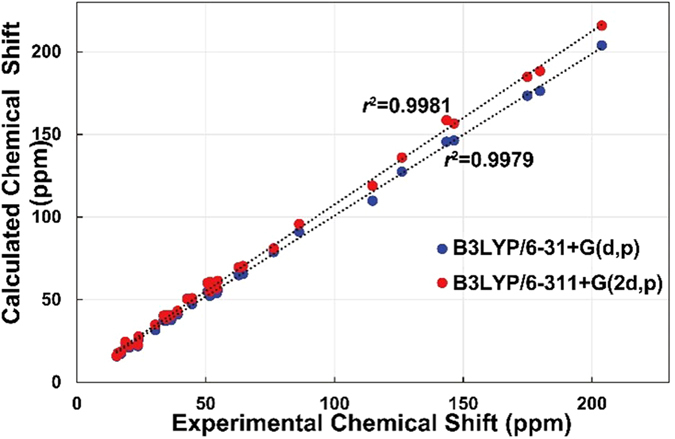
Statistical analysis of experimental and computed ^13^C NMR chemical shift values of 1.

The relative configurations of the B-ring stereogenic centers were confirmed by the NOESY correlations between H-6/H-9, H_3_-19, H-28b, and H-29, H-5/H-1a and H-7, and H-7/OCH_3_-8 as depicted in Fig. 2. The NOESY correlations of H-30/H-20 and OCH_3_-8, H-16a/H-21, and H-16b/H-18 and H-21 (Fig. 2), and the similar NMR chemical shift values of C-20 – C-27 of **1** to those of the aforementioned analogue, defined the relative configurations of **1**^8^. The presence of the conformationally flexible moieties in **1** and its mass limitation negated the application of X-ray crystallography. Instead, the NMR-based initial structural assignment of **1** was reaffirmed by the comparison of the experimental and computed NMR chemical shift values. The NMR deshielding properties were calculated employing gauge-invariant atomic orbital (GIAO)-based NMR chemical shift calculations at the B3LYP hybrid density-functional theory (DFT) method using the 6–31 + G(d,p) and B3LYP/6–311 + G(2d,p) level basis sets with PCM (CHCl_3_) (Fig. 3)^9^,^10^. The predicted chemical shift data at the two different basis sets for **1** were plotted with the experimental data. The statistical analyses of the experimental and the calculated chemical shift values at the B3LYP/6–31 + G(d,p) and B3LYP/6–311 + G(2d,p) levels, generated the correlation slopes with *r*^2^ values 0.9979 and 0.9981, respectively, verifying the 2D structure and relative configurational assignments.

**Figure 4:**
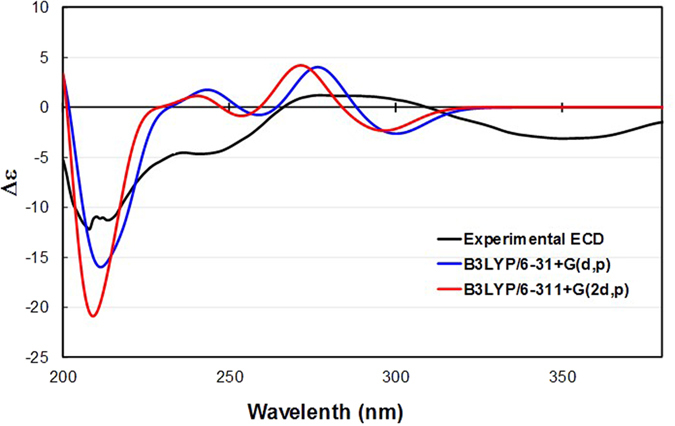
Experimental and simulated ECD spectra of 1.

The absolute configuration of **1** was established via comparison of the experimental and calculated ECD spectra (Fig. 4)^11^. The full array of conformers used for the aforementioned chemical shift computations at the B3LYP/6–31 + G(d,p) and B3LYP/6–311 + G(2d,p) levels (S1–S4) was employed for excited state DFT calculations in CHCl_3_. The excitation energies and rotational strengths of the respective conformers were Boltzmann-averaged based on the calculated Gibbs free energies and fitted to the Gaussian functions to simulate ECD curves^12^. The experimental and computed ECD spectra of **1** at the two different levels were not matched well, particularly a UV range over 300 nm, presumably due to the weak chromophores and conformational changes in the molecule. However, the comparison clearly exhibited negative Cotton effects at ca. 210 nm characteristic of the π → π* electronic transitions of olefinic moieties and positive effects at ca. 280 nm originating from the n → π* transitions of the carbonyl functionalities. This, along with the consistent biosynthetic pathway towards the formation of the lanostane architecture, permitted the assignment of the absolute configuration of **1** as (5*S*, 6*R*, 8*S*, 9*S*, 10*S*, 13*S*, 17*S*, 20*R*, 23*R*, and 25*R*).

Compound **2** was obtained as a colorless gum and its molecular formula C_31_H_46_O_4_ was established based on the protonated HRFABMS ion (*m/z* 483.3473 [M + H]^+^, calcd for C_31_H_47_O_4_ *m/z* 483.3474) and ^13^C NMR data. The ^1^H and ^13^C NMR spectra of **2** resembled those of (23*R*,25*R*)-3,4-*seco*-17,14-*friedo*-9*βH*-lanosta-4(28),6,8-(14)-trien-26,23-olid-3-oic acid^8^ except for the presence of a methoxy group deduced from the NMR resonances [*δ*_H_ 3.67 (3 H, s); *δ*_C_ 51.6]. The HMBC correlation of OCH_3_-3/C-3 confirmed that the methoxy motif was attached to C-3. The full structural assignment of **2** was carried out by analyzing the COSY, HSQC, and HMBC data (Fig. S2). The relative configuration was determined by the NOESY correlations (Fig. S2) and the absolute configuration was confirmed to be identical to that of the aforementioned lanostane^8^ based upon its specific rotation 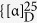
–109.0 (*c* 0.05, CHCl_3_) for **2** vs. 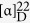
–136.8 (*c* 0.46, CHCl_3_) for the known analogue}. Thus, compound **2** was established as methyl (23*R*,25*R*)-3,4-*seco*-17,14-*friedo*-9*βH*-lanosta-4(28),6,8-(14)-trien-26,23-olid-3-oate.

**Figure 5:**
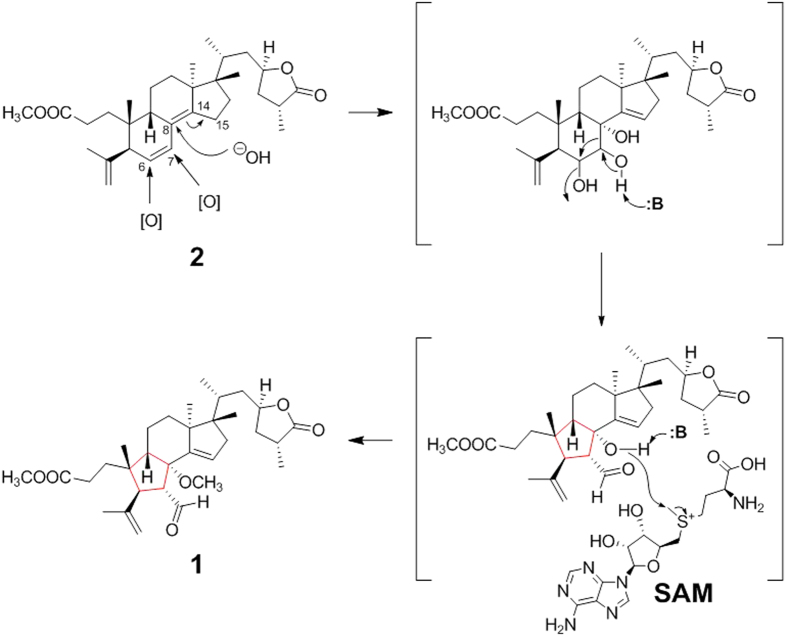
Plausible biosynthesis pathway of compound 1 based on Pinacol-Pinacolone-type rearrangement.

Holophyllane A (**1**) is a novel triterpenoid possessing an unprecedented B-nor-3,4-*seco*-17,14-*friedo*-lanostane scaffold. A proposed biosynthesis pathway of **1** is outlined in Fig. 5. A likely biogenetic precursor **2** could undergo oxidation at C-6 and C-7, and hydroxylation at C-8 with subsequent formation of the Δ^14(15)^ olefinic bond (the 1^st^ step in Fig. 5). The unique cyclopentane-based B-ring of **1** might be formed via a pinacol-pinacolone-type rearrangement (the 2^nd^ step) followed by methylation via *S*-adenosyl methionine (SAM) (the 3^rd^ step) to generate **1**. This hypothesis is supported by our previous report of the two rearranged diterpenoids, holophyllins A and B, presumably biosynthesized via such a rearrangement from abietic acid^5^.

The cytotoxic activities of compounds **1** and **2** were evaluated using quantitative staining of cellular proteins by sulforhodamine B (SRB) against the various cancer cell lines such as A549 (non-small cell lung adenocarcinoma), SK-OV-3 (ovary malignant ascites), SK-MEL-2 (skin melanoma), and HCT15 (colon adenocarcinoma)^13^. As a positive controls, etoposide and doxorubicin were employed with the respective IC_50_’s of 0.81, 1.96, 0.53, and 1.71 μM (etoposide) and 0.0014, 0.0217, 0.0025, and 0.1077 μM (doxorubicin) against the different cancer cell lines. Compound **2** exerted moderate cytotoxicity against the SK-MEL-2 cell line with the IC_50_ value of 6.86 μM, and weak activities against the other cell lines (IC_50_’s of 10.07, 12.96, and 12.08 μM for A549, SK-OV-3, and HCT15, respectively). Compound **1** exhibited weak cytotoxicity against the SK-MEL-2 cell line with an IC_50_ of 15.63 μM (Fig. S1, Supplementary Information).

**Figure 6:**
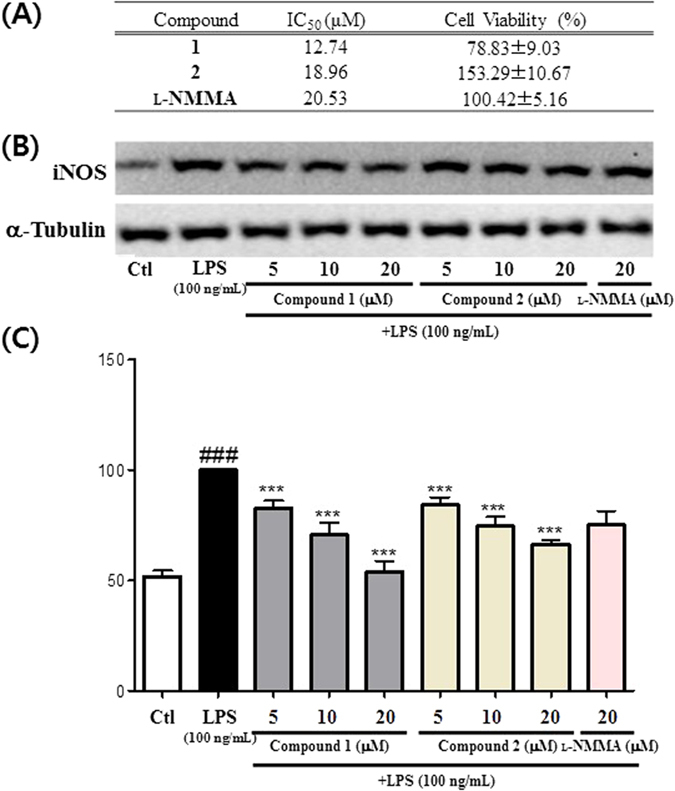
Effect of compounds 1 and 2 inhibiting NO production and iNOS expression in LPS (100 ng/mL)-stimulated BV2 cells. (**A**) IC_50_ (μM) value and cell viability at 20 μM compound treatment against LPS treated group (set as 100%). (**B**) iNOS expression in LPS-stimulated BV2 cells. (**C**) Densitometric analysis of iNOS expression. All data are presented as the mean ± SEM of three independent experiments. ^###^p < 0.001 vs. untreated Ctl cells. *p < 0.5, **p < 0.01, ***p < 0.001 vs. LPS treated cells.

The inhibitory potential of compounds **1** and **2** on NO production levels in lipopolysaccharide (LPS)-stimulated murine microglia were also evaluated^14^,^15^. Compounds **1** and **2** impeded NO production with IC_50_ values of 12.74 and 18.96 μM, respectively, without significant cell toxicity at 20 μM (Fig. 6). Thus, compounds **1** and **2** are slightly more potent than the positive control *N*^G^-monomethyl-L-arginine (L-NMMA) (IC_50_ 20.53 μM)^16^. To investigate an underlying mechanism by which these compound hampered NO production, compounds **1** and **2** were evaluated for their inhibitory potentials against inducible nitric oxide synthase (iNOS) expression in LPS-stimulated BV2 cells. The tested compounds indeed inhibited the iNOS expression in a concentration-dependent manner and similar to that of the NO inhibition result, compounds **1** and **2** were more potent than the positive control in inhibition of the target protein expression (Fig. 6). This implies that the inhibitory activity of those bioactives against NO production is cohesively correlated to their capability capable of inhibiting the iNOS expression at the cellular level.

In this current study, we have demonstrated the identification of a novel architecture **1** and its biosynthetic precursor **2** from *A. holophylla* capable of modestly hampering the growth of several cancer cell lines as well as NO production. The discovery of the new bioactive architecture may provide a basis for the development of novel chemopreventive and anti-inflammatory drug prototypes. Also, QM-NMR coupled analyses have again been shown to possess significant utility in the full structural elucidation of new bioactive entities in cases where these metabolites are mass-limited and/or X-ray crystallography is not applicable.

## Methods

### General experimental procedures

Optical rotations were measured on a JASCO P-1020 polarimeter and ultraviolet (UV) spectra were recorded with a Shimadzu UV-1601 UV-visible spectrophotometer (Shimadzu, Tokyo, Japan). NMR spectra were measured on a Varian unity INOVA 500 NMR spectrometer (Varian, Palo Alto, CA, USA) (500 MHz for ^1^H and 125 MHz for ^13^C). The HRFABMS spectra were generated utilizing a JEOL JMS700 mass spectrometer (Tokyo, Japan). The HPLC-DAD data were measured using an Agilent 1260 Infinity HPLC system (Agilent, Santa Clara, CA, USA) using a Kinetex C_18_ 5 μm column (250 mm length × 4.6 mm i.d.; Phenomenex, Torrance, CA, USA). The semi-preparative purification was performed using a Gilson 306 pump (Middleton, WI, USA) equipped with a Shodex refractive index detector (New York, NY, USA) and an Apollo Silica 5 μm column (250 mm length × 10 mm i.d.; Apollo, Manchester, UK). Low-pressure liquid chromatography (LPLC) was carried out with a LiChroprep Lobar-A Si 60 column (240 mm length × 10 mm i.d.; Merck, Darmstadt, Germany) with an FMI QSY-0 pump (Teledyne Isco, Lincoln, NE, USA). Gravity column chromatography was implemented with silica gel 60 (70–230 and 230–400 mesh; Merck, Darmstadt, Germany) and RP-C_18_ silica gel (Merck, 230–400 mesh). Precoated silica gel F_254_ plates and RP-18 F_254s_ plates (Merck) were used for thin-layer chromatography (TLC). Spots were detected by TLC under UV light or by heating upon spraying samples with anisaldehyde-sulfuric acid.

### Plant material

Trunks of *A. holophylla* were collected in Seoul, Korea in January 2012, and the plant was identified by one of the authors (K.R.L.). A voucher specimen (SKKU-NPL 1205) has been deposited in the herbarium of the School of Pharmacy, Sungkyunkwan University, Suwon, Korea.

### Extraction and isolation

Trunks of *A. holophylla* (5.0 kg) were extracted with 80% aq. MeOH under reflux and filtered. The filtrate was concentrated under a reduced pressure to obtain a MeOH extract (280 g). The crude extract was suspended in distilled H_2_O and successively partitioned with hexane, CHCl_3_, EtOAc, and *n*-butanol, yielding 23, 43, 17, and 35 g of the respective solvent residues. The CHCl_3_-soluble fraction (20 g) was chromatographed on a silica gel column (CHCl_3_-MeOH, 50:1 → 1:1) to generate 10 crude fractions (C1–C10). Fraction C2 (8.2 g) was fractionated into 10 subfractions (C21–C210) using RP-C_18_ silica gel chromatography eluting with a gradient solvent system of 90 → 100% aq. MeOH. Fraction C26 (230 mg) was applied to LPLC eluting with a solvent mixture of CHCl_3_-MeOH (100:1) to give five subfractions (C261–C265). Fraction C261 (40 mg) was purified by semi-preparative HPLC (2 mL/min, hexane/EtOAc, 7:1) to yield compound **1** (3 mg, Rt = 13.5 min). Fraction C29 (430 mg) was subjected to LPLC with an isocratic condition of hexane/EtOAc (7:1) to yield four subfractions (C291–C294). Fraction C293 (30 mg) was further purified employing semi-preparative HPLC (2 mL/min, 97% aqueous MeOH) to afford compound **2** (3 mg, Rt = 11.7 min). Each compound was analyzed by utilizing the HPLC–DAD system with a gradient program (70% aq. MeCN → 100% MeCN, 0.7 mL/min, 37 min) (Figs S3 and S4, Supplementary Information).

Holophyllane A (**1**): colorless gum; 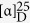
–35.5 (*c* 0.15, CHCl_3_); IR (KBr) *ν*_max_ 2966, 2840, 1747, 1727 cm^−1^; ECD (CHCl_3_) λ_max_ (Δε): 208 (−12.2), 277 (+1.1) nm, 350 (−3.1); ^1^H (CDCl_3_, 500 MHz) and ^13^C (CDCl_3_, 125 MHz) NMR data, see Table 1; positive HRFABMS *m/z* 551.3347 [M + Na]^+^ (calcd. for C_32_H_48_O_6_Na 551.3349).

Holophyllane B (**2**): colorless gum; 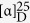
–109.0 (*c* 0.05, CHCl_3_); IR (KBr) *ν*_max_ 2959, 2843, 1759, 1647 cm^−1^; UV (MeOH) λ_max_ (log ε): 249 (4.72) nm; ^1^H NMR (CDCl_3_, 500 MHz) δ_H_ 6.22 (1 H, d, *J* = 10.0 Hz, H-7), 5.37 (1 H, dd, *J* = 10.0, 5.5 Hz, H-6), 4.97 (1 H, brs, H-28a), 4.76 (1 H, d, *J* = 1.9 Hz, H-28b), 4.63 (1 H, m, H-23), 3.67 (3 H, s, OCH_3_-3), 2.71 (1 H, m, H-25), 2.64 (1 H, d, *J* = 5.5 Hz, H-5), 2.43 (1 H, overlap, H-11a), 2.40 (1 H, m, H-9), 2.26 (2 H, t, *J* = 8.4 Hz, H-2), 2.25 (1 H, overlap, H-11b), 2.09 (1 H, overlap, H-20), 2.08 (2 H, overlap, H-24), 1.92 (1 H, m, H-22a), 1.79 (3 H, s, H-29), 1.71 (1 H, overlap, H-16a), 1.62 (2 H, overlap, H-12), 1.60 (2 H, overlap, H-1), 1.60 (2 H, overlap, H-15), 1.51 (1 H, overlap, H-16b), 1.31 (3 H, d, *J* = 7.3 Hz, H-27), 1.22 (1 H, ddd, *J* = 14.2, 11.2, 2.8 Hz, H-22b), 1.04 (3 H, s, H-30), 0.94 (3 H, d, *J* = 6.7 Hz, H-21), 0.85 (3 H, s, H-19), 0.67 (3 H, s, H-18); ^13^C NMR (CDCl_3_, 125 MHz) δ_C_ 179.9 (C-26), 174.9 (C-3), 146.7 (C-14), 145.9 (C-4), 126.7 (C-6), 125.2 (C-7), 125.1 (C-8), 115.4 (C-28), 76.7 (C-23), 51.6 (OCH_3_-3), 50.6 (C-5), 49.1 (C-17), 47.5 (C-13), 39.6 (C-9), 38.8 (C-22), 37.1 (C-10), 36.5 (C-24), 36.2 (C-16), 34.4 (C-20), 34.1 (C-25), 32.5 (C-12), 29.8 (C-2), 28.7 (C-1), 24.9 (C-29), 23.9 (C-11), 21.8 (C-19), 21.6 (C-30), 19.8 (C-15), 15.9 (C-27), 15.6 (C-18), 15.2 (C-21); positive HRFABMS *m/z* 483.3473 [M + H]^+^ (calcd. for C_31_H_47_O_4_ 483.3474).

### GIAO-based NMR chemical shift calculations

Conformational searches were performed using MacroModel with the MMFF force field (gas phase), a 10 kcal/mol upper energy limit and 0.001 kJ (mol Å)^− 1^ convergence threshold on the rms gradient^9^,^10^,^17^. The geometries of all the conformers of **1** were optimized using the B3LYP hybrid DFT method not only with the 6–31 + G(d,p) basis set in the PCM with a dielectric constant representing CHCl_3_, but also with the 6–311 + G(2d,p) basis set in the gas phase. These two different basis sets were employed to probe into the effects of including a higher basis set. The GIAO magnetic shielding tensors were calculated at the B3LYP/6–31 + G(d,p) and B3LYP/6–311 + G(2d,p) levels in the PCM (CHCl_3_) and averaged based on the Boltzmann populations of each conformer in the associated Gibbs free energy (Tables S2 and S4). The chemical shift values were calculated via an equation below where 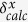 is the calculated NMR chemical shift for nucleus *x*, σ^*o*^ is the shielding tensor for the proton and carbon nuclei in tetra methylsilane calculated at the above-mentioned basis sets.


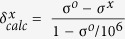


### ECD simulation

ECD calculations were performed with the conformers described in the subsection immediately above. The generated excitation energies and rotational strengths were Boltzmann-averaged on the basis of the calculated Gibbs free energy of each conformer (Tables S2 and S4, Supplementary Information) and used for ECD visualization utilizing SpecDis^12^.

### Cytotoxicity assessment

The cytotoxicity of compounds **1** and **2** was evaluated against A549 (non-small cell lung adenocarcinoma), SK-OV-3 (ovary malignant ascites), SK-MEL-2 (skin melanoma), and HCT-15 (colon adenocarcinoma) utilizing the SRB method^13^. Etoposide (≥98%; Sigma-Aldrich Co., St. Louis, MO, USA) was used as a positive control.

### Measurement of NO production and cell viability in LPS-activated BV-2 cells

The inhibitory effect of the test compounds on LPS-stimulated NO production was studied using BV2 cells. BV2 cells were seeded on a 96-well plate (4 × 10^4^ cells/well) and treated with or without different concentrations of the compounds. These cells were stimulated with LPS (100 ng/mL) and incubated for 24 h. The concentration of nitrite (NO_2_), a soluble oxidation product of NO, in the culture medium was measured using Griess reagent (0.1% *N*-1-napthylethylenediamine dihydrochloride and 1% sulfanilamide in 5% phosphoric acid). Fifty microliters of supernatant were mixed with an equal volume of the Griess reagent. Absorbance was measured after 10 min using a microplate reader (Emax, Molecular Devices, Sunnyvale, CA, USA) at 570 nm wavelength. L-NMMA, a nitric oxide synthase (NOS) inhibitor, was used as a positive control. Graded sodium nitrite solution was used as a standard to calculate nitrite concentrations. Cell viability was evaluated by the MTT assay.

## Additional Information

**How to cite this article:** Kim, C. S. *et al*. Holophyllane A: A Triterpenoid Possessing an Unprecedented B-nor-3,4-*seco*-17,14-*friedo*-lanostane Architecture from *Abies holophylla. Sci. Rep.* **7**, 43646; doi: 10.1038/srep43646 (2017).

**Publisher's note:** Springer Nature remains neutral with regard to jurisdictional claims in published maps and institutional affiliations.

## Supplementary Material

Supplementary Information

## Figures and Tables

**Figure 1:**
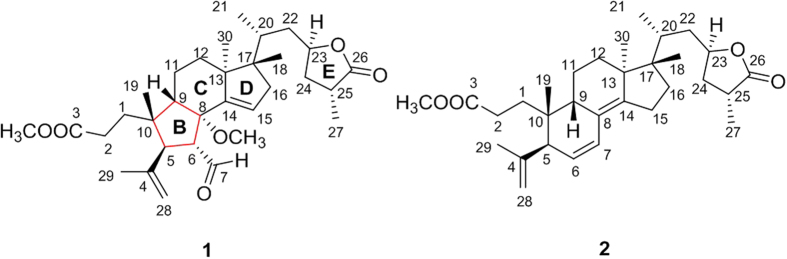
Structures of compounds 1 and 2.

**Table 1:** ^1^H and ^13^C NMR spectroscopic data of 1 in CDCl_3_.

position	*δ*_H_, mult, (*J* in Hz)	*δ*_C_
1a	2.16, overlap	35.4 (CH_2_)
1b	1.87, ddd (13.9, 12.1, 5.1)	
2a	2.49, ddd (15.2, 12.1, 5.1)	30.4 (CH_2_)
2b	2.38, ddd (15.2, 12.1, 5.1)	
3		174.9 (C)
4		143.5 (C)
5	3.15, d (11.9)	54.7 (CH)
6	2.88, dd (11.9, 4.3)	64.5 (CH)
7	9.76, d (4.3)	203.8 (CH)
8		86.3 (C)
9	1.29, overlap	62.8 (CH)
10		42.8 (C)
11a	1.92, overlap	18.8 (CH_2_)
11b	1.37, m	
12a	1.71, overlap	34.6 (CH_2_)
12b	1.41, dt (12.9, 3.2)	
13		51.7 (C)
14		146.4 (C)
15	5.86, dd (3.4, 1.6)	126.2 (CH)
16a	2.30, dd (15.9, 1.6)	44.7 (CH_2_)
16b	1.95, dd (15.9, 3.4)	
17		50.7 (C)
18	0.80, s	17.1 (CH_3_)
19	0.89, s	23.7 (CH_3_)
20	2.18, overlap	33.7 (CH)
21	0.94, d (6.4)	15.4 (CH_3_)
22a	1.72, overlap	39.1 (CH_2_)
22b	1.23, ddd (14.2, 7.5, 2.7)	
23	4.63, m	76.4 (CH)
24	2.05, m	36.6 (CH_2_)
25	2.69, m	34.1 (CH)
26		179.8 (C)
27	1.30, d (7.3)	15.9 (CH_3_)
28a	4.95, m	114.8 (CH_2_)
28b	4.73, brs	
29	1.75, s	23.9 (CH_3_)
30	1.05, s	20.3 (CH_3_)
OCH_3_-3	3.69, s	51.6 (CH_3_)
OCH_3_-8	3.01, s	54.3 (CH_3_)
