# Supplementary material for: Holophyllane A: A Triterpenoid Possessing an Unprecedented B-nor-3,4-seco-17,14-friedo-lanostane Architecture from Abies holophylla
Source: Sci Rep. 2017 Mar 2;7:43646. doi: 10.1038/srep43646 (PMC5333622; doi:10.1038/srep43646)
Supplement: Supplementary Information [file srep43646-s1.pdf]

Supporting Information

Holophyllane A: A Triterpenoid Possessing an Unprecedented B-nor-3,4-*seco*-17,14-*friedo*-lanostane Architecture from *Abies holophylla*

Chung Sub Kim[1], Joonseok Oh[2,3], Lalita Subedi[4,5], Sun Yeou Kim[4,5], Sang Un Choi[6], and Kang Ro Lee[1,*]

[1]Natural Products Laboratory, School of Pharmacy, Sungkyunkwan University, Suwon 16419, Republic of Korea

[2]Department of Chemistry, Yale University, New Haven, Connecticut 06520, United States

[3]Chemical Biology Institute, Yale University, West Haven, Connecticut 06516, United States

[4]Gachon Institute of Pharmaceutical Science, Gachon University, Incheon 21936, Republic of Korea

[5]College of Pharmacy, Gachon University, #191, Hambakmoero, Yeonsu-gu, Incheon 21936, Republic of Korea

[6]Korea Research Institute of Chemical Technology, Daejeon 34114, Republic of Korea

Contents

Figure S1. Dose response curves of compounds 1 and 2, and positive controls (etoposide and doxorubicin)........3

Figure S2. COSY (blue bolds), HMBC (red arrows), and NOESY (yellow dashed) correlations of 2........4

Figure S3. HPLC-DAD (195 nm) profiling of compound 1........5

Figure S4. HPLC-DAD (195 nm) profiling of compound 2........6

Figure S5. HRFABMS spectra of 1........7

Figure S6. ^{1}H NMR spectrum of 1........9

Figure S7. ^{13}C NMR spectrum of 1........10

Figure S8. DEPT 90 spectrum of 1........11

Figure S9. DEPT 135 spectrum of 1........12

Figure S10. COSY spectrum of 1........13

Figure S11. HSQC spectrum of 1........14

Figure S12. HMBC spectrum of 1........15

Figure S13. NOESY spectrum of 1........16

Figure S14. HRFABMS spectra of 2........18

Figure S15. ^{1}H NMR spectrum of 2........20

Figure S16. ^{13}C NMR spectrum of 2........21

Figure S17. COSY spectrum of 2........22

Figure S18. HSQC spectrum of 2........23

Figure S19. HMBC spectrum of 2........24

Figure S20. NOESY spectrum of 2........25

Table S1. Computed thermodynamic data and zero-point vibrational energy of conformers of 1 (PCM, $CHCl_3$) used for chemical shift values and ECD simulation (B3LYP/6-31+(d,p))........26

Table S2. Boltzmann populations of conformers shown in Table S1........28

Table S3. Calculated thermodynamic data and zero-point vibrational energy of conformers of 1 (gas phase) employed for chemical shift values and ECD simulation at the B3LYP/6-311+(2d,p) level........30

Table S4. Boltzmann populations of conformers described in Table S3........32

Table S5. Experimental and PCM calculation ($CHCl_3$) for ^{13}C chemical shift values of compound 1 at B3LYP/6-31G+(d,p) and B3LYP/6-311G+(2d,p)........34

Coordinates of conformers found at B3LYP/6-311G+(2d,p) in the gas phase........35

Coordinates of conformers at B3LYP/6-31G+(d,p) in the PCM ($CHCl_3$)........151

Figure S1. Dose response curves of compounds 1 and 2, and positive controls (etoposide and doxorubicin)

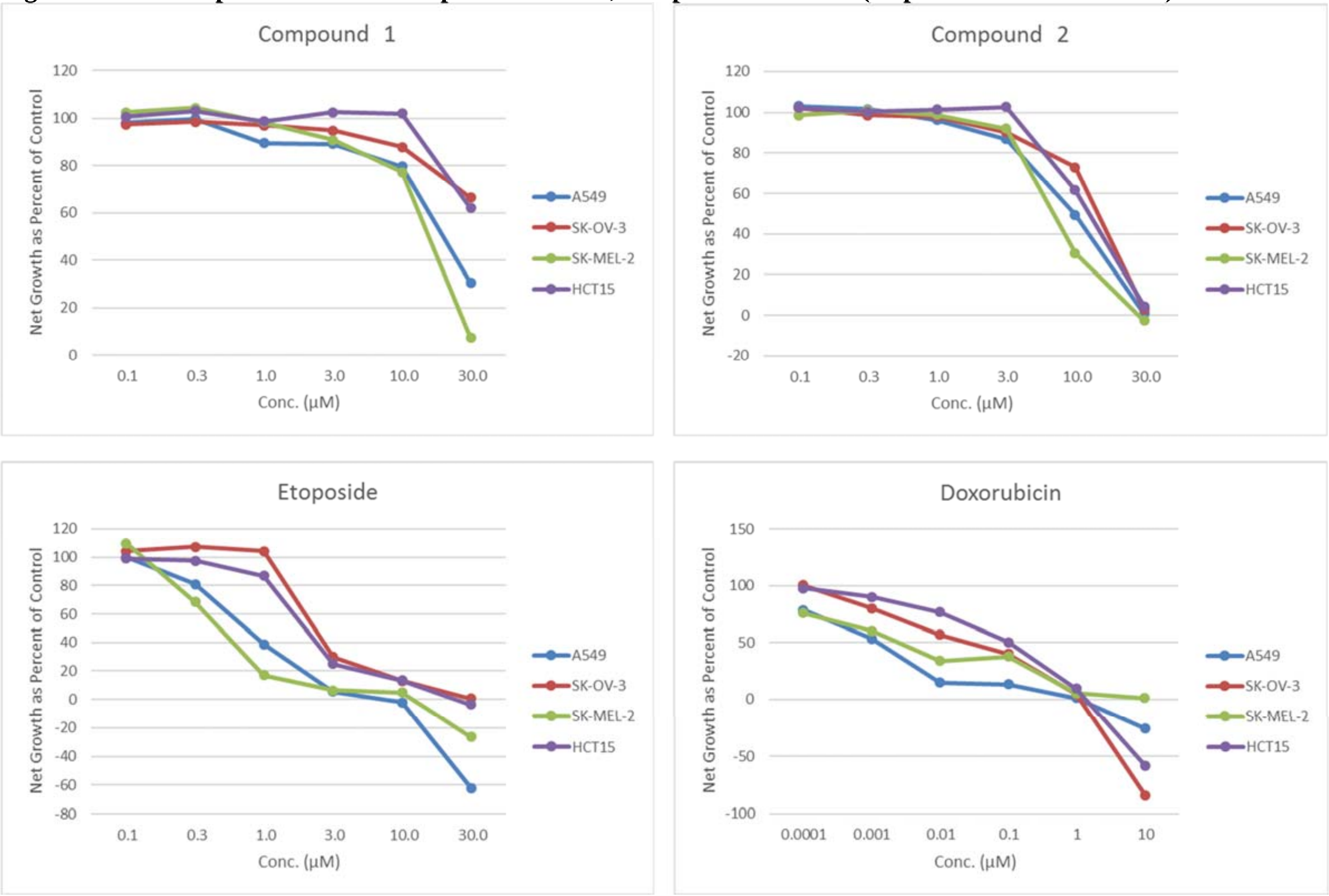

Figure S2. COSY (blue bolds), HMBC (red arrows), and NOESY (yellow dashed) correlations of 2

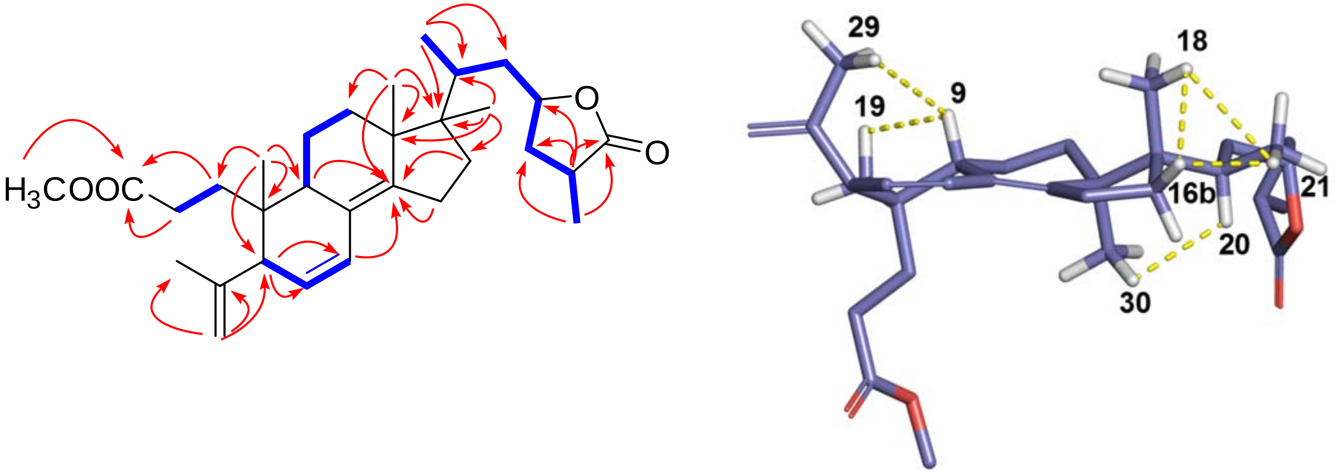

Figure S3. HPLC–DAD (195 nm) profiling of compound 1

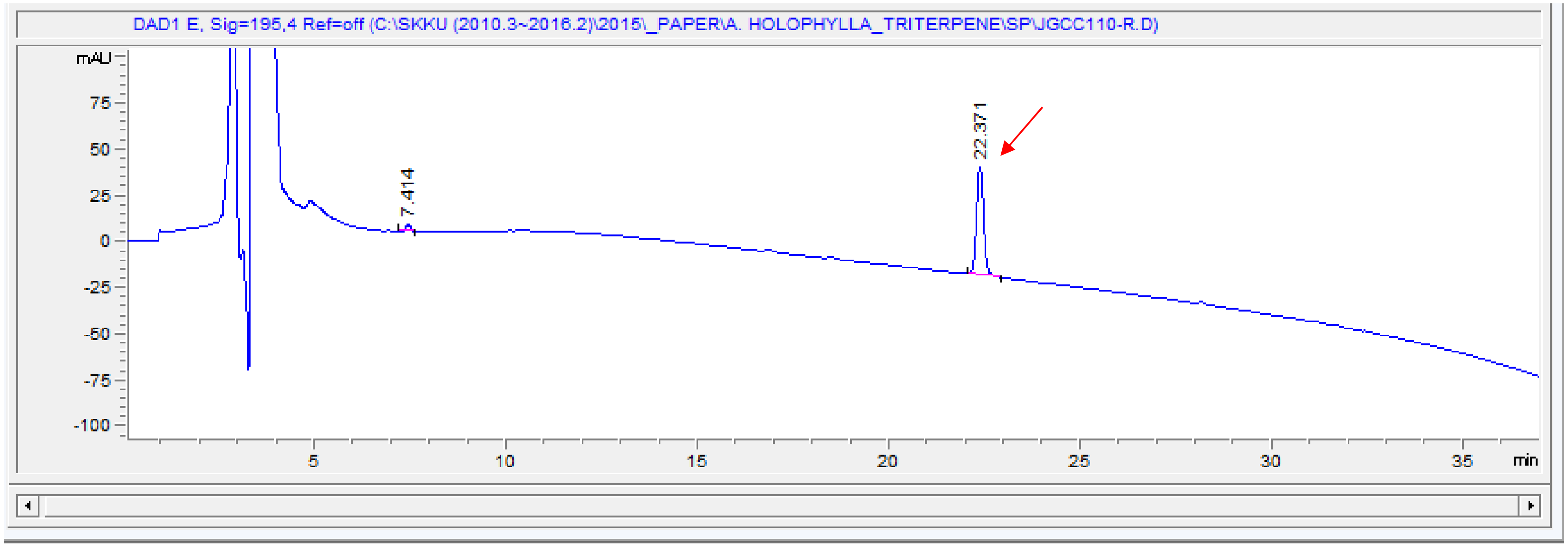

#	Time	Area	Height	Width	Area%	Symmetry
1	7.414	34.9	4	0.1426	4.463	1.056
2	22.371	746.6	58.6	0.1987	95.537	0.935

Purity of compound **1**: **95.5%**

Figure S4. HPLC–DAD (195 nm) profiling of compound 2

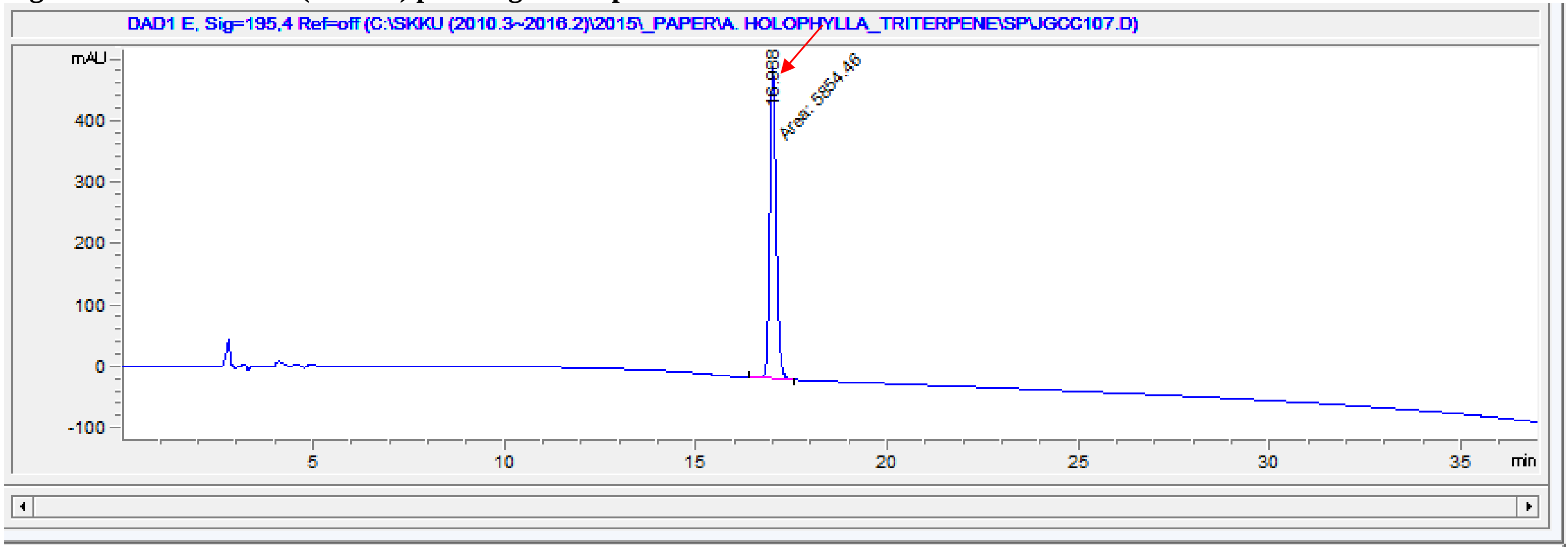

#	Time	Area	Height	Width	Area%	Symmetry
1	16.988	5854.5	508.1	0.192	100.000	0.796

Purity of compound **2**: **>98%**

Figure S5. HRFABMS spectra of 1

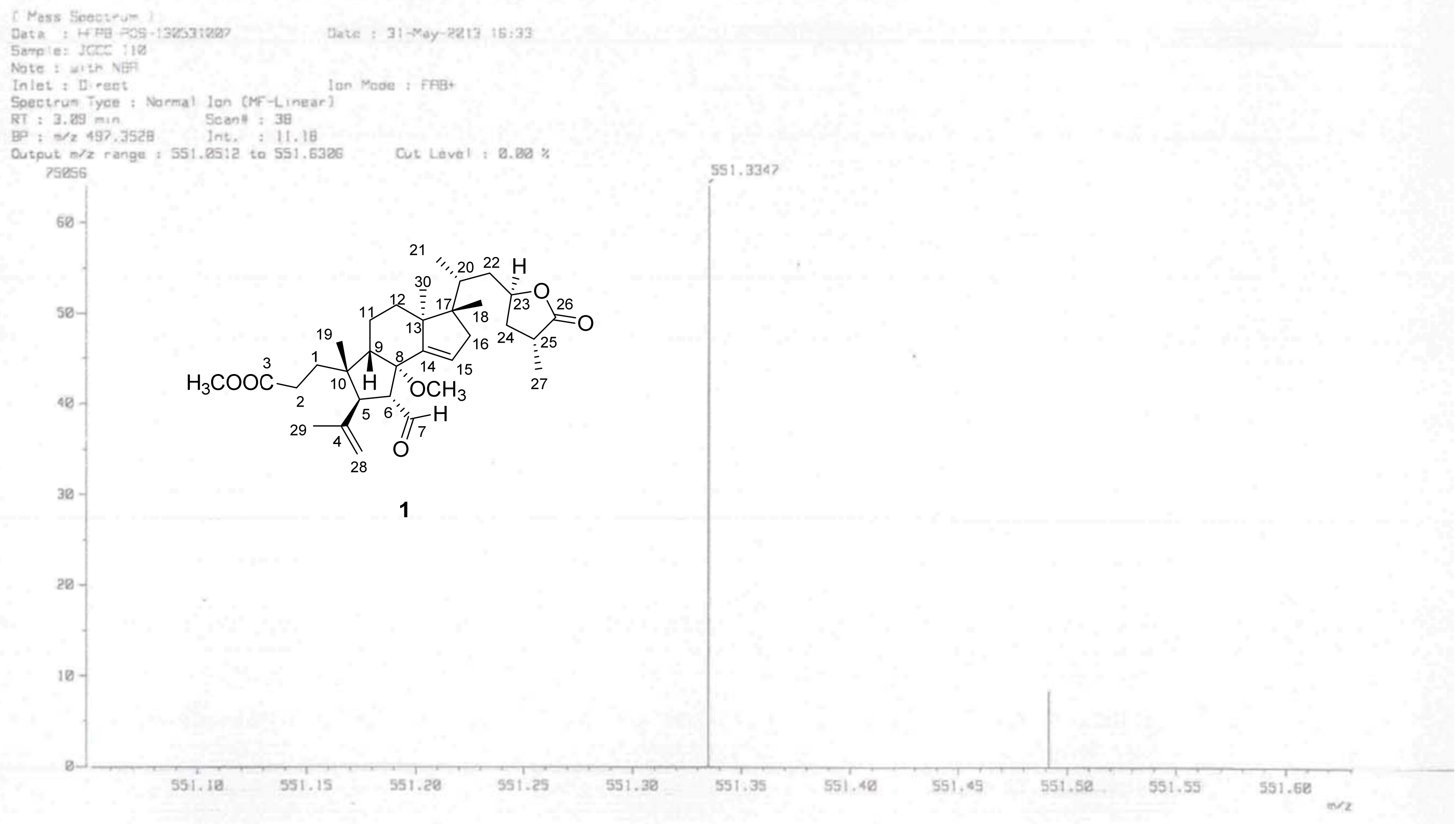

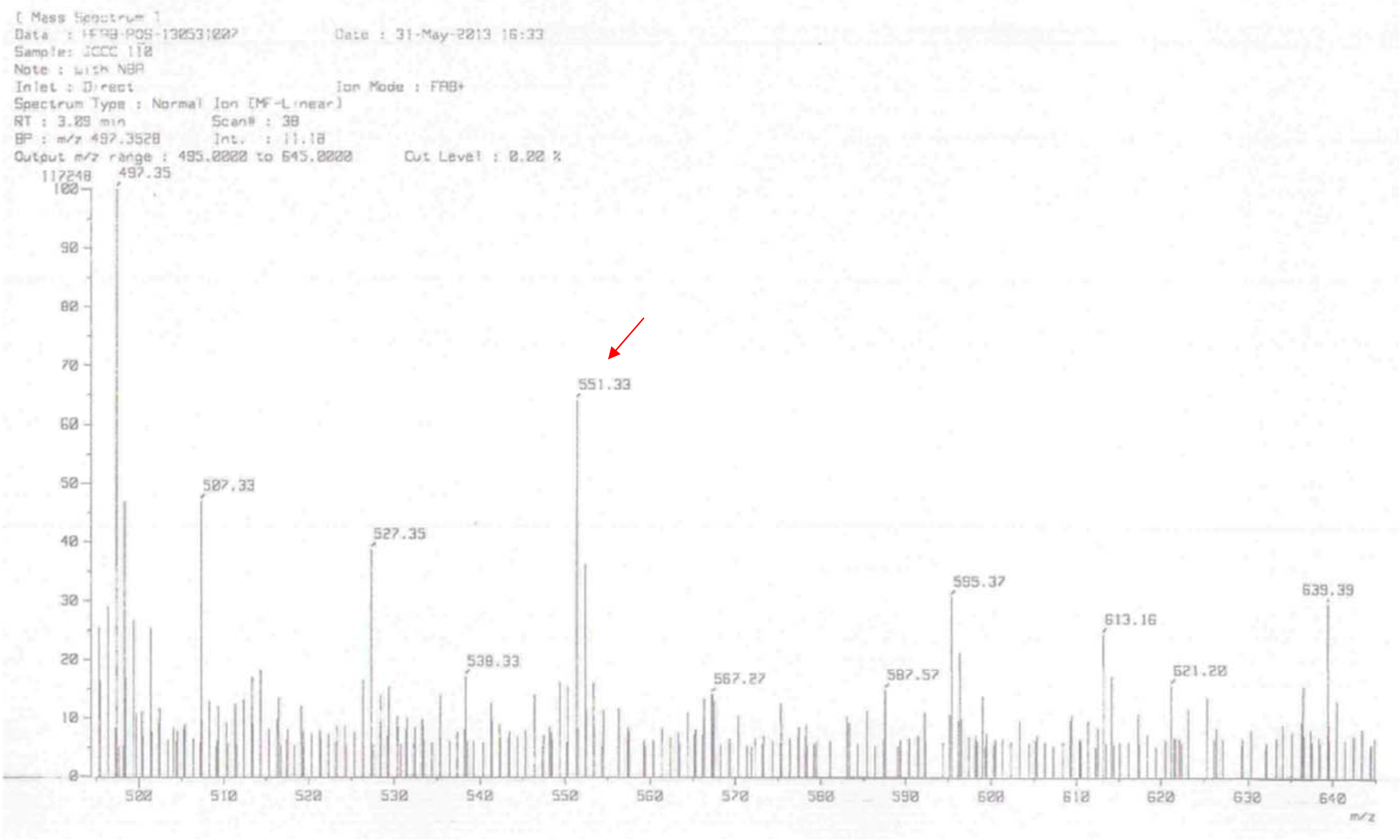
[Mass Spectrum]
Date : 31-May-2013 16:33
Note : with NBA
Inlet : Direct
Ion Mode : FAB+
Spectrum Type : Normal Ion [MF-Linear]
RT : 3.09 min
Scan# : 38
BP : m/z 497.3528
Int. : 11.18
Output m/z range : 495.0000 to 645.0000
Cut Level : 0.00 %
117248
497.35
507.33
527.35
538.33
551.33
567.27
587.57
595.37
613.16
621.20
639.39
m/z

Figure S6. ^{1}H NMR spectrum of 1

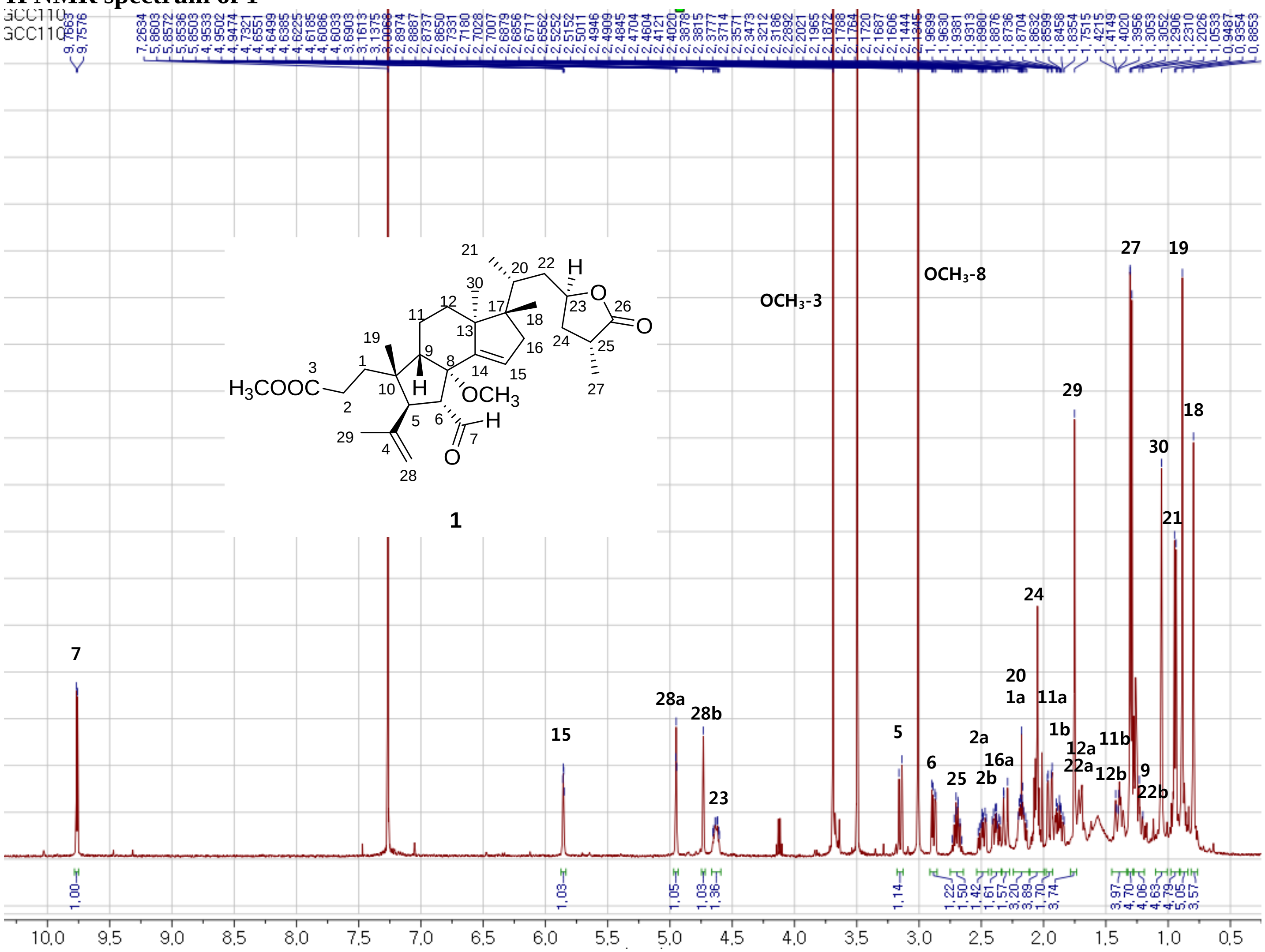

Figure S7. ^{13}C NMR spectrum of 1

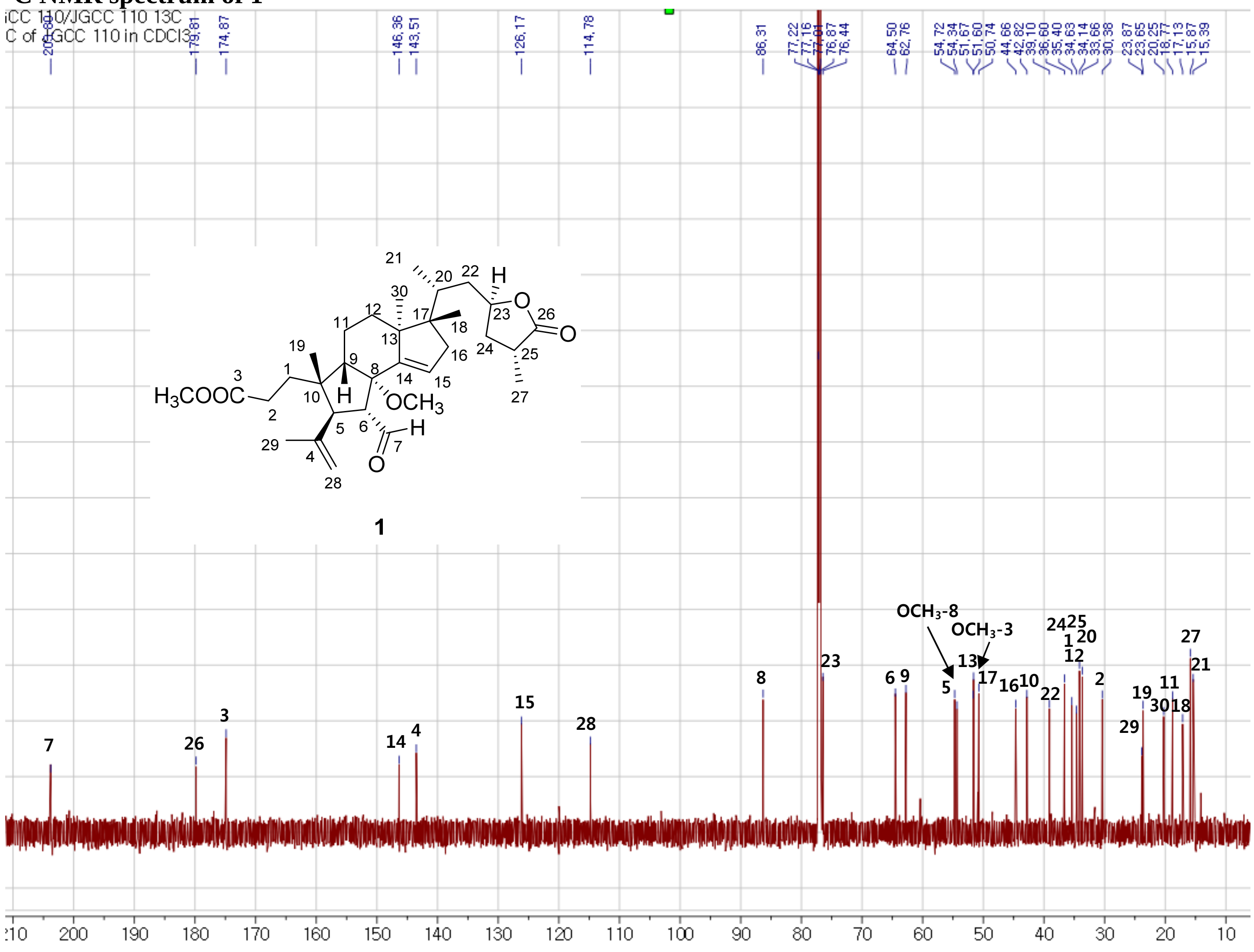

Figure S8. DEPT 90 spectrum of 1

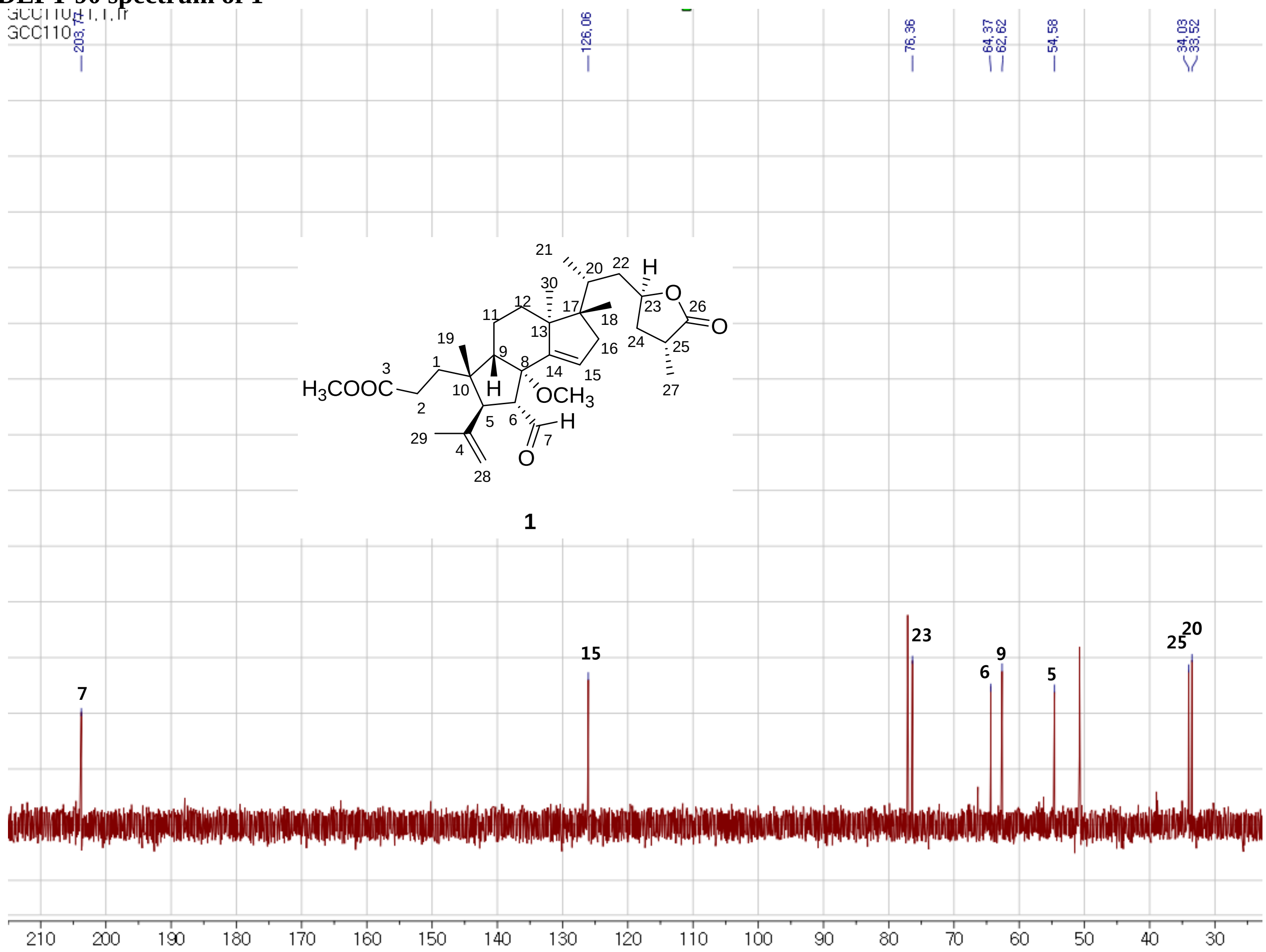

Figure S9. DEPT 135 spectrum of 1

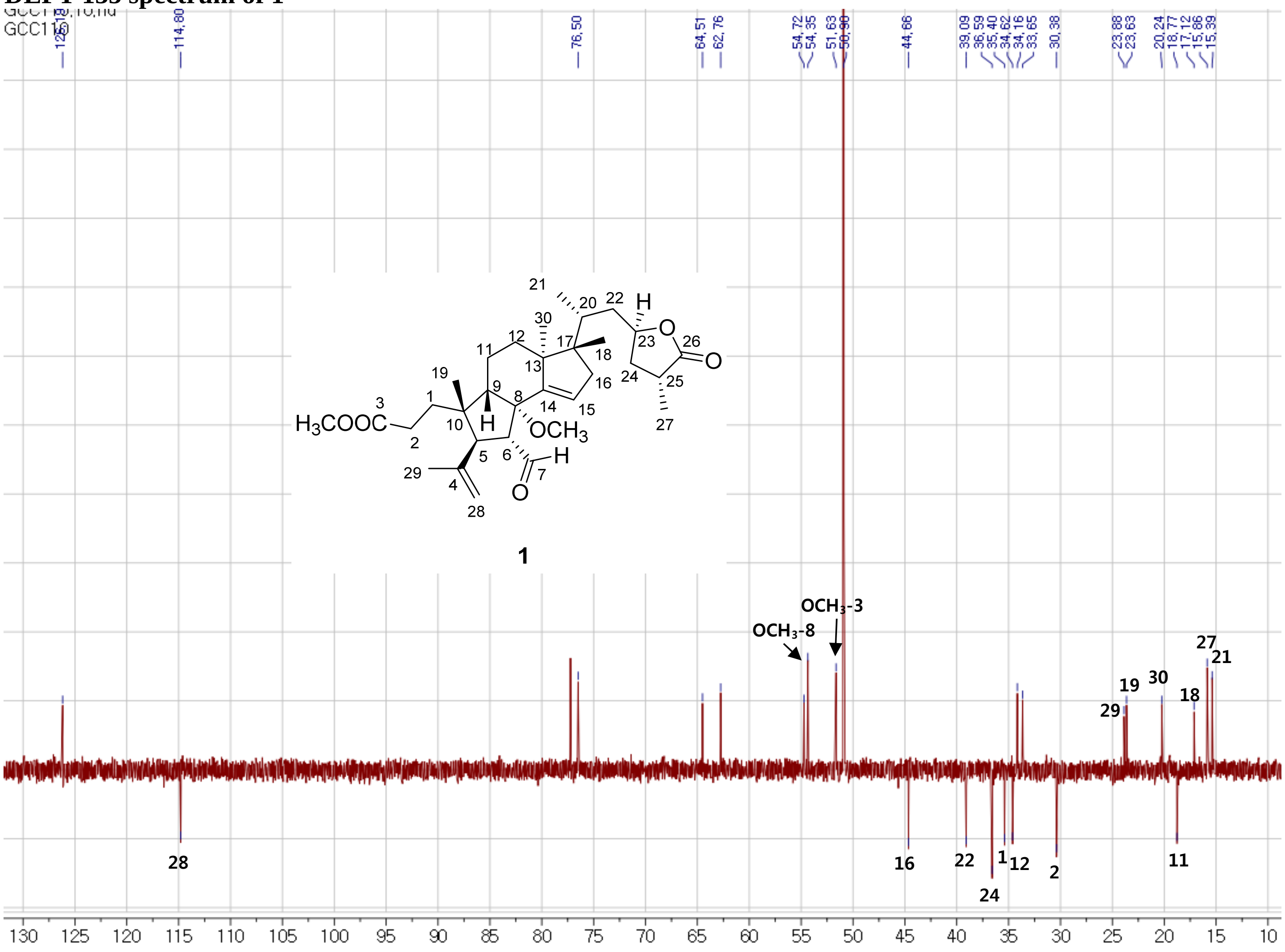

Figure S10. COSY spectrum of 1

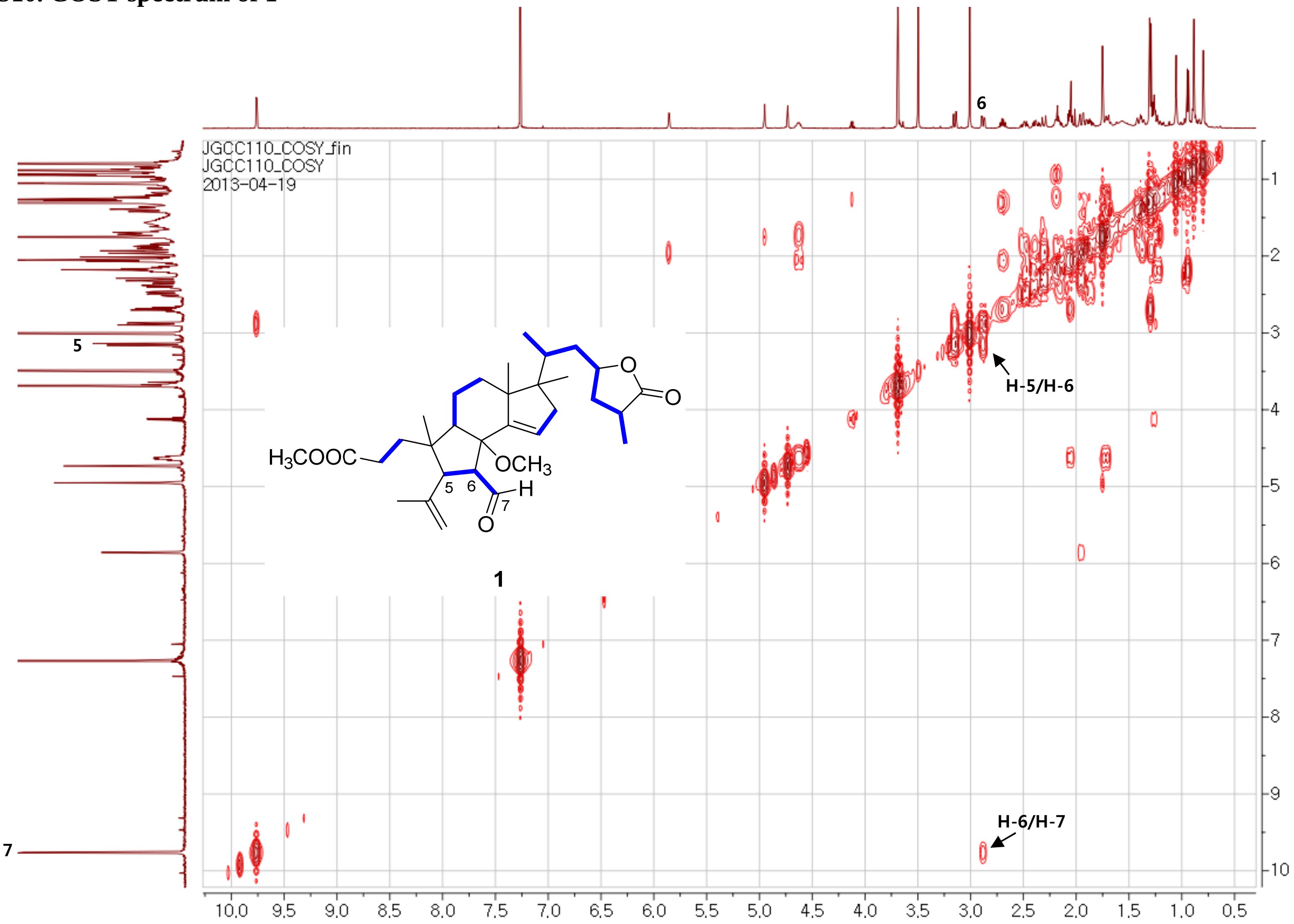

Figure S11. HSQC spectrum of 1

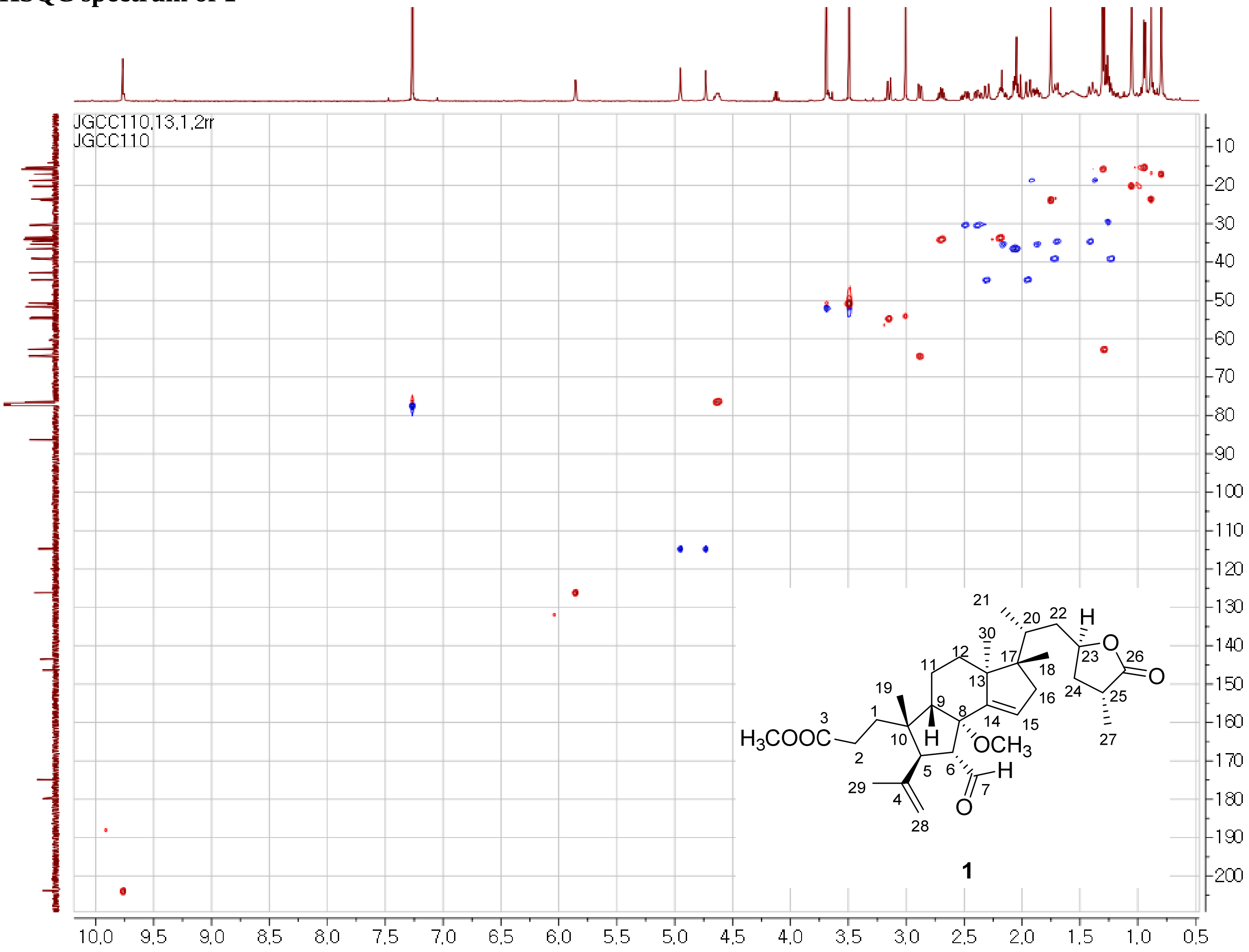

Figure S12. HMBC spectrum of 1

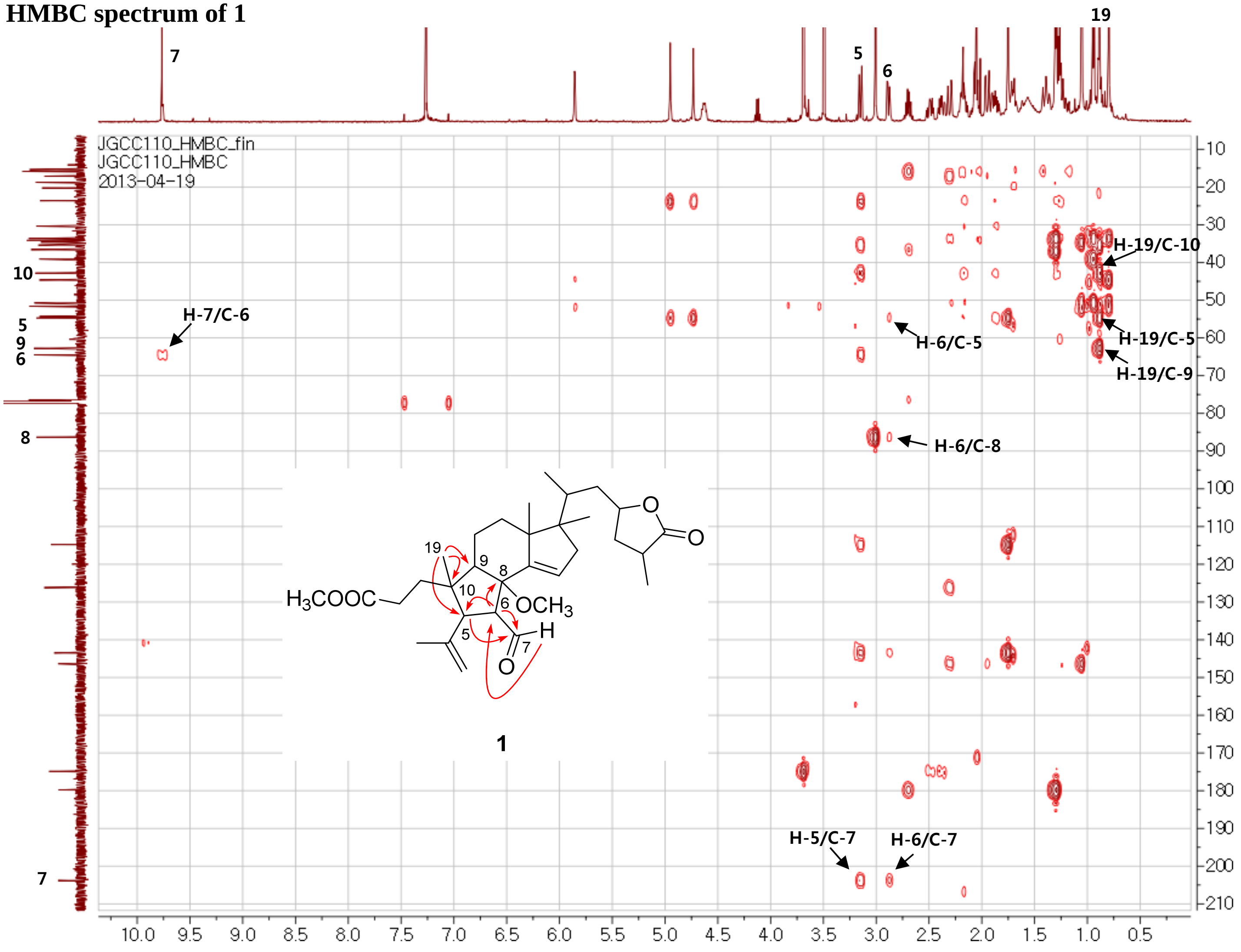

Figure S13. NOESY spectrum of 1

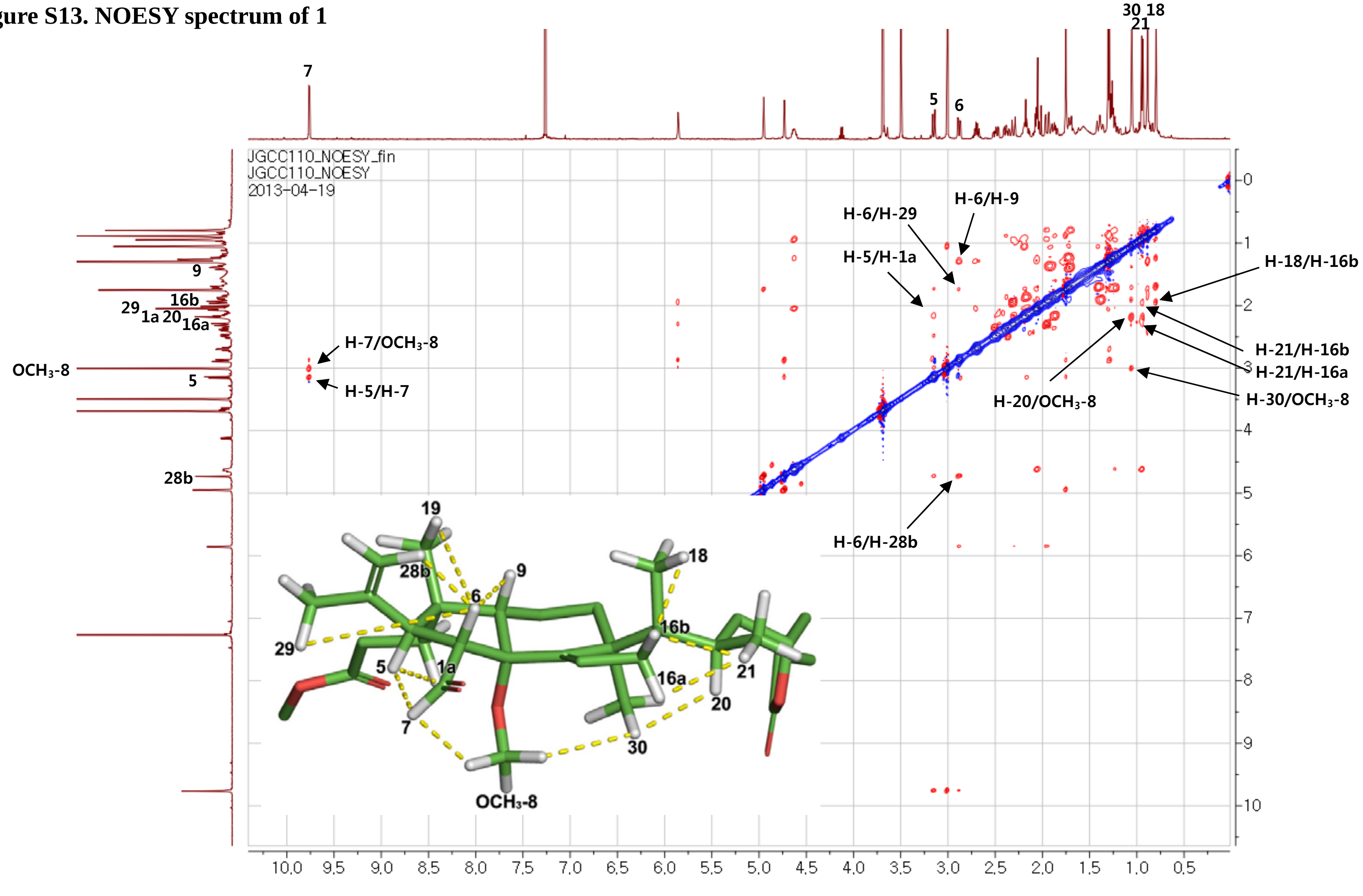

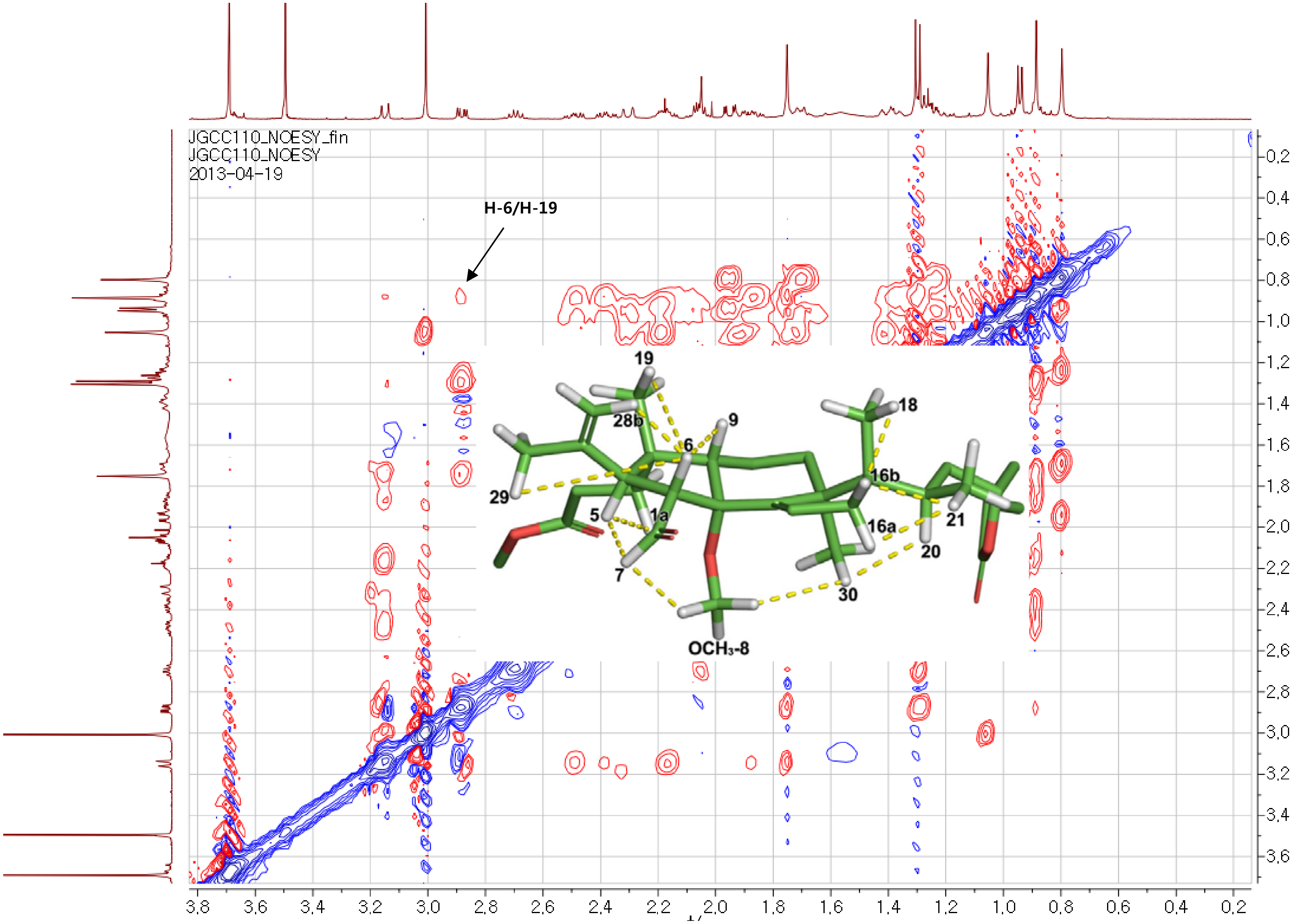

JGCC110_NOESY_fin
JGCC110_NOESY
2013-04-19
H-6/H-19
19
28b
9
18
6
16b
29
5
1a
21
16a
20
7
30
OCH₃-8

Figure S14. HRFABMS spectra of 2

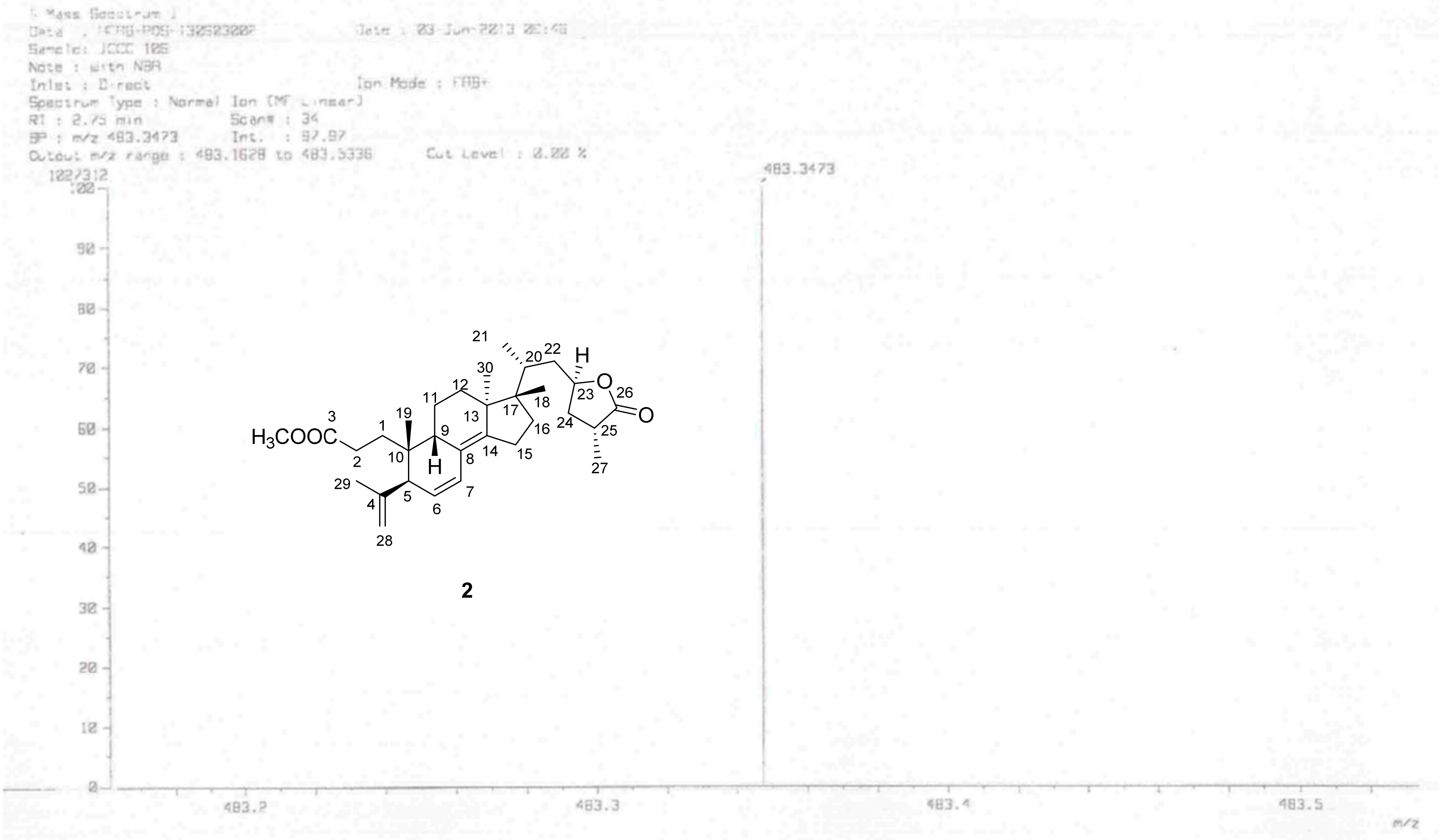

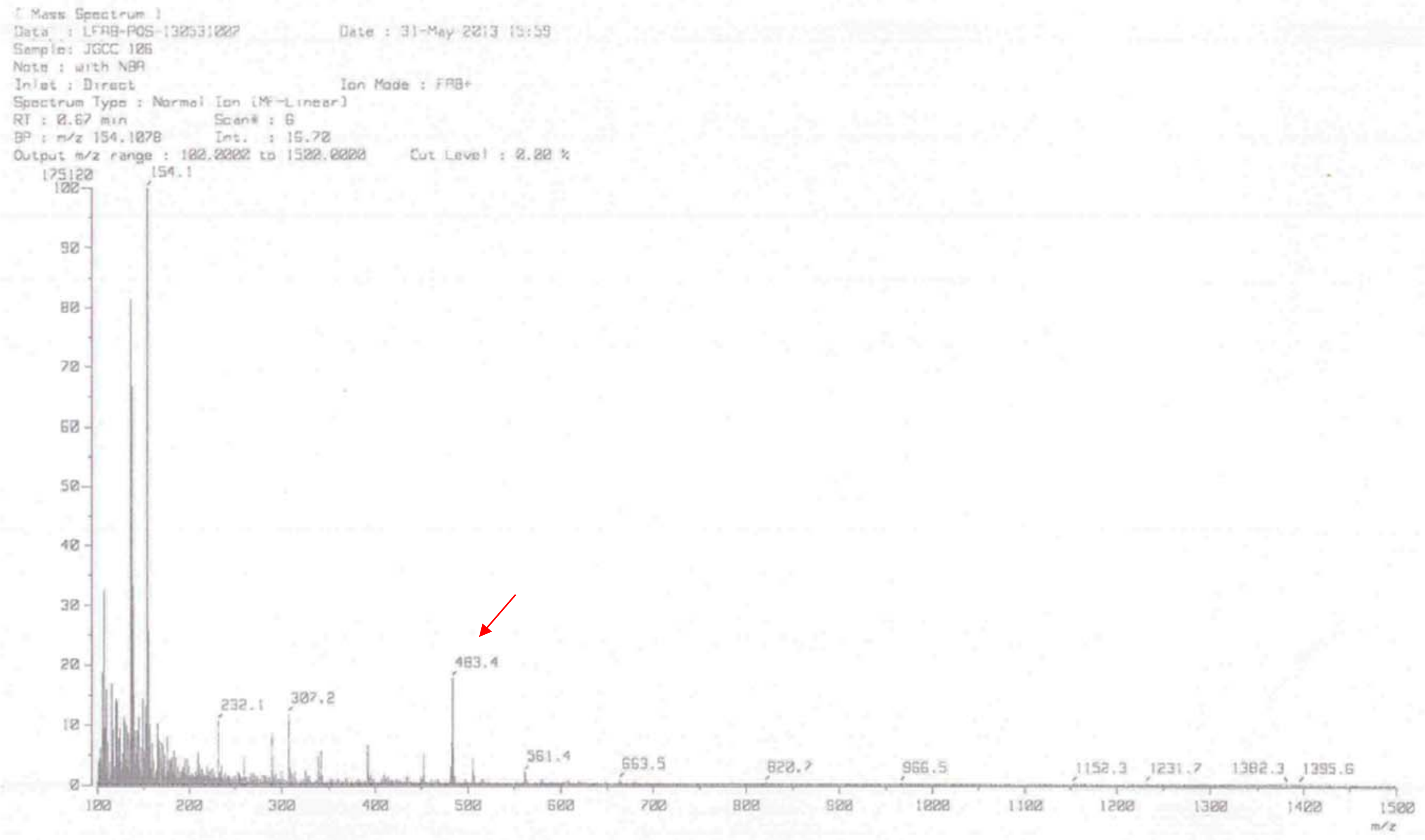
[Mass Spectrum]
Data : LFAB-POS-13053100?
Date : 31-May-2013 15:59
Sample: JGCC 106
Note : with NBA
Inlet : Direct
Ion Mode : FAB+
Spectrum Type : Normal Ion [MF-Linear]
RT : 0.67 min
Scan# : 6
BP : m/z 154.1078
Int. : 15.72
Output m/z range : 100.0000 to 1500.0000
Cut Level : 0.00 %
175120
154.1
232.1
307.2
483.4
561.4
663.5
820.7
966.5
1152.3
1231.7
1382.3
1395.6
m/z

Figure S15. ^{1}H NMR spectrum of 2

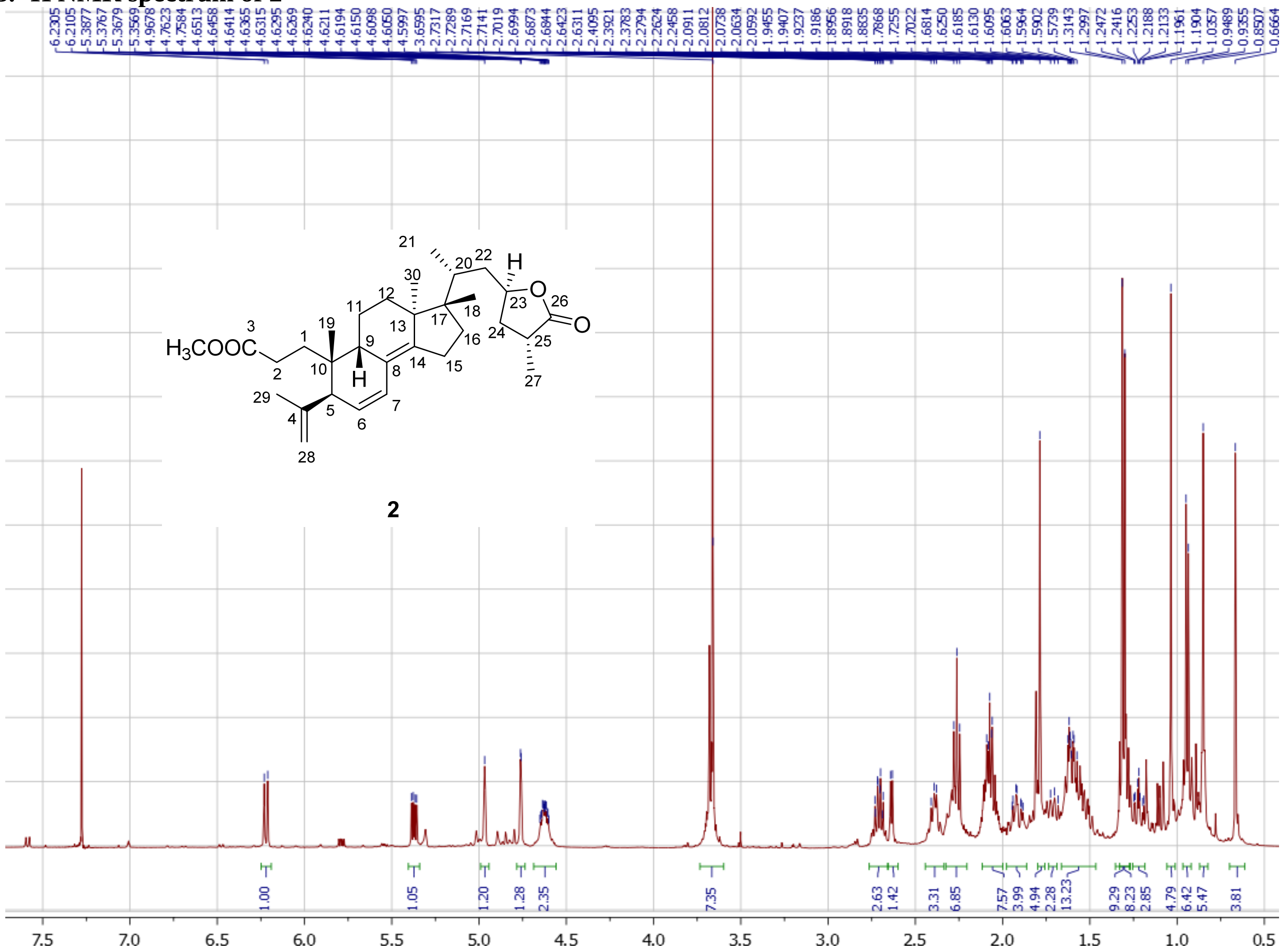

Figure S16. ^{13}C **NMR spectrum of 2**

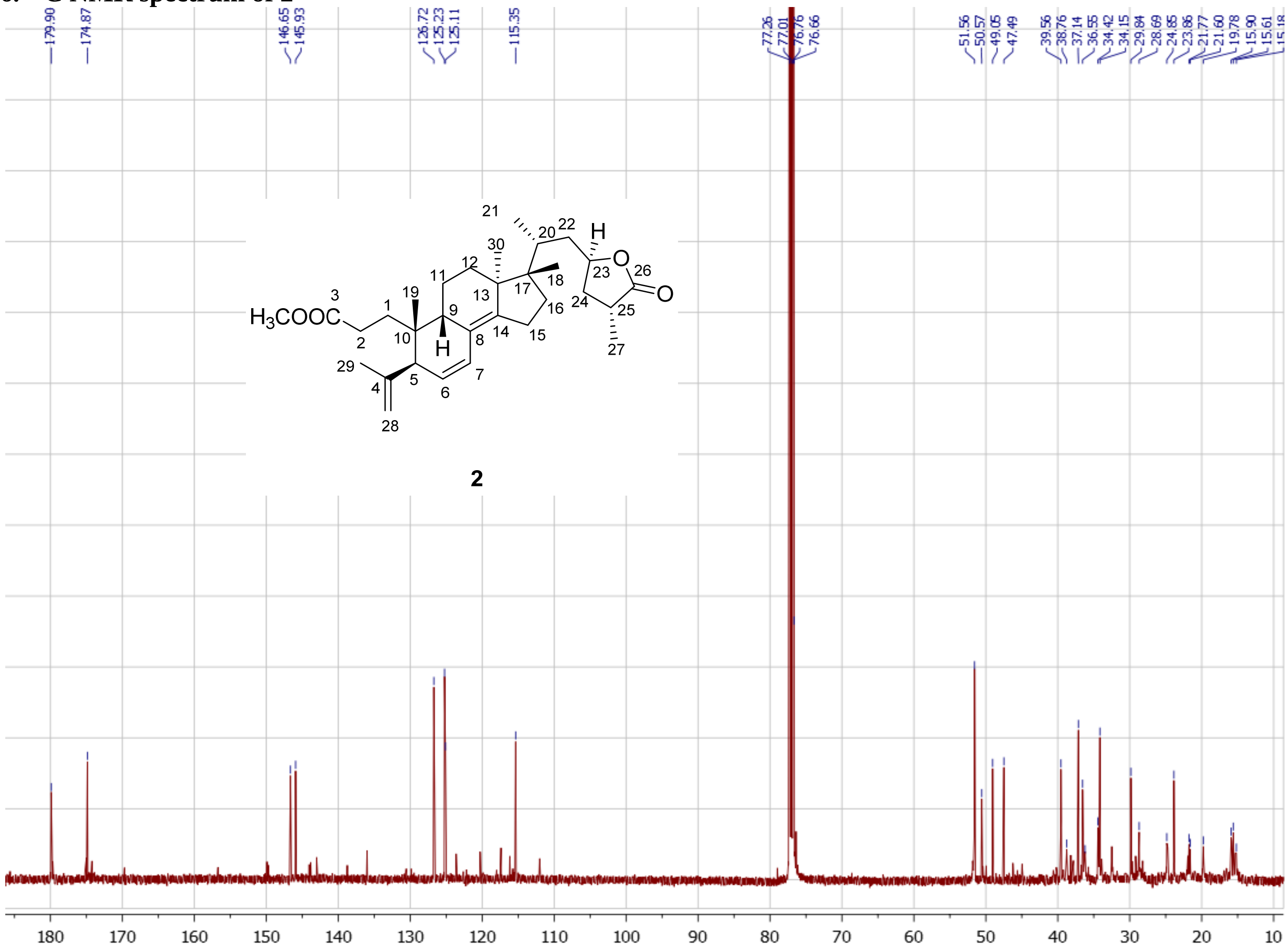

Figure S17. COSY spectrum of 2

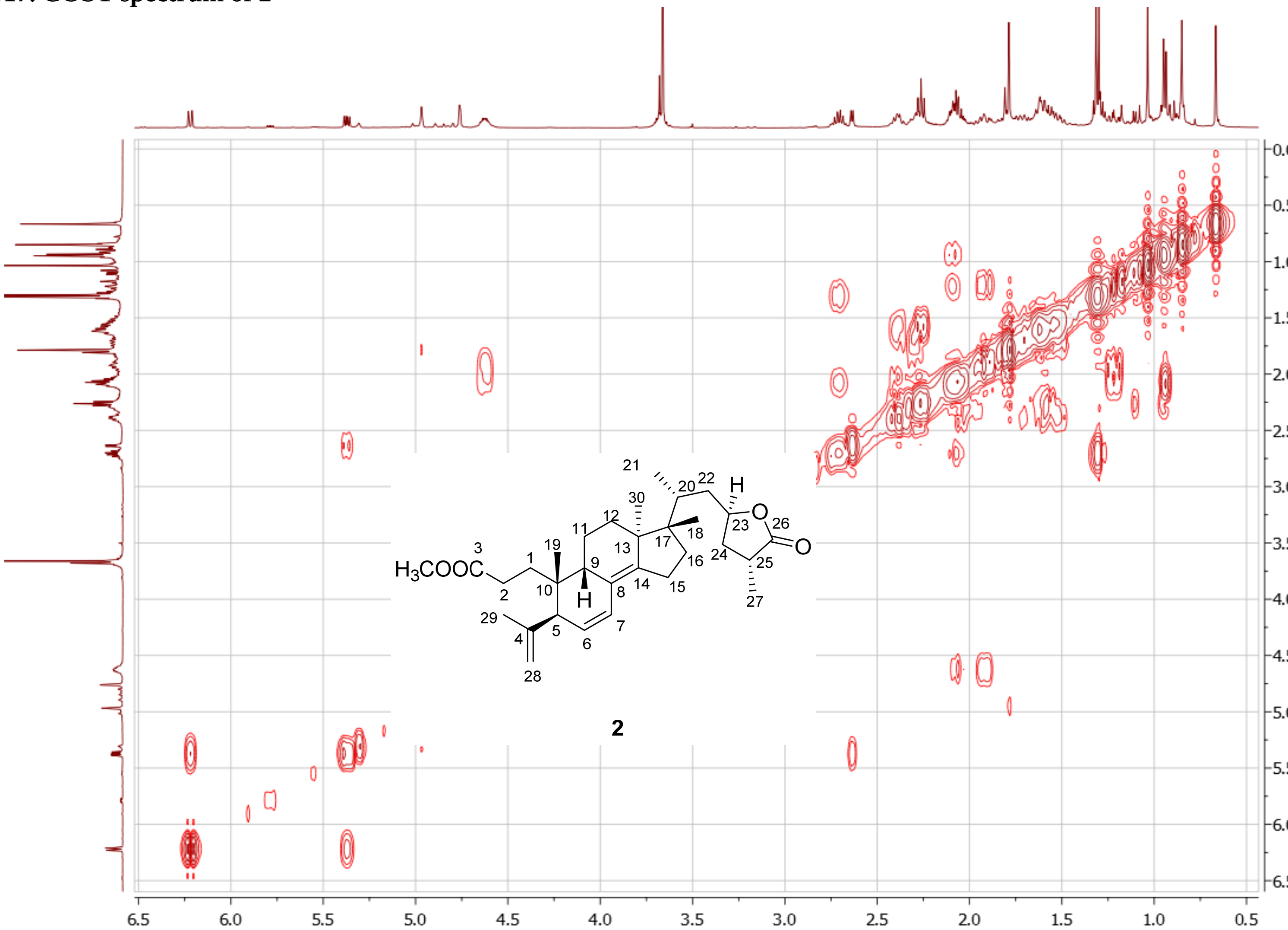

Figure S18. HSQC spectrum of 2

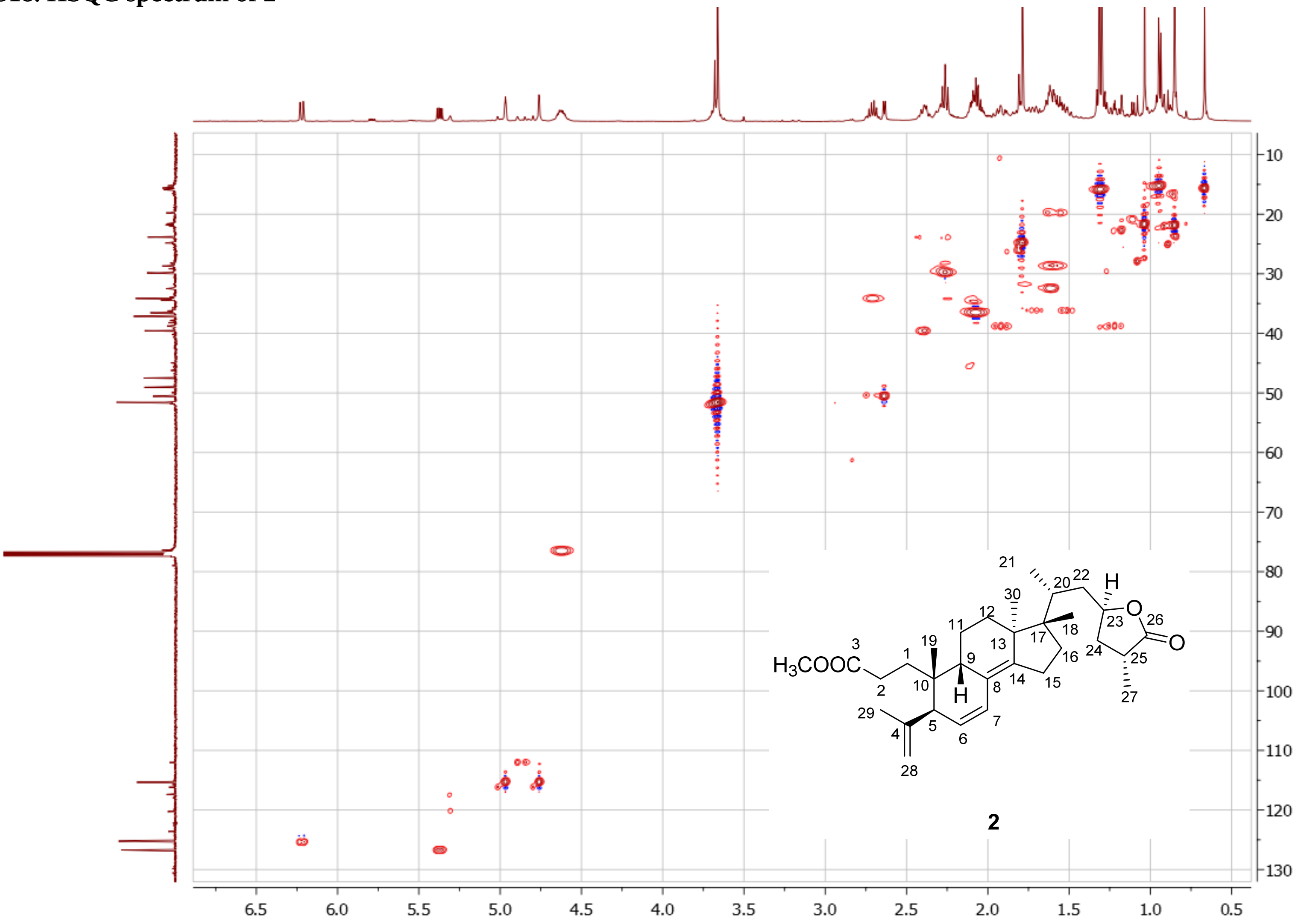

Figure S19. HMBC spectrum of 2

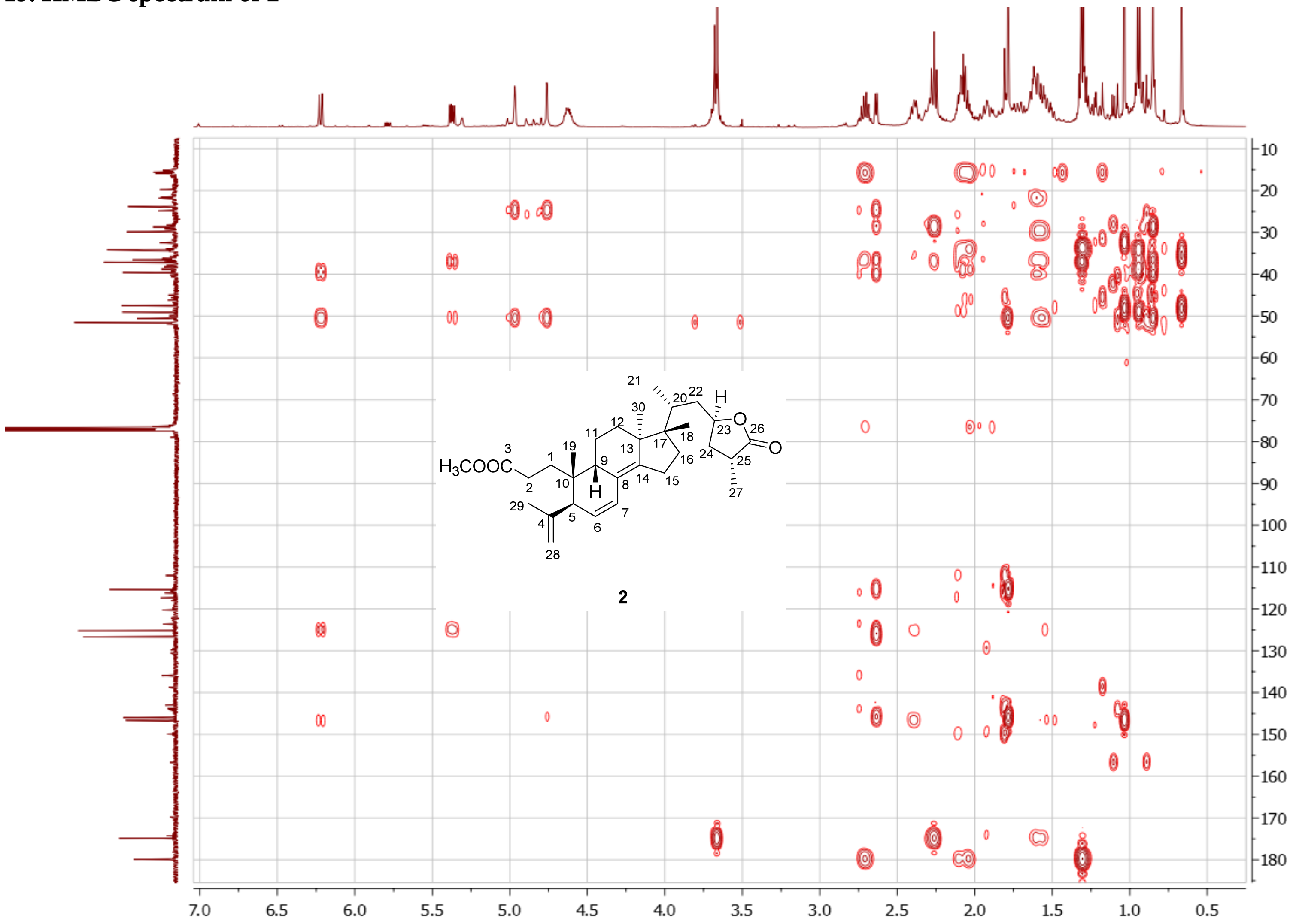

Figure S20. NOESY spectrum of 2

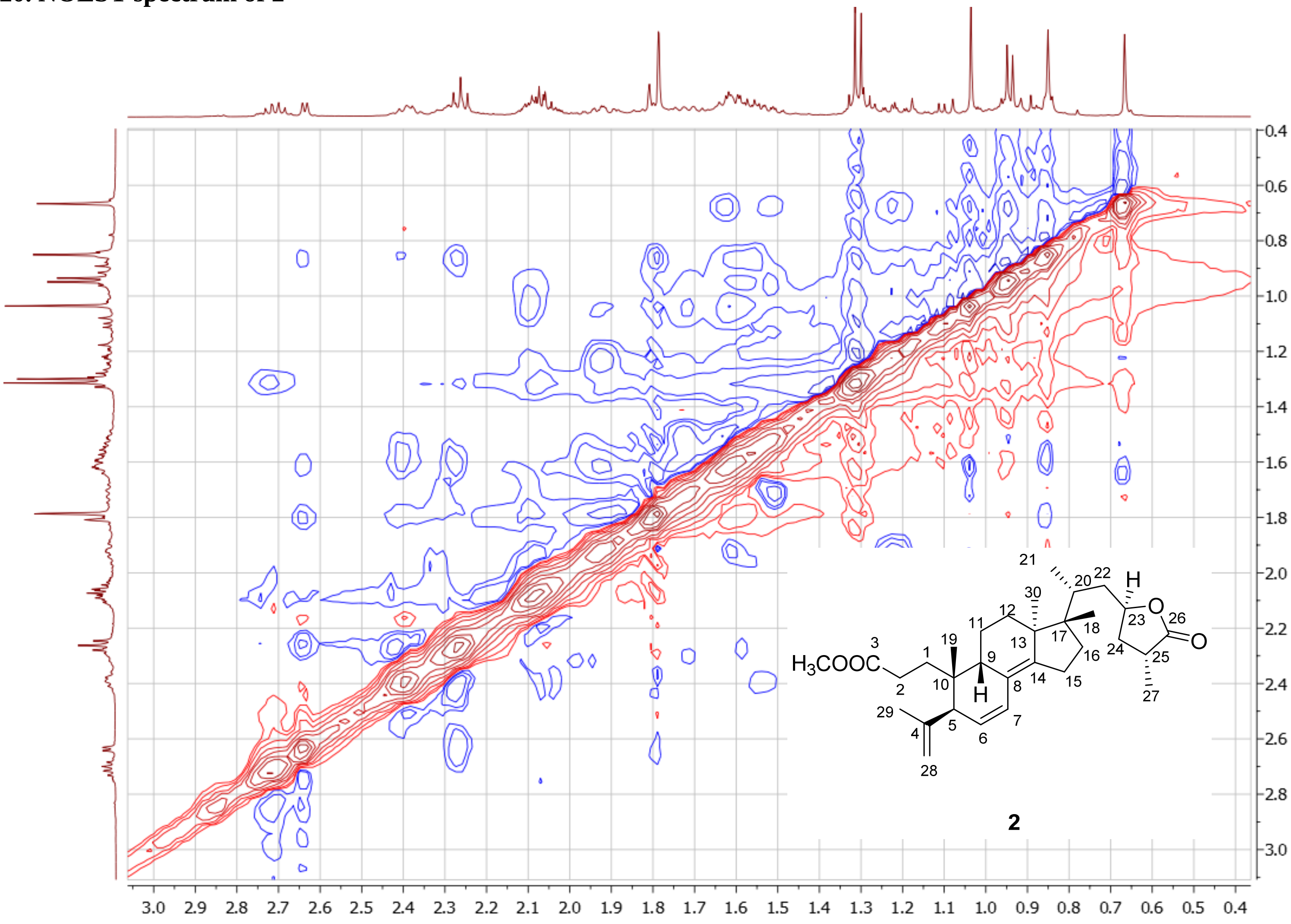

Table S1. Computed thermodynamic data and zero-point vibrational energy of conformers of 1 (PCM, $CHCl_3$) used for chemical shift values and ECD simulation (B3LYP/6-31+(d,p))

Conformer	E_{zpe}	U	H	G
1	-1698.994501	-1698.953878	-1698.952934	-1699.070035
2	-1698.99481	-1698.954304	-1698.95336	-1699.069267
3	-1698.994004	-1698.953515	-1698.95257	-1699.068612
4	-1698.993721	-1698.953367	-1698.952422	-1699.067488
5	-1698.993743	-1698.953287	-1698.952343	-1699.068117
6	-1698.99481	-1698.954304	-1698.95336	-1699.069266
7	-1698.993078	-1698.952547	-1698.951603	-1699.067593
8	-1698.994546	-1698.954063	-1698.953119	-1699.069311
9	-1698.9942	-1698.953798	-1698.952854	-1699.068609
10	-1698.994004	-1698.953515	-1698.952571	-1699.068606
11	-1698.993982	-1698.95355	-1698.952606	-1699.068543
12	-1698.993767	-1698.953294	-1698.95235	-1699.068211
13	-1698.993741	-1698.953287	-1698.952342	-1699.068091
14	-1698.993721	-1698.953367	-1698.952422	-1699.067489
15	-1698.994313	-1698.953751	-1698.952807	-1699.069012
16	-1698.993599	-1698.953133	-1698.952189	-1699.067913
17	-1698.994771	-1698.954274	-1698.95333	-1699.068961
18	-1698.993763	-1698.953401	-1698.952457	-1699.067138
19	-1698.994017	-1698.953514	-1698.95257	-1699.068669
20	-1698.993765	-1698.95326	-1698.952316	-1699.068196
21	-1698.993474	-1698.952937	-1698.951993	-1699.068435
22	-1698.994771	-1698.954274	-1698.95333	-1699.068961
23	-1698.991266	-1698.950749	-1698.949804	-1699.065602
24	-1698.993078	-1698.952547	-1698.951603	-1699.067593
25	-1698.994527	-1698.954042	-1698.953097	-1699.068907
26	-1698.993956	-1698.953532	-1698.952588	-1699.068013
27	-1698.993047	-1698.952516	-1698.951571	-1699.067424
28	-1698.993982	-1698.95355	-1698.952606	-1699.068543
29	-1698.994293	-1698.953812	-1698.952868	-1699.068594
30	-1698.991823	-1698.951509	-1698.950565	-1699.065316
31	-1698.993743	-1698.953287	-1698.952343	-1699.068118
32	-1698.992099	-1698.951788	-1698.950844	-1699.065945
33	-1698.99376	-1698.953264	-1698.95232	-1699.067787
34	-1698.993112	-1698.952613	-1698.951668	-1699.067413
35	-1698.993763	-1698.953401	-1698.952457	-1699.067137
36	-1698.993577	-1698.953079	-1698.952134	-1699.067519
37	-1698.994546	-1698.954063	-1698.953119	-1699.069312
38	-1698.993537	-1698.952932	-1698.951988	-1699.068444
39	-1698.993743	-1698.953287	-1698.952343	-1699.06812
40	-1698.993868	-1698.953213	-1698.952268	-1699.068652
41	-1698.991223	-1698.950778	-1698.949833	-1699.065069

42	-1698.991397	-1698.951115	-1698.950171	-1699.064753
43	-1698.991881	-1698.951496	-1698.950551	-1699.065722
44	-1698.989261	-1698.948951	-1698.948007	-1699.062882
45	-1698.993956	-1698.953532	-1698.952588	-1699.068014
46	-1698.994526	-1698.954042	-1698.953097	-1699.068905
47	-1698.992463	-1698.951884	-1698.95094	-1699.06701
48	-1698.993868	-1698.953213	-1698.952268	-1699.068652
49	-1698.991774	-1698.951187	-1698.950243	-1699.06654
50	-1698.992998	-1698.952368	-1698.951424	-1699.06767
51	-1698.993764	-1698.95326	-1698.952316	-1699.068194
52	-1698.992794	-1698.952393	-1698.951449	-1699.066465
53	-1698.991763	-1698.951073	-1698.950128	-1699.066546
54	-1698.991543	-1698.951108	-1698.950164	-1699.065693
55	-1698.99219	-1698.95182	-1698.950876	-1699.066227
56	-1698.99161	-1698.951067	-1698.950123	-1699.066114
57	-1698.993223	-1698.952655	-1698.951711	-1699.068107
58	-1698.992297	-1698.951968	-1698.951024	-1699.065288

E_{zpe}, U, H, and G stand for zero-point energy, thermal energy, thermal enthalpy and Gibbs free energy, respectively.

Table S2. Boltzmann populations of conformers shown in Table S1.

Conformer	E_{zpe}	U	H	G
1	3.7%	3.2%	3.2%	10.9%
2	5.1%	5.0%	5.0%	4.9%
3	2.2%	2.2%	2.2%	2.4%
4	1.6%	1.9%	1.8%	0.7%
5	1.6%	1.7%	1.7%	1.4%
6	5.1%	5.0%	5.0%	4.8%
7	0.8%	0.8%	0.8%	0.8%
8	3.9%	3.9%	3.9%	5.1%
9	2.7%	2.9%	2.9%	2.4%
10	2.2%	2.2%	2.2%	2.4%
11	2.1%	2.2%	2.2%	2.3%
12	1.7%	1.7%	1.7%	1.6%
13	1.6%	1.7%	1.7%	1.4%
14	1.6%	1.9%	1.8%	0.7%
15	3.0%	2.8%	2.8%	3.7%
16	1.4%	1.4%	1.4%	1.2%
17	4.9%	4.8%	4.8%	3.5%
18	1.7%	1.9%	1.9%	0.5%
19	2.2%	2.2%	2.2%	2.6%
20	1.7%	1.7%	1.7%	1.6%
21	1.2%	1.2%	1.2%	2.0%
22	4.9%	4.8%	4.8%	3.5%
23	0.1%	0.1%	0.1%	0.1%
24	0.8%	0.8%	0.8%	0.8%
25	3.8%	3.8%	3.8%	3.3%
26	2.1%	2.2%	2.2%	1.3%
27	0.8%	0.8%	0.8%	0.7%
28	2.1%	2.2%	2.2%	2.3%
29	2.9%	3.0%	3.0%	2.4%
30	0.2%	0.3%	0.3%	0.1%
31	1.6%	1.7%	1.7%	1.4%
32	0.3%	0.3%	0.3%	0.1%
33	1.7%	1.7%	1.7%	1.0%
34	0.8%	0.8%	0.8%	0.7%
35	1.7%	1.9%	1.9%	0.5%
36	1.4%	1.4%	1.4%	0.8%
37	3.9%	3.9%	3.9%	5.1%
38	1.3%	1.2%	1.2%	2.0%
39	1.6%	1.7%	1.7%	1.4%
40	1.9%	1.6%	1.6%	2.5%
41	0.1%	0.1%	0.1%	0.1%
42	0.1%	0.2%	0.2%	0.0%
43	0.2%	0.3%	0.3%	0.1%
44	0.0%	0.0%	0.0%	0.0%
45	2.1%	2.2%	2.2%	1.3%
46	3.8%	3.8%	3.8%	3.3%
47	0.4%	0.4%	0.4%	0.4%
48	1.9%	1.6%	1.6%	2.5%
49	0.2%	0.2%	0.2%	0.3%
50	0.7%	0.6%	0.6%	0.9%
51	1.7%	1.7%	1.7%	1.6%
52	0.6%	0.7%	0.7%	0.2%
53	0.2%	0.2%	0.2%	0.3%
54	0.2%	0.2%	0.2%	0.1%
55	0.3%	0.4%	0.4%	0.2%
56	0.2%	0.2%	0.2%	0.2%

57	0.9%	0.9%	0.9%	1.4%
58	0.4%	0.4%	0.4%	0.1%

Table S3. Calculated thermodynamic data and zero-point vibrational energy of conformers of 1 (gas phase) employed for chemical shift values and ECD simulation at the B3LYP/6-311+(2d,p) level

Conformer	E_{zpe}	U	H	G
1	-1699.368641	-1699.327891	-1699.326947	-1699.444577
2	-1699.368936	-1699.328404	-1699.32746	-1699.443481
3	-1699.368073	-1699.327447	-1699.326502	-1699.444741
4	-1699.368221	-1699.327728	-1699.326784	-1699.442408
5	-1699.367829	-1699.327197	-1699.326253	-1699.442967
6	-1699.368936	-1699.328404	-1699.32746	-1699.443481
7	-1699.368221	-1699.327728	-1699.326784	-1699.442409
8	-1699.368902	-1699.328219	-1699.327275	-1699.444321
9	-1699.368344	-1699.327739	-1699.326795	-1699.444072
10	-1699.368073	-1699.327447	-1699.326502	-1699.444746
11	-1699.368164	-1699.327533	-1699.326589	-1699.443503
12	-1699.367689	-1699.32722	-1699.326275	-1699.442275
13	-1699.367828	-1699.327197	-1699.326253	-1699.442965
14	-1699.368221	-1699.327728	-1699.326784	-1699.442406
15	-1699.368379	-1699.327747	-1699.326802	-1699.443647
16	-1699.367881	-1699.327257	-1699.326312	-1699.443019
17	-1699.368962	-1699.328419	-1699.327474	-1699.44353
18	-1699.36814	-1699.327695	-1699.326751	-1699.442089
19	-1699.368	-1699.327442	-1699.326498	-1699.443189
20	-1699.367762	-1699.327182	-1699.326238	-1699.442577
21	-1699.367308	-1699.326743	-1699.325799	-1699.442843
22	-1699.368962	-1699.328418	-1699.327474	-1699.443528
23	-1699.366089	-1699.325628	-1699.324684	-1699.439542
24	-1699.368221	-1699.327728	-1699.326784	-1699.442409
25	-1699.368714	-1699.32811	-1699.327166	-1699.443571
26	-1699.368081	-1699.327542	-1699.326598	-1699.442775
27	-1699.36814	-1699.327695	-1699.326751	-1699.442089
28	-1699.368164	-1699.327533	-1699.326589	-1699.443505
29	-1699.368417	-1699.327799	-1699.326855	-1699.443914
30	-1699.366199	-1699.325874	-1699.32493	-1699.440017
31	-1699.367828	-1699.327197	-1699.326253	-1699.442968
32	-1699.366785	-1699.326423	-1699.325478	-1699.440731
33	-1699.367746	-1699.327209	-1699.326265	-1699.442502
34	-1699.367836	-1699.327229	-1699.326285	-1699.442462
35	-1699.36814	-1699.327695	-1699.326751	-1699.442091
36	-1699.367742	-1699.327179	-1699.326235	-1699.442159
37	-1699.368902	-1699.328219	-1699.327275	-1699.44432
38	-1699.367366	-1699.326788	-1699.325843	-1699.442635
39	-1699.367829	-1699.327197	-1699.326253	-1699.442968
40	-1699.368052	-1699.327345	-1699.326401	-1699.443022
41	-1699.366268	-1699.325715	-1699.324771	-1699.440527
42	-1699.366267	-1699.325902	-1699.324958	-1699.439889
43	-1699.366427	-1699.326043	-1699.325099	-1699.440591
44	-1699.364234	-1699.32393	-1699.322985	-1699.437867
45	-1699.368081	-1699.327542	-1699.326598	-1699.442775
46	-1699.368714	-1699.32811	-1699.327166	-1699.443571
47	-1699.36695	-1699.32631	-1699.325366	-1699.441539
48	-1699.368052	-1699.327345	-1699.326401	-1699.443022
49	-1699.365718	-1699.325088	-1699.324144	-1699.440566
50	-1699.36648	-1699.325721	-1699.324777	-1699.441934
51	-1699.367762	-1699.327182	-1699.326238	-1699.442577
52	-1699.36682	-1699.32627	-1699.325326	-1699.441654
53	-1699.368714	-1699.32811	-1699.327166	-1699.443571
54	-1699.365654	-1699.325166	-1699.324222	-1699.43998

55	-1699.36682	-1699.326398	-1699.325454	-1699.44099
56	-1699.36695	-1699.32631	-1699.325366	-1699.441538
57	-1699.367825	-1699.327185	-1699.326241	-1699.442499
58	-1699.366508	-1699.325919	-1699.324975	-1699.441411

Table S4. Boltzmann populations of conformers described in Table S3

Conformer	E_{zpe}	U	H	G
1	3.2%	2.7%	2.7%	7.6%
2	4.4%	4.7%	4.7%	2.4%
3	1.8%	1.7%	1.7%	9.1%
4	2.1%	2.3%	2.3%	0.8%
5	1.4%	1.3%	1.3%	1.4%
6	4.4%	4.7%	4.7%	2.4%
7	2.1%	2.3%	2.3%	0.8%
8	4.3%	3.8%	3.8%	5.8%
9	2.4%	2.3%	2.3%	4.5%
10	1.8%	1.7%	1.7%	9.1%
11	2.0%	1.8%	1.8%	2.4%
12	1.2%	1.3%	1.3%	0.7%
13	1.4%	1.3%	1.3%	1.4%
14	2.1%	2.3%	2.3%	0.8%
15	2.5%	2.3%	2.3%	2.8%
16	1.4%	1.4%	1.4%	1.5%
17	4.5%	4.7%	4.7%	2.5%
18	1.9%	2.2%	2.2%	0.5%
19	1.6%	1.7%	1.7%	1.8%
20	1.3%	1.3%	1.3%	0.9%
21	0.8%	0.8%	0.8%	1.2%
22	4.5%	4.7%	4.7%	2.5%
23	0.2%	0.2%	0.2%	0.0%
24	2.1%	2.3%	2.3%	0.8%
25	3.5%	3.4%	3.4%	2.6%
26	1.8%	1.9%	1.9%	1.1%
27	1.9%	2.2%	2.2%	0.5%
28	2.0%	1.8%	1.8%	2.4%
29	2.6%	2.5%	2.5%	3.8%
30	0.2%	0.3%	0.3%	0.1%
31	1.4%	1.3%	1.3%	1.4%
32	0.5%	0.6%	0.6%	0.1%
33	1.3%	1.3%	1.3%	0.8%
34	1.4%	1.3%	1.3%	0.8%
35	1.9%	2.2%	2.2%	0.5%
36	1.2%	1.3%	1.3%	0.6%
37	4.3%	3.8%	3.8%	5.8%
38	0.8%	0.8%	0.8%	1.0%
39	1.4%	1.3%	1.3%	1.4%
40	1.7%	1.5%	1.5%	1.5%
41	0.3%	0.3%	0.3%	0.1%
42	0.3%	0.3%	0.3%	0.1%
43	0.3%	0.4%	0.4%	0.1%
44	0.0%	0.0%	0.0%	0.0%
45	1.8%	1.9%	1.9%	1.1%
46	3.5%	3.4%	3.4%	2.6%
47	0.5%	0.5%	0.5%	0.3%
48	1.7%	1.5%	1.5%	1.5%
49	0.1%	0.1%	0.1%	0.1%
50	0.3%	0.3%	0.3%	0.5%
51	1.3%	1.3%	1.3%	0.9%
52	0.5%	0.5%	0.5%	0.3%
53	3.5%	3.4%	3.4%	2.6%

54	0.1%	0.2%	0.2%	0.1%
55	0.5%	0.6%	0.6%	0.2%
56	0.5%	0.5%	0.5%	0.3%
57	1.4%	1.3%	1.3%	0.8%
58	0.3%	0.3%	0.3%	0.3%

Table S5. Experimental and PCM calculation ($CHCl_3$) for ^{13}C chemical shift values of compound 1 at B3LYP/6-31G+(d,p) and B3LYP/6-311G+(2d,p)

	Experimental	B3LYP/6-31G+(d,p)	B3LYP/6-311G+(2d,p)
C-12	34.60	37.18	38.62
C-11	18.80	23.8	24.44
C-5	54.70	55.81	61.33
C-6	64.50	65.6	70.33
C-8	86.30	90.99	95.75
C-9	62.80	64.79	69.57
C-7	203.80	203.86	215.82
C-16	44.70	47.32	50.83
C-17	50.70	54.9	60.1
C-13	51.70	57.83	60.76
C-14	146.40	146.28	156.58
C-15	126.20	127.43	135.97
C-18	17.10	17.28	18.52
C-30	20.30	20.93	22.28
C-20	33.70	37.47	40.33
C-22	39.10	41.07	43.16
C-23	76.40	78.75	81.01
C-24	36.60	37.61	40.38
C-25	34.10	37.98	40.37
C-26	179.80	176.29	188.37
C-27	15.90	16.75	17.65
C-10	42.80	50.57	50.32
C-19	23.70	21.89	22.84
C-1	35.40	38.62	40.54
C-2	30.40	31.57	34.84
C-3	174.90	173.36	184.8
OCH_3-3	51.60	52.26	54.93
C-4	143.50	145.54	158.6
C-28	114.80	109.81	118.93
C-29	23.90	25.8	27.66
OCH_3-8	54.30	53.87	56.89
C-21	15.40	15.68	15.96

Coordinates of conformers found at B3LYP/6-311G+(2d,p) in the gas phase
1

Center Number	Atomic Number	Atomic Type	Coordinates (Angstroms) X	Y	Z
1	6	0	-1.066123	0.153046	-1.161120
2	6	0	0.329712	0.796008	-1.055589
3	1	0	-1.092159	-0.403250	-2.098701
4	1	0	-1.815173	0.944596	-1.249149
5	1	0	0.378406	1.501882	-0.225808
6	1	0	0.519497	1.367904	-1.969025
7	6	0	3.553060	-1.177539	-0.169238
8	6	0	2.379814	-2.061994	0.328805
9	6	0	1.172933	-1.075947	0.428031
10	6	0	1.375526	-0.301708	-0.889619
11	6	0	2.773689	-2.859840	1.552945
12	1	0	2.087765	-2.793877	-0.423907
13	8	0	1.344594	-0.119354	1.492233
14	6	0	-2.082305	-3.067777	0.658423
15	6	0	-2.537008	-1.909188	-0.283386
16	6	0	-1.425918	-0.781734	0.026605
17	6	0	-0.220930	-1.657925	0.435500
18	6	0	-0.600502	-2.880927	0.792637
19	1	0	-2.564075	-2.999087	1.642602
20	1	0	-2.346214	-4.050466	0.258633
21	6	0	-2.414293	-2.433595	-1.728227
22	6	0	-1.825174	0.098697	1.234626
23	1	0	0.061539	-3.657291	1.154391
24	1	0	-2.704676	-1.696450	-2.477294
25	1	0	-1.390678	-2.743670	-1.946313
26	1	0	-3.052402	-3.308190	-1.866518
27	1	0	-1.002787	0.758854	1.506292
28	1	0	-2.691200	0.723834	1.013119
29	1	0	-2.066500	-0.502719	2.112926
30	6	0	-4.013929	-1.497557	0.024495
31	6	0	-4.546212	-0.380433	-0.898258
32	6	0	-5.854320	0.267032	-0.447903
33	6	0	-6.295454	1.463833	-1.311354
34	6	0	-5.756439	2.681091	-0.553281
35	6	0	-5.657077	2.171520	0.881646
36	8	0	-5.711941	0.814213	0.894838
37	1	0	-6.644448	-0.478940	-0.373239
38	1	0	-7.386393	1.511109	-1.358156
39	1	0	-5.921328	1.389233	-2.332784
40	1	0	-4.723718	2.884943	-0.860289
41	6	0	-6.565527	3.968503	-0.670984
42	1	0	-4.027612	-1.107439	1.043687
43	1	0	-4.722565	-0.781062	-1.902435
44	1	0	-3.799314	0.407879	-1.011766
45	8	0	-5.535055	2.816533	1.884340
46	1	0	-6.152928	4.738989	-0.018901
47	1	0	-6.554419	4.340091	-1.697775
48	1	0	-7.605664	3.803331	-0.380096
49	1	0	1.166653	-1.044074	-1.669765
50	6	0	2.898559	0.013128	-1.017595

51	6	0	3.282884	-0.016002	-2.504943
52	6	0	3.210435	1.408456	-0.417662
53	1	0	4.344887	0.170015	-2.669942
54	1	0	3.052170	-0.980838	-2.958637
55	1	0	2.727584	0.753720	-3.048331
56	6	0	4.672759	1.851922	-0.408442
57	6	0	4.841518	3.271681	0.090126
58	8	0	3.970492	4.099755	0.182476
59	8	0	6.132561	3.518802	0.408588
60	6	0	6.419720	4.857159	0.851322
61	6	0	4.677272	-1.973688	-0.822230
62	1	0	3.985689	-0.712453	0.723688
63	6	0	4.496939	-3.146125	-1.430816
64	6	0	6.065791	-1.391649	-0.723975
65	1	0	5.333763	-3.674930	-1.872166
66	1	0	3.533366	-3.630279	-1.518338
67	1	0	6.150184	-0.441946	-1.257243
68	1	0	6.332358	-1.191172	0.318984
69	1	0	6.808592	-2.073944	-1.138434
70	1	0	5.113291	1.831334	-1.410817
71	1	0	5.300960	1.208525	0.209101
72	1	0	2.829960	1.444839	0.602478
73	1	0	2.643498	2.155473	-0.976974
74	8	0	2.340070	-3.949904	1.836303
75	1	0	3.546769	-2.390124	2.197488
76	6	0	1.166110	-0.565418	2.828816
77	1	0	1.005587	0.327320	3.433542
78	1	0	0.303475	-1.227117	2.931292
79	1	0	2.055154	-1.081997	3.205838
80	6	0	-4.978894	-2.698539	0.005195
81	1	0	-5.965862	-2.408553	0.370136
82	1	0	-5.109011	-3.105563	-1.000926
83	1	0	-4.634019	-3.506991	0.649676
84	1	0	5.855298	5.088754	1.754231
85	1	0	7.487874	4.874026	1.052441
86	1	0	6.163379	5.578471	0.075674

2

Center Number	Atomic Number	Atomic Type	Coordinates (Angstroms) X	Y	Z
1	6	0	-0.857634	0.150912	-1.032275
2	6	0	0.513337	0.811110	-0.788746
3	1	0	-0.838356	-0.255552	-2.044192
4	1	0	-1.629044	0.925684	-1.023371
5	1	0	0.513717	1.390805	0.135893
6	1	0	0.699455	1.509700	-1.608677
7	6	0	3.760618	-1.143050	0.008584
8	6	0	2.620644	-2.157895	0.239632
9	6	0	1.369749	-1.237009	0.425722
10	6	0	1.596674	-0.263734	-0.748157
11	6	0	2.971126	-3.144490	1.333091
12	1	0	2.415234	-2.752327	-0.652601
13	8	0	1.465163	-0.437990	1.622287
14	6	0	-1.815085	-3.340312	0.228778
15	6	0	-2.286300	-2.061520	-0.531115
16	6	0	-1.221864	-0.964635	-0.014907
17	6	0	0.000009	-1.856815	0.292184
18	6	0	-0.344887	-3.132632	0.436323
19	1	0	-2.321165	-3.443100	1.197412
20	1	0	-2.035667	-4.254317	-0.328998
21	6	0	-2.108414	-2.346200	-2.036259
22	6	0	-1.689454	-0.292158	1.297706
23	1	0	0.334632	-3.935833	0.690126
24	1	0	-2.404667	-1.508007	-2.667350
25	1	0	-1.068978	-2.585672	-2.268236
26	1	0	-2.710088	-3.207245	-2.332948
27	1	0	-0.904936	0.352608	1.690822
28	1	0	-2.578482	0.320927	1.145187
29	1	0	-1.924949	-1.028407	2.068523
30	6	0	-3.783998	-1.747617	-0.208194
31	6	0	-4.339245	-0.531913	-0.982349
32	6	0	-5.648240	0.036688	-0.439224
33	6	0	-6.154581	1.285220	-1.185761
34	6	0	-5.619826	2.453777	-0.351845
35	6	0	-5.445841	1.833349	1.031596
36	8	0	-5.470652	0.478727	0.937997
37	1	0	-6.414163	-0.736529	-0.397627
38	1	0	-7.247381	1.302813	-1.188454
39	1	0	-5.819082	1.303902	-2.223045
40	1	0	-4.607414	2.715795	-0.681879
41	6	0	-6.471204	3.719088	-0.331041
42	1	0	-3.834165	-1.503519	0.854487
43	1	0	-4.522033	-0.811248	-2.025589
44	1	0	-3.604298	0.275441	-1.005867
45	8	0	-5.293341	2.399638	2.076921
46	1	0	-6.055025	4.447504	0.365586
47	1	0	-6.514272	4.172462	-1.323541
48	1	0	-7.492793	3.496255	-0.014171
49	1	0	1.437804	-0.882423	-1.639804
50	6	0	3.118280	0.095542	-0.771972
51	6	0	3.574492	0.168203	-2.238064

52	6	0	3.514328	1.386667	-0.005451
53	1	0	4.611499	0.496724	-2.336068
54	1	0	3.492492	-0.805153	-2.724182
55	1	0	2.952919	0.869052	-2.801203
56	6	0	3.224180	2.733602	-0.674345
57	6	0	3.867700	3.887877	0.064609
58	8	0	4.799588	3.810410	0.826199
59	8	0	3.275623	5.058921	-0.259896
60	6	0	3.843090	6.233551	0.346959
61	6	0	5.043182	-1.725115	-0.556742
62	1	0	4.017070	-0.756617	1.001218
63	6	0	5.103442	-2.887843	-1.206416
64	6	0	6.300133	-0.930196	-0.301079
65	1	0	6.048546	-3.268680	-1.576155
66	1	0	4.236102	-3.507840	-1.391891
67	1	0	6.252242	0.066686	-0.746846
68	1	0	6.452929	-0.781597	0.772744
69	1	0	7.177648	-1.436663	-0.704523
70	1	0	2.160086	2.945639	-0.765213
71	1	0	3.627932	2.767711	-1.691677
72	1	0	4.589575	1.351155	0.179913
73	1	0	3.041026	1.365386	0.977166
74	8	0	2.563519	-4.279105	1.395952
75	1	0	3.683550	-2.773115	2.098759
76	6	0	1.282156	-1.086613	2.872917
77	1	0	1.012698	-0.311281	3.590421
78	1	0	0.485182	-1.832586	2.836361
79	1	0	2.204408	-1.567461	3.215876
80	6	0	-4.702023	-2.965783	-0.423615
81	1	0	-5.715892	-2.753661	-0.078820
82	1	0	-4.768984	-3.247608	-1.477470
83	1	0	-4.359171	-3.836841	0.134467
84	1	0	4.886676	6.347475	0.054204
85	1	0	3.249001	7.066404	-0.020554
86	1	0	3.782119	6.167995	1.432877

3

Center Number	Atomic Number	Atomic Type	Coordinates (Angstroms) X	Y	Z
1	6	0	-1.056792	0.084566	-1.173521
2	6	0	0.323788	0.765682	-1.117524
3	1	0	-1.084451	-0.505939	-2.090066
4	1	0	-1.827368	0.853396	-1.275790
5	1	0	0.369162	1.498477	-0.311194
6	1	0	0.480630	1.311739	-2.052635
7	6	0	3.611899	-1.100081	-0.231620
8	6	0	2.470139	-1.994932	0.318045
9	6	0	1.241943	-1.034940	0.409727
10	6	0	1.400013	-0.300151	-0.936159
11	6	0	2.906274	-2.745378	1.558066
12	1	0	2.180236	-2.757117	-0.405077
13	8	0	1.411115	-0.038857	1.438264
14	6	0	-1.960305	-3.090128	0.780047
15	6	0	-2.462254	-1.976520	-0.190799
16	6	0	-1.370536	-0.814704	0.054112
17	6	0	-0.137052	-1.648415	0.466268
18	6	0	-0.480491	-2.866923	0.872520
19	1	0	-2.419594	-2.996150	1.772814
20	1	0	-2.211959	-4.091793	0.421746
21	6	0	-2.358665	-2.548219	-1.619092
22	6	0	-1.764852	0.099393	1.238398
23	1	0	0.206633	-3.616101	1.244732
24	1	0	-2.681326	-1.843746	-2.386103
25	1	0	-1.332874	-2.842342	-1.848585
26	1	0	-2.978934	-3.441251	-1.714458
27	1	0	-0.955043	0.791044	1.466406
28	1	0	-2.653266	0.691803	1.016446
29	1	0	-1.968072	-0.476090	2.143330
30	6	0	-3.941177	-1.586275	0.135141
31	6	0	-4.523777	-0.526968	-0.825351
32	6	0	-5.815557	0.136176	-0.352667
33	6	0	-6.333607	1.247529	-1.284565
34	6	0	-5.772707	2.533545	-0.669288
35	6	0	-5.569950	2.150486	0.794101
36	8	0	-5.601476	0.799212	0.927309
37	1	0	-6.584058	-0.612356	-0.164280
38	1	0	-7.426031	1.274608	-1.264966
39	1	0	-6.023051	1.090390	-2.317852
40	1	0	-4.766662	2.726847	-1.060336
41	6	0	-6.615618	3.792853	-0.841744
42	1	0	-3.940377	-1.149634	1.135557
43	1	0	-4.739780	-0.986524	-1.795926
44	1	0	-3.790830	0.259547	-1.016321
45	8	0	-5.391666	2.881119	1.727352
46	1	0	-6.179443	4.623063	-0.285334
47	1	0	-6.678060	4.075209	-1.894760
48	1	0	-7.631280	3.635835	-0.470816
49	1	0	1.195312	-1.072199	-1.687980
50	6	0	2.912734	0.045258	-1.104717
51	6	0	3.268490	-0.019212	-2.597606

52	6	0	3.201587	1.466370	-0.554439
53	1	0	4.319240	0.199246	-2.790984
54	1	0	3.062362	-1.006316	-3.013475
55	1	0	2.676382	0.712844	-3.153726
56	6	0	4.663004	1.949986	-0.594987
57	6	0	4.740764	3.387423	-0.135632
58	8	0	4.366745	4.337973	-0.778041
59	8	0	5.263846	3.493663	1.105674
60	6	0	5.339764	4.829741	1.635415
61	6	0	4.742632	-1.889297	-0.881457
62	1	0	4.050049	-0.595480	0.636509
63	6	0	4.581240	-3.087918	-1.442446
64	6	0	6.116003	-1.266824	-0.835314
65	1	0	5.422327	-3.610249	-1.883370
66	1	0	3.629823	-3.600809	-1.489588
67	1	0	6.866886	-1.939162	-1.251296
68	1	0	6.159040	-0.329703	-1.394973
69	1	0	6.402900	-1.031010	0.194786
70	1	0	5.051803	1.928340	-1.614639
71	1	0	5.302532	1.337564	0.038435
72	1	0	2.845565	1.520516	0.474414
73	1	0	2.609252	2.179727	-1.132639
74	8	0	2.497714	-3.832372	1.886848
75	1	0	3.685932	-2.242801	2.168764
76	6	0	1.277114	-0.446039	2.792298
77	1	0	1.103858	0.460637	3.372322
78	1	0	0.437093	-1.128673	2.936296
79	1	0	2.189932	-0.923489	3.163700
80	6	0	-4.876374	-2.809272	0.193733
81	1	0	-5.867570	-2.522736	0.550183
82	1	0	-5.005148	-3.275619	-0.786385
83	1	0	-4.508006	-3.571408	0.880290
84	1	0	4.345561	5.271599	1.695423
85	1	0	5.774504	4.725752	2.626256
86	1	0	5.969902	5.453724	1.002228

4

Center Number	Atomic Number	Atomic Type	Coordinates (Angstroms) X	Y	Z
1	6	0	-0.924904	0.011897	-1.177023
2	6	0	0.444039	0.721893	-1.162557
3	1	0	-0.970015	-0.564302	-2.101633
4	1	0	-1.714113	0.765479	-1.241739
5	1	0	0.494107	1.469095	-0.369766
6	1	0	0.565805	1.254998	-2.109671
7	6	0	3.788213	-0.929570	-0.207318
8	6	0	2.718066	-1.922392	0.302496
9	6	0	1.433069	-1.031673	0.371393
10	6	0	1.537911	-0.327396	-0.995316
11	6	0	3.168918	-2.641329	1.555624
12	1	0	2.502590	-2.698415	-0.435057
13	8	0	1.556329	-0.003665	1.374629
14	6	0	-1.677980	-3.216027	0.764088
15	6	0	-2.228042	-2.122101	-0.203025
16	6	0	-1.186512	-0.914648	0.045137
17	6	0	0.082669	-1.700195	0.448248
18	6	0	-0.208436	-2.932326	0.853279
19	1	0	-2.139151	-3.144333	1.757852
20	1	0	-1.888185	-4.226282	0.403226
21	6	0	-2.100721	-2.684565	-1.633066
22	6	0	-1.617330	-0.028281	1.237775
23	1	0	0.511343	-3.652709	1.220516
24	1	0	-2.458935	-1.994680	-2.397476
25	1	0	-1.062764	-2.928974	-1.866022
26	1	0	-2.678139	-3.605728	-1.728750
27	1	0	-0.846563	0.709710	1.455750
28	1	0	-2.541198	0.511711	1.028379
29	1	0	-1.775325	-0.615862	2.144016
30	6	0	-3.723059	-1.798886	0.124386
31	6	0	-4.351939	-0.760005	-0.829675
32	6	0	-5.662175	-0.144530	-0.343737
33	6	0	-6.234741	0.941659	-1.273436
34	6	0	-5.715223	2.251454	-0.671871
35	6	0	-5.475839	1.883550	0.789836
36	8	0	-5.456824	0.532852	0.930263
37	1	0	-6.399045	-0.920816	-0.141672
38	1	0	-7.327007	0.927119	-1.238768
39	1	0	-5.932577	0.791175	-2.310194
40	1	0	-4.723841	2.482752	-1.079270
41	6	0	-6.609945	3.475759	-0.836168
42	1	0	-3.742404	-1.370338	1.128062
43	1	0	-4.559784	-1.226890	-1.798531
44	1	0	-3.649911	0.052680	-1.026438
45	8	0	-5.309538	2.625151	1.716690
46	1	0	-6.198534	4.325110	-0.289896
47	1	0	-6.699647	3.750554	-1.889201
48	1	0	-7.612761	3.280250	-0.448950
49	1	0	1.288196	-1.121669	-1.707805
50	6	0	3.049290	-0.008700	-1.270925
51	6	0	3.352416	-0.452446	-2.715859

52	6	0	3.428786	1.492106	-1.192521
53	1	0	4.389669	-0.262237	-2.997078
54	1	0	3.163179	-1.517992	-2.857443
55	1	0	2.715896	0.097400	-3.415148
56	6	0	3.417096	2.195463	0.164620
57	6	0	3.562670	3.694875	0.036107
58	8	0	3.428863	4.339872	-0.974780
59	8	0	3.848049	4.254596	1.233721
60	6	0	3.958253	5.688531	1.241337
61	6	0	5.102728	-1.552869	-0.636276
62	1	0	4.021649	-0.289086	0.648951
63	6	0	5.245532	-2.842790	-0.940836
64	6	0	6.292197	-0.625209	-0.660336
65	1	0	6.211628	-3.242168	-1.227321
66	1	0	4.426484	-3.549893	-0.916853
67	1	0	7.201268	-1.151084	-0.953775
68	1	0	6.144282	0.206358	-1.354389
69	1	0	6.458099	-0.181699	0.327094
70	1	0	4.214199	1.844232	0.823402
71	1	0	2.490526	2.005324	0.710086
72	1	0	2.764959	2.040496	-1.864114
73	1	0	4.424548	1.616191	-1.627443
74	8	0	2.805598	-3.744335	1.885701
75	1	0	3.909829	-2.096942	2.177120
76	6	0	1.456845	-0.386522	2.739577
77	1	0	1.210518	0.515942	3.299547
78	1	0	0.674943	-1.131360	2.900786
79	1	0	2.405898	-0.779160	3.118939
80	6	0	-4.603655	-3.062189	0.172764
81	1	0	-5.609469	-2.820214	0.521371
82	1	0	-4.703769	-3.530566	-0.809675
83	1	0	-4.208146	-3.809251	0.860893
84	1	0	4.758285	6.012940	0.576294
85	1	0	3.021609	6.144538	0.921275
86	1	0	4.182785	5.957601	2.270380

5

Center Number	Atomic Number	Atomic Type	Coordinates (Angstroms) X	Y	Z
1	6	0	-1.022014	0.150989	-1.103712
2	6	0	0.358601	0.821602	-0.971634
3	1	0	-1.037918	-0.361984	-2.065837
4	1	0	-1.789830	0.927732	-1.152160
5	1	0	0.390690	1.487883	-0.108826
6	1	0	0.531437	1.439386	-1.858311
7	6	0	3.631019	-1.117777	-0.185604
8	6	0	2.479016	-2.045207	0.283645
9	6	0	1.250094	-1.092109	0.426262
10	6	0	1.431064	-0.257240	-0.856585
11	6	0	2.896052	-2.882497	1.473773
12	1	0	2.198906	-2.753290	-0.495889
13	8	0	1.398228	-0.176647	1.528788
14	6	0	-1.952788	-3.174317	0.568080
15	6	0	-2.438854	-1.985929	-0.318844
16	6	0	-1.355864	-0.845235	0.040889
17	6	0	-0.129041	-1.708085	0.409204
18	6	0	-0.476242	-2.955661	0.709557
19	1	0	-2.433325	-3.163963	1.555017
20	1	0	-2.192696	-4.143863	0.123436
21	6	0	-2.305857	-2.440548	-1.786430
22	6	0	-1.778471	-0.029163	1.285470
23	1	0	0.206886	-3.729848	1.035086
24	1	0	-2.611723	-1.675590	-2.500566
25	1	0	-1.276108	-2.719088	-2.017895
26	1	0	-2.925369	-3.321015	-1.965412
27	1	0	-0.972877	0.638062	1.588044
28	1	0	-2.660051	0.582912	1.089807
29	1	0	-2.004749	-0.674583	2.136268
30	6	0	-3.925769	-1.625896	0.005886
31	6	0	-4.494779	-0.501795	-0.887587
32	6	0	-5.777762	0.147680	-0.374112
33	6	0	-6.297114	1.302569	-1.250880
34	6	0	-5.720569	2.556151	-0.585597
35	6	0	-5.506312	2.107931	0.857418
36	8	0	-5.547369	0.752317	0.931827
37	1	0	-6.549363	-0.603707	-0.211245
38	1	0	-7.389066	1.336281	-1.218881
39	1	0	-5.998084	1.188841	-2.293188
40	1	0	-4.717009	2.759356	-0.978023
41	6	0	-6.555397	3.828002	-0.693738
42	1	0	-3.947715	-1.264253	1.035379
43	1	0	-4.716661	-0.900107	-1.883582
44	1	0	-3.752364	0.285742	-1.032270
45	8	0	-5.312672	2.795967	1.819506
46	1	0	-6.108314	4.629022	-0.104135
47	1	0	-6.624771	4.158301	-1.732303
48	1	0	-7.569010	3.661659	-0.321320
49	1	0	1.237971	-0.968465	-1.669017
50	6	0	2.946758	0.096294	-0.973507
51	6	0	3.324274	0.152763	-2.461632

52	6	0	3.232817	1.463630	-0.299724
53	1	0	4.380964	0.369096	-2.622991
54	1	0	3.109528	-0.791994	-2.962824
55	1	0	2.750258	0.937705	-2.962121
56	6	0	4.697778	1.940055	-0.286344
57	6	0	4.808874	3.262065	0.434989
58	8	0	5.048288	3.395339	1.609956
59	8	0	4.576022	4.303438	-0.395264
60	6	0	4.610177	5.605585	0.215513
61	6	0	4.761573	-1.862477	-0.887280
62	1	0	4.066670	-0.684805	0.721633
63	6	0	4.593523	-3.006352	-1.551074
64	6	0	6.141466	-1.263079	-0.773492
65	1	0	5.434139	-3.499390	-2.025337
66	1	0	3.636900	-3.501480	-1.652734
67	1	0	6.890838	-1.912415	-1.227188
68	1	0	6.204873	-0.287628	-1.261668
69	1	0	6.414835	-1.108385	0.275363
70	1	0	5.081803	2.057933	-1.300383
71	1	0	5.329250	1.237394	0.254547
72	1	0	2.871824	1.426495	0.727573
73	1	0	2.641613	2.223923	-0.817384
74	8	0	2.482902	-3.990247	1.716222
75	1	0	3.664877	-2.424713	2.131291
76	6	0	1.247568	-0.686154	2.846745
77	1	0	0.421946	-1.397091	2.921001
78	1	0	2.164992	-1.167973	3.200403
79	1	0	1.041831	0.170556	3.488534
80	6	0	-4.855893	-2.853004	-0.047002
81	1	0	-5.861466	-2.592803	0.289239
82	1	0	-4.945308	-3.256437	-1.058806
83	1	0	-4.508510	-3.655609	0.603431
84	1	0	4.399907	6.309136	-0.586056
85	1	0	3.856282	5.677971	0.999040
86	1	0	5.592304	5.796999	0.647056

6

Center Number	Atomic Number	Atomic Type	Coordinates (Angstroms) X	Y	Z
1	6	0	-0.857634	0.150912	-1.032277
2	6	0	0.513337	0.811110	-0.788748
3	1	0	-0.838355	-0.255553	-2.044194
4	1	0	-1.629043	0.925683	-1.023373
5	1	0	0.513717	1.390805	0.135891
6	1	0	0.699456	1.509700	-1.608679
7	6	0	3.760618	-1.143050	0.008584
8	6	0	2.620644	-2.157894	0.239632
9	6	0	1.369749	-1.237008	0.425722
10	6	0	1.596674	-0.263734	-0.748158
11	6	0	2.971126	-3.144489	1.333092
12	1	0	2.415235	-2.752327	-0.652600
13	8	0	1.465163	-0.437989	1.622286
14	6	0	-1.815085	-3.340312	0.228779
15	6	0	-2.286299	-2.061520	-0.531116
16	6	0	-1.221864	-0.964635	-0.014908
17	6	0	0.000009	-1.856815	0.292183
18	6	0	-0.344887	-3.132632	0.436323
19	1	0	-2.321164	-3.443099	1.197412
20	1	0	-2.035667	-4.254318	-0.328997
21	6	0	-2.108415	-2.346202	-2.036259
22	6	0	-1.689454	-0.292158	1.297704
23	1	0	0.334633	-3.935833	0.690127
24	1	0	-2.404669	-1.508009	-2.667351
25	1	0	-1.068978	-2.585672	-2.268236
26	1	0	-2.710088	-3.207248	-2.332947
27	1	0	-0.904935	0.352609	1.690820
28	1	0	-2.578481	0.320928	1.145185
29	1	0	-1.924949	-1.028406	2.068522
30	6	0	-3.783998	-1.747617	-0.208194
31	6	0	-4.339245	-0.531913	-0.982350
32	6	0	-5.648239	0.036688	-0.439225
33	6	0	-6.154581	1.285220	-1.185762
34	6	0	-5.619825	2.453777	-0.351844
35	6	0	-5.445841	1.833348	1.031596
36	8	0	-5.470653	0.478727	0.937997
37	1	0	-6.414163	-0.736529	-0.397628
38	1	0	-7.247380	1.302813	-1.188455
39	1	0	-5.819081	1.303903	-2.223045
40	1	0	-4.607414	2.715795	-0.681877
41	6	0	-6.471203	3.719088	-0.331040
42	1	0	-3.834164	-1.503519	0.854487
43	1	0	-4.522032	-0.811248	-2.025589
44	1	0	-3.604298	0.275441	-1.005867
45	8	0	-5.293342	2.399636	2.076922
46	1	0	-6.055025	4.447504	0.365587
47	1	0	-6.514270	4.172462	-1.323540
48	1	0	-7.492793	3.496255	-0.014171
49	1	0	1.437805	-0.882423	-1.639805
50	6	0	3.118281	0.095542	-0.771972
51	6	0	3.574493	0.168203	-2.238064

52	6	0	3.514327	1.386667	-0.005451
53	1	0	4.611500	0.496724	-2.336068
54	1	0	3.492494	-0.805153	-2.724182
55	1	0	2.952921	0.869052	-2.801203
56	6	0	3.224180	2.733602	-0.674346
57	6	0	3.867698	3.887877	0.064609
58	8	0	4.799585	3.810411	0.826201
59	8	0	3.275622	5.058921	-0.259898
60	6	0	3.843088	6.233552	0.346958
61	6	0	5.043182	-1.725115	-0.556741
62	1	0	4.017071	-0.756617	1.001218
63	6	0	5.103443	-2.887844	-1.206415
64	6	0	6.300134	-0.930196	-0.301079
65	1	0	6.048547	-3.268681	-1.576155
66	1	0	4.236102	-3.507840	-1.391890
67	1	0	6.452929	-0.781597	0.772744
68	1	0	7.177649	-1.436663	-0.704523
69	1	0	6.252243	0.066686	-0.746846
70	1	0	2.160086	2.945638	-0.765215
71	1	0	3.627933	2.767711	-1.691677
72	1	0	4.589575	1.351156	0.179914
73	1	0	3.041025	1.365386	0.977166
74	8	0	2.563520	-4.279104	1.395954
75	1	0	3.683549	-2.773114	2.098760
76	6	0	1.282156	-1.086612	2.872916
77	1	0	0.485182	-1.832584	2.836360
78	1	0	2.204408	-1.567459	3.215876
79	1	0	1.012697	-0.311279	3.590420
80	6	0	-4.702023	-2.965783	-0.423615
81	1	0	-5.715892	-2.753661	-0.078820
82	1	0	-4.768985	-3.247608	-1.477469
83	1	0	-4.359171	-3.836841	0.134467
84	1	0	4.886675	6.347475	0.054206
85	1	0	3.249001	7.066404	-0.020558
86	1	0	3.782115	6.167996	1.432876

7

Center Number	Atomic Number	Atomic Type	Coordinates (Angstroms) X	Y	Z
1	6	0	-0.924903	0.011901	-1.177021
2	6	0	0.444041	0.721896	-1.162555
3	1	0	-0.970014	-0.564299	-2.101631
4	1	0	-1.714112	0.765482	-1.241738
5	1	0	0.494109	1.469098	-0.369765
6	1	0	0.565806	1.255001	-2.109670
7	6	0	3.788214	-0.929572	-0.207321
8	6	0	2.718065	-1.922392	0.302495
9	6	0	1.433071	-1.031671	0.371392
10	6	0	1.537912	-0.327394	-0.995316
11	6	0	3.168916	-2.641330	1.555622
12	1	0	2.502587	-2.698415	-0.435058
13	8	0	1.556332	-0.003665	1.374631
14	6	0	-1.677979	-3.216022	0.764095
15	6	0	-2.228041	-2.122098	-0.203020
16	6	0	-1.186511	-0.914645	0.045139
17	6	0	0.082670	-1.700192	0.448250
18	6	0	-0.208434	-2.932322	0.853283
19	1	0	-2.139148	-3.144326	1.757860
20	1	0	-1.888185	-4.226278	0.403236
21	6	0	-2.100720	-2.684566	-1.633061
22	6	0	-1.617327	-0.028276	1.237778
23	1	0	0.511345	-3.652704	1.220520
24	1	0	-2.458936	-1.994684	-2.397472
25	1	0	-1.062762	-2.928972	-1.866018
26	1	0	-2.678135	-3.605730	-1.728742
27	1	0	-0.846558	0.709712	1.455753
28	1	0	-2.541194	0.511718	1.028381
29	1	0	-1.775325	-0.615858	2.144018
30	6	0	-3.723058	-1.798883	0.124389
31	6	0	-4.351938	-0.760003	-0.829673
32	6	0	-5.662177	-0.144532	-0.343740
33	6	0	-6.234744	0.941653	-1.273442
34	6	0	-5.715231	2.251452	-0.671877
35	6	0	-5.475852	1.883550	0.789832
36	8	0	-5.456834	0.532852	0.930260
37	1	0	-6.399045	-0.920822	-0.141678
38	1	0	-7.327010	0.927110	-1.238776
39	1	0	-5.932577	0.791169	-2.310199
40	1	0	-4.723847	2.482750	-1.079272
41	6	0	-6.609954	3.475754	-0.836181
42	1	0	-3.742404	-1.370334	1.128066
43	1	0	-4.559778	-1.226888	-1.798531
44	1	0	-3.649911	0.052684	-1.026434
45	8	0	-5.309560	2.625152	1.716686
46	1	0	-6.198546	4.325109	-0.289911
47	1	0	-6.699654	3.750544	-1.889215
48	1	0	-7.612771	3.280245	-0.448965
49	1	0	1.288196	-1.121666	-1.707806
50	6	0	3.049291	-0.008699	-1.270924
51	6	0	3.352416	-0.452442	-2.715860

52	6	0	3.428788	1.492107	-1.192518
53	1	0	4.389670	-0.262233	-2.997078
54	1	0	3.163178	-1.517988	-2.857447
55	1	0	2.715897	0.097406	-3.415147
56	6	0	3.417100	2.195462	0.164624
57	6	0	3.562670	3.694875	0.036113
58	8	0	3.428858	4.339873	-0.974772
59	8	0	3.848067	4.254592	1.233725
60	6	0	3.958274	5.688528	1.241343
61	6	0	5.102727	-1.552873	-0.636284
62	1	0	4.021654	-0.289090	0.648949
63	6	0	5.245527	-2.842793	-0.940849
64	6	0	6.292199	-0.625217	-0.660340
65	1	0	6.211622	-3.242173	-1.227335
66	1	0	4.426477	-3.549894	-0.916869
67	1	0	7.201267	-1.151092	-0.953785
68	1	0	6.144284	0.206356	-1.354385
69	1	0	6.458105	-0.181716	0.327093
70	1	0	4.214204	1.844230	0.823404
71	1	0	2.490532	2.005321	0.710091
72	1	0	2.764961	2.040499	-1.864109
73	1	0	4.424550	1.616193	-1.627441
74	8	0	2.805591	-3.744333	1.885701
75	1	0	3.909832	-2.096947	2.177116
76	6	0	1.456853	-0.386526	2.739579
77	1	0	1.210522	0.515934	3.299552
78	1	0	0.674955	-1.131369	2.900785
79	1	0	2.405908	-0.779162	3.118937
80	6	0	-4.603655	-3.062185	0.172767
81	1	0	-5.609469	-2.820208	0.521374
82	1	0	-4.703769	-3.530562	-0.809672
83	1	0	-4.208147	-3.809247	0.860897
84	1	0	4.758302	6.012935	0.576294
85	1	0	3.021629	6.144537	0.921290
86	1	0	4.182816	5.957593	2.270384

8

Center Number	Atomic Number	Atomic Type	Coordinates (Angstroms) X	Y	Z
1	6	0	-1.074232	0.190310	-1.137443
2	6	0	0.320541	0.836044	-1.027078
3	1	0	-1.099282	-0.356090	-2.080807
4	1	0	-1.825779	0.980389	-1.216596
5	1	0	0.369258	1.536051	-0.192213
6	1	0	0.509343	1.414322	-1.936688
7	6	0	3.548218	-1.124894	-0.137926
8	6	0	2.378067	-2.025112	0.350650
9	6	0	1.167646	-1.039897	0.446904
10	6	0	1.367889	-0.261002	-0.869252
11	6	0	2.769746	-2.811699	1.580115
12	1	0	2.102571	-2.761441	-0.405516
13	8	0	1.335857	-0.084242	1.511891
14	6	0	-2.073203	-3.055029	0.652381
15	6	0	-2.532350	-1.892890	-0.283302
16	6	0	-1.432092	-0.758197	0.040602
17	6	0	-0.222650	-1.628297	0.448926
18	6	0	-0.593743	-2.856796	0.795418
19	1	0	-2.560901	-2.997880	1.634332
20	1	0	-2.326828	-4.036713	0.243635
21	6	0	-2.397824	-2.405230	-1.731401
22	6	0	-1.845458	0.110009	1.252616
23	1	0	0.072665	-3.631564	1.153171
24	1	0	-2.690656	-1.664678	-2.476113
25	1	0	-1.370607	-2.705244	-1.946709
26	1	0	-3.027972	-3.283887	-1.879613
27	1	0	-1.031681	0.777982	1.531089
28	1	0	-2.717031	0.727230	1.031130
29	1	0	-2.084129	-0.498829	2.126487
30	6	0	-4.014420	-1.496439	0.019680
31	6	0	-4.552117	-0.378011	-0.898578
32	6	0	-5.861967	0.262623	-0.443512
33	6	0	-6.313106	1.456769	-1.305472
34	6	0	-5.778034	2.677290	-0.549845
35	6	0	-5.669028	2.168555	0.884659
36	8	0	-5.716914	0.810879	0.898444
37	1	0	-6.647782	-0.487561	-0.365335
38	1	0	-7.404453	1.497859	-1.348047
39	1	0	-5.942565	1.384020	-2.328336
40	1	0	-4.748009	2.887526	-0.861547
41	6	0	-6.595613	3.959688	-0.663916
42	1	0	-4.037102	-1.113592	1.041441
43	1	0	-4.728288	-0.776089	-1.903813
44	1	0	-3.808615	0.413751	-1.010663
45	8	0	-5.545220	2.814284	1.886624
46	1	0	-6.184831	4.732778	-0.013777
47	1	0	-6.591480	4.331237	-1.690767
48	1	0	-7.633365	3.788063	-0.368301
49	1	0	1.153990	-1.001384	-1.649980
50	6	0	2.889490	0.047857	-1.002417
51	6	0	3.285077	0.040566	-2.489055

52	6	0	3.233664	1.435265	-0.400925
53	1	0	4.367039	0.049014	-2.626775
54	1	0	2.893968	-0.832276	-3.013311
55	1	0	2.879069	0.924858	-2.988262
56	6	0	4.704830	1.847821	-0.469598
57	6	0	4.938708	3.245834	0.059315
58	8	0	4.094543	4.089282	0.231968
59	8	0	6.252533	3.456001	0.302830
60	6	0	6.597128	4.773635	0.765412
61	6	0	4.690106	-1.924726	-0.748317
62	1	0	3.956188	-0.643377	0.754105
63	6	0	5.950285	-1.703022	-0.373169
64	6	0	4.377468	-3.028767	-1.730775
65	1	0	6.774473	-2.269618	-0.791402
66	1	0	6.205701	-0.955658	0.369088
67	1	0	5.289353	-3.392708	-2.205334
68	1	0	3.907166	-3.878813	-1.226935
69	1	0	3.691501	-2.708176	-2.516231
70	1	0	5.079296	1.844498	-1.498236
71	1	0	5.356291	1.168165	0.081085
72	1	0	2.904724	1.465372	0.637339
73	1	0	2.649581	2.194949	-0.924775
74	8	0	2.418337	-3.943693	1.812324
75	1	0	3.462673	-2.295974	2.276252
76	6	0	1.122879	-0.517325	2.848353
77	1	0	0.948547	0.381611	3.439943
78	1	0	0.256583	-1.176048	2.935588
79	1	0	2.000733	-1.031244	3.253064
80	6	0	-4.968302	-2.705959	-0.013482
81	1	0	-5.961864	-2.425813	0.341121
82	1	0	-5.083572	-3.111103	-1.022128
83	1	0	-4.623569	-3.513160	0.632668
84	1	0	7.675649	4.761509	0.901036
85	1	0	6.311228	5.523541	0.028098
86	1	0	6.094076	4.990263	1.707486

9

Center Number	Atomic Number	Atomic Type	Coordinates (Angstroms) X	Y	Z
1	6	0	-1.066679	0.127531	-1.144170
2	6	0	0.309751	0.818421	-1.099480
3	1	0	-1.095549	-0.464479	-2.059460
4	1	0	-1.844279	0.889351	-1.241860
5	1	0	0.357481	1.554511	-0.295900
6	1	0	0.457161	1.360751	-2.038060
7	6	0	3.617261	-1.025969	-0.237910
8	6	0	2.484201	-1.928939	0.332740
9	6	0	1.253131	-0.966089	0.430690
10	6	0	1.392181	-0.241489	-0.923480
11	6	0	2.944661	-2.642909	1.577880
12	1	0	2.198941	-2.703949	-0.379540
13	8	0	1.430861	0.036321	1.450500
14	6	0	-1.926949	-3.049349	0.831950
15	6	0	-2.441269	-1.950659	-0.149890
16	6	0	-1.365109	-0.772619	0.086810
17	6	0	-0.120599	-1.588299	0.502530
18	6	0	-0.450229	-2.806339	0.920600
19	1	0	-2.387569	-2.951569	1.823570
20	1	0	-2.165919	-4.056919	0.482220
21	6	0	-2.325859	-2.532149	-1.573420
22	6	0	-1.770279	0.140861	1.268080
23	1	0	0.244941	-3.546169	1.296800
24	1	0	-2.657669	-1.838359	-2.345960
25	1	0	-1.294889	-2.811349	-1.798560
26	1	0	-2.932659	-3.434959	-1.662000
27	1	0	-0.971249	0.847241	1.490190
28	1	0	-2.667119	0.718061	1.039780
29	1	0	-1.963799	-0.433009	2.176120
30	6	0	-3.927009	-1.579229	0.169500
31	6	0	-4.518719	-0.533509	-0.800050
32	6	0	-5.839789	0.091801	-0.362470
33	6	0	-6.366599	1.186141	-1.306790
34	6	0	-5.861779	2.490661	-0.679960
35	6	0	-5.689839	2.115031	0.785150
36	8	0	-5.675629	0.772231	0.925980
37	1	0	-6.591309	-0.674689	-0.183770
38	1	0	-7.458939	1.179041	-1.316090
39	1	0	-6.021529	1.040151	-2.330040
40	1	0	-4.850449	2.711281	-1.041280
41	6	0	-6.737289	3.722001	-0.889860
42	1	0	-3.934319	-1.139749	1.168900
43	1	0	-4.703639	-0.996939	-1.774540
44	1	0	-3.804349	0.272881	-0.976960
45	8	0	-5.564609	2.852391	1.730360
46	1	0	-6.334369	4.579611	-0.349940
47	1	0	-6.786989	3.976681	-1.950150
48	1	0	-7.754759	3.542571	-0.534990
49	1	0	1.176891	-1.020619	-1.664400
50	6	0	2.899631	0.097741	-1.120060
51	6	0	3.244021	0.054591	-2.618160

52	6	0	3.232641	1.511181	-0.573200
53	1	0	4.320001	0.084001	-2.793770
54	1	0	2.854051	-0.841889	-3.100740
55	1	0	2.801851	0.914981	-3.127980
56	6	0	4.695521	1.967051	-0.753360
57	6	0	4.877931	3.352880	-0.186920
58	8	0	4.581751	4.379341	-0.757770
59	8	0	5.387681	3.327060	1.059110
60	6	0	5.552281	4.605320	1.706360
61	6	0	4.750031	-1.828409	-0.862240
62	1	0	4.051511	-0.502619	0.616780
63	6	0	6.017361	-1.577450	-0.529510
64	6	0	4.424831	-2.962279	-1.805520
65	1	0	6.837681	-2.142890	-0.957330
66	1	0	6.280661	-0.805860	0.184980
67	1	0	5.327611	-3.324920	-2.298080
68	1	0	3.983671	-3.805629	-1.265370
69	1	0	3.708681	-2.673839	-2.575910
70	1	0	4.956211	2.016440	-1.810550
71	1	0	5.383101	1.283640	-0.257820
72	1	0	2.972661	1.550831	0.484550
73	1	0	2.594871	2.236611	-1.084860
74	8	0	2.679691	-3.793169	1.850540
75	1	0	3.606681	-2.061259	2.247940
76	6	0	1.259081	-0.343499	2.810670
77	1	0	2.156841	-0.821779	3.214170
78	1	0	1.081201	0.575971	3.368480
79	1	0	0.408011	-1.013039	2.946850
80	6	0	-4.843629	-2.816259	0.230930
81	1	0	-5.838979	-2.543779	0.586610
82	1	0	-4.965069	-3.285139	-0.748720
83	1	0	-4.462739	-3.570999	0.918920
84	1	0	5.971201	4.383750	2.684020
85	1	0	6.231211	5.234820	1.132720
86	1	0	4.589761	5.105291	1.806570

10

Center Number	Atomic Number	Atomic Type	Coordinates (Angstroms) X	Y	Z
1	6	0	-1.056794	0.084571	-1.173516
2	6	0	0.323787	0.765684	-1.117519
3	1	0	-1.084454	-0.505933	-2.090063
4	1	0	-1.827368	0.853401	-1.275782
5	1	0	0.369163	1.498478	-0.311187
6	1	0	0.480629	1.311743	-2.052628
7	6	0	3.611897	-1.100081	-0.231620
8	6	0	2.470137	-1.994933	0.318043
9	6	0	1.241942	-1.034942	0.409728
10	6	0	1.400011	-0.300150	-0.936157
11	6	0	2.906273	-2.745383	1.558062
12	1	0	2.180233	-2.757117	-0.405081
13	8	0	1.411113	-0.038860	1.438266
14	6	0	-1.960308	-3.090129	0.780044
15	6	0	-2.462257	-1.976517	-0.190798
16	6	0	-1.370537	-0.814703	0.054115
17	6	0	-0.137054	-1.648416	0.466267
18	6	0	-0.480494	-2.866925	0.872517
19	1	0	-2.419596	-2.996153	1.772811
20	1	0	-2.211963	-4.091793	0.421740
21	6	0	-2.358669	-2.548212	-1.619093
22	6	0	-1.764850	0.099391	1.238404
23	1	0	0.206631	-3.616104	1.244726
24	1	0	-2.681329	-1.843735	-2.386102
25	1	0	-1.332878	-2.842336	-1.848587
26	1	0	-2.978940	-3.441243	-1.714461
27	1	0	-0.955041	0.791040	1.466413
28	1	0	-2.653264	0.691804	1.016454
29	1	0	-1.968071	-0.476094	2.143335
30	6	0	-3.941179	-1.586272	0.135143
31	6	0	-4.523782	-0.526969	-0.825350
32	6	0	-5.815560	0.136178	-0.352664
33	6	0	-6.333612	1.247528	-1.284565
34	6	0	-5.772710	2.533546	-0.669294
35	6	0	-5.569949	2.150492	0.794096
36	8	0	-5.601475	0.799218	0.927308
37	1	0	-6.584061	-0.612354	-0.164272
38	1	0	-7.426036	1.274607	-1.264963
39	1	0	-6.023060	1.090385	-2.317852
40	1	0	-4.766665	2.726845	-1.060345
41	6	0	-6.615620	3.792854	-0.841752
42	1	0	-3.940377	-1.149628	1.135558
43	1	0	-4.739788	-0.986528	-1.795923
44	1	0	-3.790836	0.259545	-1.016326
45	8	0	-5.391660	2.881128	1.727344
46	1	0	-6.179443	4.623065	-0.285346
47	1	0	-6.678065	4.075206	-1.894770
48	1	0	-7.631281	3.635838	-0.470820
49	1	0	1.195308	-1.072196	-1.687980
50	6	0	2.912733	0.045258	-1.104716
51	6	0	3.268487	-0.019213	-2.597606

52	6	0	3.201588	1.466370	-0.554439
53	1	0	4.319237	0.199245	-2.790985
54	1	0	3.062358	-1.006317	-3.013473
55	1	0	2.676378	0.712842	-3.153725
56	6	0	4.663006	1.949981	-0.594985
57	6	0	4.740770	3.387418	-0.135629
58	8	0	4.366742	4.337968	-0.778034
59	8	0	5.263865	3.493658	1.105671
60	6	0	5.339788	4.829736	1.635411
61	6	0	4.742632	-1.889295	-0.881457
62	1	0	4.050046	-0.595481	0.636510
63	6	0	4.581243	-3.087918	-1.442445
64	6	0	6.116001	-1.266818	-0.835316
65	1	0	5.422330	-3.610247	-1.883368
66	1	0	3.629827	-3.600812	-1.489585
67	1	0	6.866886	-1.939155	-1.251298
68	1	0	6.159035	-0.329698	-1.394976
69	1	0	6.402898	-1.031002	0.194783
70	1	0	5.051807	1.928334	-1.614637
71	1	0	5.302532	1.337558	0.038437
72	1	0	2.845564	1.520517	0.474413
73	1	0	2.609256	2.179728	-1.132640
74	8	0	2.497712	-3.832376	1.886843
75	1	0	3.685933	-2.242808	2.168760
76	6	0	1.277108	-0.446043	2.792300
77	1	0	1.103853	0.460633	3.372324
78	1	0	0.437085	-1.128675	2.936294
79	1	0	2.189925	-0.923495	3.163703
80	6	0	-4.876375	-2.809270	0.193743
81	1	0	-5.867570	-2.522734	0.550195
82	1	0	-5.005151	-3.275620	-0.786373
83	1	0	-4.508003	-3.571404	0.880301
84	1	0	4.345585	5.271594	1.695430
85	1	0	5.774538	4.725747	2.626248
86	1	0	5.969919	5.453720	1.002218

11

Center Number	Atomic Number	Atomic Type	Coordinates (Angstroms) X	Y	Z
1	6	0	-1.033548	0.162013	-1.105224
2	6	0	0.345559	0.839687	-0.987782
3	1	0	-1.050884	-0.363228	-2.060694
4	1	0	-1.804713	0.934921	-1.160912
5	1	0	0.379866	1.518508	-0.134774
6	1	0	0.513384	1.444725	-1.884075
7	6	0	3.627108	-1.067124	-0.168394
8	6	0	2.480315	-2.001532	0.311438
9	6	0	1.247790	-1.047948	0.436354
10	6	0	1.421836	-0.233647	-0.861640
11	6	0	2.899168	-2.805466	1.521293
12	1	0	2.215059	-2.727991	-0.457845
13	8	0	1.395960	-0.111381	1.520737
14	6	0	-1.940744	-3.146776	0.614663
15	6	0	-2.435986	-1.974188	-0.288388
16	6	0	-1.360129	-0.821035	0.053334
17	6	0	-0.127483	-1.670592	0.432332
18	6	0	-0.465542	-2.916141	0.750680
19	1	0	-2.420124	-3.125373	1.601984
20	1	0	-2.174733	-4.124081	0.184227
21	6	0	-2.302554	-2.448163	-1.749667
22	6	0	-1.786899	0.010357	1.286284
23	1	0	0.222731	-3.682433	1.084558
24	1	0	-2.616208	-1.695628	-2.473567
25	1	0	-1.271181	-2.721889	-1.979694
26	1	0	-2.915543	-3.335803	-1.915105
27	1	0	-0.987621	0.691275	1.574863
28	1	0	-2.675179	0.610062	1.083659
29	1	0	-2.004131	-0.623041	2.148345
30	6	0	-3.924717	-1.620143	0.034546
31	6	0	-4.503186	-0.510081	-0.870372
32	6	0	-5.788612	0.136789	-0.359604
33	6	0	-6.319773	1.277761	-1.247414
34	6	0	-5.750777	2.543374	-0.598452
35	6	0	-5.525857	2.112892	0.848346
36	8	0	-5.556895	0.757943	0.938268
37	1	0	-6.554331	-0.617932	-0.184816
38	1	0	-7.411820	1.303346	-1.211899
39	1	0	-6.023538	1.154573	-2.289446
40	1	0	-4.750964	2.751147	-0.997967
41	6	0	-6.597411	3.806585	-0.716226
42	1	0	-3.947207	-1.247108	1.059960
43	1	0	-4.725597	-0.921298	-1.860989
44	1	0	-3.766340	0.280526	-1.026430
45	8	0	-5.332105	2.812970	1.801665
46	1	0	-6.154381	4.618065	-0.137991
47	1	0	-6.675170	4.124581	-1.758018
48	1	0	-7.607524	3.635407	-0.336561
49	1	0	1.223823	-0.959485	-1.660018
50	6	0	2.935279	0.114961	-0.992964
51	6	0	3.324003	0.169872	-2.479723

52	6	0	3.253843	1.485683	-0.340391
53	1	0	4.404898	0.207881	-2.620328
54	1	0	2.949077	-0.692609	-3.031904
55	1	0	2.897497	1.062263	-2.946570
56	6	0	4.719014	1.953749	-0.454369
57	6	0	4.928345	3.217705	0.343173
58	8	0	5.292176	3.263476	1.492890
59	8	0	4.626802	4.319673	-0.380450
60	6	0	4.749339	5.573124	0.314528
61	6	0	4.774472	-1.832016	-0.812737
62	1	0	4.039889	-0.600808	0.729250
63	6	0	6.033532	-1.608052	-0.435024
64	6	0	4.469650	-2.909200	-1.827181
65	1	0	6.862043	-2.152257	-0.874028
66	1	0	6.283072	-0.883509	0.331542
67	1	0	5.383816	-3.249376	-2.314770
68	1	0	4.010029	-3.778890	-1.347537
69	1	0	3.777958	-2.573637	-2.601384
70	1	0	4.986836	2.140940	-1.494491
71	1	0	5.399087	1.206961	-0.048184
72	1	0	2.974504	1.446701	0.712094
73	1	0	2.622833	2.246158	-0.808470
74	8	0	2.565456	-3.946285	1.735656
75	1	0	3.593722	-2.293513	2.218105
76	6	0	1.218174	-0.583198	2.849819
77	1	0	1.002078	0.291956	3.462761
78	1	0	0.388826	-1.289009	2.928623
79	1	0	2.126670	-1.056593	3.235147
80	6	0	-4.846916	-2.853866	-0.002477
81	1	0	-5.853221	-2.596406	0.333634
82	1	0	-4.936549	-3.268745	-1.009607
83	1	0	-4.493062	-3.647264	0.655745
84	1	0	4.473209	6.335959	-0.409089
85	1	0	4.080360	5.598662	1.174448
86	1	0	5.773845	5.719830	0.655543

12

Center Number	Atomic Number	Atomic Type	Coordinates (Angstroms) X	Y	Z
1	6	0	-0.872707	0.183452	-1.107184
2	6	0	0.510392	0.827533	-0.890369
3	1	0	-0.856887	-0.273819	-2.097132
4	1	0	-1.628176	0.972895	-1.143082
5	1	0	0.521555	1.456885	0.000835
6	1	0	0.712225	1.476915	-1.746095
7	6	0	3.711727	-1.152339	0.024688
8	6	0	2.550188	-2.133563	0.289961
9	6	0	1.318244	-1.178475	0.425484
10	6	0	1.571102	-0.265877	-0.792164
11	6	0	2.872996	-3.077099	1.428828
12	1	0	2.339419	-2.762539	-0.576916
13	8	0	1.429066	-0.325733	1.583071
14	6	0	-1.907369	-3.224825	0.305424
15	6	0	-2.349400	-1.970842	-0.512017
16	6	0	-1.265129	-0.873353	-0.039913
17	6	0	-0.063609	-1.774912	0.315062
18	6	0	-0.435045	-3.035635	0.515311
19	1	0	-2.424142	-3.278310	1.272434
20	1	0	-2.139340	-4.157548	-0.215560
21	6	0	-2.172173	-2.326592	-2.002192
22	6	0	-1.723559	-0.131425	1.237975
23	1	0	0.226546	-3.840252	0.809044
24	1	0	-2.445593	-1.510802	-2.671815
25	1	0	-1.138173	-2.601541	-2.218781
26	1	0	-2.794230	-3.184940	-2.261569
27	1	0	-0.925110	0.509745	1.607950
28	1	0	-2.593327	0.499591	1.050429
29	1	0	-1.984524	-0.826151	2.038253
30	6	0	-3.842605	-1.616473	-0.209645
31	6	0	-4.357853	-0.398645	-1.005960
32	6	0	-5.714572	0.144779	-0.562462
33	6	0	-6.131655	1.449318	-1.266879
34	6	0	-5.681079	2.547656	-0.298517
35	6	0	-5.671707	1.829243	1.048022
36	8	0	-5.686301	0.484723	0.853977
37	1	0	-6.483288	-0.620509	-0.660103
38	1	0	-7.217969	1.482471	-1.382814
39	1	0	-5.688315	1.537121	-2.259233
40	1	0	-4.634538	2.810964	-0.493528
41	6	0	-6.513282	3.825517	-0.284213
42	1	0	-3.898046	-1.354440	0.848574
43	1	0	-4.455887	-0.661232	-2.064889
44	1	0	-3.633809	0.417254	-0.958744
45	8	0	-5.643380	2.317247	2.142156
46	1	0	-6.170923	4.494437	0.505974
47	1	0	-6.435549	4.348468	-1.239701
48	1	0	-7.567406	3.602468	-0.102936
49	1	0	1.405969	-0.922116	-1.655298
50	6	0	3.100505	0.059196	-0.820500
51	6	0	3.573220	0.053184	-2.282404

52	6	0	3.509356	1.381394	-0.112880
53	1	0	4.623407	0.335483	-2.382342
54	1	0	3.456228	-0.935685	-2.728148
55	1	0	2.986149	0.755405	-2.879573
56	6	0	3.293075	2.697357	-0.889306
57	6	0	3.755146	3.877401	-0.067925
58	8	0	3.038964	4.608759	0.570304
59	8	0	5.100755	4.013414	-0.109633
60	6	0	5.642443	5.088944	0.677046
61	6	0	4.988879	-1.782370	-0.499840
62	1	0	3.963812	-0.725985	1.002142
63	6	0	5.033424	-2.970698	-1.102238
64	6	0	6.258502	-1.001964	-0.261983
65	1	0	5.974720	-3.384161	-1.445666
66	1	0	4.155857	-3.580248	-1.273947
67	1	0	6.405540	-0.818636	0.807507
68	1	0	7.129887	-1.538455	-0.639003
69	1	0	6.232325	-0.021655	-0.745106
70	1	0	2.242529	2.863694	-1.113051
71	1	0	3.855193	2.684343	-1.824317
72	1	0	4.575619	1.318385	0.118206
73	1	0	2.985542	1.438030	0.842494
74	8	0	2.450703	-4.203037	1.533318
75	1	0	3.579307	-2.682294	2.188666
76	6	0	1.215380	-0.905690	2.862489
77	1	0	0.395962	-1.627630	2.854942
78	1	0	2.119014	-1.397576	3.238079
79	1	0	0.964308	-0.087559	3.537825
80	6	0	-4.786092	-2.817380	-0.412908
81	1	0	-5.784607	-2.593503	-0.033640
82	1	0	-4.888349	-3.085821	-1.467676
83	1	0	-4.439850	-3.700381	0.123879
84	1	0	5.393363	4.954323	1.729391
85	1	0	6.718531	5.045630	0.528736
86	1	0	5.246140	6.045391	0.337009

13

Center Number	Atomic Number	Atomic Type	Coordinates (Angstroms) X	Y	Z
1	6	0	-1.022014	0.150989	-1.103712
2	6	0	0.358601	0.821602	-0.971635
3	1	0	-1.037918	-0.361984	-2.065837
4	1	0	-1.789830	0.927732	-1.152160
5	1	0	0.390690	1.487883	-0.108827
6	1	0	0.531437	1.439386	-1.858311
7	6	0	3.631019	-1.117777	-0.185604
8	6	0	2.479016	-2.045207	0.283645
9	6	0	1.250094	-1.092109	0.426262
10	6	0	1.431064	-0.257240	-0.856585
11	6	0	2.896052	-2.882497	1.473773
12	1	0	2.198906	-2.753290	-0.495889
13	8	0	1.398228	-0.176647	1.528788
14	6	0	-1.952788	-3.174317	0.568080
15	6	0	-2.438854	-1.985929	-0.318843
16	6	0	-1.355864	-0.845235	0.040889
17	6	0	-0.129041	-1.708084	0.409204
18	6	0	-0.476242	-2.955661	0.709557
19	1	0	-2.433325	-3.163963	1.555017
20	1	0	-2.192696	-4.143863	0.123436
21	6	0	-2.305857	-2.440548	-1.786430
22	6	0	-1.778471	-0.029163	1.285470
23	1	0	0.206886	-3.729848	1.035086
24	1	0	-2.611723	-1.675590	-2.500566
25	1	0	-1.276108	-2.719088	-2.017895
26	1	0	-2.925369	-3.321015	-1.965412
27	1	0	-0.972877	0.638062	1.588044
28	1	0	-2.660051	0.582912	1.089807
29	1	0	-2.004749	-0.674583	2.136268
30	6	0	-3.925769	-1.625896	0.005886
31	6	0	-4.494779	-0.501795	-0.887587
32	6	0	-5.777762	0.147680	-0.374112
33	6	0	-6.297114	1.302569	-1.250880
34	6	0	-5.720569	2.556151	-0.585597
35	6	0	-5.506312	2.107931	0.857418
36	8	0	-5.547369	0.752317	0.931827
37	1	0	-6.549363	-0.603707	-0.211245
38	1	0	-7.389066	1.336281	-1.218881
39	1	0	-5.998085	1.188841	-2.293188
40	1	0	-4.717009	2.759356	-0.978023
41	6	0	-6.555397	3.828002	-0.693739
42	1	0	-3.947715	-1.264253	1.035379
43	1	0	-4.716661	-0.900107	-1.883582
44	1	0	-3.752364	0.285742	-1.032270
45	8	0	-5.312672	2.795967	1.819506
46	1	0	-6.108314	4.629022	-0.104136
47	1	0	-6.624771	4.158301	-1.732304
48	1	0	-7.569010	3.661659	-0.321320
49	1	0	1.237971	-0.968465	-1.669017
50	6	0	2.946758	0.096294	-0.973507
51	6	0	3.324274	0.152763	-2.461632

52	6	0	3.232817	1.463630	-0.299724
53	1	0	4.380964	0.369096	-2.622991
54	1	0	3.109528	-0.791994	-2.962824
55	1	0	2.750258	0.937705	-2.962121
56	6	0	4.697778	1.940055	-0.286344
57	6	0	4.808874	3.262065	0.434989
58	8	0	5.048288	3.395339	1.609956
59	8	0	4.576022	4.303438	-0.395264
60	6	0	4.610176	5.605585	0.215514
61	6	0	4.761573	-1.862477	-0.887280
62	1	0	4.066670	-0.684805	0.721633
63	6	0	4.593523	-3.006352	-1.551074
64	6	0	6.141466	-1.263079	-0.773492
65	1	0	5.434139	-3.499390	-2.025337
66	1	0	3.636900	-3.501480	-1.652734
67	1	0	6.890838	-1.912415	-1.227188
68	1	0	6.204873	-0.287628	-1.261669
69	1	0	6.414835	-1.108385	0.275363
70	1	0	5.081803	2.057933	-1.300384
71	1	0	5.329250	1.237394	0.254546
72	1	0	2.871824	1.426495	0.727573
73	1	0	2.641613	2.223923	-0.817384
74	8	0	2.482902	-3.990247	1.716222
75	1	0	3.664877	-2.424713	2.131291
76	6	0	1.247568	-0.686154	2.846745
77	1	0	0.421946	-1.397091	2.921001
78	1	0	2.164992	-1.167972	3.200403
79	1	0	1.041831	0.170556	3.488534
80	6	0	-4.855893	-2.853004	-0.047001
81	1	0	-5.861466	-2.592803	0.289239
82	1	0	-4.945308	-3.256437	-1.058806
83	1	0	-4.508510	-3.655609	0.603431
84	1	0	4.399907	6.309136	-0.586056
85	1	0	3.856281	5.677970	0.999040
86	1	0	5.592304	5.796999	0.647056

14

Center Number	Atomic Number	Atomic Type	Coordinates (Angstroms) X	Y	Z
1	6	0	-0.924904	0.011898	-1.177022
2	6	0	0.444039	0.721893	-1.162556
3	1	0	-0.970016	-0.564301	-2.101633
4	1	0	-1.714114	0.765479	-1.241738
5	1	0	0.494107	1.469096	-0.369765
6	1	0	0.565805	1.254998	-2.109671
7	6	0	3.788213	-0.929570	-0.207318
8	6	0	2.718066	-1.922392	0.302496
9	6	0	1.433069	-1.031673	0.371393
10	6	0	1.537911	-0.327396	-0.995316
11	6	0	3.168918	-2.641329	1.555623
12	1	0	2.502589	-2.698416	-0.435057
13	8	0	1.556329	-0.003666	1.374629
14	6	0	-1.677980	-3.216027	0.764088
15	6	0	-2.228042	-2.122101	-0.203024
16	6	0	-1.186512	-0.914648	0.045137
17	6	0	0.082669	-1.700195	0.448249
18	6	0	-0.208436	-2.932327	0.853279
19	1	0	-2.139151	-3.144334	1.757852
20	1	0	-1.888185	-4.226282	0.403226
21	6	0	-2.100721	-2.684565	-1.633066
22	6	0	-1.617330	-0.028281	1.237776
23	1	0	0.511343	-3.652709	1.220516
24	1	0	-2.458935	-1.994680	-2.397476
25	1	0	-1.062764	-2.928974	-1.866022
26	1	0	-2.678139	-3.605727	-1.728750
27	1	0	-0.846563	0.709710	1.455751
28	1	0	-2.541198	0.511711	1.028380
29	1	0	-1.775326	-0.615862	2.144017
30	6	0	-3.723059	-1.798886	0.124386
31	6	0	-4.351939	-0.760005	-0.829675
32	6	0	-5.662175	-0.144530	-0.343736
33	6	0	-6.234741	0.941659	-1.273436
34	6	0	-5.715223	2.251455	-0.671871
35	6	0	-5.475838	1.883550	0.789836
36	8	0	-5.456824	0.532853	0.930263
37	1	0	-6.399045	-0.920816	-0.141671
38	1	0	-7.327007	0.927119	-1.238768
39	1	0	-5.932577	0.791175	-2.310194
40	1	0	-4.723840	2.482752	-1.079270
41	6	0	-6.609945	3.475759	-0.836169
42	1	0	-3.742405	-1.370338	1.128063
43	1	0	-4.559784	-1.226890	-1.798531
44	1	0	-3.649911	0.052680	-1.026437
45	8	0	-5.309537	2.625152	1.716690
46	1	0	-6.198533	4.325111	-0.289896
47	1	0	-6.699647	3.750554	-1.889201
48	1	0	-7.612761	3.280250	-0.448950
49	1	0	1.288195	-1.121669	-1.707805
50	6	0	3.049289	-0.008700	-1.270925
51	6	0	3.352415	-0.452446	-2.715860

52	6	0	3.428785	1.492106	-1.192521
53	1	0	4.389668	-0.262236	-2.997079
54	1	0	3.163178	-1.517992	-2.857444
55	1	0	2.715895	0.097400	-3.415148
56	6	0	3.417097	2.195462	0.164621
57	6	0	3.562668	3.694875	0.036107
58	8	0	3.428853	4.339873	-0.974778
59	8	0	3.848057	4.254595	1.233720
60	6	0	3.958258	5.688531	1.241336
61	6	0	5.102728	-1.552869	-0.636277
62	1	0	4.021650	-0.289086	0.648951
63	6	0	5.245531	-2.842790	-0.940838
64	6	0	6.292197	-0.625210	-0.660338
65	1	0	6.211627	-3.242169	-1.227323
66	1	0	4.426483	-3.549893	-0.916854
67	1	0	7.201267	-1.151085	-0.953777
68	1	0	6.144282	0.206357	-1.354390
69	1	0	6.458099	-0.181700	0.327092
70	1	0	4.214200	1.844232	0.823401
71	1	0	2.490528	2.005323	0.710087
72	1	0	2.764959	2.040497	-1.864113
73	1	0	4.424548	1.616191	-1.627443
74	8	0	2.805598	-3.744335	1.885701
75	1	0	3.909830	-2.096942	2.177119
76	6	0	1.456846	-0.386522	2.739578
77	1	0	1.210518	0.515941	3.299547
78	1	0	0.674944	-1.131361	2.900786
79	1	0	2.405898	-0.779160	3.118939
80	6	0	-4.603656	-3.062189	0.172764
81	1	0	-5.609470	-2.820214	0.521371
82	1	0	-4.703769	-3.530566	-0.809675
83	1	0	-4.208147	-3.809251	0.860893
84	1	0	4.758285	6.012941	0.576287
85	1	0	3.021611	6.144536	0.921283
86	1	0	4.182799	5.957599	2.270377

15

Center Number	Atomic Number	Atomic Type	Coordinates (Angstroms) X	Y	Z
1	6	0	-0.951481	0.175144	-1.184440
2	6	0	0.445583	0.816551	-1.085257
3	1	0	-0.973405	-0.402677	-2.109245
4	1	0	-1.697001	0.967074	-1.293917
5	1	0	0.491304	1.540850	-0.271422
6	1	0	0.642228	1.367105	-2.010242
7	6	0	3.657974	-1.145273	-0.135714
8	6	0	2.479158	-2.014354	0.376179
9	6	0	1.275095	-1.022325	0.446744
10	6	0	1.487342	-0.279715	-0.887614
11	6	0	2.864207	-2.784861	1.620317
12	1	0	2.188325	-2.762488	-0.360870
13	8	0	1.445000	-0.042454	1.489647
14	6	0	-1.987042	-2.997976	0.708497
15	6	0	-2.432889	-1.862328	-0.264190
16	6	0	-1.320729	-0.729838	0.023752
17	6	0	-0.120671	-1.599470	0.460441
18	6	0	-0.505182	-2.812785	0.844595
19	1	0	-2.472021	-2.901565	1.688804
20	1	0	-2.254086	-3.989383	0.332941
21	6	0	-2.303755	-2.421067	-1.695594
22	6	0	-1.726442	0.182644	1.205709
23	1	0	0.152490	-3.582262	1.228286
24	1	0	-2.592977	-1.702328	-2.462852
25	1	0	-1.278313	-2.732870	-1.902036
26	1	0	-2.938322	-3.300836	-1.816580
27	1	0	-0.905384	0.850489	1.463028
28	1	0	-2.592913	0.799499	0.964180
29	1	0	-1.968705	-0.396554	2.098917
30	6	0	-3.909820	-1.438364	0.024069
31	6	0	-4.437626	-0.355275	-0.941993
32	6	0	-5.683928	0.378709	-0.474224
33	6	0	-6.225500	1.426368	-1.458231
34	6	0	-6.819054	2.506633	-0.545703
35	6	0	-6.026894	2.328351	0.747666
36	8	0	-5.380020	1.132916	0.734692
37	1	0	-6.471272	-0.329531	-0.202847
38	1	0	-6.951887	0.995489	-2.148866
39	1	0	-5.401488	1.834197	-2.049222
40	1	0	-6.626405	3.514014	-0.917120
41	6	0	-8.322603	2.358079	-0.270461
42	1	0	-3.924888	-1.011223	1.028642
43	1	0	-4.675647	-0.806462	-1.911876
44	1	0	-3.673150	0.399262	-1.133488
45	8	0	-5.958338	3.083186	1.675892
46	1	0	-8.648676	3.084356	0.475167
47	1	0	-8.892199	2.524463	-1.187029
48	1	0	-8.565897	1.359648	0.101782
49	1	0	1.280780	-1.039782	-1.651187
50	6	0	3.011964	0.027892	-1.014161
51	6	0	3.405496	-0.035677	-2.497974

52	6	0	3.325062	1.435492	-0.443933
53	1	0	4.469551	0.141579	-2.659358
54	1	0	3.173162	-1.009289	-2.931605
55	1	0	2.857710	0.724525	-3.062008
56	6	0	4.788452	1.875738	-0.440135
57	6	0	4.959209	3.305208	0.029162
58	8	0	4.088994	4.135551	0.107058
59	8	0	6.251202	3.557883	0.339951
60	6	0	6.539535	4.904354	0.756411
61	6	0	4.783055	-1.960035	-0.763601
62	1	0	4.087011	-0.661366	0.748873
63	6	0	4.602765	-3.146176	-1.345051
64	6	0	6.172552	-1.379246	-0.672146
65	1	0	5.440460	-3.687508	-1.769243
66	1	0	3.638381	-3.629829	-1.426029
67	1	0	6.915674	-2.072478	-1.067421
68	1	0	6.261663	-0.442182	-1.226575
69	1	0	6.434656	-1.155475	0.367206
70	1	0	5.231880	1.832658	-1.440568
71	1	0	5.413708	1.244319	0.192688
72	1	0	2.941579	1.495845	0.573928
73	1	0	2.761515	2.171181	-1.021336
74	8	0	2.429016	-3.868765	1.924450
75	1	0	3.632196	-2.300729	2.260277
76	6	0	1.253276	-0.455633	2.834712
77	1	0	1.098620	0.452625	3.417451
78	1	0	0.382806	-1.105359	2.946629
79	1	0	2.134077	-0.972986	3.229644
80	6	0	-4.876071	-2.637957	0.042983
81	1	0	-5.876994	-2.325360	0.347335
82	1	0	-4.967964	-3.106334	-0.940188
83	1	0	-4.560079	-3.404724	0.750348
84	1	0	6.281692	5.610845	-0.032275
85	1	0	5.977029	5.153298	1.655906
86	1	0	7.608114	4.924856	0.954972

16

Center Number	Atomic Number	Atomic Type	Coordinates (Angstroms) X	Y	Z
1	6	0	-1.117822	0.120145	-1.142479
2	6	0	0.275109	0.772340	-1.063070
3	1	0	-1.148310	-0.455742	-2.068086
4	1	0	-1.873343	0.904232	-1.239858
5	1	0	0.328258	1.498187	-0.251047
6	1	0	0.454907	1.321778	-1.991864
7	6	0	3.520990	-1.198287	-0.224893
8	6	0	2.367040	-2.015846	0.406815
9	6	0	1.143122	-1.049628	0.467056
10	6	0	1.326598	-0.315925	-0.877215
11	6	0	2.779063	-2.729943	1.673470
12	1	0	2.054592	-2.821956	-0.266220
13	8	0	1.292093	-0.053108	1.496670
14	6	0	-2.104828	-3.063048	0.761180
15	6	0	-2.566265	-1.934732	-0.212859
16	6	0	-1.463532	-0.791370	0.067549
17	6	0	-0.247516	-1.645361	0.495122
18	6	0	-0.624393	-2.859206	0.884301
19	1	0	-2.585606	-2.967927	1.743460
20	1	0	-2.362789	-4.058116	0.389315
21	6	0	-2.437419	-2.497675	-1.642611
22	6	0	-1.866335	0.111565	1.257719
23	1	0	0.032873	-3.630290	1.266947
24	1	0	-2.736173	-1.784328	-2.410840
25	1	0	-1.410303	-2.800918	-1.853664
26	1	0	-3.065843	-3.382667	-1.757367
27	1	0	-1.051505	0.789234	1.507361
28	1	0	-2.742465	0.718409	1.026411
29	1	0	-2.094020	-0.471888	2.151679
30	6	0	-4.046729	-1.526746	0.082744
31	6	0	-4.586275	-0.438467	-0.869589
32	6	0	-5.904482	0.204151	-0.441969
33	6	0	-6.350811	1.378819	-1.332730
34	6	0	-5.829872	2.616481	-0.595281
35	6	0	-5.735854	2.136855	0.850281
36	8	0	-5.777389	0.779047	0.890222
37	1	0	-6.687651	-0.548263	-0.358657
38	1	0	-7.441746	1.413970	-1.388502
39	1	0	-5.968285	1.287603	-2.349706
40	1	0	-4.797098	2.825093	-0.898930
41	6	0	-6.651471	3.892668	-0.744805
42	1	0	-4.064448	-1.109921	1.091267
43	1	0	-4.751790	-0.864477	-1.865075
44	1	0	-3.847904	0.355582	-0.996985
45	8	0	-5.627359	2.802295	1.840850
46	1	0	-6.251849	4.680391	-0.105395
47	1	0	-6.636332	4.243621	-1.778757
48	1	0	-7.691999	3.722345	-0.458372
49	1	0	1.111290	-1.084970	-1.630628
50	6	0	2.844286	-0.002946	-1.041531
51	6	0	3.186064	0.004931	-2.540202

52	6	0	3.195966	1.377589	-0.428047
53	1	0	4.252529	0.139737	-2.725314
54	1	0	2.895626	-0.926539	-3.026465
55	1	0	2.659704	0.824777	-3.037236
56	6	0	4.667936	1.786346	-0.482957
57	6	0	4.901588	3.185321	0.045231
58	8	0	4.053776	4.015843	0.257324
59	8	0	6.221005	3.411334	0.239247
60	6	0	6.565389	4.726775	0.707412
61	6	0	4.529092	-2.066257	-0.970473
62	1	0	4.063183	-0.751962	0.609922
63	6	0	4.189534	-3.099896	-1.739433
64	6	0	5.978004	-1.700170	-0.783398
65	1	0	4.943201	-3.686861	-2.251322
66	1	0	3.162319	-3.406335	-1.899528
67	1	0	6.637183	-2.388894	-1.313165
68	1	0	6.186198	-0.689144	-1.145807
69	1	0	6.238399	-1.718048	0.279193
70	1	0	5.058301	1.778707	-1.505819
71	1	0	5.308361	1.109653	0.086174
72	1	0	2.865834	1.401394	0.609198
73	1	0	2.616785	2.144788	-0.946742
74	8	0	3.880024	-2.678050	2.165546
75	1	0	2.001492	-3.368243	2.139264
76	6	0	1.145004	-0.453386	2.852775
77	1	0	0.292878	-1.123247	2.993238
78	1	0	2.050068	-0.932761	3.234832
79	1	0	0.972796	0.459219	3.423685
80	6	0	-5.002345	-2.735311	0.094303
81	1	0	-5.990733	-2.443336	0.453434
82	1	0	-5.130978	-3.167875	-0.901285
83	1	0	-4.651733	-3.525409	0.758298
84	1	0	7.648306	4.727438	0.802592
85	1	0	6.242214	5.483062	-0.007732
86	1	0	6.095411	4.923282	1.670749

17

Center Number	Atomic Number	Atomic Type	Coordinates (Angstroms) X	Y	Z
1	6	0	-0.722658	0.217967	-1.064121
2	6	0	0.656621	0.856727	-0.809883
3	1	0	-0.697107	-0.198814	-2.071722
4	1	0	-1.480052	1.006198	-1.072503
5	1	0	0.655229	1.447287	0.107835
6	1	0	0.865755	1.541975	-1.635521
7	6	0	3.858233	-1.142589	0.056700
8	6	0	2.698108	-2.135411	0.283095
9	6	0	1.460497	-1.191039	0.439462
10	6	0	1.720393	-0.235922	-0.742691
11	6	0	3.015852	-3.113645	1.393733
12	1	0	2.495796	-2.737331	-0.604812
13	8	0	1.552889	-0.378997	1.627426
14	6	0	-1.754786	-3.244865	0.216091
15	6	0	-2.193735	-1.965904	-0.562494
16	6	0	-1.119679	-0.880875	-0.040745
17	6	0	0.082569	-1.789467	0.293161
18	6	0	-0.284679	-3.058319	0.444061
19	1	0	-2.277410	-3.329954	1.177692
20	1	0	-1.981952	-4.160584	-0.336353
21	6	0	-1.999910	-2.266764	-2.062468
22	6	0	-1.597161	-0.186235	1.256978
23	1	0	0.377927	-3.869883	0.715492
24	1	0	-2.277806	-1.430667	-2.704702
25	1	0	-0.960587	-2.520816	-2.278848
26	1	0	-2.607851	-3.123279	-2.359409
27	1	0	-0.808117	0.451041	1.653566
28	1	0	-2.475076	0.437568	1.084452
29	1	0	-1.852351	-0.910802	2.032813
30	6	0	-3.690136	-1.625025	-0.264307
31	6	0	-4.211340	-0.402633	-1.052355
32	6	0	-5.487773	0.219770	-0.510359
33	6	0	-6.024722	1.409006	-1.320720
34	6	0	-6.668842	2.308796	-0.258887
35	6	0	-5.909391	1.929354	1.010278
36	8	0	-5.237114	0.763947	0.817900
37	1	0	-6.265769	-0.538510	-0.388107
38	1	0	-6.721272	1.088705	-2.096866
39	1	0	-5.193673	1.925376	-1.808168
40	1	0	-6.489793	3.367821	-0.449427
41	6	0	-8.175819	2.088176	-0.061235
42	1	0	-3.750907	-1.374239	0.796452
43	1	0	-4.408899	-0.687983	-2.091736
44	1	0	-3.458185	0.386162	-1.086932
45	8	0	-5.882167	2.519378	2.053060
46	1	0	-8.540104	2.670824	0.785847
47	1	0	-8.722451	2.397967	-0.954233
48	1	0	-8.406518	1.036705	0.127879
49	1	0	1.562693	-0.862152	-1.629260
50	6	0	3.248314	0.096089	-0.749524
51	6	0	3.726979	0.139370	-2.209630

52	6	0	3.655768	1.391215	0.004336
53	1	0	4.772241	0.443988	-2.296284
54	1	0	3.631035	-0.838382	-2.684245
55	1	0	3.128974	0.845751	-2.791058
56	6	0	3.401909	2.731824	-0.690954
57	6	0	4.051977	3.887450	0.039860
58	8	0	4.951667	3.805492	0.838772
59	8	0	3.507655	5.065105	-0.339891
60	6	0	4.085953	6.240195	0.255540
61	6	0	5.139435	-1.752585	-0.481716
62	1	0	4.105854	-0.747400	1.048096
63	6	0	5.190431	-2.924541	-1.115365
64	6	0	6.405150	-0.974222	-0.218349
65	1	0	6.134548	-3.325175	-1.466237
66	1	0	4.315961	-3.532991	-1.305679
67	1	0	6.545319	-0.814027	0.855519
68	1	0	7.280107	-1.499451	-0.602951
69	1	0	6.378799	0.017317	-0.677608
70	1	0	2.343325	2.960827	-0.804076
71	1	0	3.822715	2.741922	-1.701724
72	1	0	4.726817	1.338746	0.208956
73	1	0	3.166015	1.394142	0.979048
74	8	0	2.591090	-4.241598	1.463177
75	1	0	3.720746	-2.743233	2.166819
76	6	0	1.336510	-1.007384	2.882934
77	1	0	0.523601	-1.735774	2.843483
78	1	0	2.242248	-1.504268	3.246471
79	1	0	1.073703	-0.217040	3.586408
80	6	0	-4.625853	-2.827866	-0.487510
81	1	0	-5.644468	-2.590731	-0.173442
82	1	0	-4.671059	-3.121978	-1.539074
83	1	0	-4.315145	-3.698003	0.090658
84	1	0	5.144199	6.312996	0.005452
85	1	0	3.534885	7.078895	-0.162268
86	1	0	3.976612	6.210751	1.339278

18

Center Number	Atomic Number	Atomic Type	Coordinates (Angstroms) X	Y	Z
1	6	0	-0.801397	0.042415	-1.213650
2	6	0	0.576376	0.734841	-1.203748
3	1	0	-0.842864	-0.561712	-2.120602
4	1	0	-1.579310	0.804119	-1.310592
5	1	0	0.627198	1.503872	-0.432325
6	1	0	0.716063	1.238740	-2.164322
7	6	0	3.887611	-0.930966	-0.163503
8	6	0	2.798988	-1.896112	0.360305
9	6	0	1.525571	-0.986317	0.391866
10	6	0	1.654456	-0.322615	-0.993087
11	6	0	3.227434	-2.588604	1.635891
12	1	0	2.581240	-2.687803	-0.359658
13	8	0	1.654616	0.067621	1.367231
14	6	0	-1.621256	-3.111691	0.821315
15	6	0	-2.144442	-2.042311	-0.186733
16	6	0	-1.088771	-0.841650	0.034069
17	6	0	0.164621	-1.632499	0.474931
18	6	0	-0.148355	-2.846875	0.915861
19	1	0	-2.090361	-2.999944	1.807645
20	1	0	-1.844414	-4.129886	0.491666
21	6	0	-2.008793	-2.651751	-1.596809
22	6	0	-1.521384	0.088747	1.192153
23	1	0	0.557060	-3.565468	1.313060
24	1	0	-2.349538	-1.981602	-2.386358
25	1	0	-0.971154	-2.914995	-1.809659
26	1	0	-2.595718	-3.569020	-1.670758
27	1	0	-0.741954	0.821703	1.397054
28	1	0	-2.434807	0.634265	0.952996
29	1	0	-1.697242	-0.468896	2.114180
30	6	0	-3.637584	-1.685835	0.110710
31	6	0	-4.241293	-0.685172	-0.899057
32	6	0	-5.505165	0.021045	-0.436478
33	6	0	-6.131024	0.967918	-1.471339
34	6	0	-6.741099	2.085840	-0.617057
35	6	0	-5.895536	2.039318	0.653559
36	8	0	-5.196027	0.875031	0.703254
37	1	0	-6.246413	-0.701420	-0.084593
38	1	0	-6.861017	0.455170	-2.099374
39	1	0	-5.349343	1.366611	-2.122994
40	1	0	-6.609088	3.070335	-1.067911
41	6	0	-8.225473	1.892452	-0.273713
42	1	0	-3.659944	-1.209406	1.092791
43	1	0	-4.485416	-1.203789	-1.832971
44	1	0	-3.517416	0.089138	-1.157522
45	8	0	-5.826830	2.864278	1.520157
46	1	0	-8.557793	2.655228	0.431621
47	1	0	-8.834276	1.968532	-1.176916
48	1	0	-8.409528	0.913364	0.175838
49	1	0	1.403024	-1.133938	-1.685438
50	6	0	3.172558	-0.029686	-1.259542
51	6	0	3.486066	-0.514145	-2.689141

52	6	0	3.570245	1.467919	-1.216404
53	1	0	4.528424	-0.342955	-2.963836
54	1	0	3.285641	-1.580632	-2.806247
55	1	0	2.863690	0.025093	-3.409105
56	6	0	3.551988	2.207709	0.121099
57	6	0	3.729228	3.699899	-0.045137
58	8	0	3.637020	4.318306	-1.077014
59	8	0	3.991830	4.287988	1.144222
60	6	0	4.131192	5.719035	1.114865
61	6	0	5.198671	-1.581532	-0.560837
62	1	0	4.119238	-0.271135	0.678388
63	6	0	5.328866	-2.880383	-0.831141
64	6	0	6.399604	-0.669052	-0.595683
65	1	0	6.293080	-3.298504	-1.096470
66	1	0	4.501059	-3.576818	-0.798758
67	1	0	7.304974	-1.212709	-0.867399
68	1	0	6.268239	0.147153	-1.311027
69	1	0	6.561460	-0.203586	0.382273
70	1	0	4.332677	1.859668	0.801015
71	1	0	2.614479	2.048864	0.657556
72	1	0	2.921008	2.006181	-1.910110
73	1	0	4.572232	1.567711	-1.642974
74	8	0	2.845128	-3.677395	1.990972
75	1	0	3.970807	-2.039162	2.250004
76	6	0	1.533396	-0.274727	2.740961
77	1	0	0.728144	-0.991093	2.915921
78	1	0	2.466946	-0.685201	3.139675
79	1	0	1.310119	0.650027	3.273533
80	6	0	-4.535742	-2.933337	0.209275
81	1	0	-5.551042	-2.657587	0.502107
82	1	0	-4.607272	-3.463908	-0.743521
83	1	0	-4.175863	-3.638169	0.958983
84	1	0	4.953916	6.008731	0.461591
85	1	0	3.212293	6.184346	0.758709
86	1	0	4.335520	6.012591	2.141436

19

Center Number	Atomic Number	Atomic Type	Coordinates (Angstroms) X	Y	Z
1	6	0	-0.922885	0.106433	-1.214528
2	6	0	0.461247	0.781073	-1.166528
3	1	0	-0.944232	-0.513971	-2.111327
4	1	0	-1.687295	0.876110	-1.349500
5	1	0	0.502431	1.540210	-0.384882
6	1	0	0.631370	1.293918	-2.117941
7	6	0	3.728703	-1.067729	-0.176313
8	6	0	2.575832	-1.936573	0.391015
9	6	0	1.351836	-0.968164	0.434028
10	6	0	1.529005	-0.283816	-0.936069
11	6	0	2.993578	-2.645239	1.661596
12	1	0	2.289948	-2.722351	-0.308198
13	8	0	1.514612	0.063480	1.427832
14	6	0	-1.862476	-2.997509	0.835413
15	6	0	-2.347920	-1.916243	-0.178660
16	6	0	-1.255285	-0.749423	0.039482
17	6	0	-0.030617	-1.572935	0.496011
18	6	0	-0.383186	-2.776154	0.938546
19	1	0	-2.333128	-2.868106	1.818791
20	1	0	-2.114438	-4.009740	0.508161
21	6	0	-2.228883	-2.535191	-1.586065
22	6	0	-1.663755	0.207407	1.184842
23	1	0	0.296419	-3.514422	1.344667
24	1	0	-2.543043	-1.855741	-2.378802
25	1	0	-1.200378	-2.835424	-1.794469
26	1	0	-2.847100	-3.431757	-1.659688
27	1	0	-0.856309	0.907104	1.397350
28	1	0	-2.550047	0.789987	0.930817
29	1	0	-1.875733	-0.336339	2.107557
30	6	0	-3.829070	-1.510048	0.114280
31	6	0	-4.398358	-0.493586	-0.899440
32	6	0	-5.648621	0.242699	-0.446747
33	6	0	-6.240850	1.208766	-1.483592
34	6	0	-6.835256	2.335297	-0.629642
35	6	0	-6.003031	2.264306	0.648715
36	8	0	-5.330212	1.084310	0.699454
37	1	0	-6.411267	-0.462472	-0.105810
38	1	0	-6.974956	0.715842	-2.122620
39	1	0	-5.442918	1.593093	-2.124143
40	1	0	-6.678326	3.319023	-1.074116
41	6	0	-8.326467	2.171138	-0.301159
42	1	0	-3.836764	-1.028997	1.094377
43	1	0	-4.647176	-1.004204	-1.836513
44	1	0	-3.652958	0.262903	-1.149678
45	8	0	-5.924318	3.083572	1.519802
46	1	0	-8.649615	2.937265	0.404793
47	1	0	-8.925036	2.263980	-1.209614
48	1	0	-8.534776	1.193947	0.141878
49	1	0	1.327248	-1.081291	-1.661629
50	6	0	3.045734	0.046389	-1.100599
51	6	0	3.419538	-0.078093	-2.585301

52	6	0	3.337544	1.486092	-0.602352
53	1	0	4.474715	0.124285	-2.771933
54	1	0	3.210637	-1.078821	-2.965657
55	1	0	2.841239	0.636664	-3.177390
56	6	0	4.803221	1.959126	-0.648615
57	6	0	4.885171	3.410025	-0.236552
58	8	0	4.638259	4.347952	-0.954476
59	8	0	5.243588	3.544859	1.059471
60	6	0	5.297841	4.895752	1.552260
61	6	0	4.864151	-1.886505	-0.779563
62	1	0	4.157163	-0.532389	0.678057
63	6	0	4.704369	-3.104369	-1.297921
64	6	0	6.240038	-1.269455	-0.737307
65	1	0	5.548930	-3.647115	-1.706319
66	1	0	3.750838	-3.613756	-1.340266
67	1	0	6.993449	-1.961166	-1.115165
68	1	0	6.296270	-0.355576	-1.333075
69	1	0	6.512947	-0.994180	0.286775
70	1	0	5.201226	1.898828	-1.662369
71	1	0	5.429866	1.364741	0.014808
72	1	0	2.972582	1.582728	0.420108
73	1	0	2.753881	2.178237	-1.214665
74	8	0	2.576913	-3.718923	2.022553
75	1	0	3.767389	-2.124616	2.264528
76	6	0	1.362218	-0.294418	2.793633
77	1	0	1.190570	0.633674	3.339225
78	1	0	0.514726	-0.964536	2.952097
79	1	0	2.266623	-0.766060	3.192122
80	6	0	-4.766989	-2.727861	0.215886
81	1	0	-5.771709	-2.420061	0.512795
82	1	0	-4.858865	-3.255685	-0.736718
83	1	0	-4.426977	-3.444283	0.963801
84	1	0	5.594064	4.813727	2.594915
85	1	0	6.028287	5.476187	0.989292
86	1	0	4.321239	5.371434	1.465951

20

Center Number	Atomic Number	Atomic Type	Coordinates (Angstroms) X	Y	Z
1	6	0	-0.891291	0.177000	-1.150267
2	6	0	0.492187	0.842150	-1.021083
3	1	0	-0.897842	-0.366190	-2.095892
4	1	0	-1.652295	0.957333	-1.233141
5	1	0	0.518342	1.535672	-0.179779
6	1	0	0.681146	1.430201	-1.924563
7	6	0	3.740223	-1.095289	-0.135872
8	6	0	2.575736	-1.999674	0.347538
9	6	0	1.352746	-1.033265	0.447405
10	6	0	1.555251	-0.239413	-0.858411
11	6	0	2.973071	-2.803380	1.567062
12	1	0	2.299057	-2.729080	-0.413266
13	8	0	1.497468	-0.085815	1.522832
14	6	0	-1.868597	-3.084574	0.619354
15	6	0	-2.332639	-1.924567	-0.315250
16	6	0	-1.246796	-0.779796	0.021234
17	6	0	-0.031127	-1.638885	0.433492
18	6	0	-0.391820	-2.872965	0.771711
19	1	0	-2.361254	-3.034070	1.599225
20	1	0	-2.112074	-4.067415	0.207019
21	6	0	-2.182509	-2.428944	-1.764787
22	6	0	-1.680204	0.079846	1.232620
23	1	0	0.280574	-3.641201	1.131782
24	1	0	-2.478137	-1.687982	-2.508050
25	1	0	-1.150463	-2.716319	-1.973815
26	1	0	-2.801038	-3.314224	-1.922382
27	1	0	-0.872780	0.750182	1.523949
28	1	0	-2.554537	0.691051	1.004912
29	1	0	-1.920742	-0.536669	2.100999
30	6	0	-3.820541	-1.542076	-0.024778
31	6	0	-4.367059	-0.445925	-0.966009
32	6	0	-5.613102	0.270953	-0.471960
33	6	0	-6.189468	1.310256	-1.445127
34	6	0	-6.772974	2.386706	-0.521862
35	6	0	-5.948187	2.220943	0.752542
36	8	0	-5.292196	1.030684	0.729260
37	1	0	-6.384440	-0.447547	-0.182093
38	1	0	-6.927097	0.869589	-2.117400
39	1	0	-5.384152	1.725263	-2.056611
40	1	0	-6.600308	3.395019	-0.900565
41	6	0	-8.267860	2.222729	-0.210558
42	1	0	-3.852292	-1.143674	0.991066
43	1	0	-4.612043	-0.883357	-1.940422
44	1	0	-3.611191	0.318265	-1.153662
45	8	0	-5.862509	2.981006	1.675114
46	1	0	-8.583925	2.947166	0.541140
47	1	0	-8.860782	2.381145	-1.113643
48	1	0	-8.491630	1.222584	0.169193
49	1	0	1.367547	-0.974304	-1.650858
50	6	0	3.074491	0.100400	-0.966755
51	6	0	3.471942	0.111446	-2.450566

52	6	0	3.361997	1.485114	-0.329494
53	1	0	4.532909	0.312797	-2.602549
54	1	0	3.254569	-0.844956	-2.927963
55	1	0	2.912839	0.887617	-2.980801
56	6	0	4.829724	1.953115	-0.312190
57	6	0	4.939719	3.293783	0.374050
58	8	0	5.165655	3.458042	1.547796
59	8	0	4.722668	4.313217	-0.487128
60	6	0	4.755763	5.631378	0.088255
61	6	0	4.872456	-1.868719	-0.802880
62	1	0	4.168897	-0.639318	0.763331
63	6	0	4.703176	-3.030778	-1.433984
64	6	0	6.255261	-1.275342	-0.692514
65	1	0	5.545105	-3.543331	-1.884666
66	1	0	3.744178	-3.522179	-1.531108
67	1	0	7.004647	-1.942303	-1.119865
68	1	0	6.330106	-0.314748	-1.207796
69	1	0	6.519302	-1.092282	0.354168
70	1	0	5.226233	2.041350	-1.324442
71	1	0	5.450987	1.262143	0.255010
72	1	0	2.988819	1.480961	0.694016
73	1	0	2.782133	2.233566	-0.876366
74	8	0	2.552043	-3.901999	1.836350
75	1	0	3.735368	-2.329649	2.220978
76	6	0	1.317136	-0.548865	2.853729
77	1	0	1.148493	0.335971	3.467503
78	1	0	0.458767	-1.217832	2.944241
79	1	0	2.208610	-1.062291	3.228510
80	6	0	-4.760257	-2.762467	-0.047106
81	1	0	-5.774260	-2.476513	0.240391
82	1	0	-4.820953	-3.214517	-1.040191
83	1	0	-4.442936	-3.534840	0.653571
84	1	0	5.732892	5.829434	0.528058
85	1	0	4.560427	6.313786	-0.735080
86	1	0	3.991512	5.729233	0.858899

21

Center Number	Atomic Number	Atomic Type	Coordinates (Angstroms) X	Y	Z
1	6	0	-1.109220	0.060790	-1.149300
2	6	0	0.271920	0.741590	-1.120460
3	1	0	-1.145960	-0.546790	-2.054200
4	1	0	-1.881690	0.826670	-1.257850
5	1	0	0.326070	1.493610	-0.332750
6	1	0	0.422410	1.263440	-2.070010
7	6	0	3.572040	-1.137611	-0.279050
8	6	0	2.445950	-1.953970	0.402170
9	6	0	1.204970	-1.009970	0.452460
10	6	0	1.347390	-0.320620	-0.919680
11	6	0	2.893980	-2.616820	1.684420
12	1	0	2.137820	-2.788440	-0.237520
13	8	0	1.354340	0.026040	1.443660
14	6	0	-1.995780	-3.074300	0.878800
15	6	0	-2.499420	-1.988090	-0.121230
16	6	0	-1.413570	-0.815160	0.097810
17	6	0	-0.172810	-1.630500	0.529310
18	6	0	-0.517670	-2.838460	0.964260
19	1	0	-2.457340	-2.956670	1.867780
20	1	0	-2.241720	-4.085650	0.544640
21	6	0	-2.388100	-2.594960	-1.534490
22	6	0	-1.812270	0.120130	1.263950
23	1	0	0.161760	-3.584710	1.357550
24	1	0	-2.714820	-1.912020	-2.318770
25	1	0	-1.359420	-2.885230	-1.755990
26	1	0	-3.000950	-3.495090	-1.609140
27	1	0	-1.009790	0.826150	1.472200
28	1	0	-2.708400	0.696280	1.031470
29	1	0	-2.005750	-0.437220	2.182320
30	6	0	-3.981490	-1.598470	0.191350
31	6	0	-4.566580	-0.565559	-0.795630
32	6	0	-5.870840	0.091401	-0.348840
33	6	0	-6.384050	1.188031	-1.300570
34	6	0	-5.842479	2.485311	-0.691420
35	6	0	-5.658759	2.120141	0.778990
36	8	0	-5.681030	0.769561	0.926500
37	1	0	-6.635810	-0.661709	-0.164210
38	1	0	-7.476790	1.206351	-1.296930
39	1	0	-6.057380	1.022201	-2.327530
40	1	0	-4.832179	2.682191	-1.069530
41	6	0	-6.692509	3.735841	-0.890800
42	1	0	-3.985720	-1.137660	1.180870
43	1	0	-4.767340	-1.045979	-1.759300
44	1	0	-3.840650	0.225500	-0.993900
45	8	0	-5.500679	2.861641	1.707000
46	1	0	-6.270959	4.575801	-0.337820
47	1	0	-6.741469	4.005471	-1.947830
48	1	0	-7.712279	3.575081	-0.533020
49	1	0	1.130920	-1.118350	-1.642080
50	6	0	2.856050	0.011400	-1.127200
51	6	0	3.168190	-0.028130	-2.631140

52	6	0	3.194840	1.419780	-0.571040
53	1	0	4.228380	0.115619	-2.842340
54	1	0	2.882260	-0.980820	-3.077110
55	1	0	2.620390	0.766020	-3.146160
56	6	0	4.665521	1.862089	-0.693340
57	6	0	4.816781	3.290269	-0.224560
58	8	0	4.411791	4.258449	-0.820990
59	8	0	5.447811	3.367929	0.967340
60	6	0	5.604701	4.694079	1.502700
61	6	0	4.585070	-2.012561	-1.009740
62	1	0	4.118960	-0.647491	0.527610
63	6	0	4.253150	-3.077471	-1.738220
64	6	0	6.029460	-1.615791	-0.854530
65	1	0	5.009930	-3.668581	-2.240600
66	1	0	3.229320	-3.406640	-1.873380
67	1	0	6.693570	-2.308821	-1.372350
68	1	0	6.214040	-0.612691	-1.250240
69	1	0	6.304170	-1.596021	0.204370
70	1	0	4.989811	1.840249	-1.735200
71	1	0	5.322800	1.220579	-0.107890
72	1	0	2.900960	1.465040	0.477310
73	1	0	2.587481	2.155870	-1.103630
74	8	0	4.003320	-2.530911	2.151920
75	1	0	2.135580	-3.249690	2.187750
76	6	0	1.244370	-0.330700	2.815500
77	1	0	0.414860	-1.019310	2.994860
78	1	0	2.170240	-0.770030	3.195620
79	1	0	1.055380	0.595500	3.358690
80	6	0	-4.909700	-2.825169	0.277190
81	1	0	-5.902290	-2.535749	0.626940
82	1	0	-5.035740	-3.314299	-0.692130
83	1	0	-4.538120	-3.569759	0.981200
84	1	0	6.121201	4.565929	2.450540
85	1	0	6.193451	5.310549	0.823850
86	1	0	4.631551	5.160419	1.654470

22

Center Number	Atomic Number	Atomic Type	Coordinates (Angstroms) X	Y	Z
1	6	0	-0.722656	0.217965	-1.064127
2	6	0	0.656623	0.856726	-0.809889
3	1	0	-0.697104	-0.198818	-2.071727
4	1	0	-1.480050	1.006196	-1.072512
5	1	0	0.655230	1.447287	0.107829
6	1	0	0.865757	1.541972	-1.635528
7	6	0	3.858235	-1.142587	0.056700
8	6	0	2.698111	-2.135409	0.283097
9	6	0	1.460499	-1.191039	0.439459
10	6	0	1.720395	-0.235923	-0.742694
11	6	0	3.015856	-3.113638	1.393740
12	1	0	2.495800	-2.737333	-0.604808
13	8	0	1.552889	-0.378993	1.627422
14	6	0	-1.754785	-3.244864	0.216094
15	6	0	-2.193734	-1.965906	-0.562495
16	6	0	-1.119677	-0.880875	-0.040749
17	6	0	0.082570	-1.789467	0.293159
18	6	0	-0.284678	-3.058317	0.444062
19	1	0	-2.277409	-3.329949	1.177695
20	1	0	-1.981952	-4.160585	-0.336347
21	6	0	-1.999909	-2.266771	-2.062469
22	6	0	-1.597159	-0.186232	1.256972
23	1	0	0.377927	-3.869882	0.715496
24	1	0	-2.277806	-1.430676	-2.704705
25	1	0	-0.960586	-2.520825	-2.278847
26	1	0	-2.607850	-3.123287	-2.359406
27	1	0	-0.808116	0.451048	1.653557
28	1	0	-2.475076	0.437567	1.084446
29	1	0	-1.852346	-0.910797	2.032810
30	6	0	-3.690135	-1.625025	-0.264309
31	6	0	-4.211336	-0.402630	-1.052354
32	6	0	-5.487772	0.219771	-0.510361
33	6	0	-6.024717	1.409013	-1.320716
34	6	0	-6.668842	2.308794	-0.258878
35	6	0	-5.909396	1.929344	1.010286
36	8	0	-5.237119	0.763939	0.817904
37	1	0	-6.265769	-0.538509	-0.388117
38	1	0	-6.721263	1.088717	-2.096868
39	1	0	-5.193665	1.925386	-1.808155
40	1	0	-6.489792	3.367822	-0.449410
41	6	0	-8.175820	2.088173	-0.061235
42	1	0	-3.750907	-1.374242	0.796450
43	1	0	-4.408890	-0.687974	-2.091736
44	1	0	-3.458182	0.386166	-1.086923
45	8	0	-5.882181	2.519359	2.053074
46	1	0	-8.540109	2.670813	0.785850
47	1	0	-8.722448	2.397970	-0.954232
48	1	0	-8.406518	1.036700	0.127869
49	1	0	1.562695	-0.862154	-1.629263
50	6	0	3.248316	0.096089	-0.749528
51	6	0	3.726980	0.139365	-2.209635

52	6	0	3.655769	1.391217	0.004330
53	1	0	4.772243	0.443984	-2.296289
54	1	0	3.631037	-0.838389	-2.684246
55	1	0	3.128976	0.845744	-2.791065
56	6	0	3.401910	2.731825	-0.690963
57	6	0	4.051978	3.887451	0.039850
58	8	0	4.951664	3.805493	0.838766
59	8	0	3.507645	5.065105	-0.339891
60	6	0	4.085936	6.240195	0.255549
61	6	0	5.139438	-1.752584	-0.481713
62	1	0	4.105855	-0.747395	1.048094
63	6	0	5.190436	-2.924541	-1.115359
64	6	0	6.405151	-0.974215	-0.218348
65	1	0	6.134554	-3.325173	-1.466231
66	1	0	4.315968	-3.532991	-1.305674
67	1	0	6.545320	-0.814018	0.855519
68	1	0	7.280110	-1.499443	-0.602949
69	1	0	6.378797	0.017322	-0.677610
70	1	0	2.343326	2.960827	-0.804086
71	1	0	3.822716	2.741921	-1.701733
72	1	0	4.726818	1.338749	0.208950
73	1	0	3.166015	1.394145	0.979042
74	8	0	2.591092	-4.241591	1.463190
75	1	0	3.720750	-2.743223	2.166824
76	6	0	1.336515	-1.007380	2.882931
77	1	0	0.523608	-1.735772	2.843483
78	1	0	2.242255	-1.504261	3.246467
79	1	0	1.073707	-0.217036	3.586404
80	6	0	-4.625853	-2.827864	-0.487517
81	1	0	-5.644468	-2.590729	-0.173450
82	1	0	-4.671058	-3.121973	-1.539082
83	1	0	-4.315147	-3.698003	0.090648
84	1	0	3.976589	6.210745	1.339286
85	1	0	5.144183	6.313002	0.005465
86	1	0	3.534865	7.078894	-0.162259

23

Center Number	Atomic Number	Atomic Type	Coordinates (Angstroms) X	Y	Z
1	6	0	-1.263002	0.287461	-1.157174
2	6	0	0.076790	1.046069	-1.107159
3	1	0	-1.279654	-0.270106	-2.094414
4	1	0	-2.079020	1.013032	-1.211665
5	1	0	0.098134	1.748458	-0.273310
6	1	0	0.175626	1.634725	-2.024555
7	6	0	3.489709	-0.664572	-0.375596
8	6	0	2.411608	-1.633413	0.177672
9	6	0	1.134975	-0.747436	0.331194
10	6	0	1.219365	0.040710	-0.989516
11	6	0	2.923352	-2.393579	1.382844
12	1	0	2.139953	-2.390591	-0.557999
13	8	0	1.260669	0.224899	1.384600
14	6	0	-1.931839	-3.001426	0.698505
15	6	0	-2.521153	-1.887247	-0.220433
16	6	0	-1.493460	-0.672115	0.042818
17	6	0	-0.203804	-1.444629	0.395783
18	6	0	-0.465644	-2.694897	0.764659
19	1	0	-2.371173	-2.968771	1.704025
20	1	0	-2.134049	-4.003394	0.310624
21	6	0	-2.418305	-2.402768	-1.669981
22	6	0	-1.912798	0.174155	1.268419
23	1	0	0.273453	-3.414410	1.093125
24	1	0	-2.794570	-1.690553	-2.404702
25	1	0	-1.383655	-2.633541	-1.929893
26	1	0	-2.992612	-3.323882	-1.784140
27	1	0	-1.141570	0.906941	1.500974
28	1	0	-2.842802	0.715504	1.089907
29	1	0	-2.053874	-0.443505	2.157383
30	6	0	-4.013668	-1.597291	0.148598
31	6	0	-4.681110	-0.550637	-0.770314
32	6	0	-5.986309	0.039503	-0.241988
33	6	0	-6.604909	1.119608	-1.149186
34	6	0	-6.086082	2.434906	-0.559732
35	6	0	-5.798438	2.066250	0.893190
36	8	0	-5.755112	0.715745	1.028360
37	1	0	-6.703696	-0.749960	-0.021755
38	1	0	-7.695187	1.088438	-1.079269
39	1	0	-6.334137	0.976754	-2.195646
40	1	0	-5.110623	2.680957	-0.995981
41	6	0	-7.002133	3.647039	-0.693744
42	1	0	-4.017398	-1.189687	1.161092
43	1	0	-4.907669	-1.004743	-1.741033
44	1	0	-3.994635	0.274245	-0.971024
45	8	0	-5.617112	2.808115	1.817029
46	1	0	-6.586726	4.500620	-0.157131
47	1	0	-7.126829	3.923340	-1.742870
48	1	0	-7.990277	3.436552	-0.277759
49	1	0	1.043118	-0.715011	-1.764728
50	6	0	2.707575	0.480873	-1.176865
51	6	0	3.016562	0.498630	-2.682183

52	6	0	2.924435	1.897336	-0.575755
53	1	0	4.047132	0.780265	-2.902161
54	1	0	2.849890	-0.480013	-3.133947
55	1	0	2.367251	1.218736	-3.188666
56	6	0	4.302739	2.562833	-0.698384
57	6	0	5.263412	2.312080	0.444313
58	8	0	5.009255	1.763378	1.488492
59	8	0	6.477938	2.832761	0.164379
60	6	0	7.464661	2.719670	1.205222
61	6	0	4.624318	-1.373029	-1.105274
62	1	0	3.938605	-0.173673	0.490967
63	6	0	4.498720	-2.552651	-1.713677
64	6	0	5.966793	-0.686505	-1.076365
65	1	0	5.345799	-3.013534	-2.208669
66	1	0	3.572058	-3.110235	-1.745518
67	1	0	6.725731	-1.278008	-1.589721
68	1	0	5.934337	0.297114	-1.551666
69	1	0	6.293841	-0.526904	-0.044517
70	1	0	4.170210	3.650485	-0.716883
71	1	0	4.816193	2.335617	-1.634301
72	1	0	2.639881	1.883017	0.474905
73	1	0	2.216840	2.560286	-1.079807
74	8	0	2.581574	-3.510947	1.688002
75	1	0	3.691424	-1.868079	1.986904
76	6	0	1.234243	-0.236263	2.727831
77	1	0	0.943454	0.614491	3.344543
78	1	0	0.513229	-1.044422	2.870880
79	1	0	2.223312	-0.573207	3.053701
80	6	0	-4.871818	-2.876170	0.189787
81	1	0	-5.875729	-2.657519	0.558955
82	1	0	-4.980326	-3.329526	-0.798855
83	1	0	-4.452023	-3.627627	0.858489
84	1	0	7.664082	1.672087	1.429445
85	1	0	8.356291	3.202888	0.814066
86	1	0	7.122000	3.219773	2.110668

24

Center Number	Atomic Number	Atomic Type	Coordinates (Angstroms) X	Y	Z
1	6	0	-0.924902	0.011902	-1.177020
2	6	0	0.444043	0.721895	-1.162551
3	1	0	-0.970013	-0.564295	-2.101632
4	1	0	-1.714110	0.765484	-1.241735
5	1	0	0.494111	1.469096	-0.369760
6	1	0	0.565810	1.255001	-2.109665
7	6	0	3.788214	-0.929571	-0.207312
8	6	0	2.718065	-1.922393	0.302500
9	6	0	1.433069	-1.031675	0.371395
10	6	0	1.537913	-0.327396	-0.995312
11	6	0	3.168916	-2.641331	1.555628
12	1	0	2.502591	-2.698417	-0.435053
13	8	0	1.556327	-0.003670	1.374635
14	6	0	-1.677983	-3.216027	0.764081
15	6	0	-2.228042	-2.122098	-0.203030
16	6	0	-1.186512	-0.914647	0.045137
17	6	0	0.082668	-1.700196	0.448248
18	6	0	-0.208438	-2.932328	0.853275
19	1	0	-2.139155	-3.144337	1.757844
20	1	0	-1.888188	-4.226281	0.403215
21	6	0	-2.100719	-2.684557	-1.633073
22	6	0	-1.617332	-0.028283	1.237777
23	1	0	0.511339	-3.652713	1.220510
24	1	0	-2.458930	-1.994668	-2.397481
25	1	0	-1.062763	-2.928967	-1.866028
26	1	0	-2.678139	-3.605718	-1.728762
27	1	0	-0.846565	0.709707	1.455755
28	1	0	-2.541199	0.511710	1.028380
29	1	0	-1.775330	-0.615867	2.144016
30	6	0	-3.723060	-1.798883	0.124381
31	6	0	-4.351939	-0.759998	-0.829678
32	6	0	-5.662177	-0.144529	-0.343741
33	6	0	-6.234742	0.941664	-1.273437
34	6	0	-5.715229	2.251457	-0.671864
35	6	0	-5.475850	1.883546	0.789843
36	8	0	-5.456834	0.532847	0.930263
37	1	0	-6.399047	-0.920818	-0.141683
38	1	0	-7.327009	0.927122	-1.238772
39	1	0	-5.932575	0.791185	-2.310194
40	1	0	-4.723845	2.482758	-1.079257
41	6	0	-6.609952	3.475761	-0.836159
42	1	0	-3.742405	-1.370338	1.128058
43	1	0	-4.559780	-1.226879	-1.798537
44	1	0	-3.649912	0.052689	-1.026434
45	8	0	-5.309556	2.625142	1.716702
46	1	0	-6.198543	4.325112	-0.289883
47	1	0	-6.699651	3.750559	-1.889191
48	1	0	-7.612768	3.280250	-0.448944
49	1	0	1.288195	-1.121667	-1.707802
50	6	0	3.049292	-0.008703	-1.270921
51	6	0	3.352416	-0.452454	-2.715855

52	6	0	3.428790	1.492103	-1.192523
53	1	0	4.389670	-0.262247	-2.997074
54	1	0	3.163177	-1.518000	-2.857436
55	1	0	2.715898	0.097392	-3.415145
56	6	0	3.417102	2.195466	0.164615
57	6	0	3.562679	3.694878	0.036094
58	8	0	3.428878	4.339870	-0.974796
59	8	0	3.848054	4.254603	1.233708
60	6	0	3.958262	5.688538	1.241317
61	6	0	5.102729	-1.552870	-0.636267
62	1	0	4.021647	-0.289086	0.648957
63	6	0	5.245534	-2.842791	-0.940827
64	6	0	6.292199	-0.625211	-0.660324
65	1	0	6.211631	-3.242170	-1.227310
66	1	0	4.426486	-3.549894	-0.916846
67	1	0	7.201270	-1.151085	-0.953761
68	1	0	6.144285	0.206357	-1.354376
69	1	0	6.458098	-0.181702	0.327107
70	1	0	4.214203	1.844237	0.823399
71	1	0	2.490531	2.005332	0.710081
72	1	0	2.764966	2.040492	-1.864119
73	1	0	4.424554	1.616184	-1.627444
74	8	0	2.805597	-3.744337	1.885705
75	1	0	3.909828	-2.096944	2.177124
76	6	0	1.456847	-0.386530	2.739582
77	1	0	1.210521	0.515931	3.299555
78	1	0	0.674947	-1.131370	2.900790
79	1	0	2.405901	-0.779169	3.118940
80	6	0	-4.603658	-3.062185	0.172755
81	1	0	-5.609471	-2.820209	0.521363
82	1	0	-4.703772	-3.530559	-0.809686
83	1	0	-4.208150	-3.809250	0.860882
84	1	0	4.758284	6.012942	0.576259
85	1	0	3.021614	6.144547	0.921270
86	1	0	4.182813	5.957610	2.270355

25

Center Number	Atomic Number	Atomic Type	Coordinates (Angstroms) X	Y	Z
1	6	0	-0.960672	0.216039	-1.156766
2	6	0	0.435481	0.859981	-1.054185
3	1	0	-0.982700	-0.352123	-2.087514
4	1	0	-1.708649	1.006874	-1.256627
5	1	0	0.482298	1.578226	-0.234891
6	1	0	0.630336	1.417404	-1.975493
7	6	0	3.653015	-1.092603	-0.110954
8	6	0	2.477561	-1.976598	0.395062
9	6	0	1.270851	-0.984345	0.466332
10	6	0	1.478481	-0.236318	-0.866440
11	6	0	2.863408	-2.736518	1.642851
12	1	0	2.200406	-2.728946	-0.344552
13	8	0	1.440340	-0.006389	1.510473
14	6	0	-1.978924	-2.979843	0.712067
15	6	0	-2.428453	-1.842395	-0.257195
16	6	0	-1.326587	-0.702644	0.042596
17	6	0	-0.121945	-1.566722	0.477659
18	6	0	-0.498875	-2.784618	0.854332
19	1	0	-2.468784	-2.891496	1.690683
20	1	0	-2.237362	-3.971205	0.330536
21	6	0	-2.286946	-2.392410	-1.690812
22	6	0	-1.744477	0.198199	1.229185
23	1	0	0.162832	-3.552775	1.234261
24	1	0	-2.577856	-1.671860	-2.455675
25	1	0	-1.257963	-2.694968	-1.893376
26	1	0	-2.913868	-3.276442	-1.819896
27	1	0	-0.930918	0.872928	1.492482
28	1	0	-2.615805	0.808305	0.988157
29	1	0	-1.984124	-0.388174	2.118365
30	6	0	-3.910351	-1.431904	0.024809
31	6	0	-4.441467	-0.349171	-0.939900
32	6	0	-5.693674	0.376045	-0.474330
33	6	0	-6.240485	1.419856	-1.459526
34	6	0	-6.842339	2.496656	-0.548380
35	6	0	-6.051151	2.323967	0.746316
36	8	0	-5.396957	1.132350	0.735007
37	1	0	-6.476600	-0.337575	-0.204245
38	1	0	-6.963009	0.984018	-2.151094
39	1	0	-5.418219	1.832737	-2.049443
40	1	0	-6.655497	3.505083	-0.919917
41	6	0	-8.345381	2.338671	-0.275583
42	1	0	-3.934912	-1.009593	1.031214
43	1	0	-4.673444	-0.799306	-1.911730
44	1	0	-3.681103	0.410656	-1.126923
45	8	0	-5.988484	3.079590	1.674252
46	1	0	-8.677381	3.063355	0.468973
47	1	0	-8.914399	2.500833	-1.193262
48	1	0	-8.582982	1.338955	0.096900
49	1	0	1.265344	-0.993946	-1.630710
50	6	0	3.001324	0.064527	-1.001109
51	6	0	3.400677	0.026490	-2.486214

52	6	0	3.349670	1.462573	-0.426790
53	1	0	4.482970	0.030684	-2.621585
54	1	0	3.009247	-0.855897	-2.993968
55	1	0	2.997068	0.901281	-3.003745
56	6	0	4.822522	1.867881	-0.503419
57	6	0	5.062844	3.273140	0.002794
58	8	0	4.221809	4.121197	0.167692
59	8	0	6.378610	3.483667	0.236105
60	6	0	6.728358	4.806856	0.678433
61	6	0	4.792259	-1.910728	-0.701524
62	1	0	4.061568	-0.594287	0.771448
63	6	0	6.052423	-1.688022	-0.326818
64	6	0	4.477064	-3.032048	-1.663273
65	1	0	6.875048	-2.266834	-0.731183
66	1	0	6.309224	-0.927279	0.401251
67	1	0	5.388447	-3.409080	-2.128514
68	1	0	4.001762	-3.870187	-1.144408
69	1	0	3.794448	-2.723380	-2.456363
70	1	0	5.195836	1.845698	-1.532203
71	1	0	5.471715	1.194490	0.057666
72	1	0	3.020361	1.514847	0.610434
73	1	0	2.768421	2.213744	-0.965804
74	8	0	2.509486	-3.862707	1.898645
75	1	0	3.554522	-2.206765	2.330193
76	6	0	1.217970	-0.407794	2.855055
77	1	0	1.050486	0.505761	3.425857
78	1	0	0.344707	-1.055650	2.953168
79	1	0	2.088908	-0.921592	3.274710
80	6	0	-4.866743	-2.639520	0.032592
81	1	0	-5.872846	-2.335803	0.328781
82	1	0	-4.946145	-3.106205	-0.952446
83	1	0	-4.550783	-3.405356	0.741004
84	1	0	7.807470	4.794147	0.809289
85	1	0	6.440751	5.547016	-0.068017
86	1	0	6.230116	5.037936	1.619631

26

Center Number	Atomic Number	Atomic Type	Coordinates (Angstroms) X	Y	Z
1	6	0	-0.902836	0.191249	-1.148367
2	6	0	0.479725	0.862051	-1.033472
3	1	0	-0.911433	-0.363153	-2.087529
4	1	0	-1.666756	0.968000	-1.237511
5	1	0	0.509075	1.566162	-0.200992
6	1	0	0.663620	1.438780	-1.945227
7	6	0	3.736081	-1.044979	-0.120664
8	6	0	2.576355	-1.956549	0.372578
9	6	0	1.350663	-0.989332	0.457459
10	6	0	1.545651	-0.214925	-0.861995
11	6	0	2.976002	-2.729302	1.608875
12	1	0	2.313233	-2.702581	-0.378558
13	8	0	1.497676	-0.023184	1.515608
14	6	0	-1.859206	-3.052360	0.668648
15	6	0	-2.331067	-1.908521	-0.282076
16	6	0	-1.251247	-0.752731	0.036179
17	6	0	-0.030257	-1.599629	0.457739
18	6	0	-0.383240	-2.830721	0.814250
19	1	0	-2.350148	-2.989588	1.648665
20	1	0	-2.098470	-4.042325	0.271320
21	6	0	-2.179673	-2.432511	-1.724420
22	6	0	-1.687773	0.121206	1.236137
23	1	0	0.293673	-3.591627	1.181833
24	1	0	-2.482024	-1.704348	-2.477534
25	1	0	-1.145847	-2.715145	-1.931071
26	1	0	-2.791938	-3.324406	-1.868537
27	1	0	-0.886355	0.804816	1.513072
28	1	0	-2.569059	0.719844	1.002564
29	1	0	-1.918742	-0.483703	2.115141
30	6	0	-3.820794	-1.530965	0.005381
31	6	0	-4.374953	-0.450032	-0.948835
32	6	0	-5.625984	0.263670	-0.462894
33	6	0	-6.210160	1.287234	-1.447895
34	6	0	-6.802393	2.369255	-0.536763
35	6	0	-5.975505	2.225223	0.738889
36	8	0	-5.310243	1.039812	0.729183
37	1	0	-6.392097	-0.456863	-0.164411
38	1	0	-6.944073	0.833125	-2.115269
39	1	0	-5.408013	1.701782	-2.063847
40	1	0	-6.638668	3.374586	-0.927163
41	6	0	-8.295653	2.195891	-0.222634
42	1	0	-3.853593	-1.119959	1.016186
43	1	0	-4.616846	-0.900704	-1.917966
44	1	0	-3.624518	0.317248	-1.145516
45	8	0	-5.894968	2.996693	1.652362
46	1	0	-8.617581	2.926024	0.521017
47	1	0	-8.890367	2.338979	-1.127089
48	1	0	-8.510618	1.198205	0.168507
49	1	0	1.351183	-0.962718	-1.640756
50	6	0	3.062930	0.118290	-0.986052
51	6	0	3.468525	0.129184	-2.469225

52	6	0	3.385389	1.505012	-0.369501
53	1	0	4.551200	0.155777	-2.597991
54	1	0	3.093740	-0.745373	-3.002219
55	1	0	3.053544	1.011350	-2.964931
56	6	0	4.855862	1.957544	-0.479061
57	6	0	5.065992	3.243378	0.282509
58	8	0	5.427261	3.321705	1.431302
59	8	0	4.768236	4.324621	-0.473269
60	6	0	4.890199	5.597272	0.185884
61	6	0	4.884451	-1.836299	-0.730122
62	1	0	4.141998	-0.556770	0.768337
63	6	0	6.140787	-1.612607	-0.343268
64	6	0	4.582284	-2.936556	-1.720164
65	1	0	6.969898	-2.174575	-0.758110
66	1	0	6.387206	-0.870193	0.407038
67	1	0	5.498424	-3.293303	-2.191985
68	1	0	4.114218	-3.791845	-1.223098
69	1	0	3.898502	-2.615935	-2.507509
70	1	0	5.137992	2.111533	-1.520931
71	1	0	5.524822	1.218001	-0.042387
72	1	0	3.093226	1.499574	0.680196
73	1	0	2.766740	2.256532	-0.867472
74	8	0	2.631875	-3.861468	1.851205
75	1	0	3.666798	-2.203092	2.298808
76	6	0	1.294102	-0.451485	2.855305
77	1	0	0.436265	-1.120510	2.947974
78	1	0	2.179850	-0.950874	3.260334
79	1	0	1.110563	0.448671	3.442035
80	6	0	-4.753351	-2.757084	0.000027
81	1	0	-5.768262	-2.473635	0.286714
82	1	0	-4.813975	-3.221304	-0.987429
83	1	0	-4.430112	-3.519260	0.709128
84	1	0	4.615406	6.339308	-0.559544
85	1	0	4.219944	5.647459	1.043721
86	1	0	5.914245	5.753348	0.524144

27

Center Number	Atomic Number	Atomic Type	Coordinates (Angstroms) X	Y	Z
1	6	0	-0.801400	0.042420	-1.213640
2	6	0	0.576370	0.734840	-1.203740
3	1	0	-0.842870	-0.561710	-2.120590
4	1	0	-1.579320	0.804120	-1.310590
5	1	0	0.627190	1.503870	-0.432320
6	1	0	0.716060	1.238740	-2.164320
7	6	0	3.887610	-0.930970	-0.163510
8	6	0	2.798980	-1.896110	0.360300
9	6	0	1.525570	-0.986320	0.391870
10	6	0	1.654450	-0.322610	-0.993080
11	6	0	3.227440	-2.588610	1.635880
12	1	0	2.581230	-2.687800	-0.359660
13	8	0	1.654620	0.067620	1.367230
14	6	0	-1.621260	-3.111690	0.821330
15	6	0	-2.144450	-2.042310	-0.186720
16	6	0	-1.088780	-0.841650	0.034080
17	6	0	0.164620	-1.632500	0.474940
18	6	0	-0.148360	-2.846870	0.915880
19	1	0	-2.090360	-2.999940	1.807660
20	1	0	-1.844420	-4.129880	0.491680
21	6	0	-2.008790	-2.651750	-1.596800
22	6	0	-1.521390	0.088750	1.192160
23	1	0	0.557060	-3.565460	1.313080
24	1	0	-2.349540	-1.981610	-2.386350
25	1	0	-0.971160	-2.915000	-1.809640
26	1	0	-2.595720	-3.569020	-1.670740
27	1	0	-0.741960	0.821710	1.397060
28	1	0	-2.434810	0.634270	0.953000
29	1	0	-1.697240	-0.468890	2.114190
30	6	0	-3.637590	-1.685830	0.110720
31	6	0	-4.241290	-0.685170	-0.899050
32	6	0	-5.505170	0.021050	-0.436480
33	6	0	-6.131020	0.967920	-1.471350
34	6	0	-6.741090	2.085840	-0.617070
35	6	0	-5.895540	2.039320	0.653550
36	8	0	-5.196030	0.875030	0.703250
37	1	0	-6.246420	-0.701420	-0.084600
38	1	0	-6.861010	0.455170	-2.099380
39	1	0	-5.349340	1.366610	-2.123000
40	1	0	-6.609080	3.070340	-1.067930
41	6	0	-8.225470	1.892470	-0.273730
42	1	0	-3.659950	-1.209400	1.092800
43	1	0	-4.485410	-1.203790	-1.832970
44	1	0	-3.517420	0.089140	-1.157520
45	8	0	-5.826830	2.864290	1.520140
46	1	0	-8.557790	2.655240	0.431600
47	1	0	-8.834270	1.968540	-1.176940
48	1	0	-8.409530	0.913380	0.175820
49	1	0	1.403010	-1.133940	-1.685430
50	6	0	3.172550	-0.029690	-1.259540
51	6	0	3.486050	-0.514140	-2.689140

52	6	0	3.570240	1.467920	-1.216400
53	1	0	4.528410	-0.342950	-2.963840
54	1	0	3.285630	-1.580630	-2.806250
55	1	0	2.863670	0.025100	-3.409110
56	6	0	3.551990	2.207710	0.121100
57	6	0	3.729250	3.699900	-0.045130
58	8	0	3.637050	4.318300	-1.077010
59	8	0	3.991840	4.287980	1.144230
60	6	0	4.131220	5.719030	1.114870
61	6	0	5.198660	-1.581540	-0.560850
62	1	0	4.119240	-0.271140	0.678380
63	6	0	5.328860	-2.880390	-0.831150
64	6	0	6.399600	-0.669060	-0.595700
65	1	0	6.293070	-3.298510	-1.096490
66	1	0	4.501050	-3.576820	-0.798770
67	1	0	7.304970	-1.212720	-0.867410
68	1	0	6.268230	0.147140	-1.311050
69	1	0	6.561450	-0.203580	0.382250
70	1	0	4.332670	1.859650	0.801020
71	1	0	2.614480	2.048870	0.657550
72	1	0	2.921010	2.006180	-1.910110
73	1	0	4.572230	1.567710	-1.642970
74	8	0	2.845140	-3.677400	1.990960
75	1	0	3.970820	-2.039170	2.249990
76	6	0	1.533400	-0.274730	2.740960
77	1	0	0.728090	-0.991030	2.915930
78	1	0	2.466920	-0.685270	3.139660
79	1	0	1.310200	0.650040	3.273540
80	6	0	-4.535750	-2.933340	0.209290
81	1	0	-5.551050	-2.657590	0.502120
82	1	0	-4.607280	-3.463910	-0.743510
83	1	0	-4.175870	-3.638170	0.958990
84	1	0	4.335540	6.012580	2.141440
85	1	0	4.953950	6.008710	0.461600
86	1	0	3.212330	6.184350	0.758700

28

Center Number	Atomic Number	Atomic Type	Coordinates (Angstroms) X	Y	Z
1	6	0	-1.033548	0.162012	-1.105223
2	6	0	0.345559	0.839687	-0.987782
3	1	0	-1.050885	-0.363228	-2.060693
4	1	0	-1.804713	0.934920	-1.160910
5	1	0	0.379866	1.518508	-0.134773
6	1	0	0.513384	1.444725	-1.884075
7	6	0	3.627110	-1.067123	-0.168395
8	6	0	2.480317	-2.001532	0.311437
9	6	0	1.247792	-1.047948	0.436353
10	6	0	1.421837	-0.233647	-0.861640
11	6	0	2.899172	-2.805465	1.521292
12	1	0	2.215062	-2.727990	-0.457845
13	8	0	1.395960	-0.111381	1.520737
14	6	0	-1.940742	-3.146778	0.614662
15	6	0	-2.435984	-1.974190	-0.288387
16	6	0	-1.360128	-0.821036	0.053335
17	6	0	-0.127481	-1.670593	0.432331
18	6	0	-0.465540	-2.916142	0.750678
19	1	0	-2.420121	-3.125376	1.601983
20	1	0	-2.174730	-4.124083	0.184226
21	6	0	-2.302553	-2.448163	-1.749667
22	6	0	-1.786898	0.010355	1.286285
23	1	0	0.222733	-3.682434	1.084555
24	1	0	-2.616209	-1.695627	-2.473566
25	1	0	-1.271181	-2.721887	-1.979695
26	1	0	-2.915542	-3.335803	-1.915106
27	1	0	-0.987620	0.691273	1.574864
28	1	0	-2.675179	0.610060	1.083661
29	1	0	-2.004129	-0.623044	2.148346
30	6	0	-3.924715	-1.620145	0.034547
31	6	0	-4.503186	-0.510084	-0.870370
32	6	0	-5.788611	0.136787	-0.359600
33	6	0	-6.319776	1.277755	-1.247412
34	6	0	-5.750781	2.543371	-0.598454
35	6	0	-5.525857	2.112893	0.848344
36	8	0	-5.556891	0.757944	0.938269
37	1	0	-6.554329	-0.617935	-0.184808
38	1	0	-7.411823	1.303338	-1.211896
39	1	0	-6.023542	1.154566	-2.289443
40	1	0	-4.750972	2.751148	-0.997972
41	6	0	-6.597421	3.806579	-0.716227
42	1	0	-3.947205	-1.247110	1.059961
43	1	0	-4.725598	-0.921300	-1.860986
44	1	0	-3.766340	0.280523	-1.026430
45	8	0	-5.332102	2.812973	1.801662
46	1	0	-6.154391	4.618063	-0.137997
47	1	0	-6.675186	4.124571	-1.758021
48	1	0	-7.607532	3.635399	-0.336558
49	1	0	1.223823	-0.959485	-1.660018
50	6	0	2.935279	0.114962	-0.992965
51	6	0	3.324003	0.169874	-2.479724

52	6	0	3.253842	1.485684	-0.340391
53	1	0	4.404898	0.207883	-2.620329
54	1	0	2.949077	-0.692607	-3.031905
55	1	0	2.897496	1.062264	-2.946570
56	6	0	4.719014	1.953751	-0.454369
57	6	0	4.928343	3.217708	0.343172
58	8	0	5.292183	3.263481	1.492887
59	8	0	4.626789	4.319674	-0.380448
60	6	0	4.749324	5.573125	0.314530
61	6	0	4.774474	-1.832013	-0.812738
62	1	0	4.039890	-0.600806	0.729249
63	6	0	6.033534	-1.608048	-0.435024
64	6	0	4.469655	-2.909198	-1.827182
65	1	0	6.862046	-2.152252	-0.874028
66	1	0	6.283073	-0.883504	0.331542
67	1	0	5.383821	-3.249374	-2.314769
68	1	0	4.010032	-3.778887	-1.347539
69	1	0	3.777964	-2.573634	-2.601387
70	1	0	4.986835	2.140941	-1.494491
71	1	0	5.399087	1.206963	-0.048183
72	1	0	2.974503	1.446702	0.712094
73	1	0	2.622832	2.246159	-0.808470
74	8	0	2.565459	-3.946284	1.735657
75	1	0	3.593727	-2.293513	2.218102
76	6	0	1.218177	-0.583199	2.849819
77	1	0	0.388830	-1.289012	2.928623
78	1	0	2.126674	-1.056593	3.235145
79	1	0	1.002080	0.291954	3.462761
80	6	0	-4.846913	-2.853869	-0.002475
81	1	0	-5.853220	-2.596409	0.333634
82	1	0	-4.936545	-3.268749	-1.009605
83	1	0	-4.493060	-3.647266	0.655748
84	1	0	4.473181	6.335959	-0.409085
85	1	0	4.080353	5.598659	1.174456
86	1	0	5.773833	5.719839	0.655534

29

Center Number	Atomic Number	Atomic Type	Coordinates (Angstroms) X	Y	Z
1	6	0	-0.943782	0.160880	-1.177805
2	6	0	0.442276	0.832597	-1.131258
3	1	0	-0.971883	-0.448747	-2.081825
4	1	0	-1.707839	0.933168	-1.298672
5	1	0	0.491113	1.584905	-0.343294
6	1	0	0.609251	1.352886	-2.079214
7	6	0	3.711048	-1.028479	-0.172101
8	6	0	2.559909	-1.901405	0.403843
9	6	0	1.338976	-0.925842	0.453859
10	6	0	1.507055	-0.237694	-0.915968
11	6	0	2.983867	-2.595768	1.677553
12	1	0	2.276430	-2.692767	-0.291445
13	8	0	1.510751	0.103236	1.448137
14	6	0	-1.868919	-2.963195	0.854050
15	6	0	-2.356157	-1.881181	-0.158726
16	6	0	-1.270924	-0.709075	0.068442
17	6	0	-0.042742	-1.530238	0.520214
18	6	0	-0.390524	-2.736133	0.958938
19	1	0	-2.342077	-2.837707	1.836714
20	1	0	-2.116268	-3.975547	0.523827
21	6	0	-2.226268	-2.494700	-1.567583
22	6	0	-1.685892	0.236582	1.220657
23	1	0	0.291630	-3.474898	1.360425
24	1	0	-2.541389	-1.814931	-2.359572
25	1	0	-1.194781	-2.787210	-1.772135
26	1	0	-2.838178	-3.395067	-1.646841
27	1	0	-0.883785	0.941475	1.436801
28	1	0	-2.576234	0.814360	0.969836
29	1	0	-1.893955	-0.314170	2.140044
30	6	0	-3.841130	-1.484935	0.127999
31	6	0	-4.411014	-0.469638	-0.886516
32	6	0	-5.669778	0.256184	-0.440832
33	6	0	-6.261292	1.220728	-1.479413
34	6	0	-6.871134	2.339852	-0.626634
35	6	0	-6.047845	2.271715	0.657692
36	8	0	-5.365833	1.096876	0.709865
37	1	0	-6.429854	-0.455376	-0.107543
38	1	0	-6.986441	0.724145	-2.125785
39	1	0	-5.461322	1.613385	-2.112308
40	1	0	-6.718750	3.326118	-1.067032
41	6	0	-8.363446	2.162797	-0.310045
42	1	0	-3.857138	-1.006530	1.109298
43	1	0	-4.649510	-0.979399	-1.826731
44	1	0	-3.669482	0.293015	-1.129448
45	8	0	-5.982086	3.088814	1.531790
46	1	0	-8.698248	2.924539	0.395207
47	1	0	-8.955642	2.253085	-1.222920
48	1	0	-8.567342	1.182870	0.128996
49	1	0	1.290361	-1.032462	-1.640302
50	6	0	3.022248	0.076799	-1.098502
51	6	0	3.389554	-0.018858	-2.588708

52	6	0	3.363401	1.503119	-0.592342
53	1	0	4.468336	-0.007157	-2.748052
54	1	0	2.998017	-0.926992	-3.048465
55	1	0	2.964852	0.828513	-3.133979
56	6	0	4.835641	1.933475	-0.744884
57	6	0	5.007586	3.358084	-0.275391
58	8	0	4.658667	4.335613	-0.891203
59	8	0	5.582243	3.421679	0.946658
60	6	0	5.746048	4.744828	1.487800
61	6	0	4.846172	-1.860233	-0.751590
62	1	0	4.135735	-0.484137	0.674520
63	6	0	6.110927	-1.606705	-0.413594
64	6	0	4.525179	-3.027078	-1.655306
65	1	0	6.932039	-2.193869	-0.808893
66	1	0	6.372439	-0.811003	0.274259
67	1	0	5.431821	-3.414557	-2.121175
68	1	0	4.070456	-3.846480	-1.090137
69	1	0	3.823681	-2.762887	-2.447978
70	1	0	5.136109	1.907678	-1.792862
71	1	0	5.499626	1.283183	-0.178003
72	1	0	3.073238	1.584830	0.455449
73	1	0	2.753798	2.220885	-1.146894
74	8	0	2.648059	-3.711470	1.995865
75	1	0	3.685184	-2.027031	2.321825
76	6	0	1.330031	-0.242210	2.814435
77	1	0	1.145164	0.690662	3.347504
78	1	0	0.480166	-0.911583	2.961489
79	1	0	2.226133	-0.708444	3.236397
80	6	0	-4.771856	-2.708883	0.221679
81	1	0	-5.780092	-2.408059	0.513697
82	1	0	-4.855146	-3.235143	-0.732585
83	1	0	-4.431588	-3.424803	0.969959
84	1	0	6.215762	4.606574	2.458343
85	1	0	6.379496	5.345957	0.836034
86	1	0	4.778397	5.234426	1.594781

30

Center Number	Atomic Number	Atomic Type	Coordinates (Angstroms) X	Y	Z
1	6	0	-0.916276	0.513081	-0.739347
2	6	0	0.422784	1.070123	-0.220523
3	1	0	-0.807738	0.384898	-1.816821
4	1	0	-1.692304	1.269527	-0.594144
5	1	0	0.341962	1.394303	0.818388
6	1	0	0.667229	1.952094	-0.817419
7	6	0	3.608916	-1.065194	0.310012
8	6	0	2.462888	-2.090753	0.176212
9	6	0	1.193314	-1.233819	0.490863
10	6	0	1.507976	0.007319	-0.369739
11	6	0	2.734728	-3.331111	1.000812
12	1	0	2.337634	-2.430811	-0.853396
13	8	0	1.184001	-0.772576	1.858185
14	6	0	-1.945556	-3.173077	-0.507973
15	6	0	-2.357748	-1.736594	-0.955740
16	6	0	-1.356083	-0.823147	-0.082287
17	6	0	-0.155644	-1.780269	0.090325
18	6	0	-0.501069	-3.045875	-0.126107
19	1	0	-2.535244	-3.512342	0.353652
20	1	0	-2.107385	-3.911857	-1.297409
21	6	0	-2.041426	-1.629695	-2.461186
22	6	0	-1.942638	-0.509304	1.314722
23	1	0	0.160064	-3.897217	-0.028792
24	1	0	-2.284884	-0.653364	-2.880351
25	1	0	-0.983718	-1.819729	-2.652315
26	1	0	-2.608476	-2.376135	-3.020158
27	1	0	-1.200260	-0.002629	1.929717
28	1	0	-2.818114	0.137866	1.251245
29	1	0	-2.239640	-1.417823	1.841860
30	6	0	-3.881655	-1.493421	-0.699891
31	6	0	-4.372355	-0.110729	-1.180651
32	6	0	-5.729278	0.321864	-0.629773
33	6	0	-6.168288	1.731548	-1.068762
34	6	0	-5.723459	2.631905	0.088140
35	6	0	-5.676106	1.669141	1.271363
36	8	0	-5.683342	0.386274	0.825262
37	1	0	-6.492331	-0.419861	-0.861942
38	1	0	-7.255636	1.771080	-1.171380
39	1	0	-5.732738	2.012257	-2.028005
40	1	0	-4.686564	2.953568	-0.065863
41	6	0	-6.582514	3.862553	0.359885
42	1	0	-4.031080	-1.532894	0.380551
43	1	0	-4.457247	-0.108064	-2.272687
44	1	0	-3.643974	0.662760	-0.928544
45	8	0	-5.625823	1.940299	2.437846
46	1	0	-6.238647	4.376919	1.257760
47	1	0	-6.535868	4.559703	-0.479328
48	1	0	-7.627541	3.582348	0.511889
49	1	0	1.428417	-0.358390	-1.400398
50	6	0	3.025479	0.339086	-0.177414
51	6	0	3.600712	0.777356	-1.532694

52	6	0	3.329406	1.386499	0.931198
53	1	0	4.656457	1.045072	-1.469590
54	1	0	3.511674	-0.020839	-2.271518
55	1	0	3.061744	1.645527	-1.918524
56	6	0	3.110310	2.881398	0.626089
57	6	0	4.136489	3.576051	-0.242296
58	8	0	3.878511	4.340562	-1.139553
59	8	0	5.403204	3.289696	0.135820
60	6	0	6.438680	3.962978	-0.601701
61	6	0	4.939803	-1.498621	-0.277201
62	1	0	3.777631	-0.952581	1.387153
63	6	0	5.064148	-2.448027	-1.204616
64	6	0	6.162354	-0.817585	0.287302
65	1	0	6.039884	-2.731039	-1.582518
66	1	0	4.219750	-2.981918	-1.620564
67	1	0	6.232633	-0.985895	1.367222
68	1	0	7.075140	-1.193870	-0.176083
69	1	0	6.130074	0.265468	0.143133
70	1	0	3.132873	3.414747	1.583491
71	1	0	2.140195	3.085636	0.181401
72	1	0	4.370300	1.268593	1.237941
73	1	0	2.724803	1.130518	1.801944
74	8	0	2.335191	-4.438896	0.735782
75	1	0	3.380712	-3.177890	1.890516
76	6	0	0.895201	-1.717667	2.878774
77	1	0	0.094619	-2.402528	2.591109
78	1	0	1.781146	-2.301096	3.151311
79	1	0	0.578598	-1.146547	3.751868
80	6	0	-4.764888	-2.600413	-1.306359
81	1	0	-5.809721	-2.463598	-1.021060
82	1	0	-4.723378	-2.605167	-2.398559
83	1	0	-4.473650	-3.590700	-0.956177
84	1	0	7.375033	3.625859	-0.164090
85	1	0	6.389199	3.696426	-1.657264
86	1	0	6.335205	5.043134	-0.502611

31

Center Number	Atomic Number	Atomic Type	Coordinates (Angstroms) X	Y	Z
1	6	0	-1.022009	0.150986	-1.103725
2	6	0	0.358606	0.821600	-0.971646
3	1	0	-1.037910	-0.361992	-2.065847
4	1	0	-1.789825	0.927728	-1.152178
5	1	0	0.390692	1.487886	-0.108842
6	1	0	0.531446	1.439380	-1.858324
7	6	0	3.631022	-1.117776	-0.185594
8	6	0	2.479018	-2.045202	0.283658
9	6	0	1.250096	-1.092103	0.426266
10	6	0	1.431070	-0.257242	-0.856586
11	6	0	2.896051	-2.882488	1.473791
12	1	0	2.198910	-2.753289	-0.495872
13	8	0	1.398229	-0.176635	1.528786
14	6	0	-1.952783	-3.174316	0.568076
15	6	0	-2.438848	-1.985930	-0.318851
16	6	0	-1.355861	-0.845233	0.040881
17	6	0	-0.129039	-1.708079	0.409206
18	6	0	-0.476239	-2.955655	0.709563
19	1	0	-2.433327	-3.163964	1.555010
20	1	0	-2.192684	-4.143863	0.123430
21	6	0	-2.305848	-2.440549	-1.786436
22	6	0	-1.778475	-0.029155	1.285456
23	1	0	0.206889	-3.729838	1.035102
24	1	0	-2.611707	-1.675591	-2.500574
25	1	0	-1.276099	-2.719095	-2.017897
26	1	0	-2.925364	-3.321014	-1.965420
27	1	0	-0.972880	0.638068	1.588035
28	1	0	-2.660050	0.582924	1.089786
29	1	0	-2.004763	-0.674572	2.136255
30	6	0	-3.925765	-1.625899	0.005875
31	6	0	-4.494775	-0.501800	-0.887600
32	6	0	-5.777761	0.147673	-0.374129
33	6	0	-6.297104	1.302572	-1.250889
34	6	0	-5.720559	2.556145	-0.585589
35	6	0	-5.506315	2.107911	0.857423
36	8	0	-5.547377	0.752298	0.931819
37	1	0	-6.549364	-0.603714	-0.211274
38	1	0	-7.389055	1.336287	-1.218895
39	1	0	-5.998069	1.188852	-2.293197
40	1	0	-4.716994	2.759348	-0.978004
41	6	0	-6.555378	3.828002	-0.693726
42	1	0	-3.947714	-1.264255	1.035368
43	1	0	-4.716654	-0.900112	-1.883596
44	1	0	-3.752361	0.285740	-1.032280
45	8	0	-5.312681	2.795940	1.819519
46	1	0	-6.108299	4.629010	-0.104106
47	1	0	-6.624734	4.158317	-1.732287
48	1	0	-7.568998	3.661659	-0.321326
49	1	0	1.237980	-0.968472	-1.669014
50	6	0	2.946763	0.096293	-0.973505
51	6	0	3.324284	0.152756	-2.461629

52	6	0	3.232819	1.463632	-0.299727
53	1	0	4.380975	0.369085	-2.622986
54	1	0	3.109536	-0.792001	-2.962818
55	1	0	2.750272	0.937699	-2.962122
56	6	0	4.697780	1.940059	-0.286351
57	6	0	4.808882	3.262069	0.434979
58	8	0	5.048356	3.395350	1.609933
59	8	0	4.575953	4.303437	-0.395261
60	6	0	4.610102	5.605583	0.215516
61	6	0	4.761578	-1.862480	-0.887262
62	1	0	4.066671	-0.684798	0.721643
63	6	0	4.593527	-3.006352	-1.551060
64	6	0	6.141472	-1.263086	-0.773464
65	1	0	5.434145	-3.499394	-2.025317
66	1	0	3.636903	-3.501476	-1.652726
67	1	0	6.890846	-1.912429	-1.227145
68	1	0	6.204889	-0.287642	-1.261650
69	1	0	6.414830	-1.108384	0.275392
70	1	0	5.081802	2.057939	-1.300393
71	1	0	5.329255	1.237397	0.254535
72	1	0	2.871829	1.426498	0.727572
73	1	0	2.641615	2.223922	-0.817387
74	8	0	2.482897	-3.990234	1.716245
75	1	0	3.664878	-2.424704	2.131308
76	6	0	1.247541	-0.686127	2.846744
77	1	0	2.164911	-1.168063	3.200381
78	1	0	1.041929	0.170611	3.488535
79	1	0	0.421832	-1.396960	2.921022
80	6	0	-4.855885	-2.853009	-0.047014
81	1	0	-5.861458	-2.592811	0.289228
82	1	0	-4.945299	-3.256441	-1.058819
83	1	0	-4.508500	-3.655615	0.603418
84	1	0	4.399745	6.309127	-0.586035
85	1	0	3.856262	5.677937	0.999100
86	1	0	5.592254	5.797035	0.646985

32

Center Number	Atomic Number	Atomic Type	Coordinates (Angstroms) X	Y	Z
1	6	0	-1.028842	0.533532	-0.832104
2	6	0	0.315304	1.114791	-0.356523
3	1	0	-0.926034	0.316202	-1.895760
4	1	0	-1.799728	1.304067	-0.745858
5	1	0	0.237651	1.503675	0.659450
6	1	0	0.579002	1.959169	-1.000195
7	6	0	3.512869	-1.020162	0.199092
8	6	0	2.330494	-2.024390	0.250953
9	6	0	1.076693	-1.136185	0.523194
10	6	0	1.385812	0.030634	-0.435419
11	6	0	2.626816	-3.177469	1.185854
12	1	0	2.151399	-2.472893	-0.726095
13	8	0	1.083053	-0.585369	1.855998
14	6	0	-2.088567	-3.115133	-0.303156
15	6	0	-2.493742	-1.712606	-0.854147
16	6	0	-1.473669	-0.742899	-0.065181
17	6	0	-0.281651	-1.697343	0.173298
18	6	0	-0.640465	-2.972193	0.057277
19	1	0	-2.673948	-3.385207	0.585508
20	1	0	-2.262698	-3.909531	-1.033991
21	6	0	-2.196663	-1.723492	-2.366946
22	6	0	-2.043412	-0.312222	1.307295
23	1	0	0.013831	-3.819679	0.217200
24	1	0	-2.443139	-0.781327	-2.857133
25	1	0	-1.142019	-1.931609	-2.556035
26	1	0	-2.773165	-2.508695	-2.859119
27	1	0	-1.289315	0.230959	1.874729
28	1	0	-2.910767	0.340052	1.199460
29	1	0	-2.348217	-1.171612	1.907364
30	6	0	-4.012646	-1.439451	-0.598654
31	6	0	-4.499888	-0.088643	-1.166492
32	6	0	-5.848534	0.388938	-0.632867
33	6	0	-6.282677	1.768787	-1.161760
34	6	0	-5.810516	2.741787	-0.076761
35	6	0	-5.762942	1.863204	1.170120
36	8	0	-5.788375	0.552558	0.814076
37	1	0	-6.618358	-0.361688	-0.806680
38	1	0	-7.371333	1.811966	-1.248413
39	1	0	-5.861614	1.978893	-2.145318
40	1	0	-4.770851	3.035306	-0.265198
41	6	0	-6.647882	4.001693	0.117017
42	1	0	-4.149623	-1.404884	0.483632
43	1	0	-4.596266	-0.158810	-2.255258
44	1	0	-3.764625	0.695304	-0.974488
45	8	0	-5.699694	2.213822	2.314530
46	1	0	-6.285066	4.573605	0.971650
47	1	0	-6.602232	4.636671	-0.770254
48	1	0	-7.694803	3.749142	0.301202
49	1	0	1.295817	-0.415158	-1.433584
50	6	0	2.898939	0.380927	-0.277727
51	6	0	3.441065	0.846651	-1.636752

52	6	0	3.080109	1.490111	0.792393
53	1	0	4.510205	1.062962	-1.610385
54	1	0	3.289947	0.085341	-2.403581
55	1	0	2.931940	1.759766	-1.952627
56	6	0	4.503019	1.984379	1.088934
57	6	0	5.030461	3.017362	0.117217
58	8	0	4.377693	3.893257	-0.393596
59	8	0	6.361275	2.877927	-0.081888
60	6	0	6.971209	3.867782	-0.930435
61	6	0	4.724966	-1.560649	-0.553464
62	1	0	3.838559	-0.871749	1.234734
63	6	0	4.644458	-2.457912	-1.536297
64	6	0	6.079120	-1.062575	-0.111856
65	1	0	5.538742	-2.818089	-2.031431
66	1	0	3.709673	-2.871342	-1.890904
67	1	0	6.879656	-1.598564	-0.622948
68	1	0	6.212950	0.003017	-0.313933
69	1	0	6.213531	-1.199819	0.966286
70	1	0	5.224042	1.175511	1.190068
71	1	0	4.488177	2.496658	2.058016
72	1	0	2.644811	1.131241	1.723717
73	1	0	2.496115	2.361854	0.489970
74	8	0	2.203531	-4.299998	1.054291
75	1	0	3.315157	-2.944108	2.025853
76	6	0	0.792252	-1.458260	2.938490
77	1	0	0.482263	-0.828010	3.772426
78	1	0	-0.012745	-2.156387	2.699460
79	1	0	1.675951	-2.027650	3.245945
80	6	0	-4.912189	-2.577234	-1.118558
81	1	0	-5.951264	-2.415765	-0.825072
82	1	0	-4.889379	-2.651674	-2.208834
83	1	0	-4.620315	-3.545225	-0.711332
84	1	0	6.836796	4.863797	-0.509359
85	1	0	8.025770	3.607076	-0.970092
86	1	0	6.529848	3.839097	-1.926289

33

Center Number	Atomic Number	Atomic Type	Coordinates (Angstroms) X	Y	Z
1	6	0	-0.772208	0.226223	-1.109993
2	6	0	0.614874	0.862206	-0.894629
3	1	0	-0.757931	-0.236167	-2.097634
4	1	0	-1.521164	1.021440	-1.149811
5	1	0	0.628056	1.497747	-0.007912
6	1	0	0.822820	1.504253	-1.754451
7	6	0	3.801185	-1.130935	0.042706
8	6	0	2.632880	-2.103112	0.311824
9	6	0	1.406508	-1.139265	0.437250
10	6	0	1.668183	-0.237085	-0.786357
11	6	0	2.946932	-3.039887	1.458511
12	1	0	2.420628	-2.737058	-0.551046
13	8	0	1.520286	-0.278869	1.588666
14	6	0	-1.830027	-3.168183	0.320801
15	6	0	-2.263048	-1.916964	-0.504875
16	6	0	-1.173956	-0.821507	-0.036934
17	6	0	0.021185	-1.727842	0.326964
18	6	0	-0.357064	-2.985706	0.532707
19	1	0	-2.348337	-3.212492	1.287512
20	1	0	-2.067474	-4.102667	-0.194761
21	6	0	-2.086334	-2.280786	-1.993117
22	6	0	-1.634286	-0.066536	1.232640
23	1	0	0.299381	-3.792122	0.832964
24	1	0	-2.357950	-1.467232	-2.666275
25	1	0	-1.052816	-2.558806	-2.207963
26	1	0	-2.709548	-3.139356	-2.249189
27	1	0	-0.834338	0.575473	1.598199
28	1	0	-2.503208	0.563417	1.038051
29	1	0	-1.896260	-0.754387	2.038943
30	6	0	-3.753789	-1.550186	-0.208214
31	6	0	-4.265523	-0.348787	-1.033156
32	6	0	-5.544345	0.293525	-0.520362
33	6	0	-6.050885	1.481699	-1.351967
34	6	0	-6.702043	2.400868	-0.311222
35	6	0	-5.977549	2.019634	0.977804
36	8	0	-5.313333	0.845882	0.807557
37	1	0	-6.333706	-0.454408	-0.407302
38	1	0	-6.737804	1.162834	-2.137264
39	1	0	-5.204489	1.981888	-1.829612
40	1	0	-6.499647	3.454930	-0.506044
41	6	0	-8.216713	2.209271	-0.145427
42	1	0	-3.804782	-1.263619	0.843868
43	1	0	-4.455863	-0.660968	-2.066202
44	1	0	-3.508422	0.435332	-1.082655
45	8	0	-5.969765	2.614351	2.018061
46	1	0	-8.588705	2.804905	0.689168
47	1	0	-8.737724	2.522140	-1.052566
48	1	0	-8.470693	1.163595	0.046052
49	1	0	1.500889	-0.898525	-1.645128
50	6	0	3.199685	0.077821	-0.813342
51	6	0	3.676026	0.057045	-2.273914

52	6	0	3.616319	1.402632	-0.115251
53	1	0	4.728199	0.332137	-2.373208
54	1	0	3.554091	-0.934737	-2.711774
55	1	0	3.094766	0.757858	-2.878365
56	6	0	3.406594	2.714747	-0.899957
57	6	0	3.889449	3.895575	-0.091784
58	8	0	3.188948	4.633793	0.555747
59	8	0	5.235132	4.023475	-0.157264
60	6	0	5.796234	5.099180	0.615301
61	6	0	5.076167	-1.772620	-0.472964
62	1	0	4.052815	-0.698807	1.017729
63	6	0	5.115486	-2.965677	-1.066310
64	6	0	6.349735	-0.997949	-0.237312
65	1	0	6.055376	-3.387233	-1.403719
66	1	0	4.234791	-3.571207	-1.236306
67	1	0	7.219097	-1.542689	-0.607141
68	1	0	6.330835	-0.021526	-0.728539
69	1	0	6.494447	-0.806707	0.831106
70	1	0	2.355626	2.890329	-1.114551
71	1	0	3.959480	2.688885	-1.840146
72	1	0	4.682583	1.334868	0.114718
73	1	0	3.094202	1.468981	0.840411
74	8	0	2.519970	-4.163546	1.569087
75	1	0	3.651326	-2.642407	2.218767
76	6	0	1.295034	-0.846175	2.871611
77	1	0	0.465394	-1.556327	2.867368
78	1	0	2.190432	-1.348181	3.253611
79	1	0	1.053876	-0.019292	3.539853
80	6	0	-4.703058	-2.750357	-0.384144
81	1	0	-5.715032	-2.495174	-0.063277
82	1	0	-4.764534	-3.074992	-1.425904
83	1	0	-4.391882	-3.605981	0.215157
84	1	0	6.869482	5.048489	0.449660
85	1	0	5.400369	6.056522	0.277221
86	1	0	5.563540	4.971169	1.672216

34

Center Number	Atomic Number	Atomic Type	Coordinates (Angstroms) X	Y	Z
1	6	0	-1.010514	0.536273	-0.813797
2	6	0	0.339164	1.106279	-0.338798
3	1	0	-0.912728	0.328575	-1.879827
4	1	0	-1.777012	1.309992	-0.717664
5	1	0	0.269457	1.489255	0.680020
6	1	0	0.606321	1.952579	-0.978416
7	6	0	3.518709	-1.038658	0.240209
8	6	0	2.336753	-2.049032	0.262673
9	6	0	1.086731	-1.150869	0.530426
10	6	0	1.401397	0.015297	-0.427735
11	6	0	2.615649	-3.198841	1.202809
12	1	0	2.178198	-2.493154	-0.721072
13	8	0	1.098163	-0.596991	1.861743
14	6	0	-2.081685	-3.115237	-0.311236
15	6	0	-2.481009	-1.708073	-0.855546
16	6	0	-1.461193	-0.745296	-0.057427
17	6	0	-0.272874	-1.704802	0.178996
18	6	0	-0.634266	-2.978019	0.054365
19	1	0	-2.670802	-3.388548	0.573927
20	1	0	-2.255632	-3.904816	-1.047257
21	6	0	-2.178654	-1.710980	-2.367250
22	6	0	-2.034641	-0.321074	1.315516
23	1	0	0.017128	-3.829135	0.208088
24	1	0	-2.421933	-0.765599	-2.852728
25	1	0	-1.123732	-1.919901	-2.553959
26	1	0	-2.754825	-2.492320	-2.865907
27	1	0	-1.283064	0.222594	1.885845
28	1	0	-2.902811	0.329969	1.207844
29	1	0	-2.339389	-1.182715	1.912341
30	6	0	-3.999975	-1.432717	-0.603575
31	6	0	-4.481761	-0.077017	-1.164414
32	6	0	-5.830143	0.401082	-0.630533
33	6	0	-6.259347	1.785545	-1.151355
34	6	0	-5.788095	2.750201	-0.058512
35	6	0	-5.745001	1.863140	1.182490
36	8	0	-5.772410	0.554899	0.817515
37	1	0	-6.601728	-0.346204	-0.810923
38	1	0	-7.347617	1.831909	-1.241017
39	1	0	-5.834804	2.001139	-2.132221
40	1	0	-4.747508	3.043307	-0.242386
41	6	0	-6.623846	4.010147	0.141941
42	1	0	-4.140488	-1.404668	0.478439
43	1	0	-4.576131	-0.140510	-2.253760
44	1	0	-3.744455	0.703516	-0.966270
45	8	0	-5.683359	2.205737	2.329379
46	1	0	-6.262052	4.575535	1.001322
47	1	0	-6.575040	4.651129	-0.740828
48	1	0	-7.671608	3.758075	0.321937
49	1	0	1.299927	-0.428247	-1.425863
50	6	0	2.917110	0.348728	-0.280328
51	6	0	3.468267	0.812073	-1.638571

52	6	0	3.147883	1.464856	0.772596
53	1	0	4.557019	0.894230	-1.634625
54	1	0	3.194112	0.127819	-2.442683
55	1	0	3.064724	1.794740	-1.889981
56	6	0	4.597529	1.937933	0.959144
57	6	0	5.008379	3.089976	0.066618
58	8	0	4.265219	3.896420	-0.436170
59	8	0	6.351970	3.144222	-0.065939
60	6	0	6.859914	4.251571	-0.830694
61	6	0	4.761027	-1.606207	-0.431864
62	1	0	3.793862	-0.866537	1.283683
63	6	0	5.948986	-1.527795	0.169418
64	6	0	4.628098	-2.338754	-1.746048
65	1	0	6.843664	-1.936746	-0.286052
66	1	0	6.070974	-1.060427	1.139840
67	1	0	5.610508	-2.559397	-2.164868
68	1	0	4.107218	-3.291549	-1.610739
69	1	0	4.063482	-1.772487	-2.488119
70	1	0	5.326202	1.135572	0.848259
71	1	0	4.723786	2.315771	1.980340
72	1	0	2.765566	1.113622	1.729890
73	1	0	2.552526	2.338040	0.498762
74	8	0	2.288432	-4.344198	1.002838
75	1	0	3.198783	-2.943906	2.111353
76	6	0	0.767657	-1.451228	2.948615
77	1	0	1.640839	-2.010257	3.300407
78	1	0	0.422061	-0.808181	3.758582
79	1	0	-0.025169	-2.156541	2.691432
80	6	0	-4.900624	-2.564787	-1.133900
81	1	0	-5.940167	-2.402719	-0.842467
82	1	0	-4.874688	-2.631773	-2.224591
83	1	0	-4.612438	-3.536278	-0.732403
84	1	0	7.940570	4.133760	-0.830222
85	1	0	6.470844	4.222003	-1.848108
86	1	0	6.576183	5.196483	-0.367676

35

Center Number	Atomic Number	Atomic Type	Coordinates (Angstroms) X	Y	Z
1	6	0	-0.801400	0.042419	-1.213639
2	6	0	0.576373	0.734845	-1.203739
3	1	0	-0.842868	-0.561707	-2.120592
4	1	0	-1.579313	0.804123	-1.310579
5	1	0	0.627198	1.503874	-0.432316
6	1	0	0.716058	1.238746	-2.164313
7	6	0	3.887611	-0.930966	-0.163509
8	6	0	2.798989	-1.896112	0.360303
9	6	0	1.525573	-0.986315	0.391869
10	6	0	1.654453	-0.322612	-0.993084
11	6	0	3.227439	-2.588603	1.635887
12	1	0	2.581237	-2.687802	-0.359660
13	8	0	1.654621	0.067621	1.367234
14	6	0	-1.621254	-3.111689	0.821325
15	6	0	-2.144441	-2.042310	-0.186725
16	6	0	-1.088771	-0.841647	0.034079
17	6	0	0.164622	-1.632496	0.474938
18	6	0	-0.148353	-2.846872	0.915871
19	1	0	-2.090359	-2.999941	1.807654
20	1	0	-1.844412	-4.129884	0.491677
21	6	0	-2.008789	-2.651750	-1.596800
22	6	0	-1.521384	0.088746	1.192165
23	1	0	0.557062	-3.565464	1.313069
24	1	0	-2.349532	-1.981601	-2.386350
25	1	0	-0.971150	-2.914994	-1.809647
26	1	0	-2.595714	-3.569019	-1.670751
27	1	0	-0.741957	0.821705	1.397065
28	1	0	-2.434810	0.634262	0.953010
29	1	0	-1.697239	-0.468898	2.114192
30	6	0	-3.637583	-1.685834	0.110716
31	6	0	-4.241290	-0.685175	-0.899056
32	6	0	-5.505165	0.021042	-0.436482
33	6	0	-6.131023	0.967910	-1.471348
34	6	0	-6.741101	2.085836	-0.617073
35	6	0	-5.895547	2.039314	0.653549
36	8	0	-5.196033	0.875031	0.703248
37	1	0	-6.246413	-0.701423	-0.084597
38	1	0	-6.861013	0.455160	-2.099384
39	1	0	-5.349340	1.366603	-2.123003
40	1	0	-6.609086	3.070329	-1.067929
41	6	0	-8.225477	1.892451	-0.273739
42	1	0	-3.659945	-1.209401	1.092795
43	1	0	-4.485410	-1.203795	-1.832968
44	1	0	-3.517414	0.089135	-1.157521
45	8	0	-5.826838	2.864281	1.520141
46	1	0	-8.557800	2.655228	0.431592
47	1	0	-8.834275	1.968529	-1.176945
48	1	0	-8.409537	0.913363	0.175814
49	1	0	1.403019	-1.133934	-1.685435
50	6	0	3.172554	-0.029683	-1.259544
51	6	0	3.486057	-0.514139	-2.689145

52	6	0	3.570242	1.467921	-1.216403
53	1	0	4.528415	-0.342948	-2.963842
54	1	0	3.285632	-1.580626	-2.806253
55	1	0	2.863679	0.025100	-3.409105
56	6	0	3.551992	2.207707	0.121102
57	6	0	3.729235	3.699898	-0.045130
58	8	0	3.637030	4.318307	-1.077005
59	8	0	3.991828	4.287984	1.144232
60	6	0	4.131189	5.719031	1.114879
61	6	0	5.198668	-1.581534	-0.560849
62	1	0	4.119243	-0.271136	0.678382
63	6	0	5.328860	-2.880385	-0.831155
64	6	0	6.399602	-0.669055	-0.595701
65	1	0	6.293072	-3.298507	-1.096489
66	1	0	4.501052	-3.576819	-0.798766
67	1	0	7.304969	-1.212711	-0.867428
68	1	0	6.268231	0.147154	-1.311040
69	1	0	6.561466	-0.203594	0.382255
70	1	0	4.332683	1.859662	0.801014
71	1	0	2.614485	2.048863	0.657563
72	1	0	2.921002	2.006186	-1.910104
73	1	0	4.572227	1.567714	-1.642978
74	8	0	2.845136	-3.677395	1.990969
75	1	0	3.970815	-2.039161	2.249997
76	6	0	1.533409	-0.274728	2.740964
77	1	0	0.728157	-0.991093	2.915928
78	1	0	2.466961	-0.685204	3.139671
79	1	0	1.310137	0.650025	3.273539
80	6	0	-4.535742	-2.933336	0.209284
81	1	0	-5.551042	-2.657585	0.502114
82	1	0	-4.607270	-3.463910	-0.743511
83	1	0	-4.175863	-3.638165	0.958994
84	1	0	4.953917	6.008730	0.461610
85	1	0	3.212292	6.184342	0.758718
86	1	0	4.335511	6.012585	2.141452

36

Center Number	Atomic Number	Atomic Type	Coordinates (Angstroms) X	Y	Z
1	6	0	-1.002358	0.148951	-1.160396
2	6	0	0.393807	0.795248	-1.089155
3	1	0	-1.031646	-0.447616	-2.073008
4	1	0	-1.752376	0.935294	-1.277850
5	1	0	0.446778	1.539170	-0.293788
6	1	0	0.581149	1.322065	-2.029436
7	6	0	3.625085	-1.171158	-0.186898
8	6	0	2.463677	-1.965587	0.460246
9	6	0	1.244518	-0.991930	0.490028
10	6	0	1.438404	-0.293793	-0.871676
11	6	0	2.866155	-2.649780	1.746290
12	1	0	2.149748	-2.786770	-0.193578
13	8	0	1.393341	0.029394	1.494977
14	6	0	-2.012822	-2.984310	0.814840
15	6	0	-2.463844	-1.878837	-0.189021
16	6	0	-1.358873	-0.732045	0.069525
17	6	0	-0.149049	-1.580181	0.525438
18	6	0	-0.532353	-2.783213	0.941203
19	1	0	-2.497945	-2.862671	1.792030
20	1	0	-2.273891	-3.987004	0.466137
21	6	0	-2.328402	-2.475470	-1.604551
22	6	0	-1.768374	0.202729	1.232671
23	1	0	0.119652	-3.547158	1.346443
24	1	0	-2.624567	-1.780192	-2.390208
25	1	0	-1.299685	-2.781228	-1.803637
26	1	0	-2.954158	-3.364428	-1.702302
27	1	0	-0.954871	0.887432	1.467981
28	1	0	-2.644610	0.801116	0.980885
29	1	0	-1.997927	-0.357935	2.140977
30	6	0	-3.944226	-1.457860	0.086125
31	6	0	-4.477275	-0.404274	-0.909016
32	6	0	-5.737616	0.323326	-0.469917
33	6	0	-6.279237	1.344951	-1.480892
34	6	0	-6.897194	2.435098	-0.596743
35	6	0	-6.119016	2.291904	0.709370
36	8	0	-5.457215	1.104091	0.727374
37	1	0	-6.520030	-0.388900	-0.194818
38	1	0	-6.991620	0.891850	-2.171858
39	1	0	-5.452957	1.751573	-2.069536
40	1	0	-6.712226	3.437388	-0.985397
41	6	0	-8.402153	2.273187	-0.337215
42	1	0	-3.963845	-1.004154	1.078925
43	1	0	-4.699829	-0.880047	-1.870746
44	1	0	-3.720879	0.355857	-1.109674
45	8	0	-6.070880	3.064676	1.623722
46	1	0	-8.746565	3.010046	0.389511
47	1	0	-8.961915	2.414160	-1.264018
48	1	0	-8.637933	1.279385	0.051902
49	1	0	1.222116	-1.080459	-1.606384
50	6	0	2.958583	0.006567	-1.036845
51	6	0	3.308190	-0.025450	-2.533226

52	6	0	3.316130	1.399809	-0.456080
53	1	0	4.376866	0.095491	-2.714967
54	1	0	3.012660	-0.966435	-2.997598
55	1	0	2.791810	0.786141	-3.053782
56	6	0	4.789896	1.800609	-0.522777
57	6	0	5.031088	3.210105	-0.027103
58	8	0	4.187193	4.047599	0.172597
59	8	0	6.352529	3.436404	0.153356
60	6	0	6.703229	4.760430	0.591617
61	6	0	4.631422	-2.064355	-0.904562
62	1	0	4.165966	-0.706197	0.638579
63	6	0	4.289755	-3.116132	-1.647592
64	6	0	6.081452	-1.701471	-0.720204
65	1	0	5.042692	-3.720483	-2.139963
66	1	0	3.261628	-3.421090	-1.804756
67	1	0	6.739358	-2.407431	-1.228432
68	1	0	6.296761	-0.701429	-1.108074
69	1	0	6.336611	-1.692627	0.343774
70	1	0	5.178051	1.767112	-1.545878
71	1	0	5.428486	1.134231	0.060507
72	1	0	2.987227	1.449341	0.580646
73	1	0	2.739447	2.157064	-0.991824
74	8	0	3.964916	-2.590656	2.242586
75	1	0	2.083518	-3.273000	2.223911
76	6	0	1.235373	-0.336032	2.859437
77	1	0	0.376711	-0.994932	3.011172
78	1	0	2.134109	-0.813564	3.258522
79	1	0	1.067863	0.591826	3.406664
80	6	0	-4.901714	-2.663660	0.135509
81	1	0	-5.904100	-2.350744	0.434180
82	1	0	-4.992086	-3.156208	-0.835927
83	1	0	-4.580151	-3.411185	0.860871
84	1	0	7.786647	4.759900	0.681020
85	1	0	6.378409	5.502371	-0.137683
86	1	0	6.238924	4.978753	1.552995

37

Center Number	Atomic Number	Atomic Type	Coordinates (Angstroms) X	Y	Z
1	6	0	-1.074231	0.190309	-1.137446
2	6	0	0.320540	0.836045	-1.027080
3	1	0	-1.099278	-0.356093	-2.080809
4	1	0	-1.825779	0.980387	-1.216603
5	1	0	0.369257	1.536053	-0.192215
6	1	0	0.509344	1.414322	-1.936690
7	6	0	3.548218	-1.124894	-0.137927
8	6	0	2.378067	-2.025110	0.350650
9	6	0	1.167646	-1.039895	0.446905
10	6	0	1.367890	-0.261001	-0.869252
11	6	0	2.769748	-2.811698	1.580115
12	1	0	2.102570	-2.761439	-0.405516
13	8	0	1.335857	-0.084241	1.511892
14	6	0	-2.073204	-3.055025	0.652386
15	6	0	-2.532350	-1.892889	-0.283301
16	6	0	-1.432092	-0.758195	0.040601
17	6	0	-0.222651	-1.628294	0.448929
18	6	0	-0.593744	-2.856791	0.795425
19	1	0	-2.560903	-2.997873	1.634336
20	1	0	-2.326829	-4.036710	0.243643
21	6	0	-2.397823	-2.405235	-1.731397
22	6	0	-1.845460	0.110014	1.252612
23	1	0	0.072663	-3.631559	1.153180
24	1	0	-2.690659	-1.664687	-2.476113
25	1	0	-1.370605	-2.705246	-1.946705
26	1	0	-3.027967	-3.283895	-1.879606
27	1	0	-1.031682	0.777987	1.531086
28	1	0	-2.717032	0.727235	1.031123
29	1	0	-2.084133	-0.498822	2.126484
30	6	0	-4.014420	-1.496437	0.019680
31	6	0	-4.552116	-0.378009	-0.898579
32	6	0	-5.861969	0.262624	-0.443519
33	6	0	-6.313101	1.456773	-1.305478
34	6	0	-5.778033	2.677291	-0.549842
35	6	0	-5.669038	2.168550	0.884661
36	8	0	-5.716926	0.810874	0.898440
37	1	0	-6.647785	-0.487560	-0.365352
38	1	0	-7.404448	1.497864	-1.348061
39	1	0	-5.942552	1.384027	-2.328339
40	1	0	-4.748006	2.887528	-0.861536
41	6	0	-6.595612	3.959690	-0.663913
42	1	0	-4.037103	-1.113590	1.041440
43	1	0	-4.728282	-0.776085	-1.903815
44	1	0	-3.808615	0.413754	-1.010660
45	8	0	-5.545237	2.814274	1.886630
46	1	0	-6.184834	4.732777	-0.013769
47	1	0	-6.591470	4.331243	-1.690763
48	1	0	-7.633366	3.788064	-0.368308
49	1	0	1.153990	-1.001383	-1.649979
50	6	0	2.889490	0.047859	-1.002418
51	6	0	3.285076	0.040568	-2.489055

52	6	0	3.233666	1.435266	-0.400926
53	1	0	4.367038	0.049015	-2.626777
54	1	0	2.893965	-0.832272	-3.013313
55	1	0	2.879069	0.924862	-2.988261
56	6	0	4.704833	1.847822	-0.469605
57	6	0	4.938714	3.245833	0.059312
58	8	0	4.094553	4.089287	0.231955
59	8	0	6.252539	3.455991	0.302842
60	6	0	6.597140	4.773623	0.765425
61	6	0	4.690104	-1.924726	-0.748319
62	1	0	3.956190	-0.643377	0.754103
63	6	0	5.950284	-1.703025	-0.373171
64	6	0	4.377465	-3.028766	-1.730778
65	1	0	6.774472	-2.269622	-0.791404
66	1	0	6.205702	-0.955662	0.369087
67	1	0	5.289348	-3.392708	-2.205339
68	1	0	3.907162	-3.878812	-1.226937
69	1	0	3.691495	-2.708174	-2.516231
70	1	0	5.079291	1.844504	-1.498246
71	1	0	5.356297	1.168162	0.081070
72	1	0	2.904732	1.465371	0.637340
73	1	0	2.649581	2.194950	-0.924772
74	8	0	2.418340	-3.943692	1.812323
75	1	0	3.462676	-2.295973	2.276250
76	6	0	1.122876	-0.517325	2.848353
77	1	0	0.948562	0.381612	3.439946
78	1	0	0.256568	-1.176033	2.935588
79	1	0	2.000721	-1.031262	3.253061
80	6	0	-4.968303	-2.705957	-0.013481
81	1	0	-5.961863	-2.425812	0.341131
82	1	0	-5.083580	-3.111097	-1.022127
83	1	0	-4.623565	-3.513161	0.632664
84	1	0	7.675660	4.761492	0.901050
85	1	0	6.311244	5.523532	0.028112
86	1	0	6.094089	4.990253	1.707500

38

Center Number	Atomic Number	Atomic Type	Coordinates (Angstroms) X	Y	Z
1	6	0	-0.982850	0.081065	-1.189414
2	6	0	0.403810	0.751187	-1.176254
3	1	0	-1.017876	-0.560091	-2.070803
4	1	0	-1.747547	0.848902	-1.332046
5	1	0	0.457210	1.534501	-0.419803
6	1	0	0.566775	1.231849	-2.145228
7	6	0	3.682659	-1.111706	-0.221563
8	6	0	2.544015	-1.887425	0.486106
9	6	0	1.308436	-0.935905	0.479815
10	6	0	1.468973	-0.309701	-0.920347
11	6	0	2.974329	-2.493664	1.801948
12	1	0	2.237658	-2.748959	-0.117669
13	8	0	1.451934	0.143117	1.424997
14	6	0	-1.904767	-2.970070	0.950079
15	6	0	-2.392549	-1.921939	-0.096625
16	6	0	-1.305006	-0.743953	0.088208
17	6	0	-0.073241	-1.545978	0.567395
18	6	0	-0.427100	-2.735583	1.043919
19	1	0	-2.377035	-2.812947	1.928341
20	1	0	-2.151228	-3.992646	0.652129
21	6	0	-2.268168	-2.582350	-1.484703
22	6	0	-1.716997	0.239989	1.209252
23	1	0	0.244882	-3.468234	1.473862
24	1	0	-2.587052	-1.928714	-2.296716
25	1	0	-1.237398	-2.880393	-1.685340
26	1	0	-2.879791	-3.485227	-1.531957
27	1	0	-0.916514	0.954530	1.396166
28	1	0	-2.611186	0.804289	0.942530
29	1	0	-1.918499	-0.279145	2.148463
30	6	0	-3.876167	-1.515243	0.182570
31	6	0	-4.449509	-0.532510	-0.861547
32	6	0	-5.706667	0.206417	-0.432749
33	6	0	-6.295102	1.150175	-1.492179
34	6	0	-6.901233	2.289636	-0.663881
35	6	0	-6.079796	2.245868	0.622552
36	8	0	-5.400952	1.070134	0.699864
37	1	0	-6.468909	-0.496031	-0.085236
38	1	0	-7.022146	0.642205	-2.127451
39	1	0	-5.494058	1.526247	-2.133721
40	1	0	-6.744355	3.265595	-1.125141
41	6	0	-8.394597	2.125529	-0.345304
42	1	0	-3.887362	-1.004713	1.147521
43	1	0	-4.691470	-1.071194	-1.784535
44	1	0	-3.708503	0.221826	-1.130189
45	8	0	-6.012741	3.080165	1.479919
46	1	0	-8.726872	2.903429	0.343289
47	1	0	-8.985417	2.198974	-1.260553
48	1	0	-8.603028	1.155959	0.114140
49	1	0	1.252353	-1.136865	-1.608707
50	6	0	2.982169	0.001397	-1.128408
51	6	0	3.309447	-0.109716	-2.625490

52	6	0	3.327754	1.430357	-0.632349
53	1	0	4.374288	0.007030	-2.829788
54	1	0	3.013230	-1.077040	-3.031456
55	1	0	2.780525	0.668996	-3.182257
56	6	0	4.802724	1.855292	-0.769345
57	6	0	4.966399	3.294820	-0.342896
58	8	0	4.643427	4.252124	-1.003464
59	8	0	5.499985	3.397197	0.894111
60	6	0	5.651174	4.736343	1.397418
61	6	0	4.694757	-2.029233	-0.899822
62	1	0	4.226548	-0.587716	0.565564
63	6	0	4.358859	-3.120114	-1.586812
64	6	0	6.141861	-1.644508	-0.740292
65	1	0	5.114447	-3.741838	-2.052681
66	1	0	3.332454	-3.441045	-1.722541
67	1	0	6.804062	-2.367385	-1.218258
68	1	0	6.345503	-0.661631	-1.175526
69	1	0	6.401304	-1.583001	0.320859
70	1	0	5.125944	1.797498	-1.809477
71	1	0	5.451725	1.225438	-0.162037
72	1	0	3.030315	1.522357	0.411942
73	1	0	2.726707	2.146232	-1.198824
74	8	0	4.080981	-2.398243	2.274066
75	1	0	2.204926	-3.093648	2.328283
76	6	0	1.329445	-0.152737	2.810107
77	1	0	0.499160	-0.834327	3.011743
78	1	0	2.252451	-0.573350	3.217686
79	1	0	1.133991	0.796072	3.310285
80	6	0	-4.807321	-2.734779	0.320113
81	1	0	-5.813950	-2.423252	0.606032
82	1	0	-4.895067	-3.292265	-0.615848
83	1	0	-4.465345	-3.426161	1.090555
84	1	0	6.085178	4.627666	2.388123
85	1	0	6.310625	5.313468	0.749677
86	1	0	4.682841	5.233023	1.453644

39

Center Number	Atomic Number	Atomic Type	Coordinates (Angstroms) X	Y	Z
1	6	0	-1.022012	0.150986	-1.103718
2	6	0	0.358604	0.821599	-0.971640
3	1	0	-1.037915	-0.361990	-2.065842
4	1	0	-1.789827	0.927728	-1.152168
5	1	0	0.390691	1.487882	-0.108833
6	1	0	0.531442	1.439381	-1.858317
7	6	0	3.631020	-1.117777	-0.185597
8	6	0	2.479017	-2.045206	0.283652
9	6	0	1.250095	-1.092109	0.426264
10	6	0	1.431067	-0.257243	-0.856585
11	6	0	2.896051	-2.882496	1.473782
12	1	0	2.198910	-2.753291	-0.495881
13	8	0	1.398229	-0.176643	1.528788
14	6	0	-1.952785	-3.174320	0.568074
15	6	0	-2.438851	-1.985930	-0.318848
16	6	0	-1.355863	-0.845236	0.040884
17	6	0	-0.129040	-1.708084	0.409204
18	6	0	-0.476241	-2.955661	0.709556
19	1	0	-2.433326	-3.163970	1.555010
20	1	0	-2.192689	-4.143865	0.123426
21	6	0	-2.305853	-2.440546	-1.786435
22	6	0	-1.778474	-0.029161	1.285462
23	1	0	0.206888	-3.729847	1.035088
24	1	0	-2.611719	-1.675587	-2.500571
25	1	0	-1.276103	-2.719085	-2.017900
26	1	0	-2.925364	-3.321014	-1.965420
27	1	0	-0.972878	0.638059	1.588042
28	1	0	-2.660048	0.582920	1.089793
29	1	0	-2.004762	-0.674579	2.136259
30	6	0	-3.925767	-1.625900	0.005880
31	6	0	-4.494776	-0.501796	-0.887589
32	6	0	-5.777760	0.147677	-0.374112
33	6	0	-6.297112	1.302571	-1.250873
34	6	0	-5.720564	2.556148	-0.585585
35	6	0	-5.506308	2.107922	0.857428
36	8	0	-5.547367	0.752309	0.931831
37	1	0	-6.549360	-0.603711	-0.211248
38	1	0	-7.389064	1.336284	-1.218870
39	1	0	-5.998086	1.188847	-2.293183
40	1	0	-4.717002	2.759351	-0.978009
41	6	0	-6.555387	3.828003	-0.693721
42	1	0	-3.947716	-1.264262	1.035375
43	1	0	-4.716658	-0.900104	-1.883586
44	1	0	-3.752361	0.285743	-1.032268
45	8	0	-5.312667	2.795956	1.819519
46	1	0	-6.108304	4.629016	-0.104108
47	1	0	-6.624752	4.158313	-1.732283
48	1	0	-7.569003	3.661661	-0.321312
49	1	0	1.237976	-0.968470	-1.669015
50	6	0	2.946761	0.096292	-0.973505
51	6	0	3.324281	0.152758	-2.461628

52	6	0	3.232817	1.463630	-0.299725
53	1	0	4.380972	0.369089	-2.622984
54	1	0	3.109535	-0.791998	-2.962820
55	1	0	2.750268	0.937701	-2.962120
56	6	0	4.697777	1.940061	-0.286356
57	6	0	4.808876	3.262072	0.434974
58	8	0	5.048338	3.395351	1.609930
59	8	0	4.575970	4.303441	-0.395271
60	6	0	4.610124	5.605588	0.215507
61	6	0	4.761576	-1.862479	-0.887269
62	1	0	4.066669	-0.684802	0.721639
63	6	0	4.593525	-3.006348	-1.551071
64	6	0	6.141469	-1.263082	-0.773476
65	1	0	5.434143	-3.499386	-2.025334
66	1	0	3.636902	-3.501472	-1.652739
67	1	0	6.890844	-1.912422	-1.227161
68	1	0	6.204881	-0.287636	-1.261663
69	1	0	6.414832	-1.108378	0.275378
70	1	0	5.081792	2.057940	-1.300399
71	1	0	5.329256	1.237402	0.254528
72	1	0	2.871832	1.426494	0.727575
73	1	0	2.641607	2.223920	-0.817381
74	8	0	2.482895	-3.990242	1.716234
75	1	0	3.664880	-2.424714	2.131297
76	6	0	1.247560	-0.686144	2.846745
77	1	0	0.421897	-1.397032	2.921014
78	1	0	2.164960	-1.168018	3.200391
79	1	0	1.041884	0.170579	3.488536
80	6	0	-4.855889	-2.853008	-0.047015
81	1	0	-5.861464	-2.592810	0.289223
82	1	0	-4.945300	-3.256438	-1.058822
83	1	0	-4.508508	-3.655616	0.603416
84	1	0	4.399792	6.309135	-0.586051
85	1	0	3.856270	5.677953	0.999074
86	1	0	5.592270	5.797028	0.646993

40

Center Number	Atomic Number	Atomic Type	Coordinates (Angstroms) X	Y	Z
1	6	0	-0.846125	0.142327	-1.045697
2	6	0	0.517133	0.823684	-0.815774
3	1	0	-0.820130	-0.289836	-2.046780
4	1	0	-1.626523	0.907932	-1.057961
5	1	0	0.510608	1.422986	0.096198
6	1	0	0.696929	1.506302	-1.650512
7	6	0	3.785108	-1.088397	0.018168
8	6	0	2.649331	-2.104968	0.287663
9	6	0	1.394246	-1.183285	0.449210
10	6	0	1.612034	-0.238276	-0.750480
11	6	0	3.006752	-3.042904	1.417500
12	1	0	2.449947	-2.735403	-0.580871
13	8	0	1.482160	-0.351991	1.623647
14	6	0	-1.764054	-3.328629	0.301168
15	6	0	-2.250206	-2.074379	-0.489975
16	6	0	-1.199918	-0.951196	-0.000888
17	6	0	0.031564	-1.820362	0.331052
18	6	0	-0.297158	-3.096535	0.506118
19	1	0	-2.271309	-3.414990	1.270719
20	1	0	-1.971231	-4.258162	-0.235648
21	6	0	-2.068062	-2.393337	-1.987755
22	6	0	-1.678398	-0.250297	1.292749
23	1	0	0.391452	-3.886032	0.778895
24	1	0	-2.373076	-1.573761	-2.638851
25	1	0	-1.025926	-2.626844	-2.213799
26	1	0	-2.659973	-3.267904	-2.263683
27	1	0	-0.903161	0.414771	1.670004
28	1	0	-2.574104	0.348092	1.122040
29	1	0	-1.906826	-0.968524	2.082392
30	6	0	-3.752011	-1.771968	-0.174931
31	6	0	-4.320831	-0.579604	-0.974777
32	6	0	-5.641794	-0.020515	-0.450728
33	6	0	-6.155037	1.213416	-1.216595
34	6	0	-5.642665	2.397577	-0.390646
35	6	0	-5.475053	1.794904	1.001380
36	8	0	-5.483304	0.439004	0.922942
37	1	0	-6.399085	-0.802146	-0.408195
38	1	0	-7.247866	1.218056	-1.230665
39	1	0	-5.808973	1.224324	-2.250511
40	1	0	-4.630276	2.668318	-0.713608
41	6	0	-6.509452	3.652587	-0.392455
42	1	0	-3.806211	-1.505961	0.882262
43	1	0	-4.492848	-0.880363	-2.013897
44	1	0	-3.597499	0.237858	-1.008538
45	8	0	-5.339320	2.374558	2.041622
46	1	0	-6.108846	4.393862	0.299718
47	1	0	-6.548357	4.094161	-1.390427
48	1	0	-7.531300	3.420954	-0.082815
49	1	0	1.456004	-0.881317	-1.625609
50	6	0	3.129449	0.127367	-0.788015
51	6	0	3.589700	0.208041	-2.251552

52	6	0	3.545469	1.420511	-0.035340
53	1	0	4.656823	0.427600	-2.328081
54	1	0	3.398183	-0.722967	-2.786064
55	1	0	3.046780	0.992448	-2.783999
56	6	0	3.232863	2.769172	-0.687906
57	6	0	3.822256	3.928262	0.087436
58	8	0	4.658248	3.852846	0.952867
59	8	0	3.298695	5.102377	-0.332766
60	6	0	3.817398	6.280829	0.308636
61	6	0	5.065443	-1.688992	-0.537679
62	1	0	4.046603	-0.672724	0.993355
63	6	0	6.248428	-1.259016	-0.095844
64	6	0	4.989408	-2.804865	-1.551545
65	1	0	7.178086	-1.660818	-0.482857
66	1	0	6.329468	-0.493737	0.667572
67	1	0	5.982154	-3.047932	-1.931947
68	1	0	4.574835	-3.713919	-1.104889
69	1	0	4.353444	-2.556966	-2.403955
70	1	0	2.164213	2.953771	-0.794730
71	1	0	3.651388	2.836895	-1.696938
72	1	0	4.628369	1.377693	0.107683
73	1	0	3.107700	1.399043	0.963051
74	8	0	2.705028	-4.211988	1.460333
75	1	0	3.625917	-2.605321	2.226624
76	6	0	1.272180	-0.954978	2.893679
77	1	0	0.944100	-0.162870	3.567292
78	1	0	0.505613	-1.732001	2.858170
79	1	0	2.197438	-1.383471	3.291777
80	6	0	-4.654923	-3.005665	-0.364598
81	1	0	-5.670454	-2.799650	-0.021243
82	1	0	-4.720603	-3.308876	-1.412568
83	1	0	-4.300201	-3.861474	0.209499
84	1	0	4.891464	6.363496	0.143440
85	1	0	3.293885	7.115896	-0.149991
86	1	0	3.622865	6.248014	1.380317

41

Center Number	Atomic Number	Atomic Type	Coordinates (Angstroms) X	Y	Z
1	6	0	1.142325	-0.299479	-1.198669
2	6	0	-0.203370	-1.048366	-1.170736
3	1	0	1.154009	0.303343	-2.107778
4	1	0	1.950973	-1.028642	-1.296103
5	1	0	-0.223147	-1.788197	-0.369947
6	1	0	-0.316432	-1.593263	-2.113270
7	6	0	-3.593679	0.661207	-0.335502
8	6	0	-2.501727	1.590879	0.256329
9	6	0	-1.233716	0.684550	0.359969
10	6	0	-1.335336	-0.039740	-0.995823
11	6	0	-2.998818	2.299683	1.498216
12	1	0	-2.225655	2.378944	-0.444458
13	8	0	-1.364997	-0.334291	1.367726
14	6	0	1.860916	2.883353	0.819478
15	6	0	2.427872	1.815738	-0.166046
16	6	0	1.392057	0.597416	0.044965
17	6	0	0.113333	1.363369	0.449087
18	6	0	0.391521	2.589517	0.881630
19	1	0	2.307946	2.788904	1.817771
20	1	0	2.072453	3.903012	0.486291
21	6	0	2.310571	2.409397	-1.584487
22	6	0	1.817059	-0.313504	1.221256
23	1	0	-0.337140	3.298335	1.253901
24	1	0	2.669491	1.733893	-2.361275
25	1	0	1.274287	2.661262	-1.816487
26	1	0	2.891056	3.330948	-1.658261
27	1	0	1.038594	-1.046372	1.429243
28	1	0	2.737358	-0.856382	1.001653
29	1	0	1.976634	0.259709	2.136712
30	6	0	3.921484	1.489871	0.164003
31	6	0	4.569136	0.511797	-0.840928
32	6	0	5.829428	-0.183731	-0.353372
33	6	0	6.509110	-1.087562	-1.392930
34	6	0	7.106711	-2.222375	-0.552519
35	6	0	6.217424	-2.227633	0.688901
36	8	0	5.497523	-1.076833	0.749486
37	1	0	6.543484	0.541609	0.045811
38	1	0	7.252583	-0.543494	-1.977342
39	1	0	5.758653	-1.477500	-2.085417
40	1	0	7.005491	-3.194241	-1.037482
41	6	0	8.575291	-2.018551	-0.152108
42	1	0	3.932672	1.005819	1.142483
43	1	0	4.832367	1.048076	-1.759583
44	1	0	3.864778	-0.268943	-1.132137
45	8	0	6.133147	-3.080343	1.526913
46	1	0	8.895091	-2.798413	0.540225
47	1	0	9.215928	-2.056968	-1.035518
48	1	0	8.728561	-1.051707	0.333984
49	1	0	-1.158357	0.749836	-1.736383
50	6	0	-2.828534	-0.456456	-1.191709
51	6	0	-3.147177	-0.406589	-2.694234

52	6	0	-3.057730	-1.896062	-0.652469
53	1	0	-4.180833	-0.672385	-2.919126
54	1	0	-2.976958	0.589544	-3.104231
55	1	0	-2.505775	-1.108477	-3.235153
56	6	0	-4.445377	-2.538330	-0.796721
57	6	0	-5.393241	-2.332681	0.365455
58	8	0	-5.124038	-1.838131	1.432630
59	8	0	-6.616285	-2.825249	0.071531
60	6	0	-7.592641	-2.751490	1.125547
61	6	0	-4.721916	1.414570	-1.029124
62	1	0	-4.045379	0.136903	0.509628
63	6	0	-4.582638	2.615363	-1.591356
64	6	0	-6.073738	0.746285	-1.019291
65	1	0	-5.425443	3.107516	-2.063040
66	1	0	-3.648139	3.160568	-1.606488
67	1	0	-6.400265	0.556155	0.007608
68	1	0	-6.825784	1.365263	-1.509831
69	1	0	-6.055983	-0.221087	-1.527376
70	1	0	-4.327440	-3.625185	-0.871554
71	1	0	-4.963255	-2.256544	-1.715134
72	1	0	-2.767269	-1.932861	0.395988
73	1	0	-2.360890	-2.543823	-1.190218
74	8	0	-2.643113	3.397850	1.853297
75	1	0	-3.770522	1.755371	2.080747
76	6	0	-1.314629	0.061262	2.730531
77	1	0	-0.552520	0.823205	2.909626
78	1	0	-2.284082	0.433463	3.076473
79	1	0	-1.068138	-0.832398	3.304216
80	6	0	4.790436	2.754992	0.291935
81	1	0	5.808021	2.497145	0.593602
82	1	0	4.862361	3.301023	-0.652115
83	1	0	4.405366	3.440416	1.047072
84	1	0	-7.777366	-1.713857	1.402473
85	1	0	-8.493402	-3.204552	0.719492
86	1	0	-7.248314	-3.299177	2.002392

42

Center Number	Atomic Number	Atomic Type	Coordinates (Angstroms) X	Y	Z
1	6	0	-1.001112	0.511250	-0.792553
2	6	0	0.339569	1.101275	-0.317677
3	1	0	-0.906206	0.319182	-1.861807
4	1	0	-1.782025	1.268320	-0.680477
5	1	0	0.266011	1.468726	0.706641
6	1	0	0.584070	1.962019	-0.947632
7	6	0	3.571825	-0.995711	0.169569
8	6	0	2.405884	-2.018967	0.204996
9	6	0	1.141322	-1.156421	0.507231
10	6	0	1.424507	0.034540	-0.429482
11	6	0	2.727510	-3.190075	1.109394
12	1	0	2.226454	-2.446799	-0.781301
13	8	0	1.152067	-0.631936	1.850952
14	6	0	-2.005458	-3.161804	-0.326349
15	6	0	-2.431674	-1.755852	-0.851539
16	6	0	-1.420185	-0.787649	-0.050384
17	6	0	-0.212456	-1.729691	0.159126
18	6	0	-0.555845	-3.006858	0.023516
19	1	0	-2.579402	-3.452031	0.563359
20	1	0	-2.177119	-3.946728	-1.067866
21	6	0	-2.141290	-1.737121	-2.365714
22	6	0	-1.985726	-0.396080	1.335622
23	1	0	0.111038	-3.848158	0.162279
24	1	0	-2.401859	-0.789932	-2.838547
25	1	0	-1.085217	-1.929159	-2.563531
26	1	0	-2.710613	-2.520618	-2.868862
27	1	0	-1.234547	0.143304	1.910606
28	1	0	-2.862733	0.246509	1.249582
29	1	0	-2.273918	-1.273775	1.917138
30	6	0	-3.952697	-1.504717	-0.585945
31	6	0	-4.461677	-0.158347	-1.145659
32	6	0	-5.801889	0.309618	-0.583271
33	6	0	-6.270174	1.674847	-1.121126
34	6	0	-5.783311	2.671691	-0.064700
35	6	0	-5.684663	1.813265	1.193309
36	8	0	-5.703336	0.497014	0.858712
37	1	0	-6.565714	-0.454108	-0.723326
38	1	0	-7.361258	1.701126	-1.178968
39	1	0	-5.878855	1.875186	-2.118864
40	1	0	-4.754733	2.979720	-0.287609
41	6	0	-6.636307	3.920170	0.135202
42	1	0	-4.084144	-1.476949	0.497171
43	1	0	-4.580174	-0.231753	-2.232047
44	1	0	-3.729154	0.632230	-0.970857
45	8	0	-5.589978	2.183111	2.329503
46	1	0	-6.258905	4.510901	0.970508
47	1	0	-6.627597	4.542375	-0.762215
48	1	0	-7.672911	3.652775	0.353561
49	1	0	1.330981	-0.391483	-1.435748
50	6	0	2.935127	0.402505	-0.280485
51	6	0	3.455178	0.896991	-1.638455

52	6	0	3.107236	1.498290	0.804693
53	1	0	4.519889	1.130140	-1.624604
54	1	0	3.304636	0.146043	-2.415531
55	1	0	2.919611	1.802674	-1.936217
56	6	0	4.529931	1.960936	1.172104
57	6	0	5.240793	2.854353	0.181061
58	8	0	6.349455	2.664292	-0.260189
59	8	0	4.502075	3.938926	-0.135010
60	6	0	5.112497	4.865931	-1.049825
61	6	0	4.792950	-1.499815	-0.594052
62	1	0	3.895138	-0.862273	1.207949
63	6	0	4.721995	-2.373691	-1.598632
64	6	0	6.137829	-0.992509	-0.137087
65	1	0	5.620212	-2.709425	-2.103616
66	1	0	3.791920	-2.790199	-1.962455
67	1	0	6.946349	-1.494097	-0.670080
68	1	0	6.255017	0.082493	-0.294371
69	1	0	6.276687	-1.173464	0.934414
70	1	0	5.192383	1.124040	1.382932
71	1	0	4.452569	2.544234	2.095842
72	1	0	2.640455	1.135414	1.719513
73	1	0	2.540766	2.379781	0.497460
74	8	0	2.308044	-4.312088	0.962408
75	1	0	3.430663	-2.970037	1.940560
76	6	0	0.889114	-1.531628	2.918595
77	1	0	0.587105	-0.922912	3.771222
78	1	0	0.087169	-2.232700	2.677802
79	1	0	1.783557	-2.098248	3.198627
80	6	0	-4.839263	-2.652053	-1.106972
81	1	0	-5.882247	-2.496318	-0.824255
82	1	0	-4.806101	-2.732868	-2.196476
83	1	0	-4.544291	-3.615446	-0.691153
84	1	0	5.332031	4.375802	-1.998065
85	1	0	4.383834	5.660658	-1.188282
86	1	0	6.037325	5.261234	-0.630496

43

Center Number	Atomic Number	Atomic Type	Coordinates (Angstroms) X	Y	Z
1	6	0	-1.057634	0.510668	-0.818268
2	6	0	0.288257	1.095542	-0.352036
3	1	0	-0.962717	0.298965	-1.883682
4	1	0	-1.832224	1.276105	-0.721829
5	1	0	0.218424	1.485361	0.664089
6	1	0	0.545609	1.939345	-0.998528
7	6	0	3.509152	-1.046376	0.088017
8	6	0	2.325514	-2.028382	0.286122
9	6	0	1.063376	-1.146892	0.534664
10	6	0	1.360020	0.013890	-0.436065
11	6	0	2.633244	-3.131264	1.271883
12	1	0	2.115374	-2.560653	-0.648107
13	8	0	1.050236	-0.578454	1.859713
14	6	0	-2.099262	-3.146026	-0.294310
15	6	0	-2.507222	-1.745398	-0.847359
16	6	0	-1.493158	-0.771289	-0.054786
17	6	0	-0.293831	-1.717864	0.185355
18	6	0	-0.652535	-2.992248	0.066553
19	1	0	-2.685142	-3.416339	0.593755
20	1	0	-2.268600	-3.941389	-1.025140
21	6	0	-2.205091	-1.755904	-2.359166
22	6	0	-2.066994	-0.348839	1.318485
23	1	0	-0.004448	-3.846293	0.221321
24	1	0	-2.456546	-0.815806	-2.850284
25	1	0	-1.148478	-1.956071	-2.545914
26	1	0	-2.775137	-2.544996	-2.852652
27	1	0	-1.318217	0.201514	1.885557
28	1	0	-2.940577	0.294609	1.209547
29	1	0	-2.364102	-1.211447	1.917904
30	6	0	-4.028253	-1.479770	-0.596621
31	6	0	-4.520298	-0.131258	-1.165369
32	6	0	-5.875212	0.336298	-0.638301
33	6	0	-6.312271	1.717433	-1.161435
34	6	0	-5.855924	2.685599	-0.065320
35	6	0	-5.812103	1.798526	1.175566
36	8	0	-5.825937	0.489826	0.809683
37	1	0	-6.640051	-0.416776	-0.823236
38	1	0	-7.400253	1.754867	-1.258090
39	1	0	-5.883315	1.936926	-2.139517
40	1	0	-4.816937	2.988067	-0.242911
41	6	0	-6.704042	3.937979	0.130620
42	1	0	-4.168652	-1.446020	0.485215
43	1	0	-4.609962	-0.199889	-2.254774
44	1	0	-3.791114	0.656845	-0.967531
45	8	0	-5.759854	2.140501	2.322908
46	1	0	-6.352069	4.506583	0.991938
47	1	0	-6.656077	4.579389	-0.751858
48	1	0	-7.750474	3.676526	0.304841
49	1	0	1.261951	-0.441008	-1.430502
50	6	0	2.871721	0.366909	-0.304936
51	6	0	3.370712	0.924312	-1.646065

52	6	0	3.103298	1.411965	0.819359
53	1	0	4.448463	1.096688	-1.647380
54	1	0	3.161003	0.238679	-2.467375
55	1	0	2.886123	1.879167	-1.861033
56	6	0	4.548063	1.878428	1.049343
57	6	0	5.006827	3.000479	0.144017
58	8	0	4.314577	3.899859	-0.264536
59	8	0	6.329888	2.910268	-0.121778
60	6	0	6.881221	3.978883	-0.911671
61	6	0	4.590799	-1.606617	-0.830638
62	1	0	3.974676	-0.928141	1.067422
63	6	0	4.331191	-2.274715	-1.953837
64	6	0	6.013901	-1.377467	-0.393698
65	1	0	5.132638	-2.654696	-2.576607
66	1	0	3.325066	-2.467318	-2.308126
67	1	0	6.721754	-1.860653	-1.068447
68	1	0	6.258234	-0.311301	-0.361895
69	1	0	6.169474	-1.772982	0.614522
70	1	0	5.269378	1.063075	1.009564
71	1	0	4.620307	2.290626	2.062537
72	1	0	2.725209	0.998309	1.752146
73	1	0	2.505023	2.300218	0.605079
74	8	0	3.683332	-3.261924	1.852744
75	1	0	1.828825	-3.879606	1.420735
76	6	0	0.776330	-1.437152	2.959138
77	1	0	0.477031	-0.792299	3.785785
78	1	0	-0.038181	-2.133126	2.742926
79	1	0	1.662784	-1.999818	3.264394
80	6	0	-4.920252	-2.622158	-1.119627
81	1	0	-5.960886	-2.465613	-0.829463
82	1	0	-4.893680	-2.696551	-2.209793
83	1	0	-4.625797	-3.589000	-0.711281
84	1	0	6.403845	4.013323	-1.890703
85	1	0	6.737315	4.935969	-0.410859
86	1	0	7.939924	3.752263	-1.009324

44

Center Number	Atomic Number	Atomic Type	Coordinates (Angstroms) X	Y	Z
1	6	0	1.154972	0.196670	1.189353
2	6	0	-0.210662	0.914144	1.215326
3	1	0	1.225963	-0.376826	2.114088
4	1	0	1.949681	0.946394	1.226641
5	1	0	-0.280859	1.661126	0.423466
6	1	0	-0.299569	1.446698	2.167124
7	6	0	-3.593206	-0.712994	0.355059
8	6	0	-2.542939	-1.706388	-0.194581
9	6	0	-1.251952	-0.830715	-0.291279
10	6	0	-1.315394	-0.128547	1.079563
11	6	0	-3.035748	-2.410748	-1.441591
12	1	0	-2.312110	-2.491850	0.528327
13	8	0	-1.375315	0.199433	-1.290270
14	6	0	1.830132	-3.039446	-0.768681
15	6	0	2.414387	-1.949718	0.183069
16	6	0	1.374630	-0.735243	-0.037351
17	6	0	0.089375	-1.512910	-0.401516
18	6	0	0.360105	-2.746969	-0.814235
19	1	0	2.262089	-2.968881	-1.775479
20	1	0	2.045417	-4.051215	-0.415023
21	6	0	2.321406	-2.512531	1.615666
22	6	0	1.777637	0.145563	-1.243775
23	1	0	-0.375673	-3.461465	-1.160768
24	1	0	2.701482	-1.824907	2.371566
25	1	0	1.288555	-2.752933	1.874044
26	1	0	2.897227	-3.436259	1.696063
27	1	0	1.006665	0.889194	-1.440222
28	1	0	2.712506	0.677290	-1.062799
29	1	0	1.902215	-0.445161	-2.153233
30	6	0	3.902214	-1.635740	-0.183787
31	6	0	4.565256	-0.610516	0.762018
32	6	0	5.864535	0.002497	0.245189
33	6	0	6.476245	1.067361	1.174824
34	6	0	5.949169	2.391732	0.613481
35	6	0	5.659277	2.051730	-0.845959
36	8	0	5.624806	0.704156	-1.009574
37	1	0	6.587904	-0.775997	0.006414
38	1	0	7.566660	1.044460	1.104471
39	1	0	6.206463	0.900842	2.218046
40	1	0	4.973654	2.624008	1.057101
41	6	0	6.859589	3.605465	0.769901
42	1	0	3.897220	-1.198224	-1.183818
43	1	0	4.798302	-1.089429	1.719197
44	1	0	3.874037	0.204012	0.987777
45	8	0	5.469250	2.811579	-1.753288
46	1	0	6.438555	4.467734	0.251902
47	1	0	6.985711	3.860854	1.824130
48	1	0	7.847656	3.408419	0.347212
49	1	0	-1.050116	-0.925449	1.783313
50	6	0	-2.817218	0.196055	1.401163
51	6	0	-3.077962	-0.250950	2.853984

52	6	0	-3.184633	1.703029	1.348801
53	1	0	-4.102077	-0.046829	3.171911
54	1	0	-2.902937	-1.320433	2.980814
55	1	0	-2.408109	0.281110	3.536237
56	6	0	-3.131101	2.467787	-0.002200
57	6	0	-4.476345	2.597208	-0.673757
58	8	0	-4.903809	1.883365	-1.551036
59	8	0	-5.185759	3.629025	-0.163764
60	6	0	-6.493415	3.832064	-0.727456
61	6	0	-4.887579	-1.347933	0.832261
62	1	0	-3.862245	-0.066967	-0.485378
63	6	0	-5.010887	-2.641559	1.130891
64	6	0	-6.084736	-0.433042	0.906954
65	1	0	-5.963947	-3.048662	1.448782
66	1	0	-4.191223	-3.345352	1.068772
67	1	0	-6.976428	-0.974200	1.225631
68	1	0	-5.923267	0.389601	1.609795
69	1	0	-6.281034	0.022180	-0.067776
70	1	0	-2.459246	1.974592	-0.699502
71	1	0	-2.763489	3.477509	0.187450
72	1	0	-2.511244	2.213144	2.041951
73	1	0	-4.178771	1.830518	1.784180
74	8	0	-2.678795	-3.509340	-1.794675
75	1	0	-3.797412	-1.861279	-2.030411
76	6	0	-1.361923	-0.190187	-2.658344
77	1	0	-1.036778	0.679818	-3.229504
78	1	0	-0.669815	-1.015036	-2.840699
79	1	0	-2.362136	-0.471533	-3.000645
80	6	0	4.771102	-2.905029	-0.268083
81	1	0	5.769984	-2.666966	-0.638776
82	1	0	4.891888	-3.385809	0.706067
83	1	0	4.352405	-3.640588	-0.954853
84	1	0	-6.420743	4.014480	-1.799207
85	1	0	-7.123590	2.959593	-0.556185
86	1	0	-6.898417	4.702077	-0.216788

45

Center Number	Atomic Number	Atomic Type	Coordinates (Angstroms) X	Y	Z
1	6	0	-0.902839	0.191250	-1.148359
2	6	0	0.479720	0.862054	-1.033465
3	1	0	-0.911437	-0.363154	-2.087520
4	1	0	-1.666760	0.968000	-1.237503
5	1	0	0.509072	1.566163	-0.200984
6	1	0	0.663613	1.438783	-1.945221
7	6	0	3.736079	-1.044979	-0.120670
8	6	0	2.576354	-1.956548	0.372574
9	6	0	1.350663	-0.989331	0.457461
10	6	0	1.545647	-0.214922	-0.861993
11	6	0	2.976004	-2.729307	1.608867
12	1	0	2.313227	-2.702577	-0.378564
13	8	0	1.497678	-0.023183	1.515610
14	6	0	-1.859206	-3.052359	0.668655
15	6	0	-2.331067	-1.908519	-0.282068
16	6	0	-1.251249	-0.752730	0.036188
17	6	0	-0.030257	-1.599627	0.457747
18	6	0	-0.383241	-2.830718	0.814260
19	1	0	-2.350150	-2.989589	1.648671
20	1	0	-2.098468	-4.042323	0.271325
21	6	0	-2.179670	-2.432507	-1.724412
22	6	0	-1.687774	0.121208	1.236145
23	1	0	0.293673	-3.591625	1.181842
24	1	0	-2.482016	-1.704342	-2.477526
25	1	0	-1.145843	-2.715142	-1.931061
26	1	0	-2.791937	-3.324401	-1.868533
27	1	0	-0.886361	0.804825	1.513073
28	1	0	-2.569068	0.719837	1.002576
29	1	0	-1.918733	-0.483701	2.115154
30	6	0	-3.820795	-1.530966	0.005387
31	6	0	-4.374953	-0.450035	-0.948833
32	6	0	-5.625984	0.263669	-0.462896
33	6	0	-6.210156	1.287232	-1.447901
34	6	0	-6.802387	2.369257	-0.536773
35	6	0	-5.975501	2.225226	0.738881
36	8	0	-5.310244	1.039812	0.729181
37	1	0	-6.392098	-0.456862	-0.164414
38	1	0	-6.944070	0.833123	-2.115274
39	1	0	-5.408007	1.701776	-2.063854
40	1	0	-6.638659	3.374587	-0.927176
41	6	0	-8.295647	2.195896	-0.222644
42	1	0	-3.853595	-1.119957	1.016190
43	1	0	-4.616846	-0.900711	-1.917964
44	1	0	-3.624518	0.317243	-1.145517
45	8	0	-5.894959	2.996699	1.652352
46	1	0	-8.617575	2.926033	0.521003
47	1	0	-8.890361	2.338981	-1.127100
48	1	0	-8.510613	1.198212	0.168501
49	1	0	1.351177	-0.962714	-1.640755
50	6	0	3.062926	0.118292	-0.986055
51	6	0	3.468514	0.129188	-2.469229

52	6	0	3.385390	1.505013	-0.369503
53	1	0	4.551189	0.155776	-2.598000
54	1	0	3.093723	-0.745366	-3.002223
55	1	0	3.053534	1.011356	-2.964931
56	6	0	4.855861	1.957547	-0.479074
57	6	0	5.065996	3.243374	0.282507
58	8	0	5.427264	3.321689	1.431302
59	8	0	4.768240	4.324623	-0.473259
60	6	0	4.890208	5.597267	0.185907
61	6	0	4.884446	-1.836299	-0.730133
62	1	0	4.142000	-0.556772	0.768330
63	6	0	6.140782	-1.612615	-0.343278
64	6	0	4.582274	-2.936553	-1.720178
65	1	0	6.969891	-2.174585	-0.758123
66	1	0	6.387206	-0.870207	0.407032
67	1	0	5.498411	-3.293298	-2.192006
68	1	0	4.114211	-3.791844	-1.223112
69	1	0	3.898487	-2.615928	-2.507518
70	1	0	5.137979	2.111546	-1.520946
71	1	0	5.524827	1.218000	-0.042414
72	1	0	3.093235	1.499572	0.680196
73	1	0	2.766737	2.256535	-0.867466
74	8	0	2.631863	-3.861467	1.851203
75	1	0	3.666817	-2.203106	2.298790
76	6	0	1.294109	-0.451487	2.855306
77	1	0	0.436277	-1.120519	2.947976
78	1	0	2.179861	-0.950869	3.260335
79	1	0	1.110563	0.448667	3.442038
80	6	0	-4.753349	-2.757086	0.000035
81	1	0	-5.768261	-2.473639	0.286723
82	1	0	-4.813974	-3.221307	-0.987421
83	1	0	-4.430108	-3.519261	0.709135
84	1	0	4.615468	6.339314	-0.559529
85	1	0	4.219914	5.647466	1.043713
86	1	0	5.914242	5.753313	0.524216

46

Center Number	Atomic Number	Atomic Type	Coordinates (Angstroms) X	Y	Z
1	6	0	-0.960669	0.216038	-1.156768
2	6	0	0.435484	0.859981	-1.054188
3	1	0	-0.982697	-0.352123	-2.087516
4	1	0	-1.708646	1.006875	-1.256630
5	1	0	0.482301	1.578227	-0.234895
6	1	0	0.630339	1.417403	-1.975498
7	6	0	3.653017	-1.092604	-0.110954
8	6	0	2.477562	-1.976597	0.395062
9	6	0	1.270853	-0.984344	0.466331
10	6	0	1.478483	-0.236318	-0.866442
11	6	0	2.863407	-2.736517	1.642853
12	1	0	2.200408	-2.728947	-0.344551
13	8	0	1.440342	-0.006389	1.510471
14	6	0	-1.978923	-2.979841	0.712067
15	6	0	-2.428452	-1.842393	-0.257196
16	6	0	-1.326585	-0.702643	0.042595
17	6	0	-0.121942	-1.566722	0.477657
18	6	0	-0.498873	-2.784617	0.854331
19	1	0	-2.468781	-2.891495	1.690683
20	1	0	-2.237361	-3.971204	0.330536
21	6	0	-2.286947	-2.392408	-1.690812
22	6	0	-1.744473	0.198200	1.229184
23	1	0	0.162833	-3.552774	1.234262
24	1	0	-2.577864	-1.671860	-2.455674
25	1	0	-1.257964	-2.694960	-1.893379
26	1	0	-2.913866	-3.276443	-1.819895
27	1	0	-0.930914	0.872931	1.492476
28	1	0	-2.615802	0.808304	0.988159
29	1	0	-1.984113	-0.388172	2.118365
30	6	0	-3.910349	-1.431901	0.024810
31	6	0	-4.441464	-0.349167	-0.939898
32	6	0	-5.693675	0.376045	-0.474331
33	6	0	-6.240483	1.419858	-1.459526
34	6	0	-6.842345	2.496653	-0.548381
35	6	0	-6.051164	2.323961	0.746320
36	8	0	-5.396966	1.132347	0.735010
37	1	0	-6.476600	-0.337578	-0.204254
38	1	0	-6.963001	0.984020	-2.151101
39	1	0	-5.418214	1.832744	-2.049437
40	1	0	-6.655499	3.505083	-0.919911
41	6	0	-8.345388	2.338669	-0.275594
42	1	0	-3.934910	-1.009591	1.031215
43	1	0	-4.673436	-0.799299	-1.911730
44	1	0	-3.681099	0.410662	-1.126916
45	8	0	-5.988508	3.079578	1.674261
46	1	0	-8.677392	3.063349	0.468962
47	1	0	-8.914401	2.500835	-1.193275
48	1	0	-8.582992	1.338952	0.096884
49	1	0	1.265348	-0.993946	-1.630712
50	6	0	3.001327	0.064528	-1.001109
51	6	0	3.400682	0.026492	-2.486214

52	6	0	3.349672	1.462573	-0.426788
53	1	0	4.482976	0.030685	-2.621583
54	1	0	3.009252	-0.855893	-2.993970
55	1	0	2.997075	0.901285	-3.003744
56	6	0	4.822525	1.867879	-0.503414
57	6	0	5.062847	3.273138	0.002797
58	8	0	4.221811	4.121196	0.167694
59	8	0	6.378613	3.483666	0.236109
60	6	0	6.728361	4.806855	0.678437
61	6	0	4.792260	-1.910729	-0.701526
62	1	0	4.061571	-0.594289	0.771448
63	6	0	6.052424	-1.688020	-0.326825
64	6	0	4.477063	-3.032048	-1.663275
65	1	0	6.875049	-2.266829	-0.731194
66	1	0	6.309227	-0.927276	0.401242
67	1	0	5.388444	-3.409072	-2.128527
68	1	0	4.001774	-3.870192	-1.144407
69	1	0	3.794435	-2.723384	-2.456356
70	1	0	5.195843	1.845695	-1.532196
71	1	0	5.471716	1.194490	0.057677
72	1	0	3.020362	1.514846	0.610435
73	1	0	2.768425	2.213744	-0.965802
74	8	0	2.509477	-3.862702	1.898651
75	1	0	3.554524	-2.206765	2.330192
76	6	0	1.217975	-0.407794	2.855054
77	1	0	1.050469	0.505759	3.425853
78	1	0	0.344726	-1.055669	2.953166
79	1	0	2.088924	-0.921571	3.274713
80	6	0	-4.866743	-2.639516	0.032590
81	1	0	-5.872846	-2.335797	0.328777
82	1	0	-4.946144	-3.106201	-0.952447
83	1	0	-4.550785	-3.405353	0.741004
84	1	0	6.230118	5.037936	1.619634
85	1	0	7.807472	4.794146	0.809294
86	1	0	6.440756	5.547015	-0.068014

47

Center Number	Atomic Number	Atomic Type	Coordinates (Angstroms) X	Y	Z
1	6	0	-0.923725	0.021682	-1.165718
2	6	0	0.440622	0.741014	-1.154559
3	1	0	-0.965304	-0.558945	-2.087746
4	1	0	-1.718436	0.769163	-1.233159
5	1	0	0.485619	1.491678	-0.365009
6	1	0	0.561216	1.271006	-2.103596
7	6	0	3.794520	-0.884867	-0.193851
8	6	0	2.731308	-1.883233	0.332199
9	6	0	1.441352	-0.995236	0.391596
10	6	0	1.540272	-0.301434	-0.981969
11	6	0	3.184592	-2.572374	1.597929
12	1	0	2.525013	-2.674836	-0.391273
13	8	0	1.557055	0.040694	1.386678
14	6	0	-1.650985	-3.203085	0.794205
15	6	0	-2.208790	-2.120103	-0.181115
16	6	0	-1.178893	-0.901336	0.061243
17	6	0	0.096411	-1.673340	0.471781
18	6	0	-0.184135	-2.905487	0.883873
19	1	0	-2.114713	-3.129180	1.786597
20	1	0	-1.851619	-4.217329	0.439200
21	6	0	-2.072912	-2.690417	-1.607231
22	6	0	-1.620520	-0.011882	1.247580
23	1	0	0.540970	-3.618744	1.255235
24	1	0	-2.435768	-2.008641	-2.376668
25	1	0	-1.032120	-2.926346	-1.836316
26	1	0	-2.641355	-3.617599	-1.698352
27	1	0	-0.858412	0.736283	1.461154
28	1	0	-2.549855	0.516403	1.033112
29	1	0	-1.773526	-0.594766	2.157667
30	6	0	-3.707473	-1.808525	0.140961
31	6	0	-4.343693	-0.782024	-0.821588
32	6	0	-5.659167	-0.173291	-0.341210
33	6	0	-6.238664	0.902530	-1.278644
34	6	0	-5.728732	2.219950	-0.685532
35	6	0	-5.488415	1.863627	0.778870
36	8	0	-5.460317	0.513952	0.928460
37	1	0	-6.390462	-0.953751	-0.134945
38	1	0	-7.330815	0.880543	-1.244505
39	1	0	-5.934782	0.747202	-2.314188
40	1	0	-4.738503	2.455160	-1.093509
41	6	0	-6.631463	3.437062	-0.859192
42	1	0	-3.732963	-1.373285	1.141598
43	1	0	-4.546947	-1.257898	-1.787031
44	1	0	-3.647976	0.034685	-1.023886
45	8	0	-5.328140	2.612441	1.700921
46	1	0	-6.226173	4.292967	-0.318615
47	1	0	-6.722004	3.703802	-1.914216
48	1	0	-7.633306	3.237645	-0.471466
49	1	0	1.289603	-1.103504	-1.685531
50	6	0	3.047172	0.014720	-1.266234
51	6	0	3.347221	-0.408895	-2.718459

52	6	0	3.461005	1.505336	-1.185873
53	1	0	4.403969	-0.287962	-2.963852
54	1	0	3.068260	-1.446614	-2.907840
55	1	0	2.773830	0.213365	-3.411266
56	6	0	3.400385	2.235722	0.156500
57	6	0	3.560091	3.731535	0.001443
58	8	0	3.396810	4.363703	-1.013228
59	8	0	3.891029	4.306083	1.180268
60	6	0	4.010377	5.739239	1.162876
61	6	0	5.122132	-1.499255	-0.607579
62	1	0	4.015682	-0.227648	0.648057
63	6	0	6.261556	-0.858553	-0.341945
64	6	0	5.144624	-2.847910	-1.284670
65	1	0	7.222784	-1.263675	-0.637046
66	1	0	6.274979	0.092068	0.179393
67	1	0	6.161240	-3.115748	-1.574728
68	1	0	4.773121	-3.632225	-0.618169
69	1	0	4.520619	-2.875479	-2.180267
70	1	0	4.164691	1.892457	0.856076
71	1	0	2.449311	2.067050	0.665902
72	1	0	2.851902	2.063014	-1.900406
73	1	0	4.485153	1.581543	-1.565579
74	8	0	2.908064	-3.709759	1.897152
75	1	0	3.850514	-1.977916	2.256035
76	6	0	1.433136	-0.322355	2.755702
77	1	0	2.384534	-0.675238	3.166260
78	1	0	1.140783	0.579841	3.293672
79	1	0	0.672595	-1.090353	2.909475
80	6	0	-4.576458	-3.079514	0.196240
81	1	0	-5.585813	-2.843883	0.538866
82	1	0	-4.668390	-3.556758	-0.782707
83	1	0	-4.176868	-3.817203	0.892116
84	1	0	4.272547	6.021727	2.179349
85	1	0	4.788625	6.048307	0.465503
86	1	0	3.066024	6.196887	0.868817

48

Center Number	Atomic Number	Atomic Type	Coordinates (Angstroms) X	Y	Z
1	6	0	-0.846123	0.142324	-1.045702
2	6	0	0.517133	0.823684	-0.815777
3	1	0	-0.820124	-0.289844	-2.046783
4	1	0	-1.626523	0.907927	-1.057971
5	1	0	0.510604	1.422987	0.096194
6	1	0	0.696931	1.506300	-1.650515
7	6	0	3.785110	-1.088398	0.018165
8	6	0	2.649330	-2.104965	0.287666
9	6	0	1.394247	-1.183280	0.449215
10	6	0	1.612035	-0.238274	-0.750478
11	6	0	3.006752	-3.042899	1.417503
12	1	0	2.449942	-2.735401	-0.580867
13	8	0	1.482165	-0.351985	1.623652
14	6	0	-1.764051	-3.328626	0.301181
15	6	0	-2.250202	-2.074381	-0.489969
16	6	0	-1.199918	-0.951195	-0.000889
17	6	0	0.031566	-1.820358	0.331061
18	6	0	-0.297156	-3.096529	0.506134
19	1	0	-2.271309	-3.414983	1.270731
20	1	0	-1.971226	-4.258163	-0.235629
21	6	0	-2.068059	-2.393349	-1.987748
22	6	0	-1.678401	-0.250290	1.292743
23	1	0	0.391454	-3.886024	0.778917
24	1	0	-2.373076	-1.573779	-2.638849
25	1	0	-1.025921	-2.626854	-2.213791
26	1	0	-2.659967	-3.267920	-2.263669
27	1	0	-0.903167	0.414781	1.669996
28	1	0	-2.574108	0.348097	1.122029
29	1	0	-1.906830	-0.968513	2.082390
30	6	0	-3.752008	-1.771968	-0.174925
31	6	0	-4.320830	-0.579609	-0.974777
32	6	0	-5.641794	-0.020518	-0.450730
33	6	0	-6.155037	1.213408	-1.216603
34	6	0	-5.642668	2.397574	-0.390658
35	6	0	-5.475055	1.794907	1.001371
36	8	0	-5.483305	0.439006	0.922937
37	1	0	-6.399084	-0.802150	-0.408195
38	1	0	-7.247865	1.218046	-1.230675
39	1	0	-5.808970	1.224313	-2.250518
40	1	0	-4.630281	2.668316	-0.713621
41	6	0	-6.509459	3.652582	-0.392473
42	1	0	-3.806206	-1.505956	0.882265
43	1	0	-4.492849	-0.880373	-2.013896
44	1	0	-3.597499	0.237854	-1.008544
45	8	0	-5.339334	2.374563	2.041612
46	1	0	-6.108855	4.393863	0.299697
47	1	0	-6.548366	4.094149	-1.390449
48	1	0	-7.531307	3.420949	-0.082832
49	1	0	1.456007	-0.881318	-1.625605
50	6	0	3.129448	0.127370	-0.788013
51	6	0	3.589698	0.208053	-2.251550

52	6	0	3.545468	1.420512	-0.035333
53	1	0	4.656819	0.427622	-2.328078
54	1	0	3.398189	-0.722954	-2.786065
55	1	0	3.046772	0.992458	-2.783995
56	6	0	3.232847	2.769175	-0.687887
57	6	0	3.822258	3.928263	0.087445
58	8	0	4.658264	3.852843	0.952863
59	8	0	3.298694	5.102379	-0.332748
60	6	0	3.817410	6.280831	0.308646
61	6	0	5.065436	-1.688999	-0.537695
62	1	0	4.046617	-0.672727	0.993349
63	6	0	6.248428	-1.259028	-0.095876
64	6	0	4.989383	-2.804874	-1.551557
65	1	0	7.178080	-1.660834	-0.482899
66	1	0	6.329480	-0.493749	0.667538
67	1	0	5.982122	-3.047935	-1.931986
68	1	0	4.574831	-3.713931	-1.104887
69	1	0	4.353393	-2.556986	-2.403950
70	1	0	2.164195	2.953773	-0.794683
71	1	0	3.651345	2.836903	-1.696929
72	1	0	4.628370	1.377699	0.107678
73	1	0	3.107710	1.399035	0.963062
74	8	0	2.705061	-4.211993	1.460318
75	1	0	3.625924	-2.605317	2.226623
76	6	0	1.272173	-0.954968	2.893684
77	1	0	0.944084	-0.162859	3.567292
78	1	0	0.505608	-1.731993	2.858170
79	1	0	2.197427	-1.383457	3.291793
80	6	0	-4.654920	-3.005667	-0.364582
81	1	0	-5.670451	-2.799651	-0.021228
82	1	0	-4.720601	-3.308886	-1.412550
83	1	0	-4.300197	-3.861471	0.209520
84	1	0	4.891476	6.363488	0.143446
85	1	0	3.293903	7.115899	-0.149984
86	1	0	3.622881	6.248024	1.380328

49

Center Number	Atomic Number	Atomic Type	Coordinates (Angstroms) X	Y	Z
1	6	0	-0.970113	-0.053450	-1.185439
2	6	0	0.400815	0.650093	-1.205051
3	1	0	-1.022532	-0.666276	-2.085728
4	1	0	-1.757999	0.698878	-1.275781
5	1	0	0.458473	1.427549	-0.442825
6	1	0	0.522221	1.144238	-2.173064
7	6	0	3.757973	-1.004475	-0.252392
8	6	0	2.686359	-1.924904	0.369492
9	6	0	1.396203	-1.038795	0.397524
10	6	0	1.492087	-0.394129	-0.999275
11	6	0	3.124806	-2.557539	1.670941
12	1	0	2.446657	-2.763504	-0.294458
13	8	0	1.514010	0.032264	1.352551
14	6	0	-1.732768	-3.196266	0.887296
15	6	0	-2.278476	-2.139018	-0.121662
16	6	0	-1.228564	-0.928844	0.074147
17	6	0	0.041514	-1.701593	0.504238
18	6	0	-0.262262	-2.913295	0.958097
19	1	0	-2.190535	-3.082142	1.878443
20	1	0	-1.948609	-4.218816	0.566610
21	6	0	-2.158577	-2.760169	-1.528009
22	6	0	-1.648988	0.004873	1.233709
23	1	0	0.443473	-3.632199	1.355723
24	1	0	-2.515748	-2.099880	-2.318366
25	1	0	-1.122284	-3.017360	-1.754905
26	1	0	-2.740244	-3.681994	-1.584708
27	1	0	-0.875814	0.749129	1.418281
28	1	0	-2.572833	0.537320	1.006766
29	1	0	-1.803865	-0.546232	2.163085
30	6	0	-3.770181	-1.793945	0.198605
31	6	0	-4.396218	-0.790407	-0.793794
32	6	0	-5.710502	-0.161513	-0.336010
33	6	0	-6.266411	0.908909	-1.293606
34	6	0	-5.748773	2.227712	-0.710138
35	6	0	-5.531913	1.886974	0.761573
36	8	0	-5.519293	0.538468	0.927386
37	1	0	-6.452736	-0.931567	-0.129772
38	1	0	-7.359048	0.899534	-1.272616
39	1	0	-5.951753	0.737662	-2.323423
40	1	0	-4.750329	2.445732	-1.107563
41	6	0	-6.634229	3.453222	-0.911152
42	1	0	-3.782050	-1.323776	1.183630
43	1	0	-4.598024	-1.289514	-1.747707
44	1	0	-3.694314	0.016396	-1.013469
45	8	0	-5.376349	2.644630	1.676927
46	1	0	-6.225683	4.310942	-0.375956
47	1	0	-6.707431	3.707641	-1.970536
48	1	0	-7.643483	3.270879	-0.534429
49	1	0	1.238481	-1.219802	-1.674919
50	6	0	2.996914	-0.086155	-1.304603
51	6	0	3.258451	-0.517464	-2.761669

52	6	0	3.401871	1.409443	-1.235388
53	1	0	4.292497	-0.341433	-3.062697
54	1	0	3.049793	-1.577397	-2.914006
55	1	0	2.615117	0.053598	-3.437625
56	6	0	3.401763	2.132979	0.110846
57	6	0	3.635821	3.618659	-0.040131
58	8	0	3.577333	4.249532	-1.067356
59	8	0	3.907172	4.187427	1.156722
60	6	0	4.104132	5.611444	1.139722
61	6	0	5.005697	-1.708529	-0.757690
62	1	0	4.086882	-0.357368	0.561631
63	6	0	5.021036	-2.951096	-1.235516
64	6	0	6.275695	-0.903043	-0.667833
65	1	0	5.941847	-3.403908	-1.584334
66	1	0	4.134683	-3.571127	-1.296629
67	1	0	7.126956	-1.445211	-1.081319
68	1	0	6.188474	0.049361	-1.199665
69	1	0	6.494936	-0.662474	0.377328
70	1	0	4.162053	1.747548	0.793237
71	1	0	2.455294	2.004693	0.638849
72	1	0	2.755653	1.965089	-1.918585
73	1	0	4.403335	1.502628	-1.665803
74	8	0	4.152828	-2.305218	2.249160
75	1	0	2.436856	-3.321648	2.087026
76	6	0	1.412052	-0.275361	2.740002
77	1	0	2.367198	-0.610520	3.148219
78	1	0	1.123830	0.649537	3.240431
79	1	0	0.646695	-1.030661	2.934399
80	6	0	-4.657714	-3.048688	0.304205
81	1	0	-5.658399	-2.786338	0.652371
82	1	0	-4.770337	-3.555333	-0.657714
83	1	0	-4.260778	-3.770603	1.018012
84	1	0	4.306932	5.889552	2.170914
85	1	0	4.946300	5.872407	0.499146
86	1	0	3.209573	6.116179	0.775158

50

Center Number	Atomic Number	Atomic Type	Coordinates (Angstroms) X	Y	Z
1	6	0	-1.093964	0.133266	-1.141612
2	6	0	0.299343	0.784274	-1.060439
3	1	0	-1.121107	-0.447788	-2.064112
4	1	0	-1.848710	0.917074	-1.246812
5	1	0	0.350676	1.515799	-0.253479
6	1	0	0.484971	1.326419	-1.992408
7	6	0	3.543651	-1.178345	-0.187588
8	6	0	2.386153	-1.992683	0.448830
9	6	0	1.159196	-1.023461	0.489861
10	6	0	1.348129	-0.304419	-0.861649
11	6	0	2.775914	-2.686229	1.734323
12	1	0	2.071034	-2.804046	-0.217140
13	8	0	1.301265	-0.016368	1.508351
14	6	0	-2.088665	-3.039454	0.776115
15	6	0	-2.544545	-1.918326	-0.208703
16	6	0	-1.445673	-0.771347	0.072047
17	6	0	-0.231145	-1.620819	0.514437
18	6	0	-0.609720	-2.832174	0.909562
19	1	0	-2.577677	-2.939303	1.753807
20	1	0	-2.341509	-4.037367	0.408367
21	6	0	-2.404205	-2.490662	-1.633657
22	6	0	-1.858120	0.138256	1.253827
23	1	0	0.045225	-3.599375	1.303702
24	1	0	-2.699803	-1.783574	-2.408877
25	1	0	-1.374715	-2.792212	-1.835502
26	1	0	-3.029300	-3.378235	-1.746436
27	1	0	-1.047430	0.821171	1.502743
28	1	0	-2.735198	0.740161	1.013716
29	1	0	-2.088283	-0.439752	2.150610
30	6	0	-4.028001	-1.511881	0.073951
31	6	0	-4.561270	-0.424389	-0.882504
32	6	0	-5.895096	0.200656	-0.477335
33	6	0	-6.328764	1.386341	-1.359796
34	6	0	-5.839782	2.616081	-0.587873
35	6	0	-5.777959	2.112561	0.851202
36	8	0	-5.806822	0.753923	0.866967
37	1	0	-6.673618	-0.559416	-0.426797
38	1	0	-7.418076	1.412343	-1.444494
39	1	0	-5.918135	1.316175	-2.367453
40	1	0	-4.801803	2.840383	-0.861020
41	6	0	-6.670628	3.886400	-0.736651
42	1	0	-4.054565	-1.094786	1.082142
43	1	0	-4.702869	-0.845898	-1.883579
44	1	0	-3.829401	0.378443	-0.990886
45	8	0	-5.701617	2.761792	1.855417
46	1	0	-6.295150	4.667126	-0.074441
47	1	0	-6.633464	4.254875	-1.763916
48	1	0	-7.716114	3.700999	-0.479124
49	1	0	1.130897	-1.080548	-1.607361
50	6	0	2.864921	0.001668	-1.024658
51	6	0	3.221090	0.006772	-2.521288

52	6	0	3.227677	1.385050	-0.423215
53	1	0	4.297120	0.056506	-2.687494
54	1	0	2.854833	-0.883487	-3.034460
55	1	0	2.767380	0.873229	-3.010480
56	6	0	4.702874	1.781636	-0.501205
57	6	0	4.951044	3.186245	0.003224
58	8	0	4.114866	4.039062	0.170077
59	8	0	6.268362	3.390787	0.231989
60	6	0	6.625576	4.710740	0.676863
61	6	0	4.557797	-2.046139	-0.920068
62	1	0	4.083064	-0.718028	0.639659
63	6	0	5.864540	-1.832688	-0.776398
64	6	0	4.073086	-3.187252	-1.784022
65	1	0	6.598011	-2.434767	-1.300941
66	1	0	6.250747	-1.064349	-0.117892
67	1	0	4.882337	-3.570724	-2.406398
68	1	0	3.713900	-4.020114	-1.170460
69	1	0	3.248589	-2.901648	-2.441353
70	1	0	5.079059	1.755381	-1.528790
71	1	0	5.346243	1.106780	0.065603
72	1	0	2.909805	1.417608	0.617774
73	1	0	2.647410	2.151926	-0.941278
74	8	0	3.846330	-2.578922	2.280329
75	1	0	2.008404	-3.362563	2.163648
76	6	0	1.131524	-0.395094	2.869074
77	1	0	2.035234	-0.853648	3.277019
78	1	0	0.933027	0.524767	3.419501
79	1	0	0.286330	-1.074706	3.003401
80	6	0	-4.981929	-2.721909	0.078643
81	1	0	-5.970398	-2.433243	0.440007
82	1	0	-5.110091	-3.148931	-0.919448
83	1	0	-4.629937	-3.515402	0.737834
84	1	0	6.339500	5.454616	-0.066572
85	1	0	6.131222	4.941415	1.620228
86	1	0	7.704973	4.692472	0.804820

51

Center Number	Atomic Number	Atomic Type	Coordinates (Angstroms) X	Y	Z
1	6	0	-0.891294	0.176995	-1.150270
2	6	0	0.492183	0.842147	-1.021087
3	1	0	-0.897847	-0.366196	-2.095895
4	1	0	-1.652299	0.957327	-1.233143
5	1	0	0.518338	1.535668	-0.179784
6	1	0	0.681141	1.430197	-1.924568
7	6	0	3.740221	-1.095286	-0.135874
8	6	0	2.575737	-1.999671	0.347538
9	6	0	1.352744	-1.033266	0.447401
10	6	0	1.555248	-0.239415	-0.858417
11	6	0	2.973074	-2.803374	1.567065
12	1	0	2.299060	-2.729080	-0.413264
13	8	0	1.497463	-0.085809	1.522824
14	6	0	-1.868596	-3.084576	0.619359
15	6	0	-2.332641	-1.924572	-0.315248
16	6	0	-1.246798	-0.779800	0.021233
17	6	0	-0.031127	-1.638888	0.433489
18	6	0	-0.391818	-2.872968	0.771710
19	1	0	-2.361251	-3.034070	1.599230
20	1	0	-2.112074	-4.067418	0.207025
21	6	0	-2.182511	-2.428957	-1.764782
22	6	0	-1.680202	0.079843	1.232618
23	1	0	0.280576	-3.641203	1.131781
24	1	0	-2.478141	-1.687999	-2.508048
25	1	0	-1.150465	-2.716332	-1.973809
26	1	0	-2.801040	-3.314239	-1.922372
27	1	0	-0.872780	0.750186	1.523938
28	1	0	-2.554542	0.691040	1.004916
29	1	0	-1.920727	-0.536672	2.101002
30	6	0	-3.820542	-1.542083	-0.024775
31	6	0	-4.367064	-0.445935	-0.966008
32	6	0	-5.613100	0.270950	-0.471955
33	6	0	-6.189471	1.310248	-1.445124
34	6	0	-6.772965	2.386707	-0.521864
35	6	0	-5.948171	2.220949	0.752537
36	8	0	-5.292181	1.030690	0.729257
37	1	0	-6.384437	-0.447545	-0.182074
38	1	0	-6.927106	0.869577	-2.117389
39	1	0	-5.384158	1.725247	-2.056617
40	1	0	-6.600294	3.395016	-0.900574
41	6	0	-8.267849	2.222743	-0.210549
42	1	0	-3.852293	-1.143677	0.991068
43	1	0	-4.612056	-0.883370	-1.940417
44	1	0	-3.611194	0.318252	-1.153670
45	8	0	-5.862488	2.981017	1.675105
46	1	0	-8.583904	2.947188	0.541145
47	1	0	-8.860777	2.381157	-1.113632
48	1	0	-8.491625	1.222603	0.169210
49	1	0	1.367545	-0.974307	-1.650863
50	6	0	3.074488	0.100400	-0.966760
51	6	0	3.471939	0.111444	-2.450571

52	6	0	3.361991	1.485114	-0.329500
53	1	0	4.532906	0.312793	-2.602554
54	1	0	3.254564	-0.844958	-2.927968
55	1	0	2.912837	0.887615	-2.980807
56	6	0	4.829718	1.953116	-0.312195
57	6	0	4.939712	3.293782	0.374048
58	8	0	5.165646	3.458040	1.547795
59	8	0	4.722659	4.313218	-0.487128
60	6	0	4.755745	5.631376	0.088261
61	6	0	4.872458	-1.868715	-0.802878
62	1	0	4.168895	-0.639311	0.763327
63	6	0	4.703180	-3.030772	-1.433986
64	6	0	6.255263	-1.275341	-0.692505
65	1	0	5.545110	-3.543325	-1.884665
66	1	0	3.744182	-3.522172	-1.531116
67	1	0	6.519294	-1.092269	0.354178
68	1	0	7.004651	-1.942307	-1.119843
69	1	0	6.330113	-0.314752	-1.207797
70	1	0	5.226227	2.041353	-1.324447
71	1	0	5.450981	1.262141	0.255003
72	1	0	2.988812	1.480962	0.694009
73	1	0	2.782128	2.233566	-0.876373
74	8	0	2.552038	-3.901985	1.836364
75	1	0	3.735381	-2.329644	2.220972
76	6	0	1.317146	-0.548857	2.853723
77	1	0	1.148473	0.335976	3.467493
78	1	0	2.208638	-1.062252	3.228507
79	1	0	0.458798	-1.217853	2.944239
80	6	0	-4.760258	-2.762475	-0.047097
81	1	0	-5.774261	-2.476521	0.240399
82	1	0	-4.820953	-3.214530	-1.040181
83	1	0	-4.442935	-3.534844	0.653583
84	1	0	5.732896	5.829460	0.528005
85	1	0	4.560340	6.313784	-0.735059
86	1	0	3.991539	5.729202	0.858952

52

Center Number	Atomic Number	Atomic Type	Coordinates (Angstroms) X	Y	Z
1	6	0	-0.940060	0.143914	-1.135579
2	6	0	0.443988	0.811507	-1.030796
3	1	0	-0.953883	-0.416791	-2.070871
4	1	0	-1.703020	0.921272	-1.226341
5	1	0	0.477114	1.524468	-0.206222
6	1	0	0.626663	1.377602	-1.949125
7	6	0	3.705071	-1.134708	-0.187426
8	6	0	2.552791	-1.974128	0.418882
9	6	0	1.317741	-1.023229	0.480520
10	6	0	1.507618	-0.265723	-0.849423
11	6	0	2.959563	-2.708261	1.676130
12	1	0	2.256644	-2.772262	-0.271124
13	8	0	1.442321	-0.039726	1.525411
14	6	0	-1.907497	-3.080432	0.709153
15	6	0	-2.371696	-1.944516	-0.253891
16	6	0	-1.287331	-0.790774	0.056249
17	6	0	-0.065839	-1.635892	0.484307
18	6	0	-0.430935	-2.860504	0.850506
19	1	0	-2.398663	-3.003771	1.687927
20	1	0	-2.150865	-4.073260	0.321610
21	6	0	-2.219483	-2.483455	-1.690673
22	6	0	-1.718831	0.090258	1.252903
23	1	0	0.232126	-3.629109	1.227952
24	1	0	-2.520225	-1.762283	-2.450822
25	1	0	-1.185903	-2.768716	-1.894854
26	1	0	-2.833188	-3.375676	-1.826608
27	1	0	-0.914980	0.772992	1.523725
28	1	0	-2.599808	0.688437	1.017278
29	1	0	-1.948920	-0.509545	2.135749
30	6	0	-3.860507	-1.558428	0.027324
31	6	0	-4.407557	-0.483022	-0.936969
32	6	0	-5.661156	0.234842	-0.463711
33	6	0	-6.230628	1.260697	-1.455068
34	6	0	-6.827267	2.345837	-0.550440
35	6	0	-6.016111	2.197409	0.734737
36	8	0	-5.355128	1.009038	0.731659
37	1	0	-6.433237	-0.482959	-0.174079
38	1	0	-6.959810	0.809734	-2.129702
39	1	0	-5.420435	1.671337	-2.063005
40	1	0	-6.653293	3.350197	-0.938875
41	6	0	-8.325034	2.181027	-0.253769
42	1	0	-3.893514	-1.138628	1.034468
43	1	0	-4.643289	-0.938763	-1.905230
44	1	0	-3.655073	0.282330	-1.132566
45	8	0	-5.943338	2.967178	1.650045
46	1	0	-8.651522	2.913396	0.485660
47	1	0	-8.908214	2.326928	-1.165239
48	1	0	-8.550130	1.184782	0.135359
49	1	0	1.312507	-1.024171	-1.618769
50	6	0	3.024098	0.067648	-0.988419
51	6	0	3.385854	0.113696	-2.481308

52	6	0	3.350441	1.438084	-0.337703
53	1	0	4.453318	0.261762	-2.648697
54	1	0	3.109053	-0.809117	-2.991310
55	1	0	2.859417	0.940307	-2.966942
56	6	0	4.829380	1.872035	-0.368530
57	6	0	4.988665	3.214693	0.303612
58	8	0	5.187073	3.386786	1.480881
59	8	0	4.850049	4.231756	-0.578317
60	6	0	4.936802	5.554193	-0.019717
61	6	0	4.726622	-1.978176	-0.943004
62	1	0	4.237295	-0.698896	0.659276
63	6	0	4.401903	-3.000987	-1.732440
64	6	0	6.170117	-1.598895	-0.741788
65	1	0	5.164160	-3.570666	-2.251072
66	1	0	3.378726	-3.315108	-1.903024
67	1	0	6.839879	-2.271458	-1.278983
68	1	0	6.368261	-0.579846	-1.087252
69	1	0	6.424935	-1.630284	0.321745
70	1	0	5.199007	1.944229	-1.391759
71	1	0	5.448869	1.167699	0.186023
72	1	0	3.016265	1.426922	0.698498
73	1	0	2.765650	2.205297	-0.853476
74	8	0	4.061051	-2.671244	2.167520
75	1	0	2.177791	-3.348361	2.132343
76	6	0	1.299436	-0.463577	2.874998
77	1	0	0.455994	-1.146952	3.003097
78	1	0	2.210993	-0.935677	3.250362
79	1	0	1.115152	0.437469	3.460086
80	6	0	-4.797932	-2.780835	0.030439
81	1	0	-5.811446	-2.490992	0.315146
82	1	0	-4.860688	-3.251901	-0.953632
83	1	0	-4.478436	-3.539427	0.745202
84	1	0	4.807663	6.234021	-0.858211
85	1	0	4.153640	5.706358	0.722770
86	1	0	5.907847	5.704983	0.451286

53

Center Number	Atomic Number	Atomic Type	Coordinates (Angstroms) X	Y	Z
1	6	0	-0.960672	0.216039	-1.156765
2	6	0	0.435481	0.859981	-1.054183
3	1	0	-0.982700	-0.352121	-2.087515
4	1	0	-1.708649	1.006875	-1.256626
5	1	0	0.482298	1.578224	-0.234888
6	1	0	0.630336	1.417405	-1.975491
7	6	0	3.653015	-1.092603	-0.110952
8	6	0	2.477561	-1.976599	0.395061
9	6	0	1.270850	-0.984348	0.466330
10	6	0	1.478480	-0.236319	-0.866441
11	6	0	2.863407	-2.736519	1.642851
12	1	0	2.200410	-2.728947	-0.344554
13	8	0	1.440338	-0.006392	1.510472
14	6	0	-1.978925	-2.979845	0.712064
15	6	0	-2.428454	-1.842396	-0.257198
16	6	0	-1.326588	-0.702646	0.042595
17	6	0	-0.121945	-1.566726	0.477656
18	6	0	-0.498876	-2.784621	0.854329
19	1	0	-2.468785	-2.891498	1.690680
20	1	0	-2.237365	-3.971207	0.330533
21	6	0	-2.286947	-2.392411	-1.690815
22	6	0	-1.744476	0.198194	1.229186
23	1	0	0.162829	-3.552778	1.234259
24	1	0	-2.577860	-1.671861	-2.455677
25	1	0	-1.257963	-2.694966	-1.893381
26	1	0	-2.913868	-3.276444	-1.819899
27	1	0	-0.930917	0.872924	1.492482
28	1	0	-2.615805	0.808301	0.988161
29	1	0	-1.984119	-0.388180	2.118365
30	6	0	-3.910351	-1.431905	0.024805
31	6	0	-4.441464	-0.349167	-0.939901
32	6	0	-5.693672	0.376048	-0.474331
33	6	0	-6.240478	1.419864	-1.459524
34	6	0	-6.842335	2.496659	-0.548375
35	6	0	-6.051152	2.323964	0.746323
36	8	0	-5.396958	1.132346	0.735010
37	1	0	-6.476599	-0.337573	-0.204254
38	1	0	-6.963001	0.984031	-2.151097
39	1	0	-5.418210	1.832748	-2.049436
40	1	0	-6.655491	3.505089	-0.919906
41	6	0	-8.345379	2.338675	-0.275584
42	1	0	-3.934914	-1.009597	1.031211
43	1	0	-4.673439	-0.799298	-1.911733
44	1	0	-3.681098	0.410660	-1.126919
45	8	0	-5.988488	3.079580	1.674264
46	1	0	-8.677380	3.063355	0.468974
47	1	0	-8.914393	2.500842	-1.193265
48	1	0	-8.582982	1.338957	0.096892
49	1	0	1.265344	-0.993945	-1.630712
50	6	0	3.001324	0.064527	-1.001108
51	6	0	3.400679	0.026489	-2.486213

52	6	0	3.349669	1.462573	-0.426790
53	1	0	4.482972	0.030684	-2.621582
54	1	0	3.009251	-0.855898	-2.993966
55	1	0	2.997070	0.901280	-3.003745
56	6	0	4.822521	1.867882	-0.503419
57	6	0	5.062843	3.273141	0.002791
58	8	0	4.221805	4.121196	0.167690
59	8	0	6.378607	3.483670	0.236103
60	6	0	6.728354	4.806860	0.678433
61	6	0	4.792262	-1.910725	-0.701520
62	1	0	4.061565	-0.594285	0.771452
63	6	0	6.052424	-1.688016	-0.326812
64	6	0	4.477072	-3.032045	-1.663270
65	1	0	6.875052	-2.266825	-0.731175
66	1	0	6.309223	-0.927272	0.401258
67	1	0	5.388459	-3.409078	-2.128505
68	1	0	4.001766	-3.870183	-1.144409
69	1	0	3.794462	-2.723377	-2.456365
70	1	0	5.195836	1.845697	-1.532203
71	1	0	5.471714	1.194491	0.057667
72	1	0	3.020361	1.514847	0.610434
73	1	0	2.768420	2.213743	-0.965803
74	8	0	2.509484	-3.862708	1.898644
75	1	0	3.554520	-2.206766	2.330194
76	6	0	1.217968	-0.407798	2.855054
77	1	0	0.344712	-1.055664	2.953166
78	1	0	2.088911	-0.921587	3.274711
79	1	0	1.050473	0.505755	3.425855
80	6	0	-4.866745	-2.639519	0.032580
81	1	0	-5.872849	-2.335802	0.328767
82	1	0	-4.946144	-3.106202	-0.952459
83	1	0	-4.550788	-3.405359	0.740993
84	1	0	7.807465	4.794150	0.809295
85	1	0	6.440751	5.547019	-0.068019
86	1	0	6.230106	5.037939	1.619629

54

Center Number	Atomic Number	Atomic Type	Coordinates (Angstroms) X	Y	Z
1	6	0	-0.980094	0.515751	-0.760345
2	6	0	0.357268	1.107629	-0.277321
3	1	0	-0.882543	0.336313	-1.831575
4	1	0	-1.765256	1.267243	-0.640998
5	1	0	0.281939	1.464806	0.750497
6	1	0	0.599470	1.975079	-0.898890
7	6	0	3.595357	-0.976393	0.212074
8	6	0	2.434568	-2.011147	0.236225
9	6	0	1.166191	-1.150038	0.534989
10	6	0	1.446167	0.046369	-0.396816
11	6	0	2.754048	-3.171967	1.150419
12	1	0	2.272169	-2.442146	-0.752556
13	8	0	1.171743	-0.629049	1.879622
14	6	0	-1.960820	-3.170482	-0.330633
15	6	0	-2.392675	-1.763642	-0.849591
16	6	0	-1.394854	-0.793630	-0.033207
17	6	0	-0.182092	-1.728780	0.178059
18	6	0	-0.514840	-3.007364	0.030882
19	1	0	-2.539380	-3.470218	0.552851
20	1	0	-2.121528	-3.951427	-1.078683
21	6	0	-2.089624	-1.731723	-2.361019
22	6	0	-1.974496	-0.416850	1.351102
23	1	0	0.156803	-3.845799	0.165956
24	1	0	-2.353878	-0.783174	-2.828926
25	1	0	-1.030398	-1.913835	-2.551292
26	1	0	-2.648272	-2.515891	-2.874973
27	1	0	-1.233854	0.128473	1.934197
28	1	0	-2.857754	0.216532	1.262006
29	1	0	-2.257465	-1.300712	1.925747
30	6	0	-3.917512	-1.524942	-0.594922
31	6	0	-4.431642	-0.179363	-1.151827
32	6	0	-5.776330	0.278943	-0.592023
33	6	0	-6.252195	1.642227	-1.128182
34	6	0	-5.774982	2.640066	-0.068266
35	6	0	-5.672991	1.779587	1.188025
36	8	0	-5.682289	0.463856	0.850518
37	1	0	-6.534804	-0.489492	-0.735480
38	1	0	-7.343250	1.661312	-1.189050
39	1	0	-5.859440	1.847336	-2.124375
40	1	0	-4.748160	2.956085	-0.287997
41	6	0	-6.637467	3.881871	0.132553
42	1	0	-4.057567	-1.503604	0.487229
43	1	0	-4.545898	-0.249705	-2.238864
44	1	0	-3.704093	0.614636	-0.971757
45	8	0	-5.582754	2.147364	2.325204
46	1	0	-6.266236	4.473415	0.970042
47	1	0	-6.631239	4.506167	-0.763421
48	1	0	-7.672598	3.606497	0.347958
49	1	0	1.350797	-0.375983	-1.404659
50	6	0	2.954300	0.413420	-0.250902
51	6	0	3.483653	0.937942	-1.595043

52	6	0	3.158696	1.497780	0.840554
53	1	0	4.569256	1.043903	-1.597860
54	1	0	3.215466	0.279522	-2.422240
55	1	0	3.050120	1.918146	-1.809128
56	6	0	4.600764	1.974466	1.101293
57	6	0	5.195413	2.973877	0.133241
58	8	0	6.311783	2.918501	-0.321433
59	8	0	4.346121	3.992835	-0.130426
60	6	0	4.857885	5.018945	-0.997702
61	6	0	4.825055	-1.495577	-0.520813
62	1	0	3.901695	-0.831394	1.251045
63	6	0	6.034481	-1.392766	0.029901
64	6	0	4.656768	-2.202126	-1.845600
65	1	0	6.920764	-1.759794	-0.474529
66	1	0	6.187117	-0.937979	1.001599
67	1	0	5.625029	-2.365844	-2.319632
68	1	0	4.187455	-3.181498	-1.710280
69	1	0	4.029470	-1.645233	-2.543548
70	1	0	5.305112	1.147283	1.163711
71	1	0	4.613883	2.474781	2.076171
72	1	0	2.759467	1.111724	1.777803
73	1	0	2.556198	2.371556	0.585433
74	8	0	2.440310	-4.318928	0.939060
75	1	0	3.354629	-2.922028	2.049067
76	6	0	0.869450	-1.518651	2.946256
77	1	0	0.546199	-0.900990	3.784579
78	1	0	0.069299	-2.214997	2.687347
79	1	0	1.750165	-2.089686	3.257513
80	6	0	-4.791530	-2.675844	-1.129043
81	1	0	-5.838468	-2.526825	-0.857515
82	1	0	-4.746009	-2.753148	-2.218331
83	1	0	-4.495832	-3.638853	-0.712799
84	1	0	4.052614	5.742118	-1.100638
85	1	0	5.738940	5.485384	-0.557491
86	1	0	5.125052	4.599776	-1.967545

55

Center Number	Atomic Number	Atomic Type	Coordinates (Angstroms) X	Y	Z
1	6	0	-0.913464	0.606201	-0.805959
2	6	0	0.436034	1.172310	-0.327656
3	1	0	-0.815233	0.401398	-1.872594
4	1	0	-1.678449	1.380747	-0.706495
5	1	0	0.363563	1.550900	0.692551
6	1	0	0.704134	2.021565	-0.963022
7	6	0	3.620343	-0.989828	0.198009
8	6	0	2.430863	-1.985822	0.247388
9	6	0	1.184029	-1.090850	0.530060
10	6	0	1.498492	0.081445	-0.420073
11	6	0	2.722125	-3.147053	1.173520
12	1	0	2.245292	-2.426370	-0.732045
13	8	0	1.197674	-0.550812	1.867170
14	6	0	-1.994313	-3.042551	-0.308980
15	6	0	-2.388453	-1.633964	-0.851752
16	6	0	-1.365032	-0.676330	-0.052560
17	6	0	-0.178753	-1.639911	0.178180
18	6	0	-0.545194	-2.911732	0.052473
19	1	0	-2.581528	-3.313104	0.578361
20	1	0	-2.174672	-3.831472	-1.044306
21	6	0	-2.087077	-1.635717	-2.363709
22	6	0	-1.935859	-0.254730	1.322667
23	1	0	0.103776	-3.764529	0.205716
24	1	0	-2.329022	-0.689119	-2.847406
25	1	0	-1.032663	-1.846198	-2.551108
26	1	0	-2.664720	-2.415353	-2.863488
27	1	0	-1.179434	0.280286	1.895102
28	1	0	-2.800905	0.401383	1.218973
29	1	0	-2.242561	-1.118959	1.915291
30	6	0	-3.905560	-1.350724	-0.600491
31	6	0	-4.381730	0.000474	-1.178752
32	6	0	-5.686558	0.527711	-0.604841
33	6	0	-6.184259	1.838845	-1.231805
34	6	0	-6.878177	2.557811	-0.068558
35	6	0	-6.189148	1.962090	1.156971
36	8	0	-5.507259	0.839759	0.807249
37	1	0	-6.467559	-0.235912	-0.651838
38	1	0	-6.842997	1.655572	-2.081845
39	1	0	-5.331316	2.421899	-1.588537
40	1	0	-6.682198	3.630934	-0.070027
41	6	0	-8.394973	2.331827	0.013169
42	1	0	-4.043606	-1.308142	0.481503
43	1	0	-4.520561	-0.089264	-2.261933
44	1	0	-3.628135	0.775099	-1.028934
45	8	0	-6.219024	2.367444	2.284433
46	1	0	-8.798711	2.773025	0.925373
47	1	0	-8.891592	2.793381	-0.842769
48	1	0	-8.644056	1.267473	0.014235
49	1	0	1.401601	-0.355328	-1.421604
50	6	0	3.014606	0.419430	-0.265037
51	6	0	3.555070	0.892785	-1.622070

52	6	0	3.207786	1.518196	0.813652
53	1	0	4.625827	1.101155	-1.598002
54	1	0	3.395533	0.139310	-2.394892
55	1	0	3.051238	1.812152	-1.928125
56	6	0	4.635499	1.999214	1.109319
57	6	0	5.167812	3.035191	0.143491
58	8	0	4.520843	3.921446	-0.356639
59	8	0	6.496264	2.884799	-0.063944
60	6	0	7.110715	3.875437	-0.908265
61	6	0	4.824860	-1.533137	-0.564728
62	1	0	3.952241	-0.851768	1.233115
63	6	0	4.732980	-2.420262	-1.555706
64	6	0	6.184738	-1.050074	-0.124077
65	1	0	5.622189	-2.782930	-2.058118
66	1	0	3.793299	-2.822525	-1.910202
67	1	0	6.978916	-1.587144	-0.643899
68	1	0	6.325958	0.016430	-0.316073
69	1	0	6.322499	-1.199050	0.952085
70	1	0	5.350536	1.184026	1.202153
71	1	0	4.627523	2.504466	2.082156
72	1	0	2.772932	1.154862	1.743465
73	1	0	2.629351	2.396757	0.520514
74	8	0	2.293645	-4.266695	1.033930
75	1	0	3.411725	-2.923106	2.015089
76	6	0	0.898925	-1.428893	2.943097
77	1	0	0.600966	-0.801408	3.783488
78	1	0	0.083347	-2.113650	2.701338
79	1	0	1.775560	-2.013350	3.242470
80	6	0	-4.811722	-2.484832	-1.115600
81	1	0	-5.854829	-2.297743	-0.852048
82	1	0	-4.764950	-2.584102	-2.202993
83	1	0	-4.547149	-3.448361	-0.679931
84	1	0	6.986321	4.869619	-0.479856
85	1	0	8.162910	3.606331	-0.954367
86	1	0	6.664847	3.857235	-1.902373

56

Center Number	Atomic Number	Atomic Type	Coordinates (Angstroms) X	Y	Z
1	6	0	-0.923726	0.021683	-1.165714
2	6	0	0.440621	0.741015	-1.154558
3	1	0	-0.965306	-0.558944	-2.087742
4	1	0	-1.718437	0.769164	-1.233154
5	1	0	0.485620	1.491679	-0.365008
6	1	0	0.561212	1.271008	-2.103595
7	6	0	3.794522	-0.884866	-0.193858
8	6	0	2.731312	-1.883232	0.332192
9	6	0	1.441356	-0.995236	0.391592
10	6	0	1.540272	-0.301432	-0.981972
11	6	0	3.184597	-2.572376	1.597919
12	1	0	2.525015	-2.674834	-0.391282
13	8	0	1.557061	0.040693	1.386675
14	6	0	-1.650981	-3.203083	0.794212
15	6	0	-2.208788	-2.120102	-0.181108
16	6	0	-1.178890	-0.901335	0.061247
17	6	0	0.096415	-1.673340	0.471781
18	6	0	-0.184130	-2.905486	0.883876
19	1	0	-2.114707	-3.129177	1.786605
20	1	0	-1.851616	-4.217328	0.439209
21	6	0	-2.072912	-2.690418	-1.607224
22	6	0	-1.620513	-0.011882	1.247587
23	1	0	0.540975	-3.618743	1.255237
24	1	0	-2.435770	-2.008643	-2.376660
25	1	0	-1.032120	-2.926346	-1.836311
26	1	0	-2.641355	-3.617601	-1.698343
27	1	0	-0.858406	0.736285	1.461157
28	1	0	-2.549850	0.516401	1.033124
29	1	0	-1.773513	-0.594766	2.157674
30	6	0	-3.707471	-1.808525	0.140971
31	6	0	-4.343693	-0.782028	-0.821581
32	6	0	-5.659168	-0.173295	-0.341204
33	6	0	-6.238668	0.902520	-1.278643
34	6	0	-5.728738	2.219943	-0.685537
35	6	0	-5.488421	1.863628	0.778867
36	8	0	-5.460319	0.513953	0.928463
37	1	0	-6.390461	-0.953756	-0.134936
38	1	0	-7.330819	0.880531	-1.244502
39	1	0	-5.934787	0.747188	-2.314186
40	1	0	-4.738510	2.455153	-1.093514
41	6	0	-6.631470	3.437054	-0.859202
42	1	0	-3.732958	-1.373281	1.141606
43	1	0	-4.546948	-1.257906	-1.787023
44	1	0	-3.647978	0.034682	-1.023883
45	8	0	-5.328146	2.612446	1.700915
46	1	0	-6.226182	4.292962	-0.318629
47	1	0	-6.722012	3.703789	-1.914229
48	1	0	-7.633313	3.237638	-0.471477
49	1	0	1.289603	-1.103502	-1.685535
50	6	0	3.047171	0.014724	-1.266238
51	6	0	3.347218	-0.408888	-2.718465

52	6	0	3.461002	1.505340	-1.185874
53	1	0	4.403966	-0.287954	-2.963859
54	1	0	3.068256	-1.446605	-2.907848
55	1	0	2.773826	0.213375	-3.411269
56	6	0	3.400383	2.235722	0.156501
57	6	0	3.560088	3.731536	0.001447
58	8	0	3.396787	4.363708	-1.013217
59	8	0	3.891037	4.306079	1.180272
60	6	0	4.010376	5.739237	1.162885
61	6	0	5.122133	-1.499252	-0.607589
62	1	0	4.015684	-0.227648	0.648051
63	6	0	6.261558	-0.858549	-0.341956
64	6	0	5.144625	-2.847906	-1.284681
65	1	0	7.222786	-1.263671	-0.637059
66	1	0	6.274981	0.092071	0.179382
67	1	0	6.161241	-3.115743	-1.574743
68	1	0	4.773125	-3.632223	-0.618180
69	1	0	4.520618	-2.875475	-2.180277
70	1	0	4.164688	1.892455	0.856076
71	1	0	2.449308	2.067048	0.665902
72	1	0	2.851898	2.063019	-1.900405
73	1	0	4.485150	1.581549	-1.565580
74	8	0	2.908065	-3.709761	1.897142
75	1	0	3.850523	-1.977922	2.256024
76	6	0	1.433144	-0.322357	2.755698
77	1	0	0.672603	-1.090355	2.909472
78	1	0	2.384542	-0.675243	3.166254
79	1	0	1.140794	0.579837	3.293671
80	6	0	-4.576455	-3.079513	0.196256
81	1	0	-5.585809	-2.843882	0.538884
82	1	0	-4.668389	-3.556761	-0.782688
83	1	0	-4.176863	-3.817200	0.892134
84	1	0	4.788612	6.048312	0.465502
85	1	0	3.066017	6.196881	0.868842
86	1	0	4.272560	6.021721	2.179355

57

Center Number	Atomic Number	Atomic Type	Coordinates (Angstroms) X	Y	Z
1	6	0	-0.894564	0.609838	-0.785323
2	6	0	0.460206	1.163969	-0.306394
3	1	0	-0.800884	0.416730	-1.854525
4	1	0	-1.655251	1.387095	-0.674816
5	1	0	0.395405	1.534528	0.717292
6	1	0	0.731781	2.016337	-0.935990
7	6	0	3.626454	-1.008640	0.240232
8	6	0	2.437555	-2.010863	0.256766
9	6	0	1.194390	-1.106705	0.536634
10	6	0	1.514397	0.066689	-0.410903
11	6	0	2.711563	-3.171296	1.184998
12	1	0	2.273087	-2.444367	-0.730737
13	8	0	1.213064	-0.566032	1.873231
14	6	0	-1.986702	-3.042413	-0.321744
15	6	0	-2.374990	-1.628284	-0.855203
16	6	0	-1.352120	-0.679086	-0.044984
17	6	0	-0.169534	-1.647996	0.182157
18	6	0	-0.538434	-2.917887	0.045514
19	1	0	-2.577964	-3.317926	0.561336
20	1	0	-2.166475	-3.825075	-1.063808
21	6	0	-2.068032	-1.619223	-2.365920
22	6	0	-1.926943	-0.266443	1.331311
23	1	0	0.107635	-3.774454	0.191164
24	1	0	-2.306731	-0.668570	-2.843129
25	1	0	-1.013295	-1.830109	-2.551125
26	1	0	-2.645145	-2.394051	-2.873709
27	1	0	-1.172920	0.267371	1.908045
28	1	0	-2.792335	0.389268	1.228844
29	1	0	-2.234319	-1.133938	1.918746
30	6	0	-3.892204	-1.343540	-0.607008
31	6	0	-4.362606	0.014192	-1.174464
32	6	0	-5.668412	0.539067	-0.600573
33	6	0	-6.160291	1.857632	-1.216506
34	6	0	-6.856486	2.567159	-0.048823
35	6	0	-6.174242	1.957176	1.173436
36	8	0	-5.493947	0.836453	0.815213
37	1	0	-6.451132	-0.222066	-0.658334
38	1	0	-6.816496	1.684325	-2.070586
39	1	0	-5.304533	2.441752	-1.564635
40	1	0	-6.657104	3.639606	-0.038864
41	6	0	-8.374326	2.345291	0.024676
42	1	0	-4.034035	-1.310599	0.474840
43	1	0	-4.497432	-0.065140	-2.258947
44	1	0	-3.607570	0.785361	-1.014325
45	8	0	-6.207660	2.351102	2.304805
46	1	0	-8.780198	2.778320	0.939843
47	1	0	-8.866100	2.817348	-0.828324
48	1	0	-8.626944	1.281815	0.013687
49	1	0	1.405943	-0.365864	-1.413139
50	6	0	3.033052	0.387560	-0.265878
51	6	0	3.582855	0.858862	-1.621918

52	6	0	3.276058	1.492372	0.796161
53	1	0	4.672243	0.932606	-1.621195
54	1	0	3.300554	0.183965	-2.431091
55	1	0	3.185835	1.846768	-1.862959
56	6	0	4.729818	1.953790	0.980074
57	6	0	5.144897	3.109561	0.094358
58	8	0	4.405532	3.926278	-0.397245
59	8	0	6.488126	3.153631	-0.046338
60	6	0	7.000295	4.262506	-0.805996
61	6	0	4.862848	-1.578883	-0.440453
62	1	0	3.905832	-0.847748	1.284348
63	6	0	6.052890	-1.515113	0.158455
64	6	0	4.721196	-2.297951	-1.761177
65	1	0	6.943263	-1.926428	-0.303291
66	1	0	6.180911	-1.057957	1.132963
67	1	0	5.700872	-2.521054	-2.185103
68	1	0	4.194447	-3.248542	-1.633273
69	1	0	4.158081	-1.720977	-2.496097
70	1	0	5.452115	1.147042	0.859735
71	1	0	4.863123	2.322976	2.003538
72	1	0	2.895864	1.134848	1.752018
73	1	0	2.685346	2.371995	0.533230
74	8	0	2.379151	-4.313186	0.973756
75	1	0	3.296271	-2.928213	2.095803
76	6	0	0.875238	-1.427246	2.952096
77	1	0	0.069661	-2.116755	2.691748
78	1	0	1.741035	-2.004471	3.292732
79	1	0	0.544741	-0.787707	3.771089
80	6	0	-4.799271	-2.470488	-1.136034
81	1	0	-5.842567	-2.284218	-0.872758
82	1	0	-4.750300	-2.558185	-2.224334
83	1	0	-4.537532	-3.439118	-0.710052
84	1	0	6.726862	5.206244	-0.334467
85	1	0	8.079969	4.136137	-0.812934
86	1	0	6.604925	4.243487	-1.821241

58

Center Number	Atomic Number	Atomic Type	Coordinates (Angstroms) X	Y	Z
1	6	0	-0.887572	0.070735	-1.074975
2	6	0	0.475866	0.757613	-0.874266
3	1	0	-0.866753	-0.396694	-2.060017
4	1	0	-1.668954	0.834518	-1.109720
5	1	0	0.472968	1.383787	0.019084
6	1	0	0.653765	1.415205	-1.729268
7	6	0	3.764445	-1.165094	-0.089416
8	6	0	2.626135	-2.117174	0.329889
9	6	0	1.366495	-1.201783	0.456157
10	6	0	1.570677	-0.301596	-0.779041
11	6	0	2.991774	-2.987894	1.511725
12	1	0	2.382540	-2.823856	-0.471733
13	8	0	1.452152	-0.324213	1.596534
14	6	0	-1.809051	-3.347527	0.393191
15	6	0	-2.293162	-2.118826	-0.436821
16	6	0	-1.235719	-0.984139	0.009513
17	6	0	-0.001700	-1.842317	0.366712
18	6	0	-0.341604	-3.109081	0.583474
19	1	0	-2.313621	-3.400666	1.366401
20	1	0	-2.019583	-4.293467	-0.112855
21	6	0	-2.121423	-2.488744	-1.924142
22	6	0	-1.704108	-0.240019	1.282392
23	1	0	0.334104	-3.900660	0.882105
24	1	0	-2.426400	-1.689557	-2.599859
25	1	0	-1.082133	-2.734909	-2.150002
26	1	0	-2.720309	-3.368385	-2.166523
27	1	0	-0.921387	0.428281	1.636894
28	1	0	-2.594437	0.361126	1.094314
29	1	0	-1.937279	-0.931361	2.094354
30	6	0	-3.792569	-1.801830	-0.123599
31	6	0	-4.360893	-0.630686	-0.954370
32	6	0	-5.681585	-0.056798	-0.445211
33	6	0	-6.184425	1.167210	-1.233547
34	6	0	-5.671493	2.361949	-0.423208
35	6	0	-5.514429	1.780661	0.978986
36	8	0	-5.527527	0.423214	0.921499
37	1	0	-6.443083	-0.833898	-0.394532
38	1	0	-7.277077	1.176602	-1.254455
39	1	0	-5.831989	1.160272	-2.265335
40	1	0	-4.656093	2.623259	-0.744373
41	6	0	-6.532602	3.620600	-0.449916
42	1	0	-3.840994	-1.505631	0.925726
43	1	0	-4.533432	-0.957903	-1.985383
44	1	0	-3.636812	0.184778	-1.009558
45	8	0	-5.382289	2.375348	2.010962
46	1	0	-6.132367	4.371104	0.232438
47	1	0	-6.563928	4.046320	-1.454999
48	1	0	-7.557180	3.398541	-0.142326
49	1	0	1.411442	-0.977515	-1.629522
50	6	0	3.085164	0.069431	-0.848927
51	6	0	3.490924	0.173410	-2.327190

52	6	0	3.510493	1.355685	-0.088451
53	1	0	4.531585	0.484218	-2.446870
54	1	0	3.375557	-0.781833	-2.839620
55	1	0	2.866128	0.903811	-2.847348
56	6	0	3.145846	2.712159	-0.701951
57	6	0	3.839357	3.857613	0.004658
58	8	0	4.891299	3.793468	0.590740
59	8	0	3.138092	5.006623	-0.126370
60	6	0	3.740396	6.172752	0.462715
61	6	0	4.931076	-1.835141	-0.796296
62	1	0	4.170335	-0.765539	0.841490
63	6	0	4.810072	-2.886710	-1.604793
64	6	0	6.283303	-1.234896	-0.512112
65	1	0	5.677658	-3.324445	-2.084766
66	1	0	3.859373	-3.358594	-1.823437
67	1	0	6.511215	-1.312759	0.555391
68	1	0	7.072859	-1.737311	-1.072363
69	1	0	6.312577	-0.170017	-0.762157
70	1	0	2.075574	2.909576	-0.697569
71	1	0	3.466065	2.773053	-1.747585
72	1	0	4.598225	1.333544	0.008094
73	1	0	3.115364	1.306827	0.926688
74	8	0	4.046878	-2.949796	2.095178
75	1	0	2.223431	-3.724762	1.820446
76	6	0	1.279517	-0.881890	2.894051
77	1	0	2.193864	-1.358935	3.254602
78	1	0	1.041029	-0.049295	3.556217
79	1	0	0.457443	-1.601616	2.923801
80	6	0	-4.700498	-3.037413	-0.273768
81	1	0	-5.712689	-2.818665	0.071183
82	1	0	-4.774468	-3.368505	-1.312690
83	1	0	-4.345106	-3.878957	0.320826
84	1	0	4.710121	6.372247	0.007035
85	1	0	3.049125	6.988261	0.265627
86	1	0	3.872635	6.031696	1.535031

Coordinates of conformers at B3LYP/6-31G+(d,p) in the PCM ($CHCl_3$)

1

Center Number	Atomic Number	Atomic Type	Coordinates (Angstroms) X	Y	Z
1	6	0	-1.086187	0.145393	-1.149989
2	6	0	0.309409	0.798067	-1.051480
3	1	0	-1.111393	-0.415812	-2.088103
4	1	0	-1.842344	0.933797	-1.237700
5	1	0	0.358283	1.505040	-0.218361
6	1	0	0.492094	1.371400	-1.968617
7	6	0	3.556225	-1.168552	-0.193426
8	6	0	2.386393	-2.063946	0.311950
9	6	0	1.174181	-1.076507	0.427968
10	6	0	1.364402	-0.297209	-0.892340
11	6	0	2.802960	-2.862841	1.525638
12	1	0	2.087693	-2.794171	-0.443890
13	8	0	1.357361	-0.122988	1.496900
14	6	0	-2.087806	-3.074407	0.704016
15	6	0	-2.547782	-1.922599	-0.249599
16	6	0	-1.435162	-0.789607	0.045491
17	6	0	-0.221658	-1.661862	0.450149
18	6	0	-0.600885	-2.888766	0.823662
19	1	0	-2.561798	-2.990039	1.694110
20	1	0	-2.359057	-4.063831	0.318116
21	6	0	-2.430701	-2.458828	-1.694853
22	6	0	-1.828546	0.096082	1.256875
23	1	0	0.065178	-3.665001	1.185800
24	1	0	-2.733756	-1.727584	-2.448779
25	1	0	-1.402989	-2.761333	-1.918361
26	1	0	-3.064075	-3.341891	-1.822253
27	1	0	-1.002055	0.757709	1.523916
28	1	0	-2.696673	0.722501	1.033102
29	1	0	-2.067695	-0.505736	2.139125
30	6	0	-4.027679	-1.510271	0.062471
31	6	0	-4.565814	-0.399081	-0.870265
32	6	0	-5.871636	0.260586	-0.422433
33	6	0	-6.324418	1.438661	-1.309191
34	6	0	-5.763787	2.677603	-0.593949
35	6	0	-5.652095	2.206415	0.852131
36	8	0	-5.706823	0.855170	0.910900
37	1	0	-6.663323	-0.481415	-0.305630
38	1	0	-7.418381	1.489791	-1.337371
39	1	0	-5.965598	1.337024	-2.336380
40	1	0	-4.730496	2.862593	-0.920801
41	6	0	-6.566448	3.970114	-0.749547
42	1	0	-4.036838	-1.114400	1.083057
43	1	0	-4.756201	-0.813638	-1.868819
44	1	0	-3.816936	0.387423	-1.004045
45	8	0	-5.517654	2.882621	1.851147
46	1	0	-6.128629	4.771679	-0.148569
47	1	0	-6.574362	4.288796	-1.796317
48	1	0	-7.603453	3.828180	-0.427331
49	1	0	1.151775	-1.040222	-1.674661
50	6	0	2.887543	0.024454	-1.031991

51	6	0	3.260945	0.006983	-2.526430
52	6	0	3.200631	1.420004	-0.423274
53	1	0	4.325355	0.189474	-2.698444
54	1	0	3.019577	-0.954976	-2.987242
55	1	0	2.703000	0.786374	-3.058516
56	6	0	4.667212	1.863156	-0.424111
57	6	0	4.853867	3.270687	0.105201
58	8	0	3.983965	4.116248	0.214315
59	8	0	6.144619	3.500212	0.430413
60	6	0	6.465405	4.828238	0.899143
61	6	0	4.679597	-1.960164	-0.863242
62	1	0	3.995917	-0.705836	0.700428
63	6	0	4.489973	-3.122792	-1.506325
64	6	0	6.074821	-1.386554	-0.749718
65	1	0	5.326050	-3.648151	-1.960073
66	1	0	3.518658	-3.594084	-1.613398
67	1	0	6.169365	-0.434165	-1.282746
68	1	0	6.334889	-1.189235	0.298158
69	1	0	6.818785	-2.073214	-1.162415
70	1	0	5.092460	1.864458	-1.436166
71	1	0	5.301854	1.200772	0.170642
72	1	0	2.827826	1.444254	0.603051
73	1	0	2.625720	2.168621	-0.977964
74	8	0	2.420792	-3.993465	1.777533
75	1	0	3.539349	-2.373290	2.196054
76	6	0	1.168213	-0.564344	2.837633
77	1	0	1.066162	0.337831	3.445561
78	1	0	0.267226	-1.175899	2.948116
79	1	0	2.032309	-1.132220	3.205848
80	6	0	-4.992064	-2.716705	0.054889
81	1	0	-5.982173	-2.425325	0.418807
82	1	0	-5.122676	-3.132499	-0.950491
83	1	0	-4.643103	-3.520802	0.707514
84	1	0	5.908002	5.053937	1.810766
85	1	0	7.535976	4.813257	1.098722
86	1	0	6.227774	5.569024	0.132581

2

Center Number	Atomic Number	Atomic Type	Coordinates (Angstroms) X	Y	Z
1	6	0	-0.821828	0.153939	-1.055909
2	6	0	0.548310	0.821810	-0.807185
3	1	0	-0.789982	-0.266699	-2.064790
4	1	0	-1.598012	0.927546	-1.065614
5	1	0	0.541768	1.412727	0.114046
6	1	0	0.739058	1.512397	-1.636219
7	6	0	3.801985	-1.130115	0.018385
8	6	0	2.658113	-2.149205	0.248513
9	6	0	1.403117	-1.222290	0.430299
10	6	0	1.636037	-0.253285	-0.750566
11	6	0	3.015081	-3.129188	1.343581
12	1	0	2.452557	-2.745859	-0.645554
13	8	0	1.499548	-0.418950	1.626945
14	6	0	-1.795399	-3.328354	0.249760
15	6	0	-2.256657	-2.056377	-0.535609
16	6	0	-1.192587	-0.953078	-0.026544
17	6	0	0.030083	-1.843209	0.298699
18	6	0	-0.321991	-3.122949	0.462920
19	1	0	-2.310278	-3.411379	1.219094
20	1	0	-2.016928	-4.253301	-0.295123
21	6	0	-2.066616	-2.363108	-2.039143
22	6	0	-1.665731	-0.265240	1.281176
23	1	0	0.355088	-3.926713	0.731756
24	1	0	-2.361532	-1.532803	-2.685929
25	1	0	-1.021864	-2.602269	-2.260691
26	1	0	-2.665610	-3.232198	-2.327460
27	1	0	-0.875929	0.376638	1.676471
28	1	0	-2.547941	0.358469	1.111452
29	1	0	-1.916378	-0.995903	2.056437
30	6	0	-3.760864	-1.738038	-0.227516
31	6	0	-4.298418	-0.507805	-0.996855
32	6	0	-5.666842	0.005973	-0.545521
33	6	0	-6.123645	1.299576	-1.250013
34	6	0	-5.676752	2.417518	-0.295544
35	6	0	-5.639907	1.707971	1.054127
36	8	0	-5.628201	0.366110	0.878455
37	1	0	-6.421701	-0.778451	-0.618859
38	1	0	-7.214777	1.309478	-1.347590
39	1	0	-5.695508	1.392355	-2.251177
40	1	0	-4.634103	2.694751	-0.507605
41	6	0	-6.531245	3.685914	-0.291094
42	1	0	-3.822996	-1.507630	0.841184
43	1	0	-4.398003	-0.752201	-2.062383
44	1	0	-3.587070	0.321836	-0.936764
45	8	0	-5.609024	2.206194	2.160634
46	1	0	-6.178098	4.387626	0.469341
47	1	0	-6.481915	4.180151	-1.266088
48	1	0	-7.579752	3.451183	-0.079365
49	1	0	1.482882	-0.878626	-1.642040
50	6	0	3.159976	0.108296	-0.768199
51	6	0	3.624209	0.183426	-2.235573

52	6	0	3.549122	1.403480	0.002951
53	1	0	4.665748	0.508700	-2.327278
54	1	0	3.537892	-0.790749	-2.725479
55	1	0	3.006107	0.891059	-2.799087
56	6	0	3.280739	2.747631	-0.687035
57	6	0	3.826000	3.922375	0.098977
58	8	0	4.652665	3.865384	0.992182
59	8	0	3.293416	5.085906	-0.333223
60	6	0	3.773144	6.290522	0.303190
61	6	0	5.089352	-1.714378	-0.545471
62	1	0	4.056552	-0.742849	1.014168
63	6	0	5.149840	-2.877930	-1.211689
64	6	0	6.349564	-0.922269	-0.274633
65	1	0	6.098396	-3.258661	-1.581031
66	1	0	4.277791	-3.491074	-1.413755
67	1	0	6.307700	0.077050	-0.722688
68	1	0	6.491976	-0.774640	0.803500
69	1	0	7.232207	-1.431249	-0.671400
70	1	0	2.219038	2.931056	-0.864196
71	1	0	3.763324	2.793201	-1.671687
72	1	0	4.622974	1.359022	0.211169
73	1	0	3.050203	1.390046	0.975867
74	8	0	2.647052	-4.291555	1.383299
75	1	0	3.690850	-2.743998	2.134359
76	6	0	1.290677	-1.054848	2.884324
77	1	0	1.073219	-0.259307	3.601117
78	1	0	0.448854	-1.754304	2.857709
79	1	0	2.188775	-1.587107	3.223157
80	6	0	-4.681379	-2.954731	-0.467837
81	1	0	-5.689322	-2.760538	-0.089003
82	1	0	-4.772838	-3.196813	-1.532694
83	1	0	-4.319558	-3.846700	0.049269
84	1	0	4.849787	6.396094	0.153085
85	1	0	3.237023	7.105883	-0.180484
86	1	0	3.553746	6.267828	1.372705

3

Center Number	Atomic Number	Atomic Type	Coordinates (Angstroms) X	Y	Z
1	6	0	-1.052220	0.069589	-1.179980
2	6	0	0.325530	0.764189	-1.130240
3	1	0	-1.073640	-0.531321	-2.093370
4	1	0	-1.831600	0.832149	-1.289800
5	1	0	0.366260	1.504299	-0.325980
6	1	0	0.477360	1.304969	-2.072350
7	6	0	3.638430	-1.073180	-0.238330
8	6	0	2.501380	-1.982620	0.313370
9	6	0	1.263100	-1.026000	0.408730
10	6	0	1.412970	-0.295030	-0.944010
11	6	0	2.954750	-2.727640	1.548450
12	1	0	2.213820	-2.747240	-0.412450
13	8	0	1.434150	-0.024570	1.435440
14	6	0	-1.939810	-3.095091	0.819100
15	6	0	-2.446300	-1.994321	-0.170000
16	6	0	-1.357850	-0.823271	0.058880
17	6	0	-0.115510	-1.647041	0.476700
18	6	0	-0.456380	-2.867751	0.903980
19	1	0	-2.397590	-2.983941	1.814120
20	1	0	-2.192310	-4.105371	0.477100
21	6	0	-2.340510	-2.584431	-1.595060
22	6	0	-1.753360	0.100999	1.240330
23	1	0	0.235450	-3.612221	1.284070
24	1	0	-2.673360	-1.890461	-2.371260
25	1	0	-1.309370	-2.870431	-1.824330
26	1	0	-2.953760	-3.486851	-1.677680
27	1	0	-0.942460	0.797619	1.462920
28	1	0	-2.644800	0.690959	1.010130
29	1	0	-1.955930	-0.470901	2.151140
30	6	0	-3.930770	-1.606371	0.152570
31	6	0	-4.515100	-0.553421	-0.819430
32	6	0	-5.825050	0.098689	-0.373210
33	6	0	-6.335130	1.208719	-1.314840
34	6	0	-5.790010	2.503449	-0.692070
35	6	0	-5.619770	2.123069	0.775090
36	8	0	-5.637920	0.777279	0.916460
37	1	0	-6.592420	-0.654721	-0.187590
38	1	0	-7.430390	1.229309	-1.313760
39	1	0	-6.002470	1.054319	-2.344280
40	1	0	-4.773301	2.698009	-1.062450
41	6	0	-6.636221	3.760819	-0.897770
42	1	0	-3.931850	-1.164571	1.154340
43	1	0	-4.717890	-1.020171	-1.792120
44	1	0	-3.787470	0.241809	-1.008030
45	8	0	-5.469871	2.861589	1.726770
46	1	0	-6.205211	4.609599	-0.359990
47	1	0	-6.685861	4.015189	-1.960840
48	1	0	-7.658111	3.608549	-0.534510
49	1	0	1.211050	-1.073330	-1.694070
50	6	0	2.925410	0.061440	-1.118160
51	6	0	3.281050	-0.007230	-2.614920

52	6	0	3.202750	1.490860	-0.572640
53	1	0	4.334840	0.209790	-2.809670
54	1	0	3.071990	-0.997970	-3.027860
55	1	0	2.686380	0.725260	-3.173010
56	6	0	4.668320	1.980280	-0.608540
57	6	0	4.745749	3.414060	-0.136210
58	8	0	4.548439	4.391530	-0.838760
59	8	0	5.029639	3.500820	1.179750
60	6	0	5.072439	4.830020	1.744420
61	6	0	4.781270	-1.854950	-0.886190
62	1	0	4.069630	-0.560680	0.631890
63	6	0	4.625100	-3.052540	-1.471500
64	6	0	6.156230	-1.228340	-0.816850
65	1	0	5.473750	-3.569480	-1.911310
66	1	0	3.669910	-3.561830	-1.543210
67	1	0	6.918570	-1.904440	-1.212880
68	1	0	6.209080	-0.295500	-1.388670
69	1	0	6.422840	-0.979770	0.218400
70	1	0	5.064500	1.961340	-1.627020
71	1	0	5.301780	1.362000	0.030550
72	1	0	2.840880	1.547000	0.456600
73	1	0	2.605210	2.195590	-1.162180
74	8	0	2.596230	-3.853670	1.850300
75	1	0	3.695290	-2.201450	2.185470
76	6	0	1.289400	-0.419560	2.795960
77	1	0	1.156720	0.500380	3.370440
78	1	0	0.419650	-1.066461	2.947760
79	1	0	2.185740	-0.931730	3.168440
80	6	0	-4.862840	-2.836091	0.218140
81	1	0	-5.855730	-2.552271	0.580540
82	1	0	-4.995290	-3.304561	-0.763530
83	1	0	-4.485570	-3.597521	0.905260
84	1	0	4.104989	5.322800	1.626880
85	1	0	5.305749	4.688660	2.798720
86	1	0	5.847339	5.423970	1.255250

4

Center Number	Atomic Number	Atomic Type	Coordinates (Angstroms) X	Y	Z
1	6	0	-0.894205	0.018072	-1.196963
2	6	0	0.477859	0.728825	-1.183647
3	1	0	-0.931249	-0.575704	-2.114236
4	1	0	-1.685810	0.770990	-1.281787
5	1	0	0.525200	1.487423	-0.397173
6	1	0	0.606319	1.249137	-2.140150
7	6	0	3.824304	-0.928910	-0.203831
8	6	0	2.746033	-1.916691	0.315205
9	6	0	1.461420	-1.013662	0.378597
10	6	0	1.571767	-0.323175	-0.998358
11	6	0	3.202845	-2.626410	1.569961
12	1	0	2.523225	-2.697856	-0.418939
13	8	0	1.594238	0.024003	1.373247
14	6	0	-1.675296	-3.175467	0.810668
15	6	0	-2.210496	-2.097910	-0.189416
16	6	0	-1.163625	-0.889806	0.041609
17	6	0	0.103901	-1.674341	0.464976
18	6	0	-0.200177	-2.901340	0.900973
19	1	0	-2.143640	-3.073357	1.801788
20	1	0	-1.892282	-4.195473	0.473306
21	6	0	-2.071090	-2.694433	-1.609049
22	6	0	-1.593351	0.018390	1.223814
23	1	0	0.514323	-3.620876	1.286958
24	1	0	-2.424436	-2.021107	-2.394127
25	1	0	-1.027946	-2.942032	-1.828276
26	1	0	-2.648662	-3.620331	-1.687504
27	1	0	-0.813260	0.751832	1.438655
28	1	0	-2.510703	0.568353	0.996652
29	1	0	-1.765671	-0.559267	2.137135
30	6	0	-3.711017	-1.762022	0.119107
31	6	0	-4.317758	-0.715469	-0.845449
32	6	0	-5.696624	-0.179395	-0.455932
33	6	0	-6.220691	0.947946	-1.368882
34	6	0	-5.815708	2.237205	-0.637476
35	6	0	-5.713309	1.781206	0.814331
36	8	0	-5.644028	0.431321	0.879040
37	1	0	-6.419152	-0.992186	-0.368072
38	1	0	-7.311971	0.892625	-1.446317
39	1	0	-5.808020	0.879727	-2.378464
40	1	0	-4.794933	2.526644	-0.925309
41	6	0	-6.738933	3.441539	-0.829633
42	1	0	-3.738478	-1.333542	1.126382
43	1	0	-4.430985	-1.154023	-1.845341
44	1	0	-3.641667	0.137569	-0.959785
45	8	0	-5.679484	2.469657	1.813663
46	1	0	-6.406320	4.285188	-0.218927
47	1	0	-6.741108	3.754572	-1.878120
48	1	0	-7.767313	3.196371	-0.543795
49	1	0	1.320720	-1.126388	-1.704788
50	6	0	3.086526	-0.010010	-1.274225
51	6	0	3.390280	-0.460162	-2.721198

52	6	0	3.471867	1.493738	-1.200538
53	1	0	4.430663	-0.271242	-3.003126
54	1	0	3.198617	-1.528685	-2.858021
55	1	0	2.751351	0.088735	-3.423271
56	6	0	3.442496	2.204150	0.156785
57	6	0	3.617390	3.702142	0.036125
58	8	0	3.516940	4.358840	-0.986000
59	8	0	3.884780	4.258021	1.238322
60	6	0	4.023861	5.694916	1.268922
61	6	0	5.138165	-1.564405	-0.633053
62	1	0	4.064664	-0.284753	0.651011
63	6	0	5.270307	-2.862158	-0.948029
64	6	0	6.338435	-0.644183	-0.648780
65	1	0	6.236105	-3.269636	-1.234823
66	1	0	4.440679	-3.561450	-0.936534
67	1	0	7.248193	-1.179208	-0.934050
68	1	0	6.202458	0.187474	-1.349647
69	1	0	6.498996	-0.196635	0.340507
70	1	0	4.220469	1.838223	0.834247
71	1	0	2.498321	2.027352	0.682113
72	1	0	2.812767	2.036517	-1.885968
73	1	0	4.477325	1.610706	-1.621624
74	8	0	2.863032	-3.752716	1.892607
75	1	0	3.922513	-2.070668	2.205006
76	6	0	1.476442	-0.336163	2.746480
77	1	0	1.288752	0.590824	3.293715
78	1	0	0.647866	-1.029879	2.920286
79	1	0	2.403139	-0.780949	3.130598
80	6	0	-4.601003	-3.023829	0.159051
81	1	0	-5.596170	-2.785228	0.546327
82	1	0	-4.733352	-3.465338	-0.835186
83	1	0	-4.187278	-3.793498	0.815145
84	1	0	4.852296	6.010208	0.630852
85	1	0	3.101278	6.173353	0.932968
86	1	0	4.226664	5.943360	2.309808

5

Center Number	Atomic Number	Atomic Type	Coordinates (Angstroms) X	Y	Z
1	6	0	-1.022274	0.154094	-1.108582
2	6	0	0.360775	0.825180	-0.967436
3	1	0	-1.030667	-0.361871	-2.072483
4	1	0	-1.791852	0.932241	-1.164120
5	1	0	0.390017	1.490572	-0.099789
6	1	0	0.539931	1.445325	-1.854394
7	6	0	3.635614	-1.126056	-0.178350
8	6	0	2.477340	-2.060657	0.280855
9	6	0	1.247417	-1.099704	0.431220
10	6	0	1.433276	-0.258764	-0.851191
11	6	0	2.899833	-2.905413	1.461288
12	1	0	2.194675	-2.761884	-0.507975
13	8	0	1.406060	-0.190980	1.541659
14	6	0	-1.977581	-3.169782	0.578944
15	6	0	-2.451628	-1.981665	-0.321680
16	6	0	-1.363738	-0.842796	0.038011
17	6	0	-0.137903	-1.710074	0.413994
18	6	0	-0.496165	-2.960642	0.723904
19	1	0	-2.462984	-3.145190	1.566686
20	1	0	-2.224575	-4.144022	0.141410
21	6	0	-2.311460	-2.444703	-1.790167
22	6	0	-1.787952	-0.021901	1.283967
23	1	0	0.182519	-3.739738	1.055272
24	1	0	-2.618315	-1.681988	-2.510572
25	1	0	-1.276934	-2.720431	-2.017009
26	1	0	-2.929270	-3.330073	-1.967250
27	1	0	-0.976567	0.640189	1.592700
28	1	0	-2.664138	0.599325	1.077471
29	1	0	-2.027557	-0.668123	2.134133
30	6	0	-3.942587	-1.613272	-0.006021
31	6	0	-4.495815	-0.476781	-0.899386
32	6	0	-5.801062	0.161748	-0.420642
33	6	0	-6.282755	1.344684	-1.285310
34	6	0	-5.719506	2.580981	-0.567735
35	6	0	-5.577019	2.095623	0.871007
36	8	0	-5.618887	0.743350	0.916290
37	1	0	-6.581950	-0.590666	-0.297723
38	1	0	-7.377480	1.385623	-1.291112
39	1	0	-5.943616	1.257561	-2.320462
40	1	0	-4.693900	2.777840	-0.911609
41	6	0	-6.535338	3.868274	-0.695459
42	1	0	-3.969642	-1.260226	1.029833
43	1	0	-4.692191	-0.863160	-1.908035
44	1	0	-3.753105	0.318311	-1.016205
45	8	0	-5.429437	2.762590	1.874369
46	1	0	-6.093742	4.667275	-0.093797
47	1	0	-6.564334	4.197839	-1.738493
48	1	0	-7.565327	3.714263	-0.356787
49	1	0	1.238839	-0.967938	-1.668709
50	6	0	2.952299	0.092574	-0.964741
51	6	0	3.335164	0.155691	-2.455336

52	6	0	3.237620	1.460350	-0.282950
53	1	0	4.396947	0.364891	-2.612101
54	1	0	3.112751	-0.786670	-2.963720
55	1	0	2.766456	0.950187	-2.952420
56	6	0	4.710597	1.926466	-0.249768
57	6	0	4.819157	3.259973	0.453246
58	8	0	4.938077	3.407899	1.657993
59	8	0	4.737304	4.294630	-0.409259
60	6	0	4.778893	5.619276	0.165798
61	6	0	4.772804	-1.868161	-0.880977
62	1	0	4.066808	-0.697779	0.736331
63	6	0	4.603151	-3.003949	-1.575381
64	6	0	6.157909	-1.276776	-0.739284
65	1	0	5.448312	-3.494813	-2.050355
66	1	0	3.640078	-3.486542	-1.704165
67	1	0	6.913039	-1.928498	-1.186837
68	1	0	6.234722	-0.296855	-1.223160
69	1	0	6.415881	-1.128824	0.317165
70	1	0	5.117782	2.023507	-1.259036
71	1	0	5.324026	1.221765	0.315139
72	1	0	2.861820	1.422283	0.741747
73	1	0	2.656308	2.225674	-0.810586
74	8	0	2.531564	-4.050889	1.661482
75	1	0	3.626412	-2.435518	2.155847
76	6	0	1.215071	-0.693602	2.860652
77	1	0	0.312031	-1.306782	2.942053
78	1	0	2.077090	-1.280138	3.202983
79	1	0	1.116001	0.180228	3.508901
80	6	0	-4.880921	-2.838071	-0.074529
81	1	0	-5.886555	-2.577518	0.269851
82	1	0	-4.975444	-3.226127	-1.094820
83	1	0	-4.532953	-3.653749	0.564150
84	1	0	4.701128	6.303182	-0.678186
85	1	0	3.942633	5.760263	0.853903
86	1	0	5.720141	5.771104	0.698289

6

Center Number	Atomic Number	Atomic Type	Coordinates (Angstroms) X	Y	Z
1	6	0	-0.821658	0.153800	-1.056135
2	6	0	0.548436	0.821711	-0.807273
3	1	0	-0.789652	-0.266949	-2.064965
4	1	0	-1.597848	0.927398	-1.066044
5	1	0	0.541750	1.412726	0.113893
6	1	0	0.739306	1.512210	-1.636354
7	6	0	3.802001	-1.130081	0.019027
8	6	0	2.658109	-2.149171	0.249058
9	6	0	1.403069	-1.222257	0.430545
10	6	0	1.636169	-0.253362	-0.750375
11	6	0	3.014909	-3.129044	1.344279
12	1	0	2.452715	-2.745913	-0.644988
13	8	0	1.499293	-0.418798	1.627129
14	6	0	-1.795398	-3.328371	0.249710
15	6	0	-2.256563	-2.056458	-0.535816
16	6	0	-1.192564	-0.953111	-0.026709
17	6	0	0.030063	-1.843205	0.298788
18	6	0	-0.322022	-3.122936	0.463060
19	1	0	-2.310409	-3.411327	1.218980
20	1	0	-2.016844	-4.253361	-0.295134
21	6	0	-2.066343	-2.363304	-2.039304
22	6	0	-1.665889	-0.265143	1.280877
23	1	0	0.355027	-3.926671	0.732060
24	1	0	-2.361177	-1.533046	-2.686188
25	1	0	-1.021565	-2.602488	-2.260711
26	1	0	-2.665306	-3.232414	-2.327627
27	1	0	-0.876150	0.376789	1.676209
28	1	0	-2.548086	0.358536	1.110973
29	1	0	-1.916624	-0.995730	2.056180
30	6	0	-3.760809	-1.738104	-0.227930
31	6	0	-4.298277	-0.507915	-0.997401
32	6	0	-5.666735	0.005906	-0.546218
33	6	0	-6.123471	1.299468	-1.250832
34	6	0	-5.676671	2.417470	-0.296389
35	6	0	-5.639936	1.708005	1.053327
36	8	0	-5.628210	0.366134	0.877739
37	1	0	-6.421594	-0.778517	-0.619569
38	1	0	-7.214594	1.309362	-1.348515
39	1	0	-5.695236	1.392185	-2.251960
40	1	0	-4.634007	2.694703	-0.508377
41	6	0	-6.531180	3.685856	-0.292085
42	1	0	-3.823075	-1.507634	0.840749
43	1	0	-4.397769	-0.752382	-2.062922
44	1	0	-3.586924	0.321720	-0.937304
45	8	0	-5.609128	2.206296	2.159805
46	1	0	-6.178108	4.387612	0.468343
47	1	0	-6.481767	4.180041	-1.267102
48	1	0	-7.579702	3.451124	-0.080438
49	1	0	1.483174	-0.878789	-1.641817
50	6	0	3.160103	0.108246	-0.767782
51	6	0	3.624592	0.183248	-2.235081

52	6	0	3.549077	1.403516	0.003315
53	1	0	4.666152	0.508499	-2.326631
54	1	0	3.538346	-0.790965	-2.724922
55	1	0	3.006599	0.890844	-2.798759
56	6	0	3.280832	2.747585	-0.686887
57	6	0	3.825830	3.922435	0.099150
58	8	0	4.652301	3.865575	0.992543
59	8	0	3.293264	5.085893	-0.333267
60	6	0	3.772779	6.290595	0.303146
61	6	0	5.089473	-1.714370	-0.544563
62	1	0	4.056394	-0.742719	1.014817
63	6	0	5.150094	-2.877981	-1.210667
64	6	0	6.349622	-0.922205	-0.273594
65	1	0	6.098717	-3.258723	-1.579824
66	1	0	4.278091	-3.491163	-1.412817
67	1	0	6.491875	-0.774507	0.804551
68	1	0	7.232338	-1.431181	-0.670204
69	1	0	6.307786	0.077085	-0.721714
70	1	0	2.219174	2.930947	-0.864372
71	1	0	3.763697	2.793064	-1.671405
72	1	0	4.622882	1.359091	0.211778
73	1	0	3.049940	1.390186	0.976121
74	8	0	2.646884	-4.291410	1.384052
75	1	0	3.690544	-2.743770	2.135131
76	6	0	1.290228	-1.054573	2.884538
77	1	0	0.448443	-1.754072	2.857847
78	1	0	2.188291	-1.586752	3.223589
79	1	0	1.072603	-0.258967	3.601208
80	6	0	-4.681292	-2.954813	-0.468294
81	1	0	-5.689282	-2.760600	-0.089595
82	1	0	-4.772621	-3.196954	-1.533148
83	1	0	-4.319532	-3.846752	0.048906
84	1	0	4.849449	6.396215	0.153274
85	1	0	3.236718	7.105879	-0.180724
86	1	0	3.553141	6.267983	1.372613

7

Center Number	Atomic Number	Atomic Type	Coordinates (Angstroms) X	Y	Z
1	6	0	-0.857085	0.070228	-1.175256
2	6	0	0.501599	0.800828	-1.083403
3	1	0	-0.862246	-0.466932	-2.127364
4	1	0	-1.659745	0.813767	-1.232772
5	1	0	0.516559	1.507947	-0.249416
6	1	0	0.642336	1.383976	-2.001455
7	6	0	3.853824	-0.881537	-0.146654
8	6	0	2.776370	-1.913762	0.280836
9	6	0	1.479947	-1.030987	0.380964
10	6	0	1.610616	-0.242149	-0.940416
11	6	0	3.217764	-2.701611	1.493754
12	1	0	2.575870	-2.646248	-0.508049
13	8	0	1.581626	-0.065054	1.447273
14	6	0	-1.635636	-3.254115	0.603970
15	6	0	-2.169412	-2.116405	-0.327778
16	6	0	-1.138663	-0.916072	-0.001903
17	6	0	0.130029	-1.712669	0.392045
18	6	0	-0.165902	-2.970042	0.737874
19	1	0	-2.120109	-3.225539	1.592067
20	1	0	-1.834797	-4.251094	0.194617
21	6	0	-2.007277	-2.613681	-1.782793
22	6	0	-1.599247	-0.087778	1.226694
23	1	0	0.550772	-3.705853	1.087600
24	1	0	-2.356817	-1.891054	-2.524593
25	1	0	-0.959870	-2.839430	-2.005638
26	1	0	-2.576236	-3.536165	-1.931956
27	1	0	-0.830221	0.636806	1.501640
28	1	0	-2.517077	0.467837	1.015995
29	1	0	-1.783978	-0.721737	2.099392
30	6	0	-3.677359	-1.817107	-0.016681
31	6	0	-4.285325	-0.714121	-0.915700
32	6	0	-5.670118	-0.213306	-0.500629
33	6	0	-6.191301	0.974379	-1.335338
34	6	0	-5.792528	2.209198	-0.512531
35	6	0	-5.702457	1.652608	0.904517
36	8	0	-5.631388	0.301461	0.874863
37	1	0	-6.390024	-1.032818	-0.476903
38	1	0	-7.282016	0.922889	-1.422652
39	1	0	-5.772733	0.977398	-2.344807
40	1	0	-4.768955	2.516475	-0.770561
41	6	0	-6.713571	3.424996	-0.627425
42	1	0	-3.721642	-1.457566	1.016415
43	1	0	-4.389362	-1.088228	-1.942477
44	1	0	-3.614877	0.149381	-0.970737
45	8	0	-5.678436	2.269243	1.949823
46	1	0	-6.387669	4.222071	0.046251
47	1	0	-6.704620	3.812908	-1.650530
48	1	0	-7.744957	3.160638	-0.371049
49	1	0	1.396607	-0.997818	-1.709044
50	6	0	3.123671	0.116363	-1.148670
51	6	0	3.474467	-0.190214	-2.622185

52	6	0	3.493354	1.612189	-0.928975
53	1	0	4.516690	0.047846	-2.854888
54	1	0	3.314179	-1.245931	-2.859943
55	1	0	2.839729	0.405295	-3.288973
56	6	0	3.372828	2.224555	0.484903
57	6	0	3.781893	3.678781	0.448766
58	8	0	4.931815	4.085091	0.491383
59	8	0	2.718770	4.502260	0.331020
60	6	0	3.003622	5.915514	0.241459
61	6	0	5.177655	-1.473559	-0.608072
62	1	0	4.081085	-0.305349	0.758823
63	6	0	5.321126	-2.741406	-1.023754
64	6	0	6.371698	-0.548193	-0.535111
65	1	0	6.292916	-3.120371	-1.328867
66	1	0	4.495293	-3.443102	-1.079560
67	1	0	7.290847	-1.058142	-0.835708
68	1	0	6.245750	0.327802	-1.181692
69	1	0	6.507664	-0.165500	0.484354
70	1	0	4.049113	1.723625	1.182026
71	1	0	2.358564	2.129844	0.868800
72	1	0	2.872117	2.203201	-1.613352
73	1	0	4.526214	1.757878	-1.264823
74	8	0	2.890197	-3.852692	1.729797
75	1	0	3.913613	-2.179758	2.181863
76	6	0	1.434256	-0.519563	2.789215
77	1	0	1.232012	0.367482	3.394433
78	1	0	0.604109	-1.224701	2.897077
79	1	0	2.353112	-0.988763	3.163186
80	6	0	-4.553606	-3.088027	-0.074505
81	1	0	-5.558025	-2.885302	0.309212
82	1	0	-4.663192	-3.465079	-1.097546
83	1	0	-4.142859	-3.894542	0.537816
84	1	0	3.524137	6.254641	1.139597
85	1	0	3.617953	6.123485	-0.637314
86	1	0	2.032728	6.401334	0.154926

8

Center Number	Atomic Number	Atomic Type	Coordinates (Angstroms) X	Y	Z
1	6	0	-1.094073	0.178073	-1.129221
2	6	0	0.299581	0.835828	-1.029732
3	1	0	-1.118471	-0.377157	-2.070886
4	1	0	-1.853970	0.963523	-1.210500
5	1	0	0.348512	1.539391	-0.193569
6	1	0	0.479484	1.413108	-1.944902
7	6	0	3.554852	-1.112722	-0.170800
8	6	0	2.390157	-2.022273	0.332547
9	6	0	1.173245	-1.037046	0.444439
10	6	0	1.357752	-0.256821	-0.876739
11	6	0	2.807949	-2.803437	1.555129
12	1	0	2.107190	-2.760530	-0.423183
13	8	0	1.353937	-0.080910	1.511156
14	6	0	-2.072948	-3.059372	0.710058
15	6	0	-2.539746	-1.908304	-0.241442
16	6	0	-1.438027	-0.766207	0.060941
17	6	0	-0.218593	-1.630012	0.465793
18	6	0	-0.588132	-2.861171	0.834577
19	1	0	-2.550817	-2.982318	1.698852
20	1	0	-2.334359	-4.049792	0.320038
21	6	0	-2.413030	-2.438332	-1.688137
22	6	0	-1.843454	0.111598	1.274004
23	1	0	0.083311	-3.633649	1.195361
24	1	0	-2.720604	-1.707206	-2.440321
25	1	0	-1.381869	-2.730482	-1.909527
26	1	0	-3.037786	-3.326700	-1.820674
27	1	0	-1.025618	0.782975	1.543501
28	1	0	-2.718273	0.728359	1.049949
29	1	0	-2.077284	-0.494574	2.154649
30	6	0	-4.024466	-1.510823	0.066996
31	6	0	-4.570164	-0.402146	-0.864498
32	6	0	-5.879937	0.248484	-0.415012
33	6	0	-6.345327	1.419665	-1.304357
34	6	0	-5.794024	2.665995	-0.594704
35	6	0	-5.672831	2.200402	0.852444
36	8	0	-5.715612	0.848887	0.915728
37	1	0	-6.665146	-0.499560	-0.293029
38	1	0	-7.439755	1.460913	-1.329951
39	1	0	-5.988198	1.317835	-2.332111
40	1	0	-4.763706	2.859963	-0.925674
41	6	0	-6.609741	3.950192	-0.751601
42	1	0	-4.040997	-1.118406	1.088814
43	1	0	-4.758320	-0.817305	-1.863222
44	1	0	-3.826589	0.389452	-0.998161
45	8	0	-5.540381	2.880870	1.848805
46	1	0	-6.177598	4.758001	-0.154905
47	1	0	-6.624494	4.265235	-1.799395
48	1	0	-7.644166	3.799255	-0.425209
49	1	0	1.140608	-1.000682	-1.656991
50	6	0	2.878576	0.061484	-1.025072
51	6	0	3.259869	0.067401	-2.519679

52	6	0	3.223211	1.449793	-0.415662
53	1	0	4.343253	0.072572	-2.668726
54	1	0	2.858139	-0.800287	-3.049754
55	1	0	2.851827	0.961158	-3.006026
56	6	0	4.694364	1.872853	-0.516312
57	6	0	4.948923	3.249385	0.060395
58	8	0	4.114337	4.122845	0.219041
59	8	0	6.254061	3.419511	0.364168
60	6	0	6.634547	4.718058	0.869278
61	6	0	4.692014	-1.911383	-0.800646
62	1	0	3.974709	-0.631645	0.719634
63	6	0	5.963281	-1.697268	-0.426784
64	6	0	4.367364	-3.004483	-1.797421
65	1	0	6.785080	-2.262116	-0.859273
66	1	0	6.225658	-0.959001	0.326337
67	1	0	5.274672	-3.357514	-2.294912
68	1	0	3.909822	-3.867171	-1.297289
69	1	0	3.662332	-2.676624	-2.566227
70	1	0	5.027597	1.916996	-1.561242
71	1	0	5.366031	1.168681	-0.018985
72	1	0	2.917525	1.461910	0.633105
73	1	0	2.618864	2.209066	-0.922865
74	8	0	2.497170	-3.962868	1.775084
75	1	0	3.478669	-2.274200	2.261672
76	6	0	1.137467	-0.506444	2.853579
77	1	0	1.016150	0.402944	3.447103
78	1	0	0.238039	-1.121853	2.952179
79	1	0	1.996079	-1.063797	3.248717
80	6	0	-4.977166	-2.726475	0.052084
81	1	0	-5.972576	-2.444809	0.409114
82	1	0	-5.096676	-3.142806	-0.954430
83	1	0	-4.625321	-3.527605	0.706812
84	1	0	7.707748	4.656734	1.044446
85	1	0	6.409082	5.492796	0.133190
86	1	0	6.105162	4.933291	1.799944

9

Center Number	Atomic Number	Atomic Type	Coordinates (Angstroms) X	Y	Z
1	6	0	-1.071677	0.123894	-1.152169
2	6	0	0.307833	0.816322	-1.107282
3	1	0	-1.099273	-0.471334	-2.069107
4	1	0	-1.851183	0.887540	-1.252551
5	1	0	0.356155	1.553690	-0.300753
6	1	0	0.456691	1.359305	-2.048527
7	6	0	3.622345	-1.028011	-0.237654
8	6	0	2.487228	-1.936398	0.331074
9	6	0	1.251788	-0.972854	0.426371
10	6	0	1.392516	-0.246589	-0.930224
11	6	0	2.946978	-2.655494	1.576714
12	1	0	2.202805	-2.712813	-0.384913
13	8	0	1.431262	0.032765	1.447184
14	6	0	-1.942790	-3.050950	0.843467
15	6	0	-2.452151	-1.954284	-0.149118
16	6	0	-1.371941	-0.776166	0.083366
17	6	0	-0.125676	-1.594127	0.501519
18	6	0	-0.460799	-2.815760	0.930375
19	1	0	-2.403841	-2.940438	1.837011
20	1	0	-2.188875	-4.063077	0.502413
21	6	0	-2.335512	-2.545194	-1.573025
22	6	0	-1.775169	0.142732	1.266386
23	1	0	0.233807	-3.557710	1.310885
24	1	0	-2.670259	-1.854839	-2.351556
25	1	0	-1.301210	-2.823437	-1.797468
26	1	0	-2.941533	-3.452316	-1.657079
27	1	0	-0.970744	0.846914	1.489277
28	1	0	-2.671254	0.725582	1.036225
29	1	0	-1.973325	-0.431416	2.176678
30	6	0	-3.940833	-1.576986	0.166641
31	6	0	-4.527776	-0.526879	-0.806697
32	6	0	-5.855342	0.099698	-0.376196
33	6	0	-6.368754	1.209658	-1.316053
34	6	0	-5.856628	2.507563	-0.672300
35	6	0	-5.704263	2.116196	0.793975
36	8	0	-5.700449	0.768960	0.922370
37	1	0	-6.612968	-0.668029	-0.210454
38	1	0	-7.464073	1.211360	-1.333116
39	1	0	-6.016344	1.070725	-2.341126
40	1	0	-4.837414	2.722763	-1.023647
41	6	0	-6.720729	3.752207	-0.880894
42	1	0	-3.950140	-1.136669	1.169055
43	1	0	-4.708239	-0.989805	-1.785552
44	1	0	-3.810545	0.281679	-0.978395
45	8	0	-5.583739	2.847931	1.754994
46	1	0	-6.313643	4.603021	-0.327922
47	1	0	-6.756480	4.015863	-1.942262
48	1	0	-7.745937	3.579064	-0.536888
49	1	0	1.176291	-1.027086	-1.673977
50	6	0	2.903129	0.094513	-1.124889
51	6	0	3.253390	0.050228	-2.625731

52	6	0	3.231905	1.512535	-0.577412
53	1	0	4.333038	0.073342	-2.797422
54	1	0	2.860271	-0.846369	-3.112098
55	1	0	2.816262	0.915194	-3.137988
56	6	0	4.697305	1.972715	-0.757775
57	6	0	4.882846	3.356948	-0.181557
58	8	0	4.542349	4.392204	-0.729866
59	8	0	5.448832	3.327745	1.043132
60	6	0	5.625378	4.600934	1.702622
61	6	0	4.760636	-1.831543	-0.859362
62	1	0	4.053480	-0.501436	0.620852
63	6	0	6.034603	-1.578672	-0.520405
64	6	0	4.436733	-2.969715	-1.804328
65	1	0	6.858633	-2.145401	-0.946083
66	1	0	6.296536	-0.805085	0.196589
67	1	0	5.340361	-3.326281	-2.305931
68	1	0	4.005548	-3.819657	-1.260799
69	1	0	3.710363	-2.685905	-2.570736
70	1	0	4.954369	2.030161	-1.818630
71	1	0	5.389168	1.283780	-0.270153
72	1	0	2.969573	1.550236	0.482900
73	1	0	2.591561	2.238245	-1.091373
74	8	0	2.663901	-3.809906	1.852808
75	1	0	3.622029	-2.083907	2.244941
76	6	0	1.257788	-0.340978	2.811176
77	1	0	2.145575	-0.846663	3.210991
78	1	0	1.112413	0.587978	3.367904
79	1	0	0.385232	-0.985465	2.955044
80	6	0	-4.863922	-2.813878	0.226045
81	1	0	-5.858433	-2.539416	0.590949
82	1	0	-4.993583	-3.277914	-0.758133
83	1	0	-4.480620	-3.576318	0.908638
84	1	0	6.095725	4.369275	2.657168
85	1	0	6.267660	5.250984	1.104818
86	1	0	4.658458	5.084531	1.857350

10

Center Number	Atomic Number	Atomic Type	Coordinates (Angstroms) X	Y	Z
1	6	0	-1.052227	0.069587	-1.179976
2	6	0	0.325528	0.764195	-1.130232
3	1	0	-1.073648	-0.531315	-2.093365
4	1	0	-1.831601	0.832147	-1.289791
5	1	0	0.366250	1.504297	-0.325975
6	1	0	0.477348	1.304972	-2.072342
7	6	0	3.638430	-1.073176	-0.238333
8	6	0	2.501382	-1.982617	0.313365
9	6	0	1.263103	-1.026000	0.408727
10	6	0	1.412969	-0.295035	-0.944013
11	6	0	2.954749	-2.727648	1.548442
12	1	0	2.213817	-2.747239	-0.412456
13	8	0	1.434145	-0.024574	1.435437
14	6	0	-1.939811	-3.095100	0.819102
15	6	0	-2.446302	-1.994325	-0.169992
16	6	0	-1.357850	-0.823276	0.058883
17	6	0	-0.115516	-1.647041	0.476697
18	6	0	-0.456377	-2.867761	0.903980
19	1	0	-2.397588	-2.983952	1.814120
20	1	0	-2.192312	-4.105378	0.477101
21	6	0	-2.340521	-2.584432	-1.595062
22	6	0	-1.753360	0.100995	1.240334
23	1	0	0.235455	-3.612228	1.284064
24	1	0	-2.673372	-1.890453	-2.371255
25	1	0	-1.309382	-2.870424	-1.824331
26	1	0	-2.953769	-3.486851	-1.677685
27	1	0	-0.942455	0.797609	1.462930
28	1	0	-2.644798	0.690956	1.010136
29	1	0	-1.955930	-0.470909	2.151147
30	6	0	-3.930767	-1.606370	0.152584
31	6	0	-4.515107	-0.553430	-0.819420
32	6	0	-5.825045	0.098690	-0.373195
33	6	0	-6.335135	1.208711	-1.314828
34	6	0	-5.789996	2.503448	-0.692079
35	6	0	-5.619744	2.123079	0.775081
36	8	0	-5.637903	0.777295	0.916466
37	1	0	-6.592422	-0.654712	-0.187553
38	1	0	-7.430391	1.229316	-1.313730
39	1	0	-6.002495	1.054304	-2.344272
40	1	0	-4.773283	2.697992	-1.062473
41	6	0	-6.636193	3.760827	-0.897787
42	1	0	-3.931843	-1.164569	1.154347
43	1	0	-4.717908	-1.020184	-1.792108
44	1	0	-3.787472	0.241798	-1.008036
45	8	0	-5.469828	2.861616	1.726755
46	1	0	-6.205163	4.609611	-0.360021
47	1	0	-6.685837	4.015184	-1.960863
48	1	0	-7.658081	3.608577	-0.534518
49	1	0	1.211045	-1.073327	-1.694068
50	6	0	2.925405	0.061439	-1.118166
51	6	0	3.281039	-0.007225	-2.614926

52	6	0	3.202739	1.490861	-0.572642
53	1	0	4.334826	0.209799	-2.809679
54	1	0	3.071982	-0.997961	-3.027870
55	1	0	2.686365	0.725264	-3.173013
56	6	0	4.668311	1.980283	-0.608537
57	6	0	4.745743	3.414059	-0.136202
58	8	0	4.548437	4.391529	-0.838751
59	8	0	5.029626	3.500819	1.179753
60	6	0	5.072427	4.830016	1.744426
61	6	0	4.781262	-1.854953	-0.886208
62	1	0	4.069633	-0.560678	0.631881
63	6	0	4.625094	-3.052540	-1.471509
64	6	0	6.156221	-1.228333	-0.816868
65	1	0	5.473742	-3.569474	-1.911326
66	1	0	3.669903	-3.561829	-1.543211
67	1	0	6.918569	-1.904433	-1.212907
68	1	0	6.209069	-0.295492	-1.388681
69	1	0	6.422837	-0.979771	0.218386
70	1	0	5.064492	1.961348	-1.627019
71	1	0	5.301774	1.361996	0.030559
72	1	0	2.840869	1.546997	0.456606
73	1	0	2.605204	2.195595	-1.162174
74	8	0	2.596228	-3.853677	1.850294
75	1	0	3.695298	-2.201452	2.185460
76	6	0	1.289401	-0.419571	2.795956
77	1	0	1.156725	0.500370	3.370442
78	1	0	0.419649	-1.066466	2.947764
79	1	0	2.185744	-0.931739	3.168432
80	6	0	-4.862842	-2.836089	0.218151
81	1	0	-5.855733	-2.552274	0.580550
82	1	0	-4.995283	-3.304570	-0.763515
83	1	0	-4.485569	-3.597520	0.905285
84	1	0	4.104971	5.322787	1.626894
85	1	0	5.305745	4.688657	2.798729
86	1	0	5.847317	5.423974	1.255251

11

Center Number	Atomic Number	Atomic Type	Coordinates (Angstroms) X	Y	Z
1	6	0	-1.027486	0.161272	-1.106422
2	6	0	0.352299	0.842672	-0.978677
3	1	0	-1.035573	-0.366707	-2.063826
4	1	0	-1.802343	0.933554	-1.169206
5	1	0	0.381549	1.519496	-0.119777
6	1	0	0.525537	1.451844	-1.874290
7	6	0	3.640992	-1.073925	-0.166865
8	6	0	2.489959	-2.015611	0.308357
9	6	0	1.254661	-1.056623	0.443422
10	6	0	1.431048	-0.233476	-0.852543
11	6	0	2.918688	-2.824899	1.509113
12	1	0	2.221686	-2.736668	-0.468957
13	8	0	1.411724	-0.128835	1.538140
14	6	0	-1.953496	-3.147578	0.628893
15	6	0	-2.438629	-1.976075	-0.287699
16	6	0	-1.359278	-0.823468	0.053782
17	6	0	-0.125999	-1.675950	0.439086
18	6	0	-0.473493	-2.924804	0.767375
19	1	0	-2.436911	-3.112151	1.617266
20	1	0	-2.193826	-4.129871	0.206076
21	6	0	-2.297616	-2.458213	-1.749837
22	6	0	-1.788104	0.011405	1.288865
23	1	0	0.211426	-3.694790	1.107703
24	1	0	-2.613097	-1.708337	-2.479933
25	1	0	-1.261224	-2.727846	-1.975439
26	1	0	-2.907923	-3.351369	-1.913286
27	1	0	-0.983843	0.688362	1.583756
28	1	0	-2.672160	0.619002	1.076230
29	1	0	-2.017154	-0.623859	2.150065
30	6	0	-3.931928	-1.615675	0.026247
31	6	0	-4.496577	-0.495067	-0.880014
32	6	0	-5.806312	0.137671	-0.405795
33	6	0	-6.303256	1.303326	-1.285217
34	6	0	-5.752889	2.555910	-0.586038
35	6	0	-5.598470	2.091555	0.858433
36	8	0	-5.624977	0.739683	0.922161
37	1	0	-6.579229	-0.620616	-0.269289
38	1	0	-7.398371	1.331545	-1.289094
39	1	0	-5.965351	1.205972	-2.319865
40	1	0	-4.731380	2.761635	-0.936730
41	6	0	-6.586044	3.830701	-0.727359
42	1	0	-3.959759	-1.250093	1.057708
43	1	0	-4.691793	-0.895694	-1.883298
44	1	0	-3.761048	0.304966	-1.008436
45	8	0	-5.453679	2.773553	1.852057
46	1	0	-6.152169	4.643607	-0.138864
47	1	0	-6.624132	4.145378	-1.774682
48	1	0	-7.612358	3.667927	-0.381701
49	1	0	1.233880	-0.956598	-1.657216
50	6	0	2.946718	0.117563	-0.980829
51	6	0	3.336897	0.190447	-2.470578

52	6	0	3.260913	1.485569	-0.311622
53	1	0	4.420804	0.225070	-2.611251
54	1	0	2.956932	-0.664967	-3.035253
55	1	0	2.913155	1.093439	-2.925423
56	6	0	4.730690	1.957055	-0.413036
57	6	0	4.924694	3.236749	0.366156
58	8	0	5.215945	3.300219	1.548980
59	8	0	4.703474	4.329999	-0.394161
60	6	0	4.809471	5.609129	0.268351
61	6	0	4.786545	-1.836700	-0.825946
62	1	0	4.058505	-0.616344	0.736866
63	6	0	6.055882	-1.616985	-0.448920
64	6	0	4.473520	-2.903524	-1.854597
65	1	0	6.883643	-2.157469	-0.900768
66	1	0	6.310454	-0.899625	0.326719
67	1	0	5.384704	-3.231540	-2.361998
68	1	0	4.025969	-3.785673	-1.379998
69	1	0	3.764659	-2.562259	-2.614164
70	1	0	5.014411	2.127561	-1.454170
71	1	0	5.406618	1.214610	0.015822
72	1	0	2.973785	1.435909	0.741141
73	1	0	2.630654	2.250134	-0.780645
74	8	0	2.623380	-3.994212	1.695396
75	1	0	3.582346	-2.306923	2.230354
76	6	0	1.200339	-0.598450	2.866817
77	1	0	1.076249	0.291180	3.488839
78	1	0	0.303998	-1.221066	2.947064
79	1	0	2.062459	-1.163363	3.242549
80	6	0	-4.860474	-2.848835	-0.024779
81	1	0	-5.867022	-2.592290	0.319902
82	1	0	-4.955259	-3.249542	-1.040133
83	1	0	-4.504271	-3.654251	0.622325
84	1	0	4.596469	6.351259	-0.499772
85	1	0	4.082846	5.676491	1.080886
86	1	0	5.816923	5.746725	0.666856

12

Center Number	Atomic Number	Atomic Type	Coordinates (Angstroms) X	Y	Z
1	6	0	-0.880920	0.167397	-1.101454
2	6	0	0.501052	0.825054	-0.893915
3	1	0	-0.863979	-0.297486	-2.090990
4	1	0	-1.645131	0.951937	-1.139285
5	1	0	0.512457	1.459000	-0.002052
6	1	0	0.693796	1.473049	-1.756082
7	6	0	3.731114	-1.135014	0.001696
8	6	0	2.575219	-2.128956	0.276465
9	6	0	1.334729	-1.176779	0.425307
10	6	0	1.573159	-0.262700	-0.798304
11	6	0	2.923770	-3.068060	1.409356
12	1	0	2.358162	-2.758788	-0.591811
13	8	0	1.454686	-0.323094	1.584673
14	6	0	-1.893927	-3.242852	0.353575
15	6	0	-2.344188	-1.996220	-0.477805
16	6	0	-1.260030	-0.888112	-0.022902
17	6	0	-0.048159	-1.781844	0.330743
18	6	0	-0.416691	-3.048623	0.549902
19	1	0	-2.404416	-3.283235	1.327988
20	1	0	-2.129967	-4.185105	-0.154440
21	6	0	-2.173601	-2.366239	-1.969451
22	6	0	-1.712892	-0.138728	1.257519
23	1	0	0.251031	-3.850164	0.847299
24	1	0	-2.460795	-1.557689	-2.646626
25	1	0	-1.135357	-2.632526	-2.190750
26	1	0	-2.790477	-3.235489	-2.216097
27	1	0	-0.909543	0.504632	1.621414
28	1	0	-2.584146	0.494095	1.066556
29	1	0	-1.971840	-0.832402	2.063375
30	6	0	-3.841055	-1.644760	-0.169533
31	6	0	-4.366419	-0.433352	-0.976220
32	6	0	-5.727972	0.110354	-0.539030
33	6	0	-6.163815	1.394951	-1.272933
34	6	0	-5.707206	2.526912	-0.339671
35	6	0	-5.684847	1.845497	1.024548
36	8	0	-5.687527	0.500060	0.876938
37	1	0	-6.494021	-0.664471	-0.597694
38	1	0	-7.254130	1.417210	-1.377304
39	1	0	-5.728625	1.460640	-2.273191
40	1	0	-4.660311	2.786846	-0.552216
41	6	0	-6.545666	3.805684	-0.364979
42	1	0	-3.891187	-1.376835	0.890932
43	1	0	-4.471197	-0.709253	-2.033623
44	1	0	-3.645495	0.389423	-0.943457
45	8	0	-5.653713	2.366093	2.120614
46	1	0	-6.186935	4.517914	0.382958
47	1	0	-6.485659	4.279665	-1.349370
48	1	0	-7.598009	3.588537	-0.153475
49	1	0	1.405900	-0.922776	-1.661651
50	6	0	3.102270	0.074506	-0.838386
51	6	0	3.564593	0.078038	-2.307599

52	6	0	3.508232	1.400068	-0.127654
53	1	0	4.615089	0.367831	-2.413591
54	1	0	3.447781	-0.911866	-2.757719
55	1	0	2.966296	0.781480	-2.897021
56	6	0	3.286446	2.716870	-0.908925
57	6	0	3.719070	3.913922	-0.092584
58	8	0	2.967332	4.719633	0.430021
59	8	0	5.062763	3.991261	0.012132
60	6	0	5.588371	5.081974	0.799258
61	6	0	5.011200	-1.756911	-0.537648
62	1	0	3.990146	-0.708876	0.980422
63	6	0	5.056568	-2.944393	-1.161095
64	6	0	6.281298	-0.971296	-0.295500
65	1	0	6.000088	-3.350821	-1.515587
66	1	0	4.176355	-3.552407	-1.342371
67	1	0	6.426872	-0.788342	0.776910
68	1	0	7.157369	-1.504309	-0.674816
69	1	0	6.250884	0.012007	-0.778756
70	1	0	2.237062	2.868581	-1.159379
71	1	0	3.867705	2.708847	-1.836087
72	1	0	4.576977	1.341322	0.103809
73	1	0	2.980952	1.452196	0.829405
74	8	0	2.542669	-4.223486	1.496460
75	1	0	3.606136	-2.659304	2.182586
76	6	0	1.236036	-0.895868	2.870534
77	1	0	0.370915	-1.566484	2.881801
78	1	0	2.118189	-1.443631	3.226363
79	1	0	1.051990	-0.062133	3.552388
80	6	0	-4.781141	-2.855247	-0.360523
81	1	0	-5.782109	-2.634408	0.022225
82	1	0	-4.887005	-3.130519	-1.415967
83	1	0	-4.425914	-3.735027	0.181525
84	1	0	5.218387	5.022696	1.825019
85	1	0	6.670534	4.961815	0.773879
86	1	0	5.297922	6.039245	0.361115

13

Center Number	Atomic Number	Atomic Type	Coordinates (Angstroms) X	Y	Z
1	6	0	-1.022266	0.154093	-1.108601
2	6	0	0.360783	0.825179	-0.967449
3	1	0	-1.030654	-0.361873	-2.072501
4	1	0	-1.791843	0.932241	-1.164143
5	1	0	0.390019	1.490573	-0.099802
6	1	0	0.539943	1.445324	-1.854405
7	6	0	3.635617	-1.126056	-0.178345
8	6	0	2.477342	-2.060656	0.280856
9	6	0	1.247419	-1.099704	0.431212
10	6	0	1.433284	-0.258763	-0.851197
11	6	0	2.899829	-2.905413	1.461290
12	1	0	2.194683	-2.761881	-0.507976
13	8	0	1.406058	-0.190981	1.541654
14	6	0	-1.977580	-3.169783	0.578921
15	6	0	-2.451628	-1.981661	-0.321698
16	6	0	-1.363735	-0.842795	0.037994
17	6	0	-0.137902	-1.710075	0.413981
18	6	0	-0.496166	-2.960641	0.723887
19	1	0	-2.462987	-3.145198	1.566662
20	1	0	-2.224571	-4.144019	0.141381
21	6	0	-2.311465	-2.444691	-1.790187
22	6	0	-1.787952	-0.021895	1.283947
23	1	0	0.182518	-3.739739	1.055254
24	1	0	-2.618323	-1.681971	-2.510588
25	1	0	-1.276939	-2.720419	-2.017034
26	1	0	-2.929277	-3.330060	-1.967271
27	1	0	-0.976564	0.640192	1.592683
28	1	0	-2.664134	0.599335	1.077446
29	1	0	-2.027563	-0.668115	2.134111
30	6	0	-3.942585	-1.613268	-0.006028
31	6	0	-4.495822	-0.476780	-0.899391
32	6	0	-5.801067	0.161746	-0.420636
33	6	0	-6.282771	1.344681	-1.285299
34	6	0	-5.719519	2.580980	-0.567727
35	6	0	-5.577034	2.095621	0.871015
36	8	0	-5.618883	0.743347	0.916296
37	1	0	-6.581952	-0.590670	-0.297709
38	1	0	-7.377495	1.385619	-1.291093
39	1	0	-5.943639	1.257560	-2.320454
40	1	0	-4.693911	2.777837	-0.911603
41	6	0	-6.535348	3.868272	-0.695451
42	1	0	-3.969635	-1.260222	1.029825
43	1	0	-4.692204	-0.863159	-1.908039
44	1	0	-3.753114	0.318316	-1.016214
45	8	0	-5.429412	2.762588	1.874371
46	1	0	-6.093750	4.667273	-0.093791
47	1	0	-6.564346	4.197837	-1.738485
48	1	0	-7.565337	3.714262	-0.356777
49	1	0	1.238850	-0.967937	-1.668717
50	6	0	2.952306	0.092574	-0.964741
51	6	0	3.335177	0.155687	-2.455334

52	6	0	3.237625	1.460352	-0.282952
53	1	0	4.396961	0.364888	-2.612097
54	1	0	3.112766	-0.786676	-2.963715
55	1	0	2.766471	0.950179	-2.952423
56	6	0	4.710603	1.926465	-0.249756
57	6	0	4.819158	3.259973	0.453257
58	8	0	4.938065	3.407898	1.658005
59	8	0	4.737309	4.294630	-0.409247
60	6	0	4.778891	5.619276	0.165813
61	6	0	4.772810	-1.868163	-0.880964
62	1	0	4.066808	-0.697776	0.736337
63	6	0	4.603159	-3.003954	-1.575364
64	6	0	6.157917	-1.276784	-0.739263
65	1	0	5.448323	-3.494821	-2.050332
66	1	0	3.640086	-3.486546	-1.704150
67	1	0	6.913049	-1.928509	-1.186809
68	1	0	6.234737	-0.296863	-1.223143
69	1	0	6.415881	-1.128828	0.317185
70	1	0	5.117798	2.023507	-1.259023
71	1	0	5.324025	1.221764	0.315156
72	1	0	2.861818	1.422288	0.741745
73	1	0	2.656319	2.225677	-0.810593
74	8	0	2.531524	-4.050872	1.661503
75	1	0	3.626384	-2.435513	2.155869
76	6	0	1.215076	-0.693613	2.860645
77	1	0	0.312027	-1.306783	2.942049
78	1	0	2.077089	-1.280163	3.202964
79	1	0	1.116022	0.180214	3.508904
80	6	0	-4.880918	-2.838070	-0.074528
81	1	0	-5.886551	-2.577516	0.269858
82	1	0	-4.975447	-3.226127	-1.094818
83	1	0	-4.532944	-3.653746	0.564149
84	1	0	4.701133	6.303182	-0.678172
85	1	0	3.942624	5.760260	0.853909
86	1	0	5.720135	5.771104	0.698312

14

Center Number	Atomic Number	Atomic Type	Coordinates (Angstroms) X	Y	Z
1	6	0	-0.894206	0.018077	-1.196955
2	6	0	0.477859	0.728830	-1.183639
3	1	0	-0.931252	-0.575696	-2.114230
4	1	0	-1.685810	0.770997	-1.281775
5	1	0	0.525202	1.487425	-0.397163
6	1	0	0.606318	1.249143	-2.140141
7	6	0	3.824304	-0.928911	-0.203833
8	6	0	2.746033	-1.916692	0.315204
9	6	0	1.461421	-1.013661	0.378600
10	6	0	1.571765	-0.323172	-0.998354
11	6	0	3.202847	-2.626413	1.569957
12	1	0	2.523222	-2.697855	-0.418941
13	8	0	1.594241	0.024002	1.373252
14	6	0	-1.675295	-3.175469	0.810666
15	6	0	-2.210495	-2.097909	-0.189415
16	6	0	-1.163626	-0.889805	0.041615
17	6	0	0.103901	-1.674340	0.464980
18	6	0	-0.200176	-2.901340	0.900974
19	1	0	-2.143639	-3.073360	1.801787
20	1	0	-1.892280	-4.195474	0.473302
21	6	0	-2.071087	-2.694426	-1.609050
22	6	0	-1.593355	0.018388	1.223820
23	1	0	0.514324	-3.620877	1.286956
24	1	0	-2.424434	-2.021097	-2.394125
25	1	0	-1.027942	-2.942022	-1.828277
26	1	0	-2.648656	-3.620324	-1.687510
27	1	0	-0.813267	0.751831	1.438663
28	1	0	-2.510708	0.568349	0.996657
29	1	0	-1.765675	-0.559270	2.137141
30	6	0	-3.711016	-1.762023	0.119109
31	6	0	-4.317761	-0.715470	-0.845446
32	6	0	-5.696626	-0.179397	-0.455924
33	6	0	-6.220701	0.947938	-1.368876
34	6	0	-5.815713	2.237201	-0.637481
35	6	0	-5.713308	1.781210	0.814329
36	8	0	-5.644024	0.431326	0.879045
37	1	0	-6.419151	-0.992191	-0.368055
38	1	0	-7.311982	0.892615	-1.446301
39	1	0	-5.808040	0.879713	-2.378462
40	1	0	-4.794938	2.526634	-0.925320
41	6	0	-6.738935	3.441536	-0.829641
42	1	0	-3.738477	-1.333542	1.126384
43	1	0	-4.430991	-1.154025	-1.845337
44	1	0	-3.641670	0.137568	-0.959785
45	8	0	-5.679484	2.469667	1.813656
46	1	0	-6.406317	4.285186	-0.218939
47	1	0	-6.741112	3.754565	-1.878129
48	1	0	-7.767315	3.196372	-0.543800
49	1	0	1.320715	-1.126384	-1.704784
50	6	0	3.086524	-0.010010	-1.274225
51	6	0	3.390275	-0.460164	-2.721198

52	6	0	3.471867	1.493738	-1.200540
53	1	0	4.430658	-0.271246	-3.003128
54	1	0	3.198610	-1.528687	-2.858020
55	1	0	2.751345	0.088734	-3.423270
56	6	0	3.442502	2.204150	0.156783
57	6	0	3.617385	3.702143	0.036124
58	8	0	3.516898	4.358844	-0.985996
59	8	0	3.884812	4.258019	1.238313
60	6	0	4.023884	5.694914	1.268915
61	6	0	5.138164	-1.564405	-0.633058
62	1	0	4.064665	-0.284754	0.651009
63	6	0	5.270304	-2.862158	-0.948037
64	6	0	6.338434	-0.644185	-0.648784
65	1	0	6.236102	-3.269636	-1.234833
66	1	0	4.440676	-3.561449	-0.936541
67	1	0	7.248190	-1.179209	-0.934062
68	1	0	6.202456	0.187477	-1.349645
69	1	0	6.499001	-0.196644	0.340504
70	1	0	4.220482	1.838226	0.834239
71	1	0	2.498331	2.027346	0.682116
72	1	0	2.812764	2.036517	-1.885968
73	1	0	4.477324	1.610706	-1.621630
74	8	0	2.863040	-3.752722	1.892598
75	1	0	3.922512	-2.070670	2.205006
76	6	0	1.476442	-0.336164	2.746485
77	1	0	1.288768	0.590827	3.293720
78	1	0	0.647856	-1.029868	2.920291
79	1	0	2.403133	-0.780965	3.130602
80	6	0	-4.601002	-3.023830	0.159055
81	1	0	-5.596172	-2.785227	0.546325
82	1	0	-4.733347	-3.465343	-0.835181
83	1	0	-4.187280	-3.793496	0.815154
84	1	0	4.852297	6.010215	0.630820
85	1	0	3.101288	6.173347	0.932992
86	1	0	4.226719	5.943356	2.309796

15

Center Number	Atomic Number	Atomic Type	Coordinates (Angstroms) X	Y	Z
1	6	0	-0.955114	0.178442	-1.177880
2	6	0	0.443171	0.825990	-1.082930
3	1	0	-0.974686	-0.405760	-2.102141
4	1	0	-1.706047	0.968351	-1.291436
5	1	0	0.489150	1.552465	-0.266636
6	1	0	0.636842	1.376166	-2.011903
7	6	0	3.671251	-1.138244	-0.151432
8	6	0	2.491840	-2.013867	0.366085
9	6	0	1.285036	-1.016543	0.449112
10	6	0	1.490553	-0.270856	-0.888281
11	6	0	2.894008	-2.785374	1.602033
12	1	0	2.194231	-2.760388	-0.374076
13	8	0	1.465586	-0.039021	1.496503
14	6	0	-1.989929	-2.988958	0.747866
15	6	0	-2.435859	-1.858709	-0.237198
16	6	0	-1.319529	-0.724237	0.038068
17	6	0	-0.114385	-1.592992	0.474287
18	6	0	-0.503016	-2.808537	0.874483
19	1	0	-2.470605	-2.877315	1.732012
20	1	0	-2.264628	-3.985877	0.384281
21	6	0	-2.310223	-2.429810	-1.668442
22	6	0	-1.718925	0.195115	1.222219
23	1	0	0.155710	-3.579261	1.260910
24	1	0	-2.607784	-1.716306	-2.441387
25	1	0	-1.281115	-2.737508	-1.877811
26	1	0	-2.942700	-3.315698	-1.779669
27	1	0	-0.891384	0.860073	1.478112
28	1	0	-2.583385	0.817750	0.975094
29	1	0	-1.965792	-0.383142	2.118239
30	6	0	-3.915823	-1.431350	0.052353
31	6	0	-4.441974	-0.346634	-0.918151
32	6	0	-5.712548	0.368960	-0.478619
33	6	0	-6.247742	1.412499	-1.474161
34	6	0	-6.875961	2.489551	-0.573822
35	6	0	-6.103857	2.321422	0.731844
36	8	0	-5.443975	1.139990	0.742703
37	1	0	-6.497066	-0.346870	-0.212457
38	1	0	-6.958215	0.970493	-2.177925
39	1	0	-5.416092	1.832502	-2.050542
40	1	0	-6.691209	3.500943	-0.947196
41	6	0	-8.388078	2.319525	-0.330591
42	1	0	-3.929740	-1.005117	1.060983
43	1	0	-4.658509	-0.797320	-1.895956
44	1	0	-3.683587	0.421410	-1.094352
45	8	0	-6.056560	3.087404	1.672829
46	1	0	-8.747471	3.051586	0.398059
47	1	0	-8.932643	2.469482	-1.267644
48	1	0	-8.626469	1.318328	0.044370
49	1	0	1.279693	-1.031765	-1.653736
50	6	0	3.016688	0.038376	-1.023505
51	6	0	3.403069	-0.017037	-2.513608

52	6	0	3.334175	1.446183	-0.445863
53	1	0	4.471083	0.149824	-2.678768
54	1	0	3.155735	-0.986727	-2.954486
55	1	0	2.858902	0.756002	-3.068808
56	6	0	4.802550	1.882826	-0.453223
57	6	0	4.994940	3.300340	0.046790
58	8	0	4.122446	4.140788	0.172594
59	8	0	6.293960	3.544403	0.325791
60	6	0	6.618766	4.880547	0.767864
61	6	0	4.794552	-1.953311	-0.792535
62	1	0	4.106550	-0.656758	0.734613
63	6	0	4.602234	-3.129016	-1.410539
64	6	0	6.192564	-1.386447	-0.680074
65	1	0	5.438421	-3.670344	-1.844914
66	1	0	3.628512	-3.595796	-1.515598
67	1	0	6.935424	-2.086524	-1.071667
68	1	0	6.297378	-0.446517	-1.232955
69	1	0	6.445333	-1.167855	0.365375
70	1	0	5.231608	1.858737	-1.463297
71	1	0	5.432997	1.231362	0.158339
72	1	0	2.958402	1.496719	0.578350
73	1	0	2.763936	2.184011	-1.019534
74	8	0	2.506651	-3.909109	1.876059
75	1	0	3.624490	-2.282037	2.268638
76	6	0	1.261944	-0.446950	2.845579
77	1	0	1.151861	0.469802	3.429857
78	1	0	0.360679	-1.057126	2.961123
79	1	0	2.122749	-1.003944	3.237341
80	6	0	-4.884437	-2.634045	0.072567
81	1	0	-5.883752	-2.323437	0.393159
82	1	0	-4.988360	-3.094250	-0.916328
83	1	0	-4.558060	-3.408729	0.770957
84	1	0	6.347489	5.610381	0.001977
85	1	0	6.091834	5.111874	1.696090
86	1	0	7.695790	4.877311	0.929730

16

Center Number	Atomic Number	Atomic Type	Coordinates (Angstroms) X	Y	Z
1	6	0	-1.135653	0.111763	-1.125949
2	6	0	0.256548	0.773901	-1.052572
3	1	0	-1.165745	-0.466084	-2.053807
4	1	0	-1.898392	0.892896	-1.219886
5	1	0	0.309800	1.497994	-0.234583
6	1	0	0.428237	1.328639	-1.982957
7	6	0	3.527412	-1.191615	-0.253083
8	6	0	2.379068	-2.021494	0.384267
9	6	0	1.148247	-1.056950	0.465115
10	6	0	1.317271	-0.312707	-0.878310
11	6	0	2.795974	-2.756820	1.636438
12	1	0	2.056537	-2.822436	-0.294989
13	8	0	1.312844	-0.067080	1.504768
14	6	0	-2.104865	-3.076267	0.799975
15	6	0	-2.573563	-1.950783	-0.179296
16	6	0	-1.468679	-0.803767	0.089077
17	6	0	-0.243998	-1.656474	0.507400
18	6	0	-0.619222	-2.875870	0.908588
19	1	0	-2.575810	-2.970823	1.789314
20	1	0	-2.369920	-4.076351	0.438347
21	6	0	-2.453358	-2.519142	-1.612038
22	6	0	-1.864361	0.101042	1.285460
23	1	0	0.040652	-3.649092	1.289643
24	1	0	-2.765237	-1.808358	-2.381554
25	1	0	-1.422914	-2.815406	-1.831281
26	1	0	-3.077851	-3.411275	-1.718405
27	1	0	-1.044439	0.778084	1.532914
28	1	0	-2.741744	0.711430	1.054334
29	1	0	-2.090545	-0.485649	2.181225
30	6	0	-4.056419	-1.543003	0.124983
31	6	0	-4.604866	-0.459644	-0.834072
32	6	0	-5.915512	0.199668	-0.400293
33	6	0	-6.384145	1.346138	-1.319688
34	6	0	-5.837146	2.612335	-0.642530
35	6	0	-5.711755	2.184293	0.815890
36	8	0	-5.750649	0.834671	0.914005
37	1	0	-6.699107	-0.546485	-0.258044
38	1	0	-7.478656	1.383244	-1.346652
39	1	0	-6.026373	1.218811	-2.344381
40	1	0	-4.808227	2.802662	-0.979840
41	6	0	-6.659154	3.888310	-0.830839
42	1	0	-4.067520	-1.122556	1.135715
43	1	0	-4.792755	-0.900182	-1.821868
44	1	0	-3.863056	0.329795	-0.987939
45	8	0	-5.579263	2.890311	1.794234
46	1	0	-6.230419	4.712977	-0.255136
47	1	0	-6.676278	4.176614	-1.886255
48	1	0	-7.692591	3.740636	-0.499780
49	1	0	1.097420	-1.079991	-1.635941
50	6	0	2.834732	0.008519	-1.056087
51	6	0	3.160743	0.028881	-2.562431

52	6	0	3.185168	1.389406	-0.433232
53	1	0	4.226813	0.171102	-2.760917
54	1	0	2.865755	-0.903202	-3.051228
55	1	0	2.622325	0.851292	-3.047786
56	6	0	4.657359	1.811001	-0.509434
57	6	0	4.900785	3.199292	0.045328
58	8	0	4.059844	4.067978	0.195046
59	8	0	6.205907	3.384658	0.339931
60	6	0	6.577321	4.692074	0.828243
61	6	0	4.535595	-2.046498	-1.021497
62	1	0	4.077550	-0.745883	0.581268
63	6	0	4.196942	-3.102666	-1.775731
64	6	0	5.984895	-1.641067	-0.887736
65	1	0	4.952902	-3.675907	-2.305748
66	1	0	3.170968	-3.435140	-1.905614
67	1	0	6.645330	-2.329425	-1.422254
68	1	0	6.158763	-0.633465	-1.283668
69	1	0	6.283993	-1.621828	0.167550
70	1	0	5.019142	1.834902	-1.545741
71	1	0	5.315059	1.120409	0.025825
72	1	0	2.870946	1.393926	0.612475
73	1	0	2.588663	2.156005	-0.938830
74	8	0	3.915725	-2.729413	2.118581
75	1	0	2.018665	-3.390908	2.105877
76	6	0	1.156331	-0.474564	2.861030
77	1	0	0.263997	-1.092911	3.004943
78	1	0	2.035509	-1.015867	3.229281
79	1	0	1.050227	0.443429	3.444077
80	6	0	-5.010663	-2.757335	0.147851
81	1	0	-6.003028	-2.464850	0.504470
82	1	0	-5.138113	-3.199061	-0.846798
83	1	0	-4.655929	-3.542152	0.820577
84	1	0	7.651933	4.642315	0.998357
85	1	0	6.340915	5.456505	0.084797
86	1	0	6.051080	4.912875	1.759387

17

Center Number	Atomic Number	Atomic Type	Coordinates (Angstroms) X	Y	Z
1	6	0	-0.704470	0.214670	-1.067228
2	6	0	0.674688	0.863089	-0.816097
3	1	0	-0.674406	-0.210940	-2.074089
4	1	0	-1.467980	1.000571	-1.083430
5	1	0	0.672486	1.460970	0.100604
6	1	0	0.880035	1.544395	-1.649336
7	6	0	3.893939	-1.128826	0.046178
8	6	0	2.734402	-2.130351	0.274997
9	6	0	1.491252	-1.184438	0.440096
10	6	0	1.745982	-0.227422	-0.746123
11	6	0	3.068805	-3.105972	1.380835
12	1	0	2.527627	-2.731245	-0.615968
13	8	0	1.590752	-0.373594	1.631371
14	6	0	-1.733695	-3.248435	0.247152
15	6	0	-2.173926	-1.973034	-0.544259
16	6	0	-1.097428	-0.881486	-0.034111
17	6	0	0.110590	-1.786877	0.302668
18	6	0	-0.258987	-3.061544	0.468294
19	1	0	-2.254715	-3.321490	1.214056
20	1	0	-1.965346	-4.172007	-0.296006
21	6	0	-1.983069	-2.285426	-2.046418
22	6	0	-1.571218	-0.178547	1.265298
23	1	0	0.405765	-3.873161	0.744275
24	1	0	-2.269527	-1.453638	-2.695236
25	1	0	-0.939911	-2.534129	-2.264953
26	1	0	-2.588550	-3.150005	-2.334686
27	1	0	-0.776911	0.458746	1.659137
28	1	0	-2.447869	0.450201	1.086067
29	1	0	-1.830559	-0.901516	2.045185
30	6	0	-3.674180	-1.632669	-0.243693
31	6	0	-4.194664	-0.403972	-1.028874
32	6	0	-5.500973	0.192840	-0.522167
33	6	0	-6.031653	1.387133	-1.333286
34	6	0	-6.732827	2.260998	-0.279653
35	6	0	-6.000343	1.880897	1.004132
36	8	0	-5.300529	0.736066	0.828103
37	1	0	-6.272677	-0.577278	-0.422375
38	1	0	-6.697410	1.064523	-2.138362
39	1	0	-5.192643	1.929182	-1.783432
40	1	0	-6.574690	3.329013	-0.455096
41	6	0	-8.244989	2.001156	-0.135490
42	1	0	-3.734973	-1.389985	0.822479
43	1	0	-4.362205	-0.678969	-2.078814
44	1	0	-3.451798	0.398822	-1.034140
45	8	0	-6.010711	2.465424	2.068450
46	1	0	-8.658680	2.575014	0.698493
47	1	0	-8.760706	2.303708	-1.051783
48	1	0	-8.457991	0.941125	0.039807
49	1	0	1.588940	-0.856513	-1.634254
50	6	0	3.275284	0.111271	-0.756381
51	6	0	3.751139	0.164806	-2.220974

52	6	0	3.677664	1.408449	0.004717
53	1	0	4.799044	0.470367	-2.307769
54	1	0	3.650878	-0.811981	-2.702969
55	1	0	3.149819	0.878241	-2.795211
56	6	0	3.435742	2.748347	-0.702788
57	6	0	3.988091	3.924975	0.075303
58	8	0	4.792400	3.866956	0.988641
59	8	0	3.490083	5.091261	-0.389515
60	6	0	3.978688	6.297156	0.237471
61	6	0	5.178124	-1.734847	-0.501731
62	1	0	4.145102	-0.736413	1.040793
63	6	0	5.228789	-2.904765	-1.157506
64	6	0	6.446376	-0.956813	-0.227529
65	1	0	6.175237	-3.301092	-1.515692
66	1	0	4.350376	-3.508044	-1.361719
67	1	0	6.582190	-0.801611	0.850389
68	1	0	7.325466	-1.480504	-0.612891
69	1	0	6.420757	0.038976	-0.684558
70	1	0	2.378512	2.944315	-0.893302
71	1	0	3.928707	2.775871	-1.682874
72	1	0	4.748786	1.349881	0.223289
73	1	0	3.170111	1.413179	0.973230
74	8	0	2.685578	-4.263232	1.426591
75	1	0	3.742277	-2.723017	2.174646
76	6	0	1.360926	-0.995815	2.891865
77	1	0	0.509113	-1.682941	2.863239
78	1	0	2.247805	-1.538460	3.243642
79	1	0	1.148666	-0.191183	3.600040
80	6	0	-4.610056	-2.839175	-0.473792
81	1	0	-5.626929	-2.612416	-0.138360
82	1	0	-4.670301	-3.115273	-1.532397
83	1	0	-4.284190	-3.719595	0.085554
84	1	0	5.060031	6.380806	0.108475
85	1	0	3.468050	7.114059	-0.270571
86	1	0	3.736598	6.296171	1.302298

18

Center Number	Atomic Number	Atomic Type	Coordinates (Angstroms) X	Y	Z
1	6	0	-0.790091	0.037265	-1.226760
2	6	0	0.589313	0.733682	-1.220406
3	1	0	-0.826748	-0.579017	-2.129090
4	1	0	-1.571721	0.797325	-1.336228
5	1	0	0.638269	1.511438	-0.453181
6	1	0	0.730567	1.228250	-2.188708
7	6	0	3.909798	-0.928188	-0.166555
8	6	0	2.818115	-1.895339	0.362978
9	6	0	1.541192	-0.979561	0.392802
10	6	0	1.671075	-0.323690	-0.999374
11	6	0	3.256306	-2.580703	1.637706
12	1	0	2.596334	-2.690580	-0.356225
13	8	0	1.675768	0.080302	1.363546
14	6	0	-1.620688	-3.098427	0.851820
15	6	0	-2.139881	-2.037071	-0.172910
16	6	0	-1.079270	-0.835213	0.033101
17	6	0	0.176099	-1.623970	0.483342
18	6	0	-0.143143	-2.838258	0.943510
19	1	0	-2.091052	-2.969648	1.838812
20	1	0	-1.849186	-4.122778	0.535541
21	6	0	-2.005143	-2.663740	-1.580025
22	6	0	-1.508404	0.110371	1.185653
23	1	0	0.561578	-3.556000	1.350182
24	1	0	-2.353013	-2.002424	-2.377852
25	1	0	-0.963691	-2.923272	-1.793619
26	1	0	-2.588943	-3.587054	-1.641523
27	1	0	-0.722481	0.841840	1.386102
28	1	0	-2.419614	0.660240	0.935081
29	1	0	-1.691648	-0.440404	2.113667
30	6	0	-3.636184	-1.674889	0.122881
31	6	0	-4.232640	-0.663843	-0.885918
32	6	0	-5.527545	0.011232	-0.453712
33	6	0	-6.134038	0.974920	-1.487951
34	6	0	-6.794620	2.066136	-0.628811
35	6	0	-5.986689	2.003864	0.664309
36	8	0	-5.271070	0.856778	0.720444
37	1	0	-6.270325	-0.726339	-0.132952
38	1	0	-6.837262	0.463879	-2.150966
39	1	0	-5.336658	1.403668	-2.104826
40	1	0	-6.668109	3.064339	-1.057928
41	6	0	-8.290843	1.837124	-0.339866
42	1	0	-3.658594	-1.204971	1.111862
43	1	0	-4.444626	-1.170562	-1.836861
44	1	0	-3.515250	0.129856	-1.112707
45	8	0	-5.955032	2.820416	1.562557
46	1	0	-8.668446	2.586976	0.360922
47	1	0	-8.862942	1.914337	-1.269268
48	1	0	-8.472349	0.845923	0.089483
49	1	0	1.419285	-1.141364	-1.688682
50	6	0	3.191325	-0.030325	-1.267245
51	6	0	3.506173	-0.518572	-2.699386

52	6	0	3.588642	1.471598	-1.227425
53	1	0	4.550847	-0.344467	-2.975078
54	1	0	3.307423	-1.588592	-2.812523
55	1	0	2.878869	0.017855	-3.421297
56	6	0	3.553384	2.215758	0.111479
57	6	0	3.740794	3.708979	-0.044583
58	8	0	3.670859	4.338917	-1.085884
59	8	0	3.982259	4.294962	1.148840
60	6	0	4.131085	5.731155	1.144567
61	6	0	5.224026	-1.582511	-0.564562
62	1	0	4.143976	-0.265636	0.675849
63	6	0	5.351046	-2.888125	-0.847648
64	6	0	6.430579	-0.670663	-0.587484
65	1	0	6.317442	-3.308506	-1.113024
66	1	0	4.516615	-3.581527	-0.830009
67	1	0	7.339915	-1.217948	-0.849980
68	1	0	6.308139	0.145686	-1.308621
69	1	0	6.582860	-0.201611	0.393126
70	1	0	4.323777	1.861400	0.803797
71	1	0	2.604295	2.057792	0.633777
72	1	0	2.939721	2.002249	-1.931769
73	1	0	4.598442	1.569747	-1.642712
74	8	0	2.901670	-3.695629	1.983304
75	1	0	3.976684	-2.018131	2.265934
76	6	0	1.542190	-0.246162	2.743667
77	1	0	0.702581	-0.924075	2.926766
78	1	0	2.459323	-0.695094	3.145706
79	1	0	1.363438	0.695934	3.267561
80	6	0	-4.539433	-2.924148	0.212913
81	1	0	-5.551227	-2.650376	0.528188
82	1	0	-4.627290	-3.437904	-0.750853
83	1	0	-4.167725	-3.644926	0.945622
84	1	0	4.975921	6.023936	0.517359
85	1	0	3.219736	6.206509	0.775199
86	1	0	4.312034	6.005934	2.182861

19

Center Number	Atomic Number	Atomic Type	Coordinates (Angstroms) X	Y	Z
1	6	0	-0.914254	0.110503	-1.219663
2	6	0	0.472097	0.788391	-1.173774
3	1	0	-0.931905	-0.517515	-2.114652
4	1	0	-1.682079	0.879181	-1.362269
5	1	0	0.513106	1.552739	-0.392702
6	1	0	0.643183	1.296826	-2.130428
7	6	0	3.748676	-1.061540	-0.179713
8	6	0	2.592786	-1.934027	0.391656
9	6	0	1.365957	-0.959505	0.436405
10	6	0	1.542578	-0.278435	-0.939001
11	6	0	3.021138	-2.637718	1.659539
12	1	0	2.303250	-2.721876	-0.308147
13	8	0	1.534666	0.074550	1.430552
14	6	0	-1.862490	-2.984401	0.860838
15	6	0	-2.344079	-1.908166	-0.166646
16	6	0	-1.247628	-0.740243	0.042157
17	6	0	-0.020432	-1.562708	0.504737
18	6	0	-0.378819	-2.767297	0.962369
19	1	0	-2.334492	-2.841532	1.845052
20	1	0	-2.119122	-4.001677	0.543346
21	6	0	-2.224583	-2.539379	-1.573065
22	6	0	-1.653465	0.225687	1.186409
23	1	0	0.300312	-3.505965	1.375209
24	1	0	-2.544831	-1.865564	-2.372101
25	1	0	-1.192043	-2.835864	-1.781893
26	1	0	-2.840418	-3.441463	-1.638245
27	1	0	-0.839845	0.922628	1.398763
28	1	0	-2.536796	0.813931	0.922799
29	1	0	-1.873383	-0.315292	2.112208
30	6	0	-3.828974	-1.498409	0.123896
31	6	0	-4.390129	-0.469925	-0.887261
32	6	0	-5.673885	0.233023	-0.466477
33	6	0	-6.244129	1.218835	-1.500442
34	6	0	-6.892868	2.316315	-0.640318
35	6	0	-6.102883	2.226046	0.662285
36	8	0	-5.413217	1.063009	0.717663
37	1	0	-6.438112	-0.489287	-0.161881
38	1	0	-6.948737	0.729081	-2.177942
39	1	0	-5.428736	1.635420	-2.101983
40	1	0	-6.740571	3.315317	-1.059023
41	6	0	-8.396876	2.115178	-0.372034
42	1	0	-3.838903	-1.026346	1.112061
43	1	0	-4.603640	-0.967370	-1.842741
44	1	0	-3.651783	0.307670	-1.102544
45	8	0	-6.064856	3.034259	1.567752
46	1	0	-8.768268	2.866642	0.330330
47	1	0	-8.955542	2.211385	-1.307811
48	1	0	-8.603313	1.124262	0.046548
49	1	0	1.337937	-1.079545	-1.663839
50	6	0	3.061914	0.050429	-1.107765
51	6	0	3.434981	-0.077010	-2.596505

52	6	0	3.355033	1.494212	-0.610657
53	1	0	4.495441	0.113024	-2.783157
54	1	0	3.212900	-1.077472	-2.977987
55	1	0	2.862266	0.646604	-3.188222
56	6	0	4.827288	1.962714	-0.656914
57	6	0	4.922148	3.411717	-0.236941
58	8	0	4.721447	4.365235	-0.970704
59	8	0	5.227115	3.542609	1.070680
60	6	0	5.289740	4.890382	1.587353
61	6	0	4.886602	-1.883602	-0.784743
62	1	0	4.177963	-0.523815	0.676098
63	6	0	4.719273	-3.098594	-1.329624
64	6	0	6.269825	-1.275141	-0.720754
65	1	0	5.565201	-3.643343	-1.740115
66	1	0	3.757372	-3.595891	-1.395649
67	1	0	7.026361	-1.974146	-1.087022
68	1	0	6.341495	-0.361955	-1.321435
69	1	0	6.529448	-0.997082	0.308756
70	1	0	5.226885	1.900574	-1.672537
71	1	0	5.450452	1.360221	0.006831
72	1	0	2.989029	1.591252	0.414215
73	1	0	2.769742	2.185563	-1.227516
74	8	0	2.645691	-3.747695	1.998107
75	1	0	3.759019	-2.096318	2.286854
76	6	0	1.366156	-0.272843	2.801283
77	1	0	1.229394	0.666696	3.342128
78	1	0	0.490714	-0.910099	2.961105
79	1	0	2.253722	-0.776376	3.205198
80	6	0	-4.771263	-2.718780	0.212594
81	1	0	-5.772408	-2.414905	0.533953
82	1	0	-4.879328	-3.225245	-0.753002
83	1	0	-4.419139	-3.454384	0.940132
84	1	0	5.535607	4.783953	2.642916
85	1	0	6.063727	5.459181	1.067685
86	1	0	4.325503	5.388192	1.464886

20

Center Number	Atomic Number	Atomic Type	Coordinates (Angstroms) X	Y	Z
1	6	0	-0.891373	0.191303	-1.141590
2	6	0	0.496097	0.854597	-1.006084
3	1	0	-0.895608	-0.349669	-2.091770
4	1	0	-1.653335	0.974460	-1.224111
5	1	0	0.523628	1.543627	-0.156986
6	1	0	0.688392	1.448755	-1.907985
7	6	0	3.747383	-1.103051	-0.138541
8	6	0	2.577296	-2.015023	0.335854
9	6	0	1.354742	-1.039755	0.450958
10	6	0	1.558476	-0.234211	-0.851388
11	6	0	2.983124	-2.830692	1.542227
12	1	0	2.295033	-2.734949	-0.435982
13	8	0	1.512483	-0.103999	1.538807
14	6	0	-1.888810	-3.077527	0.628768
15	6	0	-2.344010	-1.912455	-0.310487
16	6	0	-1.251086	-0.772001	0.028121
17	6	0	-0.035603	-1.638550	0.438477
18	6	0	-0.406789	-2.877023	0.780377
19	1	0	-2.382187	-3.018459	1.611198
20	1	0	-2.141616	-4.062059	0.218472
21	6	0	-2.192677	-2.416790	-1.764224
22	6	0	-1.680604	0.086164	1.247081
23	1	0	0.262157	-3.652271	1.139331
24	1	0	-2.491811	-1.673634	-2.508053
25	1	0	-1.156879	-2.700550	-1.974505
26	1	0	-2.810640	-3.305608	-1.922530
27	1	0	-0.866247	0.749913	1.544767
28	1	0	-2.550635	0.707197	1.015609
29	1	0	-1.930875	-0.535497	2.112592
30	6	0	-3.834794	-1.524186	-0.021190
31	6	0	-4.371631	-0.413160	-0.956048
32	6	0	-5.641596	0.284088	-0.486197
33	6	0	-6.201862	1.337406	-1.457357
34	6	0	-6.822625	2.397041	-0.531592
35	6	0	-6.027405	2.216406	0.758407
36	8	0	-5.359540	1.039592	0.741853
37	1	0	-6.415015	-0.442742	-0.217332
38	1	0	-6.920820	0.899799	-2.155235
39	1	0	-5.383323	1.771834	-2.041840
40	1	0	-6.651202	3.414861	-0.893739
41	6	0	-8.329347	2.213381	-0.265623
42	1	0	-3.868649	-1.138523	1.003080
43	1	0	-4.592925	-0.836942	-1.944769
44	1	0	-3.618173	0.363365	-1.115804
45	8	0	-5.968806	2.970011	1.708740
46	1	0	-8.681026	2.932919	0.479092
47	1	0	-8.890504	2.373018	-1.191222
48	1	0	-8.555408	1.205585	0.099196
49	1	0	1.365454	-0.963144	-1.651733
50	6	0	3.081136	0.101619	-0.960488
51	6	0	3.478204	0.124862	-2.448402

52	6	0	3.372634	1.483553	-0.310253
53	1	0	4.543773	0.318367	-2.599144
54	1	0	3.249956	-0.826895	-2.936276
55	1	0	2.923405	0.913585	-2.969857
56	6	0	4.849527	1.936874	-0.273743
57	6	0	4.963102	3.286671	0.396662
58	8	0	5.060495	3.464350	1.599341
59	8	0	4.912805	4.299508	-0.493643
60	6	0	4.962292	5.637938	0.047879
61	6	0	4.882327	-1.873149	-0.814282
62	1	0	4.175936	-0.655910	0.768304
63	6	0	4.706723	-3.024042	-1.481848
64	6	0	6.271974	-1.291227	-0.677279
65	1	0	5.550470	-3.534134	-1.938753
66	1	0	3.739974	-3.500463	-1.606130
67	1	0	7.024068	-1.961135	-1.102557
68	1	0	6.361729	-0.324670	-1.185299
69	1	0	6.523777	-1.118814	0.376944
70	1	0	5.268218	2.004923	-1.280630
71	1	0	5.450557	1.241044	0.315163
72	1	0	2.986636	1.475374	0.711242
73	1	0	2.803804	2.240482	-0.863042
74	8	0	2.606644	-3.968601	1.768739
75	1	0	3.705187	-2.346491	2.231623
76	6	0	1.303714	-0.569487	2.868590
77	1	0	1.201344	0.322112	3.491670
78	1	0	0.396832	-1.176076	2.955847
79	1	0	2.158835	-1.150388	3.236922
80	6	0	-4.780793	-2.744238	-0.060588
81	1	0	-5.794331	-2.460406	0.239925
82	1	0	-4.848847	-3.177926	-1.064363
83	1	0	-4.459117	-3.532103	0.625188
84	1	0	5.897326	5.791894	0.590670
85	1	0	4.905861	6.300779	-0.814397
86	1	0	4.117708	5.806667	0.719317

21

Center Number	Atomic Number	Atomic Type	Coordinates (Angstroms) X	Y	Z
1	6	0	-1.101849	0.052790	-1.150619
2	6	0	0.278232	0.742912	-1.129028
3	1	0	-1.134469	-0.565610	-2.051800
4	1	0	-1.880922	0.814530	-1.266888
5	1	0	0.330065	1.501972	-0.343452
6	1	0	0.425155	1.259159	-2.085349
7	6	0	3.598337	-1.123323	-0.291670
8	6	0	2.475945	-1.940588	0.404799
9	6	0	1.227128	-0.998052	0.456965
10	6	0	1.361199	-0.316169	-0.922776
11	6	0	2.924954	-2.603969	1.685759
12	1	0	2.159694	-2.780761	-0.228285
13	8	0	1.384424	0.045095	1.445114
14	6	0	-1.978529	-3.066339	0.925933
15	6	0	-2.483532	-1.994435	-0.094295
16	6	0	-1.399313	-0.814660	0.108396
17	6	0	-0.151728	-1.622646	0.547338
18	6	0	-0.496297	-2.830989	1.005729
19	1	0	-2.438833	-2.929387	1.916322
20	1	0	-2.226322	-4.086270	0.610462
21	6	0	-2.368372	-2.622690	-1.502446
22	6	0	-1.798573	0.133120	1.269853
23	1	0	0.183922	-3.574147	1.410500
24	1	0	-2.702012	-1.952070	-2.298344
25	1	0	-1.334526	-2.907860	-1.720531
26	1	0	-2.975805	-3.530787	-1.563040
27	1	0	-0.992663	0.840882	1.474358
28	1	0	-2.694554	0.710785	1.026977
29	1	0	-1.995920	-0.419217	2.193788
30	6	0	-3.971035	-1.604236	0.211721
31	6	0	-4.554850	-0.578231	-0.788597
32	6	0	-5.883992	0.058294	-0.377741
33	6	0	-6.388190	1.152959	-1.340323
34	6	0	-5.877128	2.460739	-0.715728
35	6	0	-5.734199	2.094402	0.757910
36	8	0	-5.736095	0.749431	0.909868
37	1	0	-6.644430	-0.704911	-0.204126
38	1	0	-7.483329	1.157026	-1.364936
39	1	0	-6.029213	0.995338	-2.360430
40	1	0	-4.855497	2.668187	-1.064730
41	6	0	-6.737676	3.703307	-0.950284
42	1	0	-3.978161	-1.137142	1.201960
43	1	0	-4.731919	-1.064150	-1.756801
44	1	0	-3.837163	0.226066	-0.976703
45	8	0	-5.616386	2.842151	1.706737
46	1	0	-6.332593	4.562444	-0.408800
47	1	0	-6.766216	3.949477	-2.016057
48	1	0	-7.765356	3.537539	-0.609993
49	1	0	1.143745	-1.121423	-1.640443
50	6	0	2.869813	0.021534	-1.141130
51	6	0	3.173252	-0.022967	-2.650828

52	6	0	3.203465	1.436505	-0.588335
53	1	0	4.233442	0.128332	-2.871305
54	1	0	2.887799	-0.982656	-3.089071
55	1	0	2.613716	0.766078	-3.166559
56	6	0	4.674100	1.891981	-0.730516
57	6	0	4.831842	3.305939	-0.220642
58	8	0	4.526299	4.309261	-0.844067
59	8	0	5.328184	3.344031	1.032920
60	6	0	5.470821	4.651143	1.631173
61	6	0	4.610051	-1.995294	-1.035954
62	1	0	4.154407	-0.625311	0.508650
63	6	0	4.283376	-3.105385	-1.714067
64	6	0	6.049361	-1.540215	-0.971306
65	1	0	5.042010	-3.687898	-2.229867
66	1	0	3.265134	-3.475226	-1.793891
67	1	0	6.715224	-2.242278	-1.480615
68	1	0	6.180619	-0.555785	-1.436026
69	1	0	6.377780	-1.444048	0.070997
70	1	0	4.979713	1.897787	-1.780211
71	1	0	5.343305	1.234528	-0.171635
72	1	0	2.921572	1.476264	0.466227
73	1	0	2.578252	2.165988	-1.115700
74	8	0	4.051020	-2.532405	2.147750
75	1	0	2.165942	-3.226277	2.198855
76	6	0	1.260958	-0.300921	2.821931
77	1	0	0.398488	-0.949853	3.007233
78	1	0	2.167717	-0.783762	3.203869
79	1	0	1.120217	0.638852	3.361400
80	6	0	-4.897851	-2.836244	0.304257
81	1	0	-5.890560	-2.549076	0.664065
82	1	0	-5.031184	-3.324623	-0.667591
83	1	0	-4.515903	-3.582505	1.005315
84	1	0	5.878873	4.471020	2.624670
85	1	0	6.153692	5.265371	1.040371
86	1	0	4.499174	5.145270	1.697723

22

Center Number	Atomic Number	Atomic Type	Coordinates (Angstroms) X	Y	Z
1	6	0	-0.704536	0.214745	-1.067131
2	6	0	0.674642	0.863142	-0.816054
3	1	0	-0.674541	-0.210806	-2.074020
4	1	0	-1.468043	1.000652	-1.083238
5	1	0	0.672502	1.460974	0.100679
6	1	0	0.879939	1.544491	-1.649270
7	6	0	3.893939	-1.128838	0.045900
8	6	0	2.734410	-2.130366	0.274749
9	6	0	1.491277	-1.184454	0.439979
10	6	0	1.745933	-0.227381	-0.746209
11	6	0	3.068883	-3.106047	1.380513
12	1	0	2.527570	-2.731214	-0.616233
13	8	0	1.590864	-0.373671	1.631289
14	6	0	-1.733690	-3.248427	0.247137
15	6	0	-2.173959	-1.972991	-0.544197
16	6	0	-1.097433	-0.881467	-0.034052
17	6	0	0.110603	-1.786878	0.302613
18	6	0	-0.258970	-3.061551	0.468205
19	1	0	-2.254657	-3.321519	1.214067
20	1	0	-1.965375	-4.171975	-0.296046
21	6	0	-1.983170	-2.285317	-2.046378
22	6	0	-1.571152	-0.178598	1.265422
23	1	0	0.405795	-3.873183	0.744110
24	1	0	-2.269652	-1.453499	-2.695146
25	1	0	-0.940022	-2.534015	-2.264968
26	1	0	-2.588667	-3.149881	-2.334658
27	1	0	-0.776821	0.458668	1.659255
28	1	0	-2.447809	0.450164	1.086271
29	1	0	-1.830458	-0.901609	2.045283
30	6	0	-3.674197	-1.632638	-0.243546
31	6	0	-4.194719	-0.403911	-1.028655
32	6	0	-5.501013	0.192872	-0.521877
33	6	0	-6.031731	1.387193	-1.332928
34	6	0	-6.732882	2.261005	-0.279236
35	6	0	-6.000351	1.880860	1.004509
36	8	0	-5.300526	0.736046	0.828409
37	1	0	-6.272708	-0.577255	-0.422088
38	1	0	-6.697510	1.064611	-2.137998
39	1	0	-5.192740	1.929272	-1.783076
40	1	0	-6.574765	3.329030	-0.454640
41	6	0	-8.245036	2.001138	-0.135034
42	1	0	-3.734940	-1.389995	0.822639
43	1	0	-4.362297	-0.678865	-2.078600
44	1	0	-3.451857	0.398887	-1.033916
45	8	0	-6.010689	2.465343	2.068852
46	1	0	-8.658706	2.574957	0.698987
47	1	0	-8.760787	2.303722	-1.051298
48	1	0	-8.458020	0.941097	0.040226
49	1	0	1.588823	-0.856426	-1.634360
50	6	0	3.275237	0.111301	-0.756558
51	6	0	3.750988	0.164903	-2.221182

52	6	0	3.677683	1.408438	0.004574
53	1	0	4.798884	0.470476	-2.308037
54	1	0	3.650699	-0.811864	-2.703211
55	1	0	3.149621	0.878359	-2.795346
56	6	0	3.435715	2.748375	-0.702841
57	6	0	3.988157	3.924955	0.075256
58	8	0	4.792564	3.866881	0.988503
59	8	0	3.490107	5.091271	-0.389445
60	6	0	3.978793	6.297129	0.237549
61	6	0	5.178081	-1.734843	-0.502127
62	1	0	4.145173	-0.736475	1.040515
63	6	0	5.228693	-2.904727	-1.157965
64	6	0	6.446359	-0.956836	-0.227965
65	1	0	6.175114	-3.301045	-1.516233
66	1	0	4.350262	-3.507987	-1.362154
67	1	0	6.582244	-0.801691	0.849952
68	1	0	7.325419	-1.480516	-0.613411
69	1	0	6.420722	0.038977	-0.684940
70	1	0	2.378471	2.944369	-0.893247
71	1	0	3.928589	2.775942	-1.682972
72	1	0	4.748822	1.349852	0.223058
73	1	0	3.170206	1.413122	0.973128
74	8	0	2.685640	-4.263303	1.426243
75	1	0	3.742423	-2.723141	2.174290
76	6	0	1.361130	-0.995959	2.891766
77	1	0	0.509301	-1.683068	2.863171
78	1	0	2.248027	-1.538642	3.243440
79	1	0	1.148944	-0.191363	3.600004
80	6	0	-4.610083	-2.839136	-0.473646
81	1	0	-5.626940	-2.612394	-0.138155
82	1	0	-4.670380	-3.115194	-1.532259
83	1	0	-4.284187	-3.719577	0.085650
84	1	0	3.736804	6.296101	1.302399
85	1	0	5.060125	6.380764	0.108455
86	1	0	3.468122	7.114063	-0.270408

23

Center Number	Atomic Number	Atomic Type	Coordinates (Angstroms) X	Y	Z
1	6	0	1.239352	-0.303746	-1.162915
2	6	0	-0.101452	-1.066445	-1.104470
3	1	0	1.244644	0.261301	-2.099188
4	1	0	2.059449	-1.027383	-1.230584
5	1	0	-0.117242	-1.772917	-0.269660
6	1	0	-0.207837	-1.653159	-2.025221
7	6	0	-3.519026	0.654348	-0.367202
8	6	0	-2.434719	1.623500	0.192026
9	6	0	-1.157164	0.727607	0.348291
10	6	0	-1.245629	-0.058500	-0.977553
11	6	0	-2.954970	2.377632	1.394112
12	1	0	-2.157897	2.382486	-0.543827
13	8	0	-1.294874	-0.243831	1.405603
14	6	0	1.927448	2.975690	0.727068
15	6	0	2.503956	1.870608	-0.217874
16	6	0	1.477862	0.651508	0.043294
17	6	0	0.187219	1.421104	0.414736
18	6	0	0.457243	2.672284	0.802614
19	1	0	2.378763	2.923792	1.729875
20	1	0	2.129273	3.985850	0.352545
21	6	0	2.380535	2.405526	-1.663172
22	6	0	1.907295	-0.203900	1.264442
23	1	0	-0.278603	3.391953	1.146269
24	1	0	2.754842	1.704179	-2.413232
25	1	0	1.338275	2.630669	-1.909114
26	1	0	2.947954	3.334732	-1.771457
27	1	0	1.132647	-0.935381	1.503518
28	1	0	2.832648	-0.752325	1.067211
29	1	0	2.065572	0.411506	2.155428
30	6	0	4.005287	1.577715	0.130123
31	6	0	4.653295	0.517323	-0.792057
32	6	0	6.018762	-0.004922	-0.341393
33	6	0	6.597053	-1.122268	-1.233541
34	6	0	6.166479	-2.418629	-0.530154
35	6	0	5.995000	-1.972107	0.918205
36	8	0	5.910769	-0.623098	0.986902
37	1	0	6.727857	0.814762	-0.215918
38	1	0	7.690382	-1.057822	-1.255929
39	1	0	6.234718	-1.051253	-2.262090
40	1	0	5.161514	-2.711129	-0.866716
41	6	0	7.103894	-3.616890	-0.687981
42	1	0	4.022817	1.180242	1.150142
43	1	0	4.801046	0.940507	-1.794073
44	1	0	3.986015	-0.341227	-0.916560
45	8	0	5.922776	-2.666516	1.911255
46	1	0	6.747210	-4.467205	-0.100548
47	1	0	7.157155	-3.921150	-1.737722
48	1	0	8.116370	-3.369331	-0.351912
49	1	0	-1.067589	0.701925	-1.751757
50	6	0	-2.736460	-0.495156	-1.168012
51	6	0	-3.044724	-0.513800	-2.677573

52	6	0	-2.959591	-1.914195	-0.565781
53	1	0	-4.082607	-0.779158	-2.897223
54	1	0	-2.858480	0.462034	-3.134293
55	1	0	-2.405366	-1.249731	-3.179357
56	6	0	-4.343325	-2.576256	-0.693894
57	6	0	-5.308147	-2.332620	0.449489
58	8	0	-5.033422	-1.857338	1.536843
59	8	0	-6.546646	-2.767540	0.132533
60	6	0	-7.550393	-2.672716	1.166685
61	6	0	-4.649634	1.371383	-1.103716
62	1	0	-3.974837	0.164786	0.500412
63	6	0	-4.501622	2.533440	-1.758153
64	6	0	-6.008873	0.712911	-1.038323
65	1	0	-5.345414	2.998785	-2.260716
66	1	0	-3.557379	3.063643	-1.825073
67	1	0	-6.763809	1.302181	-1.565825
68	1	0	-5.998474	-0.288663	-1.482064
69	1	0	-6.331086	0.594185	0.003592
70	1	0	-4.210075	-3.666709	-0.712633
71	1	0	-4.852540	-2.345271	-1.633933
72	1	0	-2.674256	-1.901317	0.487626
73	1	0	-2.252883	-2.580809	-1.071490
74	8	0	-2.671093	3.533547	1.663196
75	1	0	-3.673390	1.828344	2.036070
76	6	0	-1.200527	0.203785	2.753838
77	1	0	-1.034994	-0.688515	3.362315
78	1	0	-0.367675	0.899070	2.900743
79	1	0	-2.129952	0.680220	3.089974
80	6	0	4.867109	2.859479	0.146771
81	1	0	5.865475	2.651801	0.543658
82	1	0	4.994969	3.281243	-0.856522
83	1	0	4.433446	3.634139	0.783966
84	1	0	-7.691462	-1.631991	1.465851
85	1	0	-8.461718	-3.069833	0.721812
86	1	0	-7.256760	-3.266003	2.035377

24

Center Number	Atomic Number	Atomic Type	Coordinates (Angstroms) X	Y	Z
1	6	0	-0.857083	0.070231	-1.175260
2	6	0	0.501601	0.800829	-1.083405
3	1	0	-0.862242	-0.466929	-2.127369
4	1	0	-1.659742	0.813770	-1.232777
5	1	0	0.516560	1.507948	-0.249416
6	1	0	0.642340	1.383978	-2.001455
7	6	0	3.853823	-0.881540	-0.146653
8	6	0	2.776368	-1.913763	0.280835
9	6	0	1.479945	-1.030986	0.380963
10	6	0	1.610617	-0.242148	-0.940416
11	6	0	3.217758	-2.701616	1.493751
12	1	0	2.575866	-2.646248	-0.508052
13	8	0	1.581625	-0.065055	1.447274
14	6	0	-1.635639	-3.254113	0.603961
15	6	0	-2.169414	-2.116401	-0.327785
16	6	0	-1.138664	-0.916070	-0.001908
17	6	0	0.130026	-1.712668	0.392042
18	6	0	-0.165905	-2.970041	0.737871
19	1	0	-2.120115	-3.225540	1.592056
20	1	0	-1.834798	-4.251091	0.194604
21	6	0	-2.007281	-2.613675	-1.782800
22	6	0	-1.599249	-0.087775	1.226688
23	1	0	0.550767	-3.705851	1.087599
24	1	0	-2.356822	-1.891047	-2.524598
25	1	0	-0.959874	-2.839422	-2.005648
26	1	0	-2.576240	-3.536159	-1.931964
27	1	0	-0.830222	0.636806	1.501637
28	1	0	-2.517076	0.467844	1.015986
29	1	0	-1.783987	-0.721734	2.099385
30	6	0	-3.677361	-1.817104	-0.016685
31	6	0	-4.285328	-0.714114	-0.915700
32	6	0	-5.670121	-0.213301	-0.500625
33	6	0	-6.191305	0.974387	-1.335328
34	6	0	-5.792530	2.209202	-0.512518
35	6	0	-5.702460	1.652607	0.904529
36	8	0	-5.631390	0.301460	0.874869
37	1	0	-6.390026	-1.032814	-0.476903
38	1	0	-7.282019	0.922897	-1.422642
39	1	0	-5.772738	0.977409	-2.344799
40	1	0	-4.768956	2.516478	-0.770546
41	6	0	-6.713571	3.425003	-0.627408
42	1	0	-3.721643	-1.457567	1.016412
43	1	0	-4.389366	-1.088217	-1.942478
44	1	0	-3.614881	0.149387	-0.970733
45	8	0	-5.678437	2.269237	1.949838
46	1	0	-6.387667	4.222075	0.046272
47	1	0	-6.704618	3.812918	-1.650511
48	1	0	-7.744957	3.160645	-0.371034
49	1	0	1.396609	-0.997817	-1.709045
50	6	0	3.123673	0.116363	-1.148668
51	6	0	3.474469	-0.190213	-2.622183

52	6	0	3.493357	1.612188	-0.928971
53	1	0	4.516692	0.047849	-2.854886
54	1	0	3.314184	-1.245930	-2.859942
55	1	0	2.839731	0.405295	-3.288971
56	6	0	3.372832	2.224553	0.484907
57	6	0	3.781902	3.678778	0.448772
58	8	0	4.931825	4.085085	0.491413
59	8	0	2.718785	4.502259	0.330994
60	6	0	3.003642	5.915511	0.241430
61	6	0	5.177653	-1.473564	-0.608071
62	1	0	4.081085	-0.305353	0.758825
63	6	0	5.321122	-2.741410	-1.023755
64	6	0	6.371698	-0.548201	-0.535106
65	1	0	6.292911	-3.120377	-1.328868
66	1	0	4.495287	-3.443105	-1.079564
67	1	0	7.290846	-1.058152	-0.835702
68	1	0	6.245755	0.327795	-1.181687
69	1	0	6.507663	-0.165510	0.484359
70	1	0	4.049115	1.723620	1.182031
71	1	0	2.358567	2.129845	0.868802
72	1	0	2.872122	2.203201	-1.613348
73	1	0	4.526219	1.757874	-1.264819
74	8	0	2.890189	-3.852697	1.729790
75	1	0	3.913607	-2.179766	2.181864
76	6	0	1.434247	-0.519567	2.789214
77	1	0	1.232011	0.367479	3.394434
78	1	0	0.604091	-1.224697	2.897072
79	1	0	2.353097	-0.988778	3.163187
80	6	0	-4.553607	-3.088023	-0.074513
81	1	0	-5.558025	-2.885301	0.309206
82	1	0	-4.663194	-3.465073	-1.097554
83	1	0	-4.142859	-3.894541	0.537806
84	1	0	3.524140	6.254643	1.139576
85	1	0	3.617991	6.123474	-0.637332
86	1	0	2.032751	6.401333	0.154874

25

Center Number	Atomic Number	Atomic Type	Coordinates (Angstroms) X	Y	Z
1	6	0	-0.966058	0.219807	-1.149856
2	6	0	0.431337	0.869941	-1.053363
3	1	0	-0.986690	-0.355566	-2.079596
4	1	0	-1.719524	1.008601	-1.253721
5	1	0	0.479208	1.590916	-0.232106
6	1	0	0.622562	1.426269	-1.979226
7	6	0	3.666009	-1.084400	-0.132090
8	6	0	2.490962	-1.972951	0.385123
9	6	0	1.281145	-0.975837	0.468404
10	6	0	1.479967	-0.226837	-0.868513
11	6	0	2.895775	-2.729500	1.627301
12	1	0	2.205818	-2.725946	-0.355082
13	8	0	1.462689	0.001633	1.515203
14	6	0	-1.981227	-2.968213	0.756957
15	6	0	-2.431417	-1.838049	-0.226551
16	6	0	-1.325727	-0.695405	0.058597
17	6	0	-0.115337	-1.557692	0.493555
18	6	0	-0.496057	-2.777305	0.888612
19	1	0	-2.466289	-2.862706	1.739604
20	1	0	-2.247378	-3.965961	0.389423
21	6	0	-2.293247	-2.402672	-1.659214
22	6	0	-1.737149	0.213406	1.246706
23	1	0	0.167002	-3.545530	1.273090
24	1	0	-2.593601	-1.689099	-2.430976
25	1	0	-1.260342	-2.700115	-1.864604
26	1	0	-2.917036	-3.293829	-1.776790
27	1	0	-0.917640	0.886634	1.507099
28	1	0	-2.607454	0.828026	1.000198
29	1	0	-1.979952	-0.371283	2.139592
30	6	0	-3.916486	-1.424460	0.056377
31	6	0	-4.446914	-0.342021	-0.914415
32	6	0	-5.723108	0.364987	-0.477452
33	6	0	-6.266801	1.400191	-1.477005
34	6	0	-6.903504	2.475645	-0.580782
35	6	0	-6.129474	2.319206	0.725198
36	8	0	-5.460361	1.142950	0.740743
37	1	0	-6.501839	-0.355988	-0.208253
38	1	0	-6.973807	0.949596	-2.178813
39	1	0	-5.438678	1.824584	-2.055264
40	1	0	-6.727369	3.487013	-0.958365
41	6	0	-8.414033	2.294053	-0.336038
42	1	0	-3.939962	-1.001418	1.066170
43	1	0	-4.657848	-0.793380	-1.893120
44	1	0	-3.693189	0.431211	-1.088047
45	8	0	-6.087724	3.089483	1.662894
46	1	0	-8.779094	3.026292	0.389597
47	1	0	-8.960249	2.435423	-1.273463
48	1	0	-8.643993	1.292580	0.043440
49	1	0	1.261795	-0.986537	-1.633079
50	6	0	3.003846	0.076273	-1.015066
51	6	0	3.391948	0.047761	-2.507684

52	6	0	3.357938	1.474132	-0.433215
53	1	0	4.475959	0.042065	-2.652009
54	1	0	2.985877	-0.827678	-3.021490
55	1	0	2.992330	0.934134	-3.014150
56	6	0	4.831505	1.883516	-0.543957
57	6	0	5.104043	3.260321	0.023921
58	8	0	4.267436	4.109317	0.276068
59	8	0	6.427965	3.459857	0.205022
60	6	0	6.822591	4.758655	0.697981
61	6	0	4.800324	-1.905897	-0.737114
62	1	0	4.084796	-0.587443	0.750053
63	6	0	6.070344	-1.696051	-0.356531
64	6	0	4.474062	-3.014507	-1.715979
65	1	0	6.890752	-2.276111	-0.771141
66	1	0	6.332784	-0.945858	0.384748
67	1	0	5.382357	-3.385326	-2.198531
68	1	0	4.003958	-3.863358	-1.204009
69	1	0	3.779216	-2.694320	-2.497154
70	1	0	5.163976	1.913226	-1.589290
71	1	0	5.499047	1.177232	-0.042626
72	1	0	3.052312	1.510477	0.614808
73	1	0	2.758220	2.227156	-0.955249
74	8	0	2.577096	-3.882266	1.870095
75	1	0	3.564173	-2.188695	2.327229
76	6	0	1.232895	-0.392686	2.864817
77	1	0	1.110793	0.530209	3.436941
78	1	0	0.329934	-1.001924	2.969303
79	1	0	2.085647	-0.944802	3.279575
80	6	0	-4.874464	-2.635798	0.067683
81	1	0	-5.879440	-2.334129	0.378984
82	1	0	-4.965154	-3.096665	-0.922183
83	1	0	-4.547744	-3.407652	0.769063
84	1	0	7.908670	4.722255	0.771026
85	1	0	6.510358	5.540105	0.001724
86	1	0	6.377797	4.943866	1.678034

26

Center Number	Atomic Number	Atomic Type	Coordinates (Angstroms) X	Y	Z
1	6	0	-0.897902	0.202554	-1.136628
2	6	0	0.487495	0.873648	-1.014341
3	1	0	-0.903076	-0.349032	-2.080755
4	1	0	-1.664019	0.980957	-1.224972
5	1	0	0.516592	1.572572	-0.173267
6	1	0	0.674305	1.457801	-1.923872
7	6	0	3.750322	-1.053489	-0.127155
8	6	0	2.586264	-1.972103	0.361563
9	6	0	1.359813	-0.997435	0.463193
10	6	0	1.554373	-0.209162	-0.851683
11	6	0	2.997246	-2.754698	1.585926
12	1	0	2.317059	-2.710030	-0.399495
13	8	0	1.518767	-0.044192	1.535520
14	6	0	-1.870263	-3.050621	0.679151
15	6	0	-2.334615	-1.901731	-0.275657
16	6	0	-1.248942	-0.749311	0.045459
17	6	0	-0.026987	-1.602685	0.464254
18	6	0	-0.389205	-2.838664	0.824136
19	1	0	-2.361766	-2.980169	1.661745
20	1	0	-2.117851	-4.042382	0.283504
21	6	0	-2.181533	-2.424736	-1.722521
22	6	0	-1.682360	0.121718	1.253868
23	1	0	0.285023	-3.605572	1.191520
24	1	0	-2.488174	-1.694338	-2.475857
25	1	0	-1.143738	-2.702580	-1.930736
26	1	0	-2.792286	-3.320740	-1.867724
27	1	0	-0.874382	0.799013	1.538297
28	1	0	-2.559678	0.730018	1.016721
29	1	0	-1.922932	-0.489525	2.129406
30	6	0	-3.827670	-1.520101	0.010676
31	6	0	-4.373422	-0.424699	-0.937320
32	6	0	-5.648781	0.268022	-0.475548
33	6	0	-6.219808	1.301950	-1.461080
34	6	0	-6.849095	2.368992	-0.549717
35	6	0	-6.049674	2.215000	0.741112
36	8	0	-5.371602	1.043849	0.740949
37	1	0	-6.415459	-0.461454	-0.194971
38	1	0	-6.935559	0.847523	-2.151462
39	1	0	-5.406100	1.735454	-2.052945
40	1	0	-6.688384	3.383019	-0.927105
41	6	0	-8.353361	2.174508	-0.277660
42	1	0	-3.862996	-1.122059	1.030180
43	1	0	-4.591422	-0.862211	-1.920751
44	1	0	-3.626304	0.355951	-1.106627
45	8	0	-5.995691	2.983406	1.679763
46	1	0	-8.710362	2.901318	0.457388
47	1	0	-8.918038	2.315251	-1.204176
48	1	0	-8.568785	1.169982	0.102267
49	1	0	1.356196	-0.950636	-1.639230
50	6	0	3.074342	0.123455	-0.976722
51	6	0	3.476906	0.155556	-2.464628

52	6	0	3.397988	1.505037	-0.340270
53	1	0	4.562242	0.175975	-2.596553
54	1	0	3.093056	-0.709459	-3.011812
55	1	0	3.065802	1.051300	-2.944647
56	6	0	4.873580	1.958029	-0.443105
57	6	0	5.076362	3.253994	0.306361
58	8	0	5.353387	3.342975	1.490989
59	8	0	4.880888	4.330914	-0.483666
60	6	0	4.996754	5.624460	0.148489
61	6	0	4.894469	-1.842972	-0.756372
62	1	0	4.163684	-0.577328	0.768777
63	6	0	6.161887	-1.628608	-0.369901
64	6	0	4.580764	-2.928091	-1.765378
65	1	0	6.988717	-2.187430	-0.800667
66	1	0	6.415553	-0.897219	0.392855
67	1	0	5.493001	-3.273767	-2.258998
68	1	0	4.122401	-3.796738	-1.276425
69	1	0	3.880548	-2.596072	-2.536860
70	1	0	5.166214	2.100225	-1.486004
71	1	0	5.538384	1.218720	0.008108
72	1	0	3.103107	1.485879	0.711321
73	1	0	2.779359	2.263796	-0.833611
74	8	0	2.690529	-3.916575	1.798648
75	1	0	3.658406	-2.224158	2.300245
76	6	0	1.290814	-0.477287	2.873718
77	1	0	0.388738	-1.090546	2.961117
78	1	0	2.144686	-1.039002	3.272475
79	1	0	1.167989	0.429145	3.471276
80	6	0	-4.765196	-2.747163	-0.012200
81	1	0	-5.779927	-2.466943	0.287636
82	1	0	-4.832768	-3.192831	-1.010742
83	1	0	-4.436649	-3.524882	0.681850
84	1	0	4.801723	6.350161	-0.639837
85	1	0	4.262554	5.721046	0.951174
86	1	0	6.001671	5.758778	0.554494

27

Center Number	Atomic Number	Atomic Type	Coordinates (Angstroms) X	Y	Z
1	6	0	-0.758710	0.107201	-1.185526
2	6	0	0.609796	0.820327	-1.102994
3	1	0	-0.769357	-0.445796	-2.128550
4	1	0	-1.549538	0.862034	-1.256763
5	1	0	0.632272	1.542904	-0.282529
6	1	0	0.761134	1.384154	-2.031411
7	6	0	3.936014	-0.888992	-0.125709
8	6	0	2.843462	-1.898242	0.318328
9	6	0	1.558894	-0.995993	0.398243
10	6	0	1.703774	-0.234607	-0.937503
11	6	0	3.270883	-2.668879	1.547042
12	1	0	2.635046	-2.642451	-0.457342
13	8	0	1.671624	-0.011435	1.446037
14	6	0	-1.584791	-3.175293	0.651966
15	6	0	-2.101201	-2.049029	-0.302367
16	6	0	-1.057478	-0.854126	0.004886
17	6	0	0.199772	-1.659149	0.418275
18	6	0	-0.112036	-2.906708	0.785247
19	1	0	-2.071278	-3.121187	1.638110
20	1	0	-1.796646	-4.177165	0.261096
21	6	0	-1.940939	-2.571600	-1.748651
22	6	0	-1.515142	0.004653	1.213321
23	1	0	0.594707	-3.644563	1.150473
24	1	0	-2.286879	-1.858644	-2.501585
25	1	0	-0.894441	-2.805549	-1.967026
26	1	0	-2.513812	-3.493762	-1.883992
27	1	0	-0.738714	0.725755	1.477078
28	1	0	-2.426528	0.564352	0.985980
29	1	0	-1.709210	-0.609865	2.098124
30	6	0	-3.605853	-1.725475	-0.003736
31	6	0	-4.201391	-0.648653	-0.943304
32	6	0	-5.503520	-0.014081	-0.472929
33	6	0	-6.120805	1.003512	-1.447429
34	6	0	-6.791889	2.034776	-0.524692
35	6	0	-5.984477	1.902121	0.763469
36	8	0	-5.256482	0.761653	0.750404
37	1	0	-6.238332	-0.777435	-0.197610
38	1	0	-6.819128	0.525362	-2.139574
39	1	0	-5.328384	1.476186	-2.038204
40	1	0	-6.673686	3.058151	-0.892553
41	6	0	-8.286487	1.776473	-0.252313
42	1	0	-3.648335	-1.332455	1.017468
43	1	0	-4.404405	-1.089360	-1.928510
44	1	0	-3.488721	0.164031	-1.110241
45	8	0	-5.962283	2.662689	1.709856
46	1	0	-8.671191	2.480910	0.490480
47	1	0	-8.858189	1.903221	-1.176528
48	1	0	-8.460484	0.760477	0.118199
49	1	0	1.481218	-1.001720	-1.692238
50	6	0	3.222085	0.099709	-1.148318
51	6	0	3.571182	-0.239474	-2.615197

52	6	0	3.610036	1.594589	-0.956228
53	1	0	4.616886	-0.020459	-2.850960
54	1	0	3.396270	-1.297171	-2.833105
55	1	0	2.945699	0.352244	-3.294044
56	6	0	3.500886	2.233984	0.446489
57	6	0	3.927985	3.682213	0.383318
58	8	0	5.083616	4.074049	0.402582
59	8	0	2.873762	4.517633	0.271219
60	6	0	3.174537	5.925925	0.159335
61	6	0	5.252519	-1.508070	-0.572215
62	1	0	4.169014	-0.299437	0.769664
63	6	0	5.380337	-2.786487	-0.959736
64	6	0	6.458484	-0.596989	-0.517382
65	1	0	6.347698	-3.184242	-1.254820
66	1	0	4.545867	-3.478904	-1.001073
67	1	0	7.371040	-1.124768	-0.807104
68	1	0	6.344028	0.267397	-1.181400
69	1	0	6.599045	-0.195696	0.494278
70	1	0	4.173599	1.737629	1.150480
71	1	0	2.486953	2.159534	0.835759
72	1	0	2.994008	2.180351	-1.649750
73	1	0	4.643661	1.722230	-1.296980
74	8	0	2.928087	-3.811219	1.803393
75	1	0	3.971027	-2.143123	2.227795
76	6	0	1.510938	-0.437204	2.795664
77	1	0	0.667375	-1.124553	2.913635
78	1	0	2.419509	-0.916009	3.182481
79	1	0	1.322878	0.464961	3.382856
80	6	0	-4.495681	-2.987718	-0.024106
81	1	0	-5.516409	-2.749056	0.290997
82	1	0	-4.558872	-3.427773	-1.025397
83	1	0	-4.129160	-3.757595	0.659592
84	1	0	2.208501	6.422503	0.079891
85	1	0	3.711496	6.270065	1.045830
86	1	0	3.779083	6.115057	-0.730389

28

Center Number	Atomic Number	Atomic Type	Coordinates (Angstroms) X	Y	Z
1	6	0	-1.027483	0.161270	-1.106431
2	6	0	0.352304	0.842668	-0.978691
3	1	0	-1.035573	-0.366711	-2.063834
4	1	0	-1.802338	0.933553	-1.169216
5	1	0	0.381554	1.519496	-0.119793
6	1	0	0.525541	1.451836	-1.874306
7	6	0	3.640996	-1.073921	-0.166862
8	6	0	2.489964	-2.015606	0.308361
9	6	0	1.254663	-1.056622	0.443415
10	6	0	1.431052	-0.233479	-0.852552
11	6	0	2.918689	-2.824888	1.509123
12	1	0	2.221696	-2.736669	-0.468950
13	8	0	1.411720	-0.128829	1.538130
14	6	0	-1.953492	-3.147579	0.628885
15	6	0	-2.438629	-1.976073	-0.287702
16	6	0	-1.359275	-0.823468	0.053775
17	6	0	-0.125996	-1.675951	0.439077
18	6	0	-0.473490	-2.924805	0.767364
19	1	0	-2.436905	-3.112156	1.617259
20	1	0	-2.193824	-4.129871	0.206065
21	6	0	-2.297624	-2.458208	-1.749841
22	6	0	-1.788093	0.011408	1.288858
23	1	0	0.211430	-3.694791	1.107691
24	1	0	-2.613114	-1.708333	-2.479934
25	1	0	-1.261233	-2.727837	-1.975451
26	1	0	-2.907930	-3.351367	-1.913288
27	1	0	-0.983826	0.688357	1.583750
28	1	0	-2.672143	0.619014	1.076224
29	1	0	-2.017150	-0.623854	2.150058
30	6	0	-3.931926	-1.615675	0.026252
31	6	0	-4.496584	-0.495071	-0.880010
32	6	0	-5.806317	0.137665	-0.405785
33	6	0	-6.303271	1.303314	-1.285210
34	6	0	-5.752904	2.555904	-0.586042
35	6	0	-5.598472	2.091557	0.858431
36	8	0	-5.624977	0.739685	0.922166
37	1	0	-6.579231	-0.620624	-0.269271
38	1	0	-7.398386	1.331529	-1.289081
39	1	0	-5.965372	1.205956	-2.319860
40	1	0	-4.731399	2.761630	-0.936741
41	6	0	-6.586065	3.830691	-0.727363
42	1	0	-3.959753	-1.250090	1.057713
43	1	0	-4.691805	-0.895702	-1.883291
44	1	0	-3.761058	0.304963	-1.008440
45	8	0	-5.453685	2.773560	1.852052
46	1	0	-6.152189	4.643601	-0.138874
47	1	0	-6.624161	4.145363	-1.774687
48	1	0	-7.612376	3.667915	-0.381697
49	1	0	1.233887	-0.956604	-1.657223
50	6	0	2.946723	0.117561	-0.980833
51	6	0	3.336905	0.190440	-2.470582

52	6	0	3.260915	1.485570	-0.311631
53	1	0	4.420812	0.225068	-2.611254
54	1	0	2.956946	-0.664979	-3.035254
55	1	0	2.913161	1.093428	-2.925432
56	6	0	4.730691	1.957057	-0.413044
57	6	0	4.924696	3.236747	0.366154
58	8	0	5.215963	3.300211	1.548975
59	8	0	4.703463	4.330000	-0.394153
60	6	0	4.809461	5.609127	0.268365
61	6	0	4.786552	-1.836697	-0.825936
62	1	0	4.058505	-0.616335	0.736868
63	6	0	6.055887	-1.616980	-0.448905
64	6	0	4.473533	-2.903525	-1.854584
65	1	0	6.883650	-2.157465	-0.900749
66	1	0	6.310456	-0.899617	0.326733
67	1	0	5.384718	-3.231542	-2.361980
68	1	0	4.025979	-3.785672	-1.379983
69	1	0	3.764674	-2.562264	-2.614156
70	1	0	5.014411	2.127568	-1.454178
71	1	0	5.406620	1.214610	0.015809
72	1	0	2.973783	1.435913	0.741131
73	1	0	2.630657	2.250134	-0.780658
74	8	0	2.623365	-3.994195	1.695421
75	1	0	3.582361	-2.306914	2.230354
76	6	0	1.200348	-0.598445	2.866808
77	1	0	0.304019	-1.221078	2.947059
78	1	0	2.062480	-1.163343	3.242537
79	1	0	1.076244	0.291183	3.488830
80	6	0	-4.860470	-2.848837	-0.024764
81	1	0	-5.867019	-2.592291	0.319916
82	1	0	-4.955256	-3.249551	-1.040115
83	1	0	-4.504265	-3.654247	0.622345
84	1	0	4.596450	6.351260	-0.499752
85	1	0	4.082843	5.676482	1.080907
86	1	0	5.816917	5.746724	0.666862

29

Center Number	Atomic Number	Atomic Type	Coordinates (Angstroms) X	Y	Z
1	6	0	-0.941090	0.163596	-1.182302
2	6	0	0.446077	0.840901	-1.145425
3	1	0	-0.967593	-0.457934	-2.081639
4	1	0	-1.710302	0.933133	-1.311750
5	1	0	0.496138	1.601684	-0.361224
6	1	0	0.610347	1.352954	-2.101383
7	6	0	3.730798	-1.014672	-0.191099
8	6	0	2.580225	-1.887070	0.402495
9	6	0	1.355343	-0.906633	0.454791
10	6	0	1.515677	-0.228852	-0.924401
11	6	0	3.022326	-2.567394	1.676073
12	1	0	2.290147	-2.685149	-0.286855
13	8	0	1.536493	0.130355	1.443224
14	6	0	-1.862603	-2.941639	0.900906
15	6	0	-2.350213	-1.872435	-0.131253
16	6	0	-1.263223	-0.695644	0.077145
17	6	0	-0.029442	-1.510799	0.536246
18	6	0	-0.380000	-2.715624	0.999051
19	1	0	-2.334906	-2.797059	1.884676
20	1	0	-2.113511	-3.961662	0.587845
21	6	0	-2.220604	-2.506919	-1.535378
22	6	0	-1.675007	0.264228	1.224284
23	1	0	0.303705	-3.450743	1.411125
24	1	0	-2.544654	-1.838737	-2.337499
25	1	0	-1.184682	-2.794195	-1.740183
26	1	0	-2.827826	-3.414872	-1.599457
27	1	0	-0.867975	0.969522	1.434993
28	1	0	-2.564431	0.844186	0.962704
29	1	0	-1.887787	-0.279523	2.150065
30	6	0	-3.839301	-1.473951	0.153002
31	6	0	-4.405666	-0.456236	-0.866087
32	6	0	-5.697360	0.237644	-0.455038
33	6	0	-6.272763	1.210230	-1.498599
34	6	0	-6.934821	2.308065	-0.649221
35	6	0	-6.149066	2.234858	0.657044
36	8	0	-5.448585	1.078809	0.723709
37	1	0	-6.456074	-0.489359	-0.147861
38	1	0	-6.970421	0.708965	-2.174851
39	1	0	-5.459055	1.629722	-2.100397
40	1	0	-6.790062	3.305273	-1.074818
41	6	0	-8.437985	2.094972	-0.385471
42	1	0	-3.856613	-0.996485	1.138480
43	1	0	-4.610613	-0.961093	-1.819522
44	1	0	-3.673413	0.326746	-1.082666
45	8	0	-6.122249	3.050056	1.556577
46	1	0	-8.819271	2.848009	0.309867
47	1	0	-8.993587	2.179166	-1.324225
48	1	0	-8.637021	1.105233	0.039448
49	1	0	1.294660	-1.031362	-1.642814
50	6	0	3.031644	0.086274	-1.120008
51	6	0	3.389086	-0.012796	-2.616512

52	6	0	3.375574	1.517375	-0.617952
53	1	0	4.469685	-0.009627	-2.783677
54	1	0	2.986157	-0.919946	-3.074433
55	1	0	2.965438	0.840102	-3.159408
56	6	0	4.848323	1.951438	-0.803275
57	6	0	5.047783	3.353730	-0.277659
58	8	0	4.748182	4.373014	-0.877314
59	8	0	5.574162	3.362003	0.964975
60	6	0	5.756776	4.656614	1.579481
61	6	0	4.861217	-1.854742	-0.778071
62	1	0	4.165566	-0.464578	0.650703
63	6	0	6.136355	-1.608126	-0.438756
64	6	0	4.528852	-3.019054	-1.687613
65	1	0	6.955283	-2.199617	-0.839879
66	1	0	6.404246	-0.814804	0.254039
67	1	0	5.431702	-3.404385	-2.168970
68	1	0	4.081457	-3.844733	-1.120404
69	1	0	3.813341	-2.751241	-2.469847
70	1	0	5.114595	1.965146	-1.863166
71	1	0	5.526436	1.272334	-0.283444
72	1	0	3.106228	1.594062	0.438545
73	1	0	2.748291	2.233311	-1.160881
74	8	0	2.723991	-3.708389	1.989568
75	1	0	3.698473	-1.980717	2.329997
76	6	0	1.346959	-0.197092	2.816884
77	1	0	1.203115	0.750541	3.341609
78	1	0	0.468353	-0.830485	2.972987
79	1	0	2.227143	-0.695727	3.241632
80	6	0	-4.771058	-2.702058	0.245746
81	1	0	-5.776738	-2.404754	0.558957
82	1	0	-4.869038	-3.216970	-0.716467
83	1	0	-4.416663	-3.428766	0.981097
84	1	0	6.193121	4.453893	2.556500
85	1	0	6.430056	5.270119	0.977169
86	1	0	4.795707	5.164498	1.685196

30

Center Number	Atomic Number	Atomic Type	Coordinates (Angstroms) X	Y	Z
1	6	0	-0.908224	0.522189	-0.759024
2	6	0	0.435120	1.081082	-0.242622
3	1	0	-0.796259	0.380403	-1.837285
4	1	0	-1.683907	1.284743	-0.625136
5	1	0	0.355740	1.417563	0.795768
6	1	0	0.683748	1.957517	-0.850805
7	6	0	3.620892	-1.064099	0.310848
8	6	0	2.469202	-2.091960	0.179686
9	6	0	1.198356	-1.224776	0.491889
10	6	0	1.519110	0.010193	-0.381814
11	6	0	2.746702	-3.325239	1.009957
12	1	0	2.340897	-2.436038	-0.851224
13	8	0	1.194784	-0.753585	1.858160
14	6	0	-1.962909	-3.165263	-0.478799
15	6	0	-2.363150	-1.728259	-0.949589
16	6	0	-1.354856	-0.808418	-0.086499
17	6	0	-0.156370	-1.770628	0.098524
18	6	0	-0.513514	-3.044346	-0.099327
19	1	0	-2.556654	-3.485067	0.391336
20	1	0	-2.132497	-3.917540	-1.257826
21	6	0	-2.042860	-1.643055	-2.459594
22	6	0	-1.940406	-0.476475	1.311316
23	1	0	0.142958	-3.901235	0.008652
24	1	0	-2.288147	-0.670729	-2.894197
25	1	0	-0.981341	-1.833539	-2.645299
26	1	0	-2.609196	-2.400527	-3.009619
27	1	0	-1.191837	0.031504	1.922737
28	1	0	-2.811215	0.180597	1.236418
29	1	0	-2.246865	-1.379935	1.847534
30	6	0	-3.890065	-1.473779	-0.698260
31	6	0	-4.367892	-0.081159	-1.174127
32	6	0	-5.756748	0.338824	-0.689323
33	6	0	-6.165953	1.768983	-1.097831
34	6	0	-5.785706	2.626623	0.119070
35	6	0	-5.822354	1.619190	1.263696
36	8	0	-5.797030	0.355976	0.778840
37	1	0	-6.510368	-0.392854	-0.984914
38	1	0	-7.247732	1.818106	-1.263007
39	1	0	-5.670061	2.086443	-2.018396
40	1	0	-4.735304	2.941771	0.041601
41	6	0	-6.651356	3.862029	0.371653
42	1	0	-4.043352	-1.521554	0.384755
43	1	0	-4.405271	-0.057323	-2.270907
44	1	0	-3.654886	0.693503	-0.875141
45	8	0	-5.854734	1.843146	2.456375
46	1	0	-6.347601	4.365931	1.293154
47	1	0	-6.553314	4.570036	-0.456876
48	1	0	-7.707547	3.587488	0.463428
49	1	0	1.437466	-0.364617	-1.412054
50	6	0	3.039944	0.339379	-0.189984
51	6	0	3.620522	0.768375	-1.550327

52	6	0	3.342738	1.397909	0.913192
53	1	0	4.679561	1.034703	-1.486601
54	1	0	3.528988	-0.035176	-2.286968
55	1	0	3.080018	1.636518	-1.942302
56	6	0	3.148645	2.893745	0.580682
57	6	0	4.225690	3.575118	-0.238875
58	8	0	4.013499	4.384548	-1.126620
59	8	0	5.469807	3.240032	0.160010
60	6	0	6.563755	3.892084	-0.520941
61	6	0	4.955829	-1.505407	-0.272420
62	1	0	3.787468	-0.944967	1.390271
63	6	0	5.079674	-2.455516	-1.212022
64	6	0	6.181611	-0.831952	0.304217
65	1	0	6.058315	-2.742773	-1.587359
66	1	0	4.230878	-2.977951	-1.640841
67	1	0	6.240536	-0.997804	1.387841
68	1	0	7.098433	-1.215879	-0.151505
69	1	0	6.157953	0.253539	0.155972
70	1	0	3.124166	3.441962	1.532623
71	1	0	2.200906	3.099062	0.083738
72	1	0	4.378865	1.269302	1.240458
73	1	0	2.717236	1.162163	1.777924
74	8	0	2.383784	-4.453722	0.722532
75	1	0	3.358510	-3.164981	1.921320
76	6	0	0.884320	-1.683542	2.891480
77	1	0	0.037258	-2.325284	2.628615
78	1	0	1.746710	-2.314243	3.143035
79	1	0	0.624857	-1.091025	3.772201
80	6	0	-4.779525	-2.575310	-1.315406
81	1	0	-5.823435	-2.448232	-1.012630
82	1	0	-4.752183	-2.558707	-2.410757
83	1	0	-4.477851	-3.573036	-0.986914
84	1	0	7.467949	3.498357	-0.058717
85	1	0	6.542002	3.655277	-1.586867
86	1	0	6.503192	4.974127	-0.386153

31

Center Number	Atomic Number	Atomic Type	Coordinates (Angstroms) X	Y	Z
1	6	0	-1.022266	0.154088	-1.108607
2	6	0	0.360782	0.825176	-0.967454
3	1	0	-1.030652	-0.361880	-2.072506
4	1	0	-1.791844	0.932236	-1.164153
5	1	0	0.390016	1.490570	-0.099808
6	1	0	0.539943	1.445319	-1.854411
7	6	0	3.635616	-1.126057	-0.178343
8	6	0	2.477341	-2.060656	0.280862
9	6	0	1.247416	-1.099704	0.431213
10	6	0	1.433283	-0.258766	-0.851198
11	6	0	2.899827	-2.905406	1.461301
12	1	0	2.194682	-2.761885	-0.507967
13	8	0	1.406053	-0.190978	1.541653
14	6	0	-1.977582	-3.169784	0.578922
15	6	0	-2.451630	-1.981664	-0.321700
16	6	0	-1.363737	-0.842797	0.037989
17	6	0	-0.137905	-1.710076	0.413980
18	6	0	-0.496168	-2.960642	0.723888
19	1	0	-2.462989	-3.145196	1.566663
20	1	0	-2.224574	-4.144021	0.141385
21	6	0	-2.311470	-2.444699	-1.790187
22	6	0	-1.787956	-0.021894	1.283940
23	1	0	0.182516	-3.739739	1.055257
24	1	0	-2.618335	-1.681984	-2.510590
25	1	0	-1.276944	-2.720422	-2.017036
26	1	0	-2.929277	-3.330072	-1.967265
27	1	0	-0.976568	0.640194	1.592674
28	1	0	-2.664137	0.599334	1.077436
29	1	0	-2.027567	-0.668112	2.134106
30	6	0	-3.942587	-1.613269	-0.006029
31	6	0	-4.495821	-0.476780	-0.899391
32	6	0	-5.801063	0.161750	-0.420635
33	6	0	-6.282761	1.344690	-1.285294
34	6	0	-5.719501	2.580983	-0.567719
35	6	0	-5.577021	2.095620	0.871022
36	8	0	-5.618878	0.743347	0.916299
37	1	0	-6.581952	-0.590663	-0.297711
38	1	0	-7.377485	1.385635	-1.291086
39	1	0	-5.943633	1.257569	-2.320450
40	1	0	-4.693892	2.777833	-0.911594
41	6	0	-6.535322	3.868281	-0.695443
42	1	0	-3.969636	-1.260224	1.029824
43	1	0	-4.692207	-0.863158	-1.908038
44	1	0	-3.753111	0.318313	-1.016216
45	8	0	-5.429395	2.762584	1.874379
46	1	0	-6.093717	4.667280	-0.093783
47	1	0	-6.564317	4.197846	-1.738477
48	1	0	-7.565311	3.714278	-0.356769
49	1	0	1.238850	-0.967943	-1.668717
50	6	0	2.952305	0.092571	-0.964742
51	6	0	3.335177	0.155681	-2.455334

52	6	0	3.237624	1.460351	-0.282956
53	1	0	4.396961	0.364882	-2.612097
54	1	0	3.112767	-0.786683	-2.963713
55	1	0	2.766471	0.950172	-2.952426
56	6	0	4.710601	1.926466	-0.249766
57	6	0	4.819157	3.259975	0.453246
58	8	0	4.938079	3.407901	1.657992
59	8	0	4.737287	4.294632	-0.409257
60	6	0	4.778867	5.619277	0.165801
61	6	0	4.772808	-1.868167	-0.880960
62	1	0	4.066808	-0.697776	0.736338
63	6	0	4.603155	-3.003960	-1.575357
64	6	0	6.157914	-1.276787	-0.739263
65	1	0	5.448319	-3.494829	-2.050324
66	1	0	3.640082	-3.486552	-1.704140
67	1	0	6.913046	-1.928514	-1.186808
68	1	0	6.234734	-0.296868	-1.223147
69	1	0	6.415880	-1.128829	0.317184
70	1	0	5.117791	2.023508	-1.259035
71	1	0	5.324027	1.221767	0.315144
72	1	0	2.861820	1.422289	0.741743
73	1	0	2.656315	2.225674	-0.810597
74	8	0	2.531524	-4.050866	1.661519
75	1	0	3.626379	-2.435500	2.155879
76	6	0	1.215072	-0.693608	2.860645
77	1	0	2.077095	-1.280142	3.202970
78	1	0	1.116002	0.180219	3.508901
79	1	0	0.312034	-1.306793	2.942049
80	6	0	-4.880921	-2.838070	-0.074528
81	1	0	-5.886554	-2.577515	0.269858
82	1	0	-4.975452	-3.226127	-1.094818
83	1	0	-4.532948	-3.653747	0.564148
84	1	0	4.701112	6.303183	-0.678184
85	1	0	3.942597	5.760263	0.853894
86	1	0	5.720109	5.771107	0.698304

32

Center Number	Atomic Number	Atomic Type	Coordinates (Angstroms) X	Y	Z
1	6	0	-1.003255	0.555487	-0.839329
2	6	0	0.341193	1.137406	-0.354764
3	1	0	-0.889754	0.330068	-1.903161
4	1	0	-1.775884	1.329156	-0.766090
5	1	0	0.257984	1.530424	0.662698
6	1	0	0.612152	1.980089	-1.002311
7	6	0	3.541402	-1.011776	0.203053
8	6	0	2.350670	-2.015798	0.263914
9	6	0	1.096432	-1.117468	0.539657
10	6	0	1.411640	0.047285	-0.425208
11	6	0	2.657385	-3.160344	1.202726
12	1	0	2.164875	-2.468916	-0.712768
13	8	0	1.111238	-0.565334	1.874184
14	6	0	-2.086466	-3.094184	-0.280089
15	6	0	-2.475436	-1.692326	-0.855767
16	6	0	-1.457090	-0.718403	-0.066157
17	6	0	-0.267200	-1.675933	0.191121
18	6	0	-0.636279	-2.957292	0.089925
19	1	0	-2.683506	-3.345577	0.610010
20	1	0	-2.261382	-3.900167	-1.002196
21	6	0	-2.158485	-1.723780	-2.368497
22	6	0	-2.037192	-0.276254	1.303143
23	1	0	0.013069	-3.809437	0.262483
24	1	0	-2.400083	-0.786522	-2.876348
25	1	0	-1.098251	-1.932785	-2.541027
26	1	0	-2.729720	-2.518318	-2.857762
27	1	0	-1.280836	0.262363	1.877575
28	1	0	-2.896456	0.388779	1.179449
29	1	0	-2.360031	-1.132378	1.903481
30	6	0	-3.999853	-1.408496	-0.621011
31	6	0	-4.464544	-0.042616	-1.180026
32	6	0	-5.865539	0.401562	-0.755092
33	6	0	-6.245910	1.823937	-1.214713
34	6	0	-5.890814	2.709816	-0.010437
35	6	0	-5.983271	1.735477	1.159288
36	8	0	-5.957622	0.459095	0.709829
37	1	0	-6.615212	-0.331169	-1.057910
38	1	0	-7.321945	1.880557	-1.412176
39	1	0	-5.720448	2.110934	-2.128953
40	1	0	-4.832676	3.003965	-0.061604
41	6	0	-6.741186	3.966952	0.178253
42	1	0	-4.153867	-1.388299	0.462756
43	1	0	-4.472897	-0.074096	-2.277222
44	1	0	-3.758637	0.745893	-0.901670
45	8	0	-6.056402	1.992404	2.343438
46	1	0	-6.459802	4.491215	1.095563
47	1	0	-6.601492	4.649687	-0.665388
48	1	0	-7.804929	3.714232	0.240941
49	1	0	1.320648	-0.403217	-1.424254
50	6	0	2.927584	0.394224	-0.268278
51	6	0	3.470060	0.868935	-1.628434

52	6	0	3.116273	1.498456	0.810744
53	1	0	4.542519	1.083119	-1.601689
54	1	0	3.314851	0.112150	-2.402526
55	1	0	2.957663	1.785897	-1.938190
56	6	0	4.547062	1.981332	1.111923
57	6	0	5.087675	3.019518	0.150669
58	8	0	4.446066	3.943174	-0.318677
59	8	0	6.403217	2.840030	-0.095917
60	6	0	7.041940	3.822301	-0.941525
61	6	0	4.745562	-1.558340	-0.566479
62	1	0	3.879339	-0.865827	1.237787
63	6	0	4.645602	-2.427784	-1.583874
64	6	0	6.111313	-1.094721	-0.110285
65	1	0	5.534481	-2.791449	-2.092303
66	1	0	3.698605	-2.805805	-1.954419
67	1	0	6.905918	-1.636554	-0.630460
68	1	0	6.263870	-0.025605	-0.295585
69	1	0	6.239747	-1.251099	0.968473
70	1	0	5.260742	1.161409	1.204520
71	1	0	4.535507	2.484471	2.088441
72	1	0	2.680660	1.133636	1.742535
73	1	0	2.532622	2.376485	0.515228
74	8	0	2.296027	-4.312951	1.033287
75	1	0	3.291283	-2.910265	2.078684
76	6	0	0.772885	-1.420096	2.961874
77	1	0	0.596754	-0.767197	3.820098
78	1	0	-0.130734	-2.004593	2.762760
79	1	0	1.591737	-2.106221	3.213008
80	6	0	-4.900392	-2.537245	-1.169424
81	1	0	-5.937979	-2.396455	-0.851569
82	1	0	-4.894642	-2.570835	-2.264830
83	1	0	-4.591306	-3.518962	-0.802006
84	1	0	6.963149	4.815601	-0.494520
85	1	0	8.084002	3.512634	-1.007268
86	1	0	6.579492	3.828644	-1.930837

33

Center Number	Atomic Number	Atomic Type	Coordinates (Angstroms) X	Y	Z
1	6	0	-0.768233	0.227861	-1.107686
2	6	0	0.620877	0.868144	-0.893871
3	1	0	-0.750593	-0.241286	-2.095187
4	1	0	-1.521058	1.022789	-1.153563
5	1	0	0.634576	1.506096	-0.004949
6	1	0	0.828243	1.509318	-1.757843
7	6	0	3.818962	-1.130989	0.031232
8	6	0	2.647562	-2.106922	0.305559
9	6	0	1.419207	-1.136909	0.441477
10	6	0	1.677626	-0.233069	-0.785706
11	6	0	2.976752	-3.043305	1.446286
12	1	0	2.426858	-2.739100	-0.560079
13	8	0	1.544296	-0.278054	1.596179
14	6	0	-1.833623	-3.164000	0.353608
15	6	0	-2.263367	-1.915150	-0.484701
16	6	0	-1.169127	-0.817440	-0.026712
17	6	0	0.028986	-1.724306	0.340252
18	6	0	-0.355759	-2.986137	0.560360
19	1	0	-2.351346	-3.195291	1.324600
20	1	0	-2.077742	-4.105008	-0.153054
21	6	0	-2.089127	-2.290210	-1.974584
22	6	0	-1.624543	-0.054233	1.244560
23	1	0	0.300122	-3.794452	0.865671
24	1	0	-2.367202	-1.480323	-2.654101
25	1	0	-1.052034	-2.565028	-2.190805
26	1	0	-2.711348	-3.155197	-2.222742
27	1	0	-0.816772	0.582799	1.610080
28	1	0	-2.488676	0.584821	1.042317
29	1	0	-1.895249	-0.740306	2.053313
30	6	0	-3.757138	-1.544230	-0.187160
31	6	0	-4.265622	-0.336415	-1.011305
32	6	0	-5.566430	0.289572	-0.525732
33	6	0	-6.072914	1.475434	-1.364436
34	6	0	-6.766286	2.381963	-0.333529
35	6	0	-6.050674	2.014764	0.963460
36	8	0	-5.367689	0.855843	0.815037
37	1	0	-6.350358	-0.466926	-0.416907
38	1	0	-6.739049	1.147127	-2.166921
39	1	0	-5.222557	1.994372	-1.820455
40	1	0	-6.587945	3.443354	-0.528663
41	6	0	-8.283793	2.151420	-0.196103
42	1	0	-3.807914	-1.264062	0.870240
43	1	0	-4.435341	-0.642339	-2.052359
44	1	0	-3.514631	0.458165	-1.040954
45	8	0	-6.061258	2.618924	2.016676
46	1	0	-8.693644	2.748924	0.623053
47	1	0	-8.787358	2.444382	-1.122227
48	1	0	-8.516396	1.098900	-0.001437
49	1	0	1.506618	-0.895781	-1.646311
50	6	0	3.211331	0.082468	-0.818996
51	6	0	3.681638	0.071521	-2.285575

52	6	0	3.634599	1.405246	-0.113275
53	1	0	4.736149	0.348070	-2.386974
54	1	0	3.555233	-0.919533	-2.730507
55	1	0	3.095223	0.778560	-2.882522
56	6	0	3.420877	2.724630	-0.893759
57	6	0	3.910839	3.905543	-0.086995
58	8	0	3.203658	4.660728	0.558628
59	8	0	5.254799	4.025544	-0.139353
60	6	0	5.838545	5.097150	0.632900
61	6	0	5.092972	-1.774323	-0.497557
62	1	0	4.079102	-0.702919	1.008803
63	6	0	5.124762	-2.965700	-1.114343
64	6	0	6.372952	-1.005608	-0.252930
65	1	0	6.064331	-3.387495	-1.461291
66	1	0	4.236914	-3.561929	-1.297554
67	1	0	7.243454	-1.553747	-0.623400
68	1	0	6.359466	-0.025404	-0.743138
69	1	0	6.514839	-0.817423	0.819068
70	1	0	2.366627	2.899934	-1.106260
71	1	0	3.970411	2.700441	-1.838949
72	1	0	4.704971	1.334435	0.109119
73	1	0	3.115475	1.464903	0.847626
74	8	0	2.582146	-4.193953	1.537132
75	1	0	3.657802	-2.637760	2.222360
76	6	0	1.303154	-0.837145	2.883870
77	1	0	0.416850	-1.479397	2.895597
78	1	0	2.165639	-1.411355	3.246230
79	1	0	1.143740	0.005983	3.560334
80	6	0	-4.709823	-2.745871	-0.369301
81	1	0	-5.720468	-2.496212	-0.031530
82	1	0	-4.783452	-3.055313	-1.417808
83	1	0	-4.388709	-3.611767	0.215008
84	1	0	6.911800	5.025741	0.461959
85	1	0	5.458206	6.061717	0.289987
86	1	0	5.608476	4.969744	1.692887

34

Center Number	Atomic Number	Atomic Type	Coordinates (Angstroms) X	Y	Z
1	6	0	-0.986992	0.563204	-0.819840
2	6	0	0.360547	1.134180	-0.330063
3	1	0	-0.875509	0.351990	-1.886773
4	1	0	-1.756917	1.338412	-0.735337
5	1	0	0.281734	1.516445	0.691882
6	1	0	0.634624	1.982317	-0.969131
7	6	0	3.546541	-1.017599	0.253454
8	6	0	2.360195	-2.032120	0.271448
9	6	0	1.105268	-1.130115	0.543930
10	6	0	1.425413	0.040013	-0.413268
11	6	0	2.650565	-3.182740	1.205363
12	1	0	2.199644	-2.471334	-0.717430
13	8	0	1.120993	-0.581976	1.879884
14	6	0	-2.077354	-3.092718	-0.305287
15	6	0	-2.461656	-1.684032	-0.867957
16	6	0	-1.445606	-0.719775	-0.063545
17	6	0	-0.258226	-1.682191	0.188523
18	6	0	-0.628629	-2.961850	0.072581
19	1	0	-2.678745	-3.352411	0.579443
20	1	0	-2.250119	-3.890788	-1.036613
21	6	0	-2.138062	-1.699533	-2.379409
22	6	0	-2.031638	-0.291468	1.307652
23	1	0	0.018108	-3.817510	0.238033
24	1	0	-2.377704	-0.756851	-2.878042
25	1	0	-1.077088	-1.906715	-2.549611
26	1	0	-2.707117	-2.488743	-2.879686
27	1	0	-1.278680	0.243689	1.889738
28	1	0	-2.891200	0.373446	1.186321
29	1	0	-2.356008	-1.153186	1.899060
30	6	0	-3.986735	-1.401216	-0.636568
31	6	0	-4.448126	-0.028545	-1.181476
32	6	0	-5.849139	0.412762	-0.753526
33	6	0	-6.227872	1.840082	-1.199052
34	6	0	-5.875456	2.713405	0.015164
35	6	0	-5.968580	1.726766	1.174472
36	8	0	-5.942731	0.455114	0.711746
37	1	0	-6.599143	-0.316132	-1.064691
38	1	0	-7.303391	1.899084	-1.398552
39	1	0	-5.700071	2.136332	-2.108964
40	1	0	-4.817671	3.009539	-0.031187
41	6	0	-6.727816	3.967324	0.216070
42	1	0	-4.145174	-1.393573	0.446729
43	1	0	-4.454965	-0.048074	-2.278962
44	1	0	-3.741521	0.755864	-0.893490
45	8	0	-6.042387	1.971273	2.361196
46	1	0	-6.448619	4.481870	1.139509
47	1	0	-6.587663	4.659425	-0.619821
48	1	0	-7.791313	3.712480	0.274191
49	1	0	1.327524	-0.402789	-1.415084
50	6	0	2.942639	0.374517	-0.259310
51	6	0	3.497783	0.851904	-1.615215

52	6	0	3.169914	1.485241	0.805520
53	1	0	4.588467	0.943428	-1.605380
54	1	0	3.232580	0.170600	-2.428023
55	1	0	3.082088	1.833934	-1.862798
56	6	0	4.624670	1.949739	1.010442
57	6	0	5.068194	3.085251	0.110256
58	8	0	4.349730	3.966838	-0.327918
59	8	0	6.398743	3.046097	-0.114458
60	6	0	6.954290	4.128222	-0.893193
61	6	0	4.789114	-1.583468	-0.428868
62	1	0	3.825619	-0.851858	1.299992
63	6	0	5.985950	-1.512275	0.175327
64	6	0	4.651193	-2.304881	-1.753310
65	1	0	6.881228	-1.918151	-0.288474
66	1	0	6.110712	-1.054330	1.153031
67	1	0	5.634048	-2.510259	-2.185956
68	1	0	4.142152	-3.267910	-1.623066
69	1	0	4.068868	-1.738397	-2.485087
70	1	0	5.345041	1.133643	0.926698
71	1	0	4.731032	2.344494	2.030312
72	1	0	2.779570	1.126933	1.759946
73	1	0	2.575681	2.363818	0.534163
74	8	0	2.354960	-4.345665	0.982891
75	1	0	3.211616	-2.929341	2.127521
76	6	0	0.755828	-1.428811	2.965781
77	1	0	1.566253	-2.116182	3.238723
78	1	0	0.563547	-0.769908	3.815949
79	1	0	-0.145552	-2.010838	2.751039
80	6	0	-4.885818	-2.522668	-1.202083
81	1	0	-5.924962	-2.383757	-0.888514
82	1	0	-4.874032	-2.544366	-2.297735
83	1	0	-4.580226	-3.508773	-0.843611
84	1	0	8.022995	3.925536	-0.947748
85	1	0	6.514518	4.140622	-1.892802
86	1	0	6.768886	5.085203	-0.400541

35

Center Number	Atomic Number	Atomic Type	Coordinates (Angstroms) X	Y	Z
1	6	0	-0.790087	0.037268	-1.226766
2	6	0	0.589318	0.733683	-1.220411
3	1	0	-0.826744	-0.579015	-2.129094
4	1	0	-1.571715	0.797330	-1.336235
5	1	0	0.638274	1.511440	-0.453188
6	1	0	0.730573	1.228250	-2.188714
7	6	0	3.909800	-0.928192	-0.166557
8	6	0	2.818115	-1.895340	0.362977
9	6	0	1.541194	-0.979560	0.392798
10	6	0	1.671079	-0.323689	-0.999377
11	6	0	3.256304	-2.580701	1.637707
12	1	0	2.596333	-2.690582	-0.356225
13	8	0	1.675770	0.080303	1.363543
14	6	0	-1.620690	-3.098420	0.851817
15	6	0	-2.139883	-2.037062	-0.172912
16	6	0	-1.079268	-0.835207	0.033097
17	6	0	0.176099	-1.623966	0.483339
18	6	0	-0.143145	-2.838253	0.943507
19	1	0	-2.091054	-2.969641	1.838809
20	1	0	-1.849189	-4.122771	0.535538
21	6	0	-2.005149	-2.663732	-1.580027
22	6	0	-1.508400	0.110379	1.185648
23	1	0	0.561574	-3.555997	1.350178
24	1	0	-2.353022	-2.002416	-2.377853
25	1	0	-0.963698	-2.923263	-1.793625
26	1	0	-2.588950	-3.587046	-1.641524
27	1	0	-0.722474	0.841845	1.386097
28	1	0	-2.419608	0.660251	0.935075
29	1	0	-1.691647	-0.440395	2.113662
30	6	0	-3.636184	-1.674878	0.122883
31	6	0	-4.232641	-0.663833	-0.885916
32	6	0	-5.527553	0.011233	-0.453717
33	6	0	-6.134041	0.974926	-1.487954
34	6	0	-6.794633	2.066135	-0.628813
35	6	0	-5.986713	2.003857	0.664314
36	8	0	-5.271092	0.856772	0.720448
37	1	0	-6.270332	-0.726343	-0.132969
38	1	0	-6.837258	0.463887	-2.150978
39	1	0	-5.336657	1.403681	-2.104820
40	1	0	-6.668123	3.064341	-1.057923
41	6	0	-8.290858	1.837115	-0.339881
42	1	0	-3.658589	-1.204957	1.111863
43	1	0	-4.444620	-1.170551	-1.836861
44	1	0	-3.515254	0.129871	-1.112700
45	8	0	-5.955063	2.820405	1.562566
46	1	0	-8.668470	2.586961	0.360908
47	1	0	-8.862949	1.914332	-1.269287
48	1	0	-8.472364	0.845911	0.089460
49	1	0	1.419288	-1.141364	-1.688685
50	6	0	3.191329	-0.030326	-1.267246
51	6	0	3.506178	-0.518570	-2.699388

52	6	0	3.588648	1.471596	-1.227423
53	1	0	4.550852	-0.344466	-2.975079
54	1	0	3.307427	-1.588591	-2.812528
55	1	0	2.878875	0.017859	-3.421298
56	6	0	3.553391	2.215752	0.111483
57	6	0	3.740811	3.708973	-0.044575
58	8	0	3.670882	4.338914	-1.085875
59	8	0	3.982278	4.294951	1.148850
60	6	0	4.131113	5.731143	1.144582
61	6	0	5.224027	-1.582517	-0.564564
62	1	0	4.143980	-0.265640	0.675847
63	6	0	5.351044	-2.888131	-0.847650
64	6	0	6.430581	-0.670671	-0.587486
65	1	0	6.317439	-3.308514	-1.113029
66	1	0	4.516611	-3.581532	-0.830012
67	1	0	7.339914	-1.217956	-0.849991
68	1	0	6.308139	0.145682	-1.308617
69	1	0	6.582868	-0.201627	0.393127
70	1	0	4.323779	1.861388	0.803803
71	1	0	2.604299	2.057791	0.633778
72	1	0	2.939729	2.002249	-1.931766
73	1	0	4.598449	1.569744	-1.642709
74	8	0	2.901667	-3.695626	1.983307
75	1	0	3.976682	-2.018128	2.265935
76	6	0	1.542188	-0.246160	2.743663
77	1	0	0.702578	-0.924072	2.926759
78	1	0	2.459319	-0.695093	3.145705
79	1	0	1.363434	0.695936	3.267557
80	6	0	-4.539435	-2.924135	0.212921
81	1	0	-5.551226	-2.650360	0.528203
82	1	0	-4.627299	-3.437891	-0.750843
83	1	0	-4.167724	-3.644913	0.945629
84	1	0	4.975954	6.023920	0.517379
85	1	0	3.219770	6.206504	0.775210
86	1	0	4.312058	6.005918	2.182878

36

Center Number	Atomic Number	Atomic Type	Coordinates (Angstroms) X	Y	Z
1	6	0	-1.002686	0.152722	-1.151326
2	6	0	0.394477	0.804347	-1.078844
3	1	0	-1.028099	-0.446290	-2.065899
4	1	0	-1.757410	0.937942	-1.270907
5	1	0	0.446213	1.546465	-0.277195
6	1	0	0.580639	1.335888	-2.019895
7	6	0	3.639686	-1.169203	-0.199172
8	6	0	2.477757	-1.972232	0.447554
9	6	0	1.254822	-0.995265	0.492580
10	6	0	1.443396	-0.287149	-0.867739
11	6	0	2.876450	-2.680526	1.721066
12	1	0	2.153720	-2.786536	-0.214849
13	8	0	1.417837	0.018860	1.508689
14	6	0	-2.016718	-2.981977	0.839520
15	6	0	-2.465940	-1.876944	-0.171206
16	6	0	-1.356198	-0.730923	0.081852
17	6	0	-0.142799	-1.582166	0.535025
18	6	0	-0.531127	-2.789153	0.960615
19	1	0	-2.498109	-2.850177	1.820598
20	1	0	-2.285202	-3.988285	0.498119
21	6	0	-2.333020	-2.479075	-1.589120
22	6	0	-1.760491	0.206428	1.250084
23	1	0	0.118773	-3.557535	1.367666
24	1	0	-2.635246	-1.785272	-2.377812
25	1	0	-1.300938	-2.781988	-1.790767
26	1	0	-2.957883	-3.372483	-1.681772
27	1	0	-0.939205	0.884897	1.489760
28	1	0	-2.632321	0.814468	0.993223
29	1	0	-1.998788	-0.357025	2.157767
30	6	0	-3.949251	-1.452282	0.105276
31	6	0	-4.479072	-0.392805	-0.890642
32	6	0	-5.759279	0.319371	-0.474431
33	6	0	-6.295603	1.338621	-1.494233
34	6	0	-6.939733	2.427458	-0.619740
35	6	0	-6.176338	2.291500	0.694761
36	8	0	-5.506712	1.115862	0.733707
37	1	0	-6.539751	-0.398099	-0.200955
38	1	0	-6.997078	0.876807	-2.194276
39	1	0	-5.463336	1.754472	-2.072713
40	1	0	-6.760781	3.432937	-1.011449
41	6	0	-8.452077	2.248914	-0.384504
42	1	0	-3.968442	-1.003442	1.103978
43	1	0	-4.684278	-0.864998	-1.860654
44	1	0	-3.726852	0.378859	-1.076626
45	8	0	-6.142735	3.075756	1.621031
46	1	0	-8.823373	2.992060	0.326718
47	1	0	-8.990625	2.375381	-1.328458
48	1	0	-8.684511	1.253260	0.008502
49	1	0	1.223032	-1.071391	-1.607652
50	6	0	2.965324	0.015516	-1.039797
51	6	0	3.305921	-0.007574	-2.542715

52	6	0	3.323977	1.408844	-0.449934
53	1	0	4.375695	0.115934	-2.733166
54	1	0	3.004255	-0.948752	-3.009527
55	1	0	2.783071	0.808279	-3.055479
56	6	0	4.798811	1.817399	-0.534125
57	6	0	5.054937	3.215738	-0.011045
58	8	0	4.210660	4.066806	0.205096
59	8	0	6.375918	3.430615	0.173714
60	6	0	6.757463	4.746592	0.629740
61	6	0	4.647995	-2.052923	-0.934101
62	1	0	4.185167	-0.706390	0.629031
63	6	0	4.308333	-3.127788	-1.660961
64	6	0	6.098983	-1.653931	-0.799478
65	1	0	5.064793	-3.721224	-2.167510
66	1	0	3.281130	-3.456412	-1.790758
67	1	0	6.759068	-2.362107	-1.307901
68	1	0	6.283112	-0.659617	-1.223298
69	1	0	6.389346	-1.605601	0.257337
70	1	0	5.165805	1.809412	-1.568424
71	1	0	5.450027	1.135715	0.021334
72	1	0	3.007025	1.442421	0.594279
73	1	0	2.733621	2.166603	-0.975842
74	8	0	3.991070	-2.648631	2.214728
75	1	0	2.090297	-3.298990	2.196659
76	6	0	1.242892	-0.353307	2.872656
77	1	0	0.341345	-0.956654	3.022478
78	1	0	2.111317	-0.896528	3.262987
79	1	0	1.143030	0.579970	3.432022
80	6	0	-4.910250	-2.660445	0.150067
81	1	0	-5.911727	-2.349164	0.462918
82	1	0	-5.010248	-3.142195	-0.828922
83	1	0	-4.580579	-3.418164	0.865389
84	1	0	7.842970	4.719594	0.714444
85	1	0	6.447272	5.503978	-0.093550
86	1	0	6.301909	4.959152	1.599218

37

Center Number	Atomic Number	Atomic Type	Coordinates (Angstroms) X	Y	Z
1	6	0	-1.094100	0.178121	-1.129191
2	6	0	0.299561	0.835866	-1.029719
3	1	0	-1.118530	-0.377072	-2.070876
4	1	0	-1.853996	0.963579	-1.210417
5	1	0	0.348517	1.539399	-0.193533
6	1	0	0.479439	1.413177	-1.944874
7	6	0	3.554849	-1.112721	-0.170938
8	6	0	2.390164	-2.022287	0.332407
9	6	0	1.173257	-1.037061	0.444362
10	6	0	1.357732	-0.256792	-0.876794
11	6	0	2.807987	-2.803491	1.554954
12	1	0	2.107177	-2.760519	-0.423339
13	8	0	1.353979	-0.080960	1.511106
14	6	0	-2.072931	-3.059393	0.709989
15	6	0	-2.539752	-1.908286	-0.241454
16	6	0	-1.438024	-0.766203	0.060945
17	6	0	-0.218580	-1.630026	0.465732
18	6	0	-0.588111	-2.861198	0.834477
19	1	0	-2.550774	-2.982379	1.698798
20	1	0	-2.334351	-4.049798	0.319936
21	6	0	-2.413077	-2.438259	-1.688172
22	6	0	-1.843418	0.111556	1.274052
23	1	0	0.083341	-3.633692	1.195212
24	1	0	-2.720664	-1.707100	-2.440321
25	1	0	-1.381925	-2.730409	-1.909601
26	1	0	-3.037845	-3.326616	-1.820728
27	1	0	-1.025577	0.782926	1.543549
28	1	0	-2.718247	0.728323	1.050044
29	1	0	-2.077221	-0.494648	2.154681
30	6	0	-4.024463	-1.510816	0.067042
31	6	0	-4.570184	-0.402095	-0.864388
32	6	0	-5.879955	0.248503	-0.414853
33	6	0	-6.345344	1.419746	-1.304116
34	6	0	-5.794025	2.666023	-0.594383
35	6	0	-5.672841	2.200332	0.852735
36	8	0	-5.715632	0.848813	0.915928
37	1	0	-6.665164	-0.499550	-0.292923
38	1	0	-7.439773	1.461005	-1.329697
39	1	0	-5.988226	1.317983	-2.331880
40	1	0	-4.763702	2.859994	-0.925338
41	6	0	-6.609720	3.950244	-0.751196
42	1	0	-4.040965	-1.118442	1.088876
43	1	0	-4.758351	-0.817204	-1.863131
44	1	0	-3.826615	0.389514	-0.998021
45	8	0	-5.540391	2.880733	1.849142
46	1	0	-6.177559	4.758008	-0.154453
47	1	0	-6.624472	4.265351	-1.798972
48	1	0	-7.644145	3.799305	-0.424808
49	1	0	1.140565	-1.000625	-1.657066
50	6	0	2.878554	0.061514	-1.025156
51	6	0	3.259810	0.067475	-2.519772

52	6	0	3.223206	1.449802	-0.415711
53	1	0	4.343191	0.072648	-2.668846
54	1	0	2.858066	-0.800197	-3.049863
55	1	0	2.851758	0.961247	-3.006082
56	6	0	4.694355	1.872864	-0.516378
57	6	0	4.948927	3.249383	0.060356
58	8	0	4.114341	4.122833	0.219055
59	8	0	6.254074	3.419507	0.364086
60	6	0	6.634574	4.718043	0.869216
61	6	0	4.691994	-1.911361	-0.800841
62	1	0	3.974729	-0.631673	0.719501
63	6	0	5.963272	-1.697252	-0.427012
64	6	0	4.367318	-3.004436	-1.797634
65	1	0	6.785060	-2.262086	-0.859543
66	1	0	6.225671	-0.959006	0.326122
67	1	0	5.274610	-3.357441	-2.295171
68	1	0	3.909806	-3.867143	-1.297507
69	1	0	3.662250	-2.676565	-2.566403
70	1	0	5.027573	1.917032	-1.561313
71	1	0	5.366033	1.168682	-0.019079
72	1	0	2.917542	1.461888	0.633063
73	1	0	2.618849	2.209091	-0.922877
74	8	0	2.497221	-3.962932	1.774873
75	1	0	3.478717	-2.274275	2.261502
76	6	0	1.137540	-0.506537	2.853521
77	1	0	1.016239	0.402832	3.447077
78	1	0	0.238113	-1.121947	2.952124
79	1	0	1.996160	-1.063905	3.248619
80	6	0	-4.977167	-2.726464	0.052104
81	1	0	-5.972563	-2.444813	0.409183
82	1	0	-5.096713	-3.142745	-0.954426
83	1	0	-4.625300	-3.527627	0.706780
84	1	0	7.707784	4.656727	1.044321
85	1	0	6.409055	5.492802	0.133165
86	1	0	6.105238	4.933239	1.799919

38

Center Number	Atomic Number	Atomic Type	Coordinates (Angstroms) X	Y	Z
1	6	0	-0.962705	0.069946	-1.205386
2	6	0	0.424646	0.746052	-1.198119
3	1	0	-0.991068	-0.586760	-2.078919
4	1	0	-1.731815	0.833946	-1.364324
5	1	0	0.474235	1.541428	-0.449349
6	1	0	0.589315	1.213709	-2.176212
7	6	0	3.714922	-1.103520	-0.216479
8	6	0	2.574706	-1.870883	0.507234
9	6	0	1.332378	-0.920486	0.485857
10	6	0	1.493867	-0.312220	-0.924928
11	6	0	2.996765	-2.463336	1.831499
12	1	0	2.263016	-2.743837	-0.082423
13	8	0	1.477259	0.172920	1.420265
14	6	0	-1.889481	-2.955182	0.985744
15	6	0	-2.374796	-1.921160	-0.081679
16	6	0	-1.286352	-0.738726	0.086763
17	6	0	-0.051428	-1.533049	0.581676
18	6	0	-0.408089	-2.721801	1.080432
19	1	0	-2.364509	-2.779690	1.962936
20	1	0	-2.136667	-3.985449	0.705020
21	6	0	-2.247327	-2.603500	-1.463403
22	6	0	-1.701361	0.263737	1.195950
23	1	0	0.262407	-3.451445	1.524280
24	1	0	-2.573478	-1.963029	-2.286906
25	1	0	-1.211724	-2.897173	-1.661329
26	1	0	-2.854046	-3.513502	-1.495468
27	1	0	-0.897025	0.979468	1.378363
28	1	0	-2.593494	0.828678	0.912338
29	1	0	-1.911731	-0.244445	2.142392
30	6	0	-3.863271	-1.512063	0.190701
31	6	0	-4.429374	-0.522564	-0.855950
32	6	0	-5.719460	0.183566	-0.460339
33	6	0	-6.288228	1.142355	-1.520330
34	6	0	-6.947742	2.255961	-0.689505
35	6	0	-6.165489	2.199744	0.619568
36	8	0	-5.470056	1.041800	0.705710
37	1	0	-6.481962	-0.535590	-0.144033
38	1	0	-6.986493	0.633948	-2.190567
39	1	0	-5.471374	1.549273	-2.126483
40	1	0	-6.797860	3.245820	-1.130185
41	6	0	-8.452312	2.053165	-0.425841
42	1	0	-3.878637	-1.006124	1.161797
43	1	0	-4.636496	-1.052920	-1.794924
44	1	0	-3.696178	0.252959	-1.094398
45	8	0	-6.137325	3.028295	1.506670
46	1	0	-8.831817	2.818152	0.257320
47	1	0	-9.005600	2.125580	-1.366912
48	1	0	-8.656251	1.070753	0.013457
49	1	0	1.277782	-1.150516	-1.603979
50	6	0	3.009182	-0.000690	-1.137782
51	6	0	3.335891	-0.131283	-2.637638

52	6	0	3.348826	1.438589	-0.656400
53	1	0	4.401637	-0.006550	-2.847452
54	1	0	3.044466	-1.110058	-3.027188
55	1	0	2.796277	0.635561	-3.205583
56	6	0	4.827521	1.869263	-0.796098
57	6	0	4.996151	3.303885	-0.352453
58	8	0	4.842925	4.279574	-1.068890
59	8	0	5.307342	3.396491	0.956771
60	6	0	5.433547	4.729488	1.498305
61	6	0	4.728349	-2.024800	-0.896315
62	1	0	4.265140	-0.566748	0.562611
63	6	0	4.401807	-3.172132	-1.509259
64	6	0	6.169649	-1.574072	-0.846152
65	1	0	5.162060	-3.788323	-1.981703
66	1	0	3.382404	-3.541076	-1.576627
67	1	0	6.836246	-2.310646	-1.303102
68	1	0	6.311343	-0.622199	-1.371625
69	1	0	6.488501	-1.413778	0.191218
70	1	0	5.153338	1.817690	-1.837614
71	1	0	5.475434	1.232133	-0.189657
72	1	0	3.048714	1.538676	0.388962
73	1	0	2.740550	2.143779	-1.234643
74	8	0	4.119138	-2.385298	2.301341
75	1	0	2.220051	-3.037862	2.373117
76	6	0	1.336671	-0.101428	2.811512
77	1	0	0.481597	-0.754040	3.017613
78	1	0	2.245075	-0.548022	3.232013
79	1	0	1.171993	0.862790	3.298590
80	6	0	-4.796975	-2.735476	0.319635
81	1	0	-5.800792	-2.427539	0.628357
82	1	0	-4.899613	-3.275789	-0.628060
83	1	0	-4.441681	-3.443107	1.073059
84	1	0	5.672530	4.591836	2.551865
85	1	0	6.234592	5.270315	0.989872
86	1	0	4.494413	5.275176	1.384623

39

Center Number	Atomic Number	Atomic Type	Coordinates (Angstroms) X	Y	Z
1	6	0	-1.022269	0.154088	-1.108603
2	6	0	0.360779	0.825176	-0.967448
3	1	0	-1.030654	-0.361877	-2.072503
4	1	0	-1.791847	0.932236	-1.164147
5	1	0	0.390015	1.490568	-0.099801
6	1	0	0.539940	1.445321	-1.854405
7	6	0	3.635613	-1.126058	-0.178341
8	6	0	2.477338	-2.060660	0.280856
9	6	0	1.247414	-1.099707	0.431213
10	6	0	1.433280	-0.258767	-0.851196
11	6	0	2.899823	-2.905414	1.461293
12	1	0	2.194679	-2.761886	-0.507975
13	8	0	1.406055	-0.190984	1.541655
14	6	0	-1.977585	-3.169786	0.578921
15	6	0	-2.451633	-1.981666	-0.321699
16	6	0	-1.363739	-0.842799	0.037991
17	6	0	-0.137907	-1.710078	0.413980
18	6	0	-0.496170	-2.960644	0.723886
19	1	0	-2.462991	-3.145200	1.566662
20	1	0	-2.224576	-4.144023	0.141383
21	6	0	-2.311471	-2.444700	-1.790187
22	6	0	-1.787957	-0.021898	1.283943
23	1	0	0.182514	-3.739743	1.055255
24	1	0	-2.618338	-1.681985	-2.510590
25	1	0	-1.276943	-2.720419	-2.017037
26	1	0	-2.929275	-3.330075	-1.967266
27	1	0	-0.976569	0.640189	1.592680
28	1	0	-2.664138	0.599332	1.077441
29	1	0	-2.027569	-0.668117	2.134108
30	6	0	-3.942590	-1.613271	-0.006029
31	6	0	-4.495823	-0.476781	-0.899392
32	6	0	-5.801061	0.161754	-0.420632
33	6	0	-6.282759	1.344694	-1.285292
34	6	0	-5.719495	2.580987	-0.567720
35	6	0	-5.577010	2.095625	0.871021
36	8	0	-5.618870	0.743352	0.916300
37	1	0	-6.581953	-0.590656	-0.297704
38	1	0	-7.377482	1.385640	-1.291084
39	1	0	-5.943631	1.257571	-2.320449
40	1	0	-4.693887	2.777837	-0.911598
41	6	0	-6.535315	3.868286	-0.695441
42	1	0	-3.969639	-1.260226	1.029824
43	1	0	-4.692211	-0.863160	-1.908038
44	1	0	-3.753111	0.318310	-1.016219
45	8	0	-5.429379	2.762590	1.874377
46	1	0	-6.093710	4.667282	-0.093780
47	1	0	-6.564312	4.197853	-1.738473
48	1	0	-7.565305	3.714282	-0.356766
49	1	0	1.238847	-0.967941	-1.668716
50	6	0	2.952303	0.092571	-0.964739
51	6	0	3.335175	0.155681	-2.455332

52	6	0	3.237620	1.460350	-0.282953
53	1	0	4.396958	0.364889	-2.612093
54	1	0	3.112772	-0.786684	-2.963710
55	1	0	2.766464	0.950169	-2.952424
56	6	0	4.710597	1.926467	-0.249763
57	6	0	4.819153	3.259978	0.453245
58	8	0	4.938073	3.407906	1.657991
59	8	0	4.737285	4.294632	-0.409260
60	6	0	4.778867	5.619279	0.165796
61	6	0	4.772809	-1.868163	-0.880957
62	1	0	4.066799	-0.697778	0.736343
63	6	0	4.603161	-3.003952	-1.575360
64	6	0	6.157915	-1.276782	-0.739252
65	1	0	5.448327	-3.494819	-2.050325
66	1	0	3.640088	-3.486543	-1.704150
67	1	0	6.913050	-1.928513	-1.186786
68	1	0	6.234739	-0.296867	-1.223142
69	1	0	6.415873	-1.128817	0.317197
70	1	0	5.117788	2.023507	-1.259032
71	1	0	5.324024	1.221770	0.315148
72	1	0	2.861817	1.422288	0.741746
73	1	0	2.656310	2.225673	-0.810593
74	8	0	2.531529	-4.050878	1.661500
75	1	0	3.626367	-2.435508	2.155879
76	6	0	1.215069	-0.693614	2.860646
77	1	0	0.312027	-1.306793	2.942047
78	1	0	2.077087	-1.280153	3.202973
79	1	0	1.116002	0.180214	3.508902
80	6	0	-4.880924	-2.838072	-0.074531
81	1	0	-5.886558	-2.577517	0.269852
82	1	0	-4.975452	-3.226130	-1.094821
83	1	0	-4.532953	-3.653748	0.564148
84	1	0	4.701102	6.303184	-0.678190
85	1	0	3.942604	5.760263	0.853897
86	1	0	5.720114	5.771112	0.698289

40

Center Number	Atomic Number	Atomic Type	Coordinates (Angstroms) X	Y	Z
1	6	0	-0.812630	0.147664	-1.064948
2	6	0	0.550428	0.834714	-0.828155
3	1	0	-0.774809	-0.294389	-2.064415
4	1	0	-1.597094	0.912398	-1.092279
5	1	0	0.537548	1.442207	0.082111
6	1	0	0.735517	1.511525	-1.669801
7	6	0	3.823837	-1.076150	0.031229
8	6	0	2.684351	-2.098180	0.296492
9	6	0	1.424492	-1.171697	0.454379
10	6	0	1.648514	-0.228587	-0.750075
11	6	0	3.046853	-3.032029	1.426289
12	1	0	2.487861	-2.728015	-0.576624
13	8	0	1.513569	-0.338205	1.630644
14	6	0	-1.748347	-3.317858	0.314057
15	6	0	-2.223570	-2.067967	-0.498429
16	6	0	-1.173524	-0.940731	-0.012338
17	6	0	0.058264	-1.809009	0.335686
18	6	0	-0.278337	-3.089100	0.526747
19	1	0	-2.265165	-3.387991	1.283319
20	1	0	-1.956096	-4.256287	-0.212853
21	6	0	-2.028767	-2.403600	-1.995173
22	6	0	-1.658409	-0.228925	1.278047
23	1	0	0.407126	-3.879565	0.814133
24	1	0	-2.333290	-1.590307	-2.658968
25	1	0	-0.981126	-2.634836	-2.211589
26	1	0	-2.617052	-3.285631	-2.265589
27	1	0	-0.878648	0.433066	1.659741
28	1	0	-2.547907	0.379208	1.091419
29	1	0	-1.901608	-0.944245	2.069759
30	6	0	-3.731809	-1.761663	-0.197702
31	6	0	-4.283906	-0.552426	-0.989735
32	6	0	-5.659029	-0.048230	-0.547863
33	6	0	-6.131977	1.227221	-1.274602
34	6	0	-5.705700	2.367405	-0.337050
35	6	0	-5.657055	1.680148	1.023719
36	8	0	-5.625718	0.335943	0.869860
37	1	0	-6.403898	-0.843250	-0.608538
38	1	0	-7.222670	1.219246	-1.376750
39	1	0	-5.700967	1.309874	-2.275408
40	1	0	-4.668276	2.660802	-0.552819
41	6	0	-6.583667	3.619694	-0.352327
42	1	0	-3.797648	-1.512370	0.866490
43	1	0	-4.379731	-0.817259	-2.050740
44	1	0	-3.583343	0.287311	-0.944371
45	8	0	-5.632484	2.196598	2.121977
46	1	0	-6.244450	4.339152	0.397792
47	1	0	-6.542526	4.099997	-1.334618
48	1	0	-7.627852	3.368534	-0.137999
49	1	0	1.497122	-0.875911	-1.626347
50	6	0	3.168538	0.138791	-0.781907
51	6	0	3.634979	0.220774	-2.247507

52	6	0	3.577929	1.436869	-0.026043
53	1	0	4.703935	0.446699	-2.320370
54	1	0	3.448797	-0.713514	-2.783377
55	1	0	3.088014	1.004651	-2.781943
56	6	0	3.284906	2.782871	-0.701089
57	6	0	3.796990	3.959234	0.104589
58	8	0	4.561813	3.903050	1.051097
59	8	0	3.308437	5.124153	-0.374650
60	6	0	3.756785	6.330122	0.281363
61	6	0	5.109607	-1.679365	-0.519958
62	1	0	4.080877	-0.659197	1.010511
63	6	0	6.297810	-1.251377	-0.063478
64	6	0	5.037712	-2.792413	-1.543164
65	1	0	7.232075	-1.653315	-0.447184
66	1	0	6.373681	-0.488359	0.706815
67	1	0	6.034157	-3.033959	-1.922962
68	1	0	4.621713	-3.706528	-1.101574
69	1	0	4.401168	-2.541362	-2.397289
70	1	0	2.219360	2.945744	-0.879228
71	1	0	3.768299	2.854811	-1.683231
72	1	0	4.660722	1.386884	0.137863
73	1	0	3.115202	1.423080	0.964220
74	8	0	2.779806	-4.222718	1.451784
75	1	0	3.635449	-2.588276	2.253943
76	6	0	1.273563	-0.928523	2.905584
77	1	0	1.013928	-0.110435	3.581956
78	1	0	0.447933	-1.646633	2.879038
79	1	0	2.169825	-1.426110	3.296447
80	6	0	-4.637459	-2.993632	-0.416048
81	1	0	-5.647639	-2.804964	-0.040478
82	1	0	-4.726001	-3.255686	-1.476393
83	1	0	-4.265064	-3.871928	0.116783
84	1	0	4.841696	6.426076	0.199337
85	1	0	3.259647	7.145657	-0.242081
86	1	0	3.469605	6.318556	1.334902

41

Center Number	Atomic Number	Atomic Type	Coordinates (Angstroms) X	Y	Z
1	6	0	1.128249	-0.292116	-1.220647
2	6	0	-0.220951	-1.041688	-1.196950
3	1	0	1.134610	0.325612	-2.123044
4	1	0	1.938467	-1.021108	-1.334737
5	1	0	-0.239629	-1.794621	-0.404061
6	1	0	-0.340038	-1.573260	-2.149199
7	6	0	-3.614687	0.660598	-0.324808
8	6	0	-2.516914	1.591224	0.270718
9	6	0	-1.246166	0.677125	0.363591
10	6	0	-1.352922	-0.030564	-1.004524
11	6	0	-3.017087	2.287391	1.515595
12	1	0	-2.242331	2.384925	-0.428561
13	8	0	-1.386193	-0.351772	1.364641
14	6	0	1.865145	2.867595	0.847611
15	6	0	2.425280	1.809622	-0.158518
16	6	0	1.385440	0.588871	0.037539
17	6	0	0.106214	1.351477	0.459428
18	6	0	0.391809	2.576797	0.913235
19	1	0	2.318623	2.755641	1.844502
20	1	0	2.078708	3.894211	0.527914
21	6	0	2.305156	2.422876	-1.572757
22	6	0	1.813654	-0.341972	1.202269
23	1	0	-0.334462	3.284049	1.300274
24	1	0	2.671264	1.758106	-2.359455
25	1	0	1.264328	2.669602	-1.803679
26	1	0	2.880361	3.351728	-1.633447
27	1	0	1.032902	-1.078499	1.403950
28	1	0	2.733092	-0.884432	0.965330
29	1	0	1.982169	0.219725	2.126512
30	6	0	3.922923	1.478378	0.166630
31	6	0	4.561456	0.486861	-0.836132
32	6	0	5.851000	-0.178659	-0.374225
33	6	0	6.511086	-1.104121	-1.410381
34	6	0	7.160424	-2.205865	-0.556311
35	6	0	6.303502	-2.193193	0.706367
36	8	0	5.566231	-1.060133	0.766501
37	1	0	6.568868	0.561639	-0.006276
38	1	0	7.227624	-0.563837	-2.034904
39	1	0	5.743659	-1.530482	-2.065824
40	1	0	7.069637	-3.194229	-1.016048
41	6	0	8.640123	-1.956969	-0.205019
42	1	0	3.936211	1.003629	1.153420
43	1	0	4.795278	1.009182	-1.773447
44	1	0	3.861119	-0.312331	-1.095009
45	8	0	6.252032	-3.035381	1.579650
46	1	0	9.006156	-2.718932	0.488827
47	1	0	9.248016	-1.997463	-1.113831
48	1	0	8.785964	-0.975013	0.257834
49	1	0	-1.172244	0.770431	-1.735943
50	6	0	-2.849828	-0.440753	-1.206766
51	6	0	-3.173563	-0.358233	-2.710833

52	6	0	-3.080273	-1.894562	-0.697534
53	1	0	-4.213513	-0.609831	-2.936937
54	1	0	-2.992825	0.646348	-3.102755
55	1	0	-2.538753	-1.058291	-3.266816
56	6	0	-4.477205	-2.525989	-0.840853
57	6	0	-5.397702	-2.373142	0.353815
58	8	0	-5.078296	-1.993911	1.466421
59	8	0	-6.651985	-2.767915	0.046757
60	6	0	-7.618091	-2.748065	1.120168
61	6	0	-4.755812	1.423565	-0.996097
62	1	0	-4.054992	0.120746	0.520737
63	6	0	-4.613847	2.622551	-1.581850
64	6	0	-6.117132	0.768288	-0.944401
65	1	0	-5.464145	3.120978	-2.039775
66	1	0	-3.668189	3.151950	-1.633920
67	1	0	-6.421153	0.588810	0.094352
68	1	0	-6.878416	1.391428	-1.421346
69	1	0	-6.120754	-0.205097	-1.446688
70	1	0	-4.363966	-3.611612	-0.965118
71	1	0	-5.013616	-2.200099	-1.736545
72	1	0	-2.776768	-1.956710	0.348983
73	1	0	-2.392256	-2.533857	-1.260922
74	8	0	-2.712320	3.422167	1.845123
75	1	0	-3.741169	1.716197	2.131553
76	6	0	-1.279693	0.018931	2.734809
77	1	0	-0.436198	0.693113	2.915486
78	1	0	-2.200560	0.489383	3.101780
79	1	0	-1.124780	-0.908007	3.292086
80	6	0	4.797855	2.745490	0.280918
81	1	0	5.812307	2.489176	0.602591
82	1	0	4.884341	3.272937	-0.675559
83	1	0	4.403733	3.448758	1.018945
84	1	0	-7.737819	-1.733343	1.505650
85	1	0	-8.548457	-3.101352	0.677717
86	1	0	-7.300808	-3.410833	1.928145

42

Center Number	Atomic Number	Atomic Type	Coordinates (Angstroms) X	Y	Z
1	6	0	-0.962403	0.535103	-0.821939
2	6	0	0.377817	1.122599	-0.332078
3	1	0	-0.850649	0.330634	-1.890150
4	1	0	-1.742969	1.298995	-0.731279
5	1	0	0.294207	1.497556	0.692184
6	1	0	0.636772	1.978912	-0.966841
7	6	0	3.600959	-0.998091	0.189879
8	6	0	2.423673	-2.017820	0.224511
9	6	0	1.159426	-1.140035	0.518578
10	6	0	1.459524	0.045381	-0.425588
11	6	0	2.742678	-3.180522	1.137008
12	1	0	2.244261	-2.450099	-0.762882
13	8	0	1.170276	-0.610666	1.862540
14	6	0	-2.005197	-3.135281	-0.326907
15	6	0	-2.411403	-1.727281	-0.874795
16	6	0	-1.400023	-0.757573	-0.071130
17	6	0	-0.198906	-1.707118	0.163623
18	6	0	-0.554885	-2.990190	0.039442
19	1	0	-2.595162	-3.408858	0.561362
20	1	0	-2.175489	-3.929760	-1.062751
21	6	0	-2.100930	-1.726353	-2.389132
22	6	0	-1.979162	-0.347694	1.308569
23	1	0	0.104061	-3.838447	0.193460
24	1	0	-2.354779	-0.781862	-2.877316
25	1	0	-1.039481	-1.921183	-2.570541
26	1	0	-2.666265	-2.517030	-2.891298
27	1	0	-1.226256	0.187875	1.890328
28	1	0	-2.846377	0.309710	1.200801
29	1	0	-2.289590	-1.218832	1.893609
30	6	0	-3.937811	-1.464098	-0.628151
31	6	0	-4.421624	-0.095838	-1.164602
32	6	0	-5.818062	0.333064	-0.709943
33	6	0	-6.224199	1.750959	-1.161599
34	6	0	-5.857462	2.641146	0.035949
35	6	0	-5.911760	1.665293	1.206951
36	8	0	-5.879041	0.389531	0.756542
37	1	0	-6.565872	-0.408045	-0.996463
38	1	0	-7.304257	1.794579	-1.339182
39	1	0	-5.719079	2.044121	-2.085277
40	1	0	-4.805021	2.950828	-0.036512
41	6	0	-6.722224	3.885500	0.243056
42	1	0	-4.085845	-1.462369	0.456634
43	1	0	-4.449299	-0.116400	-2.261729
44	1	0	-3.716996	0.695447	-0.890686
45	8	0	-5.962385	1.921326	2.392468
46	1	0	-6.429265	4.413839	1.154370
47	1	0	-6.610924	4.570273	-0.603169
48	1	0	-7.780470	3.616893	0.328286
49	1	0	1.369963	-0.387545	-1.432337
50	6	0	2.973473	0.405008	-0.267809
51	6	0	3.505201	0.897446	-1.626392

52	6	0	3.148725	1.502293	0.820336
53	1	0	4.574866	1.121844	-1.606740
54	1	0	3.351300	0.146921	-2.406904
55	1	0	2.977834	1.810465	-1.925026
56	6	0	4.575890	1.961917	1.186904
57	6	0	5.291844	2.864070	0.204028
58	8	0	6.438344	2.705779	-0.183801
59	8	0	4.533458	3.913925	-0.169396
60	6	0	5.139520	4.860567	-1.075756
61	6	0	4.821870	-1.514593	-0.573519
62	1	0	3.924073	-0.863457	1.230918
63	6	0	4.745047	-2.375590	-1.600207
64	6	0	6.173439	-1.030654	-0.098155
65	1	0	5.644377	-2.717855	-2.105087
66	1	0	3.807868	-2.766877	-1.981960
67	1	0	6.982838	-1.538528	-0.629744
68	1	0	6.302218	0.046880	-0.248426
69	1	0	6.300271	-1.218479	0.976100
70	1	0	5.238648	1.121016	1.395208
71	1	0	4.503270	2.540557	2.117261
72	1	0	2.683347	1.136823	1.737932
73	1	0	2.580431	2.386279	0.514035
74	8	0	2.384081	-4.330706	0.946995
75	1	0	3.382280	-2.945909	2.013049
76	6	0	0.852086	-1.490780	2.936240
77	1	0	0.654256	-0.856481	3.803653
78	1	0	-0.034004	-2.097328	2.724307
79	1	0	1.689529	-2.156819	3.180180
80	6	0	-4.828121	-2.594749	-1.189252
81	1	0	-5.867981	-2.464102	-0.874372
82	1	0	-4.818106	-2.619139	-2.284843
83	1	0	-4.513404	-3.577226	-0.828707
84	1	0	5.413316	4.367806	-2.011181
85	1	0	4.378962	5.620209	-1.251033
86	1	0	6.028736	5.304160	-0.622745

43

Center Number	Atomic Number	Atomic Type	Coordinates (Angstroms) X	Y	Z
1	6	0	-1.025670	0.533836	-0.826773
2	6	0	0.320357	1.115285	-0.346416
3	1	0	-0.918525	0.318471	-1.893260
4	1	0	-1.800758	1.303672	-0.741513
5	1	0	0.242634	1.504036	0.673062
6	1	0	0.587158	1.960651	-0.991855
7	6	0	3.540018	-1.044958	0.101984
8	6	0	2.348469	-2.025361	0.296596
9	6	0	1.084295	-1.136876	0.544108
10	6	0	1.390972	0.026548	-0.424813
11	6	0	2.641843	-3.139785	1.273115
12	1	0	2.132390	-2.555561	-0.640966
13	8	0	1.075981	-0.572077	1.874800
14	6	0	-2.098084	-3.123308	-0.291354
15	6	0	-2.487363	-1.719663	-0.861083
16	6	0	-1.472720	-0.748361	-0.063139
17	6	0	-0.277733	-1.702506	0.190345
18	6	0	-0.649009	-2.982539	0.080653
19	1	0	-2.695628	-3.379318	0.596900
20	1	0	-2.269254	-3.926450	-1.017377
21	6	0	-2.166457	-1.742992	-2.373119
22	6	0	-2.055291	-0.320891	1.309752
23	1	0	-0.010119	-3.844495	0.247177
24	1	0	-2.411009	-0.804494	-2.876938
25	1	0	-1.104930	-1.946189	-2.544786
26	1	0	-2.733395	-2.537456	-2.867445
27	1	0	-1.302739	0.220089	1.886473
28	1	0	-2.920208	0.337327	1.190288
29	1	0	-2.370419	-1.183788	1.904533
30	6	0	-4.013205	-1.441373	-0.628462
31	6	0	-4.480536	-0.073127	-1.179029
32	6	0	-5.886858	0.359895	-0.760112
33	6	0	-6.269816	1.785917	-1.206110
34	6	0	-5.930587	2.659124	0.011944
35	6	0	-6.026156	1.670128	1.169017
36	8	0	-5.990733	0.399178	0.704349
37	1	0	-6.630801	-0.372281	-1.078015
38	1	0	-7.344190	1.839585	-1.413092
39	1	0	-5.737202	2.086380	-2.111832
40	1	0	-4.874224	2.961384	-0.026576
41	6	0	-6.791460	3.907856	0.208939
42	1	0	-4.169851	-1.429071	0.455023
43	1	0	-4.480898	-0.094643	-2.276473
44	1	0	-3.780928	0.716389	-0.888214
45	8	0	-6.109120	1.912030	2.355584
46	1	0	-6.521465	4.422633	1.134969
47	1	0	-6.649570	4.601957	-0.624988
48	1	0	-7.853854	3.646851	0.259447
49	1	0	1.294795	-0.427245	-1.422866
50	6	0	2.905955	0.374903	-0.286442
51	6	0	3.411051	0.938850	-1.627184

52	6	0	3.136302	1.415195	0.847198
53	1	0	4.490583	1.117061	-1.623505
54	1	0	3.205544	0.252947	-2.452979
55	1	0	2.918632	1.892827	-1.843417
56	6	0	4.583425	1.885170	1.087134
57	6	0	5.052809	3.006150	0.183373
58	8	0	4.372750	3.950897	-0.177898
59	8	0	6.355962	2.873349	-0.145126
60	6	0	6.932013	3.931266	-0.942438
61	6	0	4.623353	-1.599130	-0.824631
62	1	0	4.008680	-0.929622	1.084024
63	6	0	4.363949	-2.301409	-1.937631
64	6	0	6.051333	-1.315153	-0.420481
65	1	0	5.169771	-2.670688	-2.566295
66	1	0	3.356453	-2.528786	-2.274471
67	1	0	6.762860	-1.799309	-1.095234
68	1	0	6.264857	-0.239474	-0.426538
69	1	0	6.241733	-1.671724	0.599312
70	1	0	5.304758	1.066196	1.054045
71	1	0	4.645055	2.300300	2.102378
72	1	0	2.758381	0.990831	1.778396
73	1	0	2.530945	2.303944	0.640072
74	8	0	3.705967	-3.297814	1.847439
75	1	0	1.827903	-3.875335	1.425684
76	6	0	0.767755	-1.426828	2.972314
77	1	0	0.544994	-0.770295	3.816932
78	1	0	-0.105031	-2.057277	2.771646
79	1	0	1.617886	-2.062332	3.246862
80	6	0	-4.908714	-2.569235	-1.186981
81	1	0	-5.947144	-2.434452	-0.869575
82	1	0	-4.901229	-2.594430	-2.282588
83	1	0	-4.597202	-3.552790	-0.826505
84	1	0	6.411919	4.010999	-1.899408
85	1	0	6.868844	4.883579	-0.411419
86	1	0	7.971902	3.645140	-1.093917

44

Center Number	Atomic Number	Atomic Type	Coordinates (Angstroms) X	Y	Z
1	6	0	1.135205	0.233063	1.189317
2	6	0	-0.232972	0.952757	1.196936
3	1	0	1.196072	-0.334151	2.121981
4	1	0	1.933070	0.982985	1.228689
5	1	0	-0.299482	1.694088	0.395049
6	1	0	-0.330395	1.493694	2.145955
7	6	0	-3.616663	-0.683614	0.332207
8	6	0	-2.562647	-1.689256	-0.203722
9	6	0	-1.266854	-0.808867	-0.305343
10	6	0	-1.337597	-0.095589	1.063375
11	6	0	-3.057750	-2.405207	-1.440864
12	1	0	-2.333904	-2.466661	0.532632
13	8	0	-1.395533	0.212269	-1.315900
14	6	0	1.832059	-3.021296	-0.753621
15	6	0	2.401915	-1.927440	0.208747
16	6	0	1.365030	-0.711767	-0.029968
17	6	0	0.079198	-1.490923	-0.404853
18	6	0	0.358357	-2.731810	-0.817152
19	1	0	2.278608	-2.946944	-1.757141
20	1	0	2.045571	-4.035744	-0.397807
21	6	0	2.285530	-2.488188	1.644892
22	6	0	1.780964	0.160903	-1.243438
23	1	0	-0.373591	-3.450878	-1.170130
24	1	0	2.663240	-1.800819	2.406080
25	1	0	1.244520	-2.718280	1.891580
26	1	0	2.853900	-3.418845	1.733685
27	1	0	1.007260	0.901288	-1.456829
28	1	0	2.711993	0.700794	-1.050061
29	1	0	1.922834	-0.439954	-2.147055
30	6	0	3.899817	-1.617495	-0.138048
31	6	0	4.541227	-0.564334	0.796931
32	6	0	5.902993	-0.027977	0.351631
33	6	0	6.473608	1.084995	1.254177
34	6	0	6.035915	2.385040	0.561972
35	6	0	5.866924	1.950290	-0.890273
36	8	0	5.790646	0.601560	-0.970862
37	1	0	6.617732	-0.841572	0.218715
38	1	0	7.567275	1.026929	1.277077
39	1	0	6.110744	1.002591	2.281696
40	1	0	5.029338	2.669064	0.900877
41	6	0	6.966691	3.587063	0.730226
42	1	0	3.911776	-1.206207	-1.152737
43	1	0	4.692631	-0.998581	1.793666
44	1	0	3.868431	0.288240	0.932423
45	8	0	5.790176	2.652976	-1.877158
46	1	0	6.605706	4.440159	0.149491
47	1	0	7.017716	3.883033	1.782435
48	1	0	7.980680	3.347755	0.392771
49	1	0	-1.070221	-0.888680	1.775141
50	6	0	-2.842829	0.229454	1.380492
51	6	0	-3.109543	-0.215922	2.836424

52	6	0	-3.211273	1.739620	1.321429
53	1	0	-4.139351	-0.015266	3.147952
54	1	0	-2.926839	-1.286530	2.967968
55	1	0	-2.443834	0.324497	3.520133
56	6	0	-3.179390	2.481587	-0.043225
57	6	0	-4.523544	2.619587	-0.723655
58	8	0	-4.827161	2.143037	-1.805234
59	8	0	-5.378373	3.371237	0.004548
60	6	0	-6.689479	3.592983	-0.557267
61	6	0	-4.922141	-1.309788	0.802771
62	1	0	-3.871852	-0.040665	-0.519237
63	6	0	-5.050725	-2.603875	1.133994
64	6	0	-6.120077	-0.387858	0.842719
65	1	0	-6.010248	-3.003818	1.450952
66	1	0	-4.225921	-3.308161	1.105476
67	1	0	-7.019978	-0.917800	1.166294
68	1	0	-5.961776	0.454343	1.526315
69	1	0	-6.310522	0.041261	-0.148637
70	1	0	-2.503806	1.993058	-0.744098
71	1	0	-2.817653	3.501789	0.128757
72	1	0	-2.522702	2.255742	1.998866
73	1	0	-4.201003	1.875292	1.769252
74	8	0	-2.736784	-3.538693	-1.758535
75	1	0	-3.785461	-1.847799	-2.064192
76	6	0	-1.326186	-0.176749	-2.685283
77	1	0	-1.104260	0.729849	-3.253200
78	1	0	-0.536868	-0.914175	-2.862037
79	1	0	-2.283284	-0.577831	-3.040006
80	6	0	4.771972	-2.891752	-0.174574
81	1	0	5.766785	-2.670809	-0.573183
82	1	0	4.907840	-3.325300	0.822635
83	1	0	4.341771	-3.661736	-0.819676
84	1	0	-6.608386	4.106754	-1.517593
85	1	0	-7.211281	2.643469	-0.694517
86	1	0	-7.213054	4.215641	0.166990

45

Center Number	Atomic Number	Atomic Type	Coordinates (Angstroms) X	Y	Z
1	6	0	-0.897895	0.202577	-1.136623
2	6	0	0.487500	0.873668	-1.014314
3	1	0	-0.903063	-0.348992	-2.080760
4	1	0	-1.664013	0.980981	-1.224957
5	1	0	0.516591	1.572577	-0.173227
6	1	0	0.674317	1.457837	-1.923833
7	6	0	3.750321	-1.053488	-0.127141
8	6	0	2.586257	-1.972110	0.361554
9	6	0	1.359808	-0.997441	0.463193
10	6	0	1.554376	-0.209147	-0.851668
11	6	0	2.997233	-2.754719	1.585908
12	1	0	2.317058	-2.710025	-0.399518
13	8	0	1.518759	-0.044219	1.535539
14	6	0	-1.870266	-3.050635	0.679083
15	6	0	-2.334612	-1.901728	-0.275706
16	6	0	-1.248944	-0.749311	0.045443
17	6	0	-0.026992	-1.602692	0.464233
18	6	0	-0.389211	-2.838677	0.824088
19	1	0	-2.361780	-2.980204	1.661674
20	1	0	-2.117848	-4.042389	0.283414
21	6	0	-2.181514	-2.424701	-1.722580
22	6	0	-1.682377	0.121694	1.253864
23	1	0	0.285015	-3.605593	1.191462
24	1	0	-2.488148	-1.694288	-2.475903
25	1	0	-1.143717	-2.702540	-1.930790
26	1	0	-2.792264	-3.320703	-1.867808
27	1	0	-0.874404	0.798986	1.538314
28	1	0	-2.559694	0.729996	1.016720
29	1	0	-1.922957	-0.489565	2.129389
30	6	0	-3.827670	-1.520106	0.010620
31	6	0	-4.373414	-0.424684	-0.937359
32	6	0	-5.648774	0.268030	-0.475581
33	6	0	-6.219796	1.301980	-1.461094
34	6	0	-6.849087	2.369003	-0.549713
35	6	0	-6.049672	2.214984	0.741118
36	8	0	-5.371600	1.043832	0.740932
37	1	0	-6.415456	-0.461450	-0.195024
38	1	0	-6.935543	0.847569	-2.151491
39	1	0	-5.406084	1.735497	-2.052944
40	1	0	-6.688374	3.383037	-0.927078
41	6	0	-8.353354	2.174515	-0.277667
42	1	0	-3.863007	-1.122083	1.030132
43	1	0	-4.591411	-0.862179	-1.920800
44	1	0	-3.626293	0.355966	-1.106649
45	8	0	-5.995694	2.983368	1.679786
46	1	0	-8.710359	2.901316	0.457390
47	1	0	-8.918026	2.315270	-1.204184
48	1	0	-8.568781	1.169984	0.102247
49	1	0	1.356201	-0.950606	-1.639228
50	6	0	3.074345	0.123470	-0.976696
51	6	0	3.476916	0.155588	-2.464600

52	6	0	3.397991	1.505042	-0.340225
53	1	0	4.562253	0.176003	-2.596520
54	1	0	3.093064	-0.709419	-3.011796
55	1	0	3.065817	1.051340	-2.944609
56	6	0	4.873585	1.958035	-0.443046
57	6	0	5.076362	3.253995	0.306428
58	8	0	5.353377	3.342970	1.491058
59	8	0	4.880890	4.330918	-0.483594
60	6	0	4.996743	5.624462	0.148570
61	6	0	4.894472	-1.842962	-0.756361
62	1	0	4.163675	-0.577339	0.768800
63	6	0	6.161888	-1.628602	-0.369879
64	6	0	4.580775	-2.928067	-1.765383
65	1	0	6.988721	-2.187417	-0.800645
66	1	0	6.415547	-0.897222	0.392888
67	1	0	5.493016	-3.273739	-2.258999
68	1	0	4.122405	-3.796719	-1.276447
69	1	0	3.880568	-2.596037	-2.536869
70	1	0	5.166225	2.100237	-1.485943
71	1	0	5.538385	1.218722	0.008166
72	1	0	3.103104	1.485872	0.711364
73	1	0	2.779367	2.263809	-0.833562
74	8	0	2.690551	-3.916612	1.798593
75	1	0	3.658355	-2.224175	2.300258
76	6	0	1.290775	-0.477330	2.873727
77	1	0	0.388688	-1.090576	2.961102
78	1	0	2.144630	-1.039063	3.272492
79	1	0	1.167952	0.429097	3.471295
80	6	0	-4.765195	-2.747168	-0.012289
81	1	0	-5.779929	-2.466954	0.287541
82	1	0	-4.832755	-3.192817	-1.010841
83	1	0	-4.436654	-3.524900	0.681749
84	1	0	4.801674	6.350166	-0.639744
85	1	0	4.262564	5.721023	0.951277
86	1	0	6.001668	5.758798	0.554549

46

Center Number	Atomic Number	Atomic Type	Coordinates (Angstroms) X	Y	Z
1	6	0	-0.966100	0.219786	-1.149812
2	6	0	0.431303	0.869918	-1.053402
3	1	0	-0.986776	-0.355610	-2.079538
4	1	0	-1.719568	1.008580	-1.253663
5	1	0	0.479215	1.590915	-0.232166
6	1	0	0.622485	1.426221	-1.979289
7	6	0	3.666011	-1.084408	-0.132227
8	6	0	2.490987	-1.972940	0.385068
9	6	0	1.281176	-0.975821	0.468375
10	6	0	1.479937	-0.226858	-0.868571
11	6	0	2.895854	-2.729448	1.627254
12	1	0	2.205805	-2.725958	-0.355099
13	8	0	1.462765	0.001678	1.515139
14	6	0	-1.981189	-2.968183	0.757129
15	6	0	-2.431422	-1.838043	-0.226387
16	6	0	-1.325715	-0.695395	0.058680
17	6	0	-0.115307	-1.557674	0.493603
18	6	0	-0.496012	-2.777276	0.888707
19	1	0	-2.466203	-2.862647	1.739796
20	1	0	-2.247361	-3.965939	0.389635
21	6	0	-2.293320	-2.402704	-1.659041
22	6	0	-1.737077	0.213449	1.246786
23	1	0	0.167061	-3.545496	1.273172
24	1	0	-2.593712	-1.689152	-2.430808
25	1	0	-1.260426	-2.700152	-1.864473
26	1	0	-2.917114	-3.293865	-1.776564
27	1	0	-0.917555	0.886684	1.507120
28	1	0	-2.607394	0.828062	1.000305
29	1	0	-1.979836	-0.371217	2.139699
30	6	0	-3.916476	-1.424443	0.056599
31	6	0	-4.446946	-0.342025	-0.914194
32	6	0	-5.723135	0.364974	-0.477205
33	6	0	-6.266858	1.400173	-1.476747
34	6	0	-6.903556	2.475620	-0.580512
35	6	0	-6.129501	2.319186	0.725455
36	8	0	-5.460371	1.142940	0.740984
37	1	0	-6.501856	-0.356007	-0.207991
38	1	0	-6.973873	0.949570	-2.178542
39	1	0	-5.438751	1.824575	-2.055022
40	1	0	-6.727434	3.486989	-0.958095
41	6	0	-8.414079	2.294015	-0.335743
42	1	0	-3.939904	-1.001376	1.066384
43	1	0	-4.657904	-0.793401	-1.892885
44	1	0	-3.693234	0.431212	-1.087860
45	8	0	-6.087747	3.089460	1.663154
46	1	0	-8.779133	3.026250	0.389901
47	1	0	-8.960313	2.435384	-1.273159
48	1	0	-8.644026	1.292539	0.043735
49	1	0	1.261726	-0.986577	-1.633107
50	6	0	3.003811	0.076241	-1.015205
51	6	0	3.391842	0.047684	-2.507840

52	6	0	3.357935	1.474116	-0.433412
53	1	0	4.475846	0.041977	-2.652217
54	1	0	2.985742	-0.827768	-3.021601
55	1	0	2.992205	0.934044	-3.014314
56	6	0	4.831498	1.883493	-0.544239
57	6	0	5.104056	3.260335	0.023542
58	8	0	4.267463	4.109369	0.275605
59	8	0	6.427978	3.459852	0.204664
60	6	0	6.822622	4.758679	0.697533
61	6	0	4.800295	-1.905926	-0.737281
62	1	0	4.084842	-0.587427	0.749882
63	6	0	6.070333	-1.696075	-0.356763
64	6	0	4.473982	-3.014565	-1.716096
65	1	0	6.890720	-2.276150	-0.771392
66	1	0	6.332810	-0.945861	0.384484
67	1	0	5.382252	-3.385402	-2.198680
68	1	0	4.003899	-3.863399	-1.204078
69	1	0	3.779100	-2.694399	-2.497247
70	1	0	5.163922	1.913143	-1.589589
71	1	0	5.499062	1.177240	-0.042895
72	1	0	3.052359	1.510492	0.614624
73	1	0	2.758195	2.227127	-0.955440
74	8	0	2.577193	-3.882209	1.870093
75	1	0	3.564276	-2.188618	2.327139
76	6	0	1.233034	-0.392605	2.864774
77	1	0	1.110933	0.530305	3.436876
78	1	0	0.330091	-1.001860	2.969315
79	1	0	2.085815	-0.944688	3.279514
80	6	0	-4.874458	-2.635778	0.067979
81	1	0	-5.879413	-2.334102	0.379340
82	1	0	-4.965212	-3.096656	-0.921877
83	1	0	-4.547697	-3.407626	0.769346
84	1	0	6.377796	4.943984	1.677553
85	1	0	7.908697	4.722255	0.770621
86	1	0	6.510438	5.540081	0.001198

47

Center Number	Atomic Number	Atomic Type	Coordinates (Angstroms) X	Y	Z
1	6	0	-0.896417	0.027357	-1.187229
2	6	0	0.471910	0.745744	-1.179569
3	1	0	-0.930549	-0.574374	-2.099433
4	1	0	-1.692617	0.774692	-1.277848
5	1	0	0.514976	1.509428	-0.397995
6	1	0	0.599799	1.260394	-2.139281
7	6	0	3.826904	-0.892369	-0.195800
8	6	0	2.753454	-1.880586	0.344771
9	6	0	1.466371	-0.976318	0.398806
10	6	0	1.570273	-0.300213	-0.986583
11	6	0	3.213152	-2.557480	1.613550
12	1	0	2.537814	-2.678441	-0.373319
13	8	0	1.597473	0.072004	1.381630
14	6	0	-1.656058	-3.152958	0.853031
15	6	0	-2.197488	-2.090032	-0.159603
16	6	0	-1.160158	-0.871384	0.059827
17	6	0	0.112536	-1.642706	0.492904
18	6	0	-0.183189	-2.866812	0.942238
19	1	0	-2.126757	-3.044014	1.842260
20	1	0	-1.864722	-4.178025	0.525986
21	6	0	-2.050606	-2.700595	-1.572477
22	6	0	-1.598684	0.045457	1.232043
23	1	0	0.535168	-3.578342	1.336355
24	1	0	-2.407396	-2.038297	-2.365316
25	1	0	-1.005075	-2.942302	-1.786929
26	1	0	-2.620750	-3.631773	-1.642123
27	1	0	-0.825369	0.787980	1.440205
28	1	0	-2.520054	0.585350	0.997396
29	1	0	-1.768028	-0.523744	2.151150
30	6	0	-3.701126	-1.762544	0.142837
31	6	0	-4.313940	-0.730765	-0.833673
32	6	0	-5.697895	-0.201846	-0.452491
33	6	0	-6.229581	0.911432	-1.378241
34	6	0	-5.837995	2.211773	-0.659195
35	6	0	-5.734101	1.771562	0.797401
36	8	0	-5.652962	0.423111	0.876066
37	1	0	-6.413807	-1.019649	-0.357480
38	1	0	-7.320131	0.845571	-1.457735
39	1	0	-5.813836	0.836489	-2.386089
40	1	0	-4.819467	2.508021	-0.948058
41	6	0	-6.772085	3.405391	-0.865265
42	1	0	-3.733689	-1.323739	1.145520
43	1	0	-4.421488	-1.180232	-1.829326
44	1	0	-3.644243	0.126318	-0.954967
45	8	0	-5.708145	2.470367	1.789740
46	1	0	-6.448740	4.258226	-0.262388
47	1	0	-6.774961	3.707884	-1.916836
48	1	0	-7.798711	3.153375	-0.579128
49	1	0	1.317101	-1.112643	-1.681687
50	6	0	3.080453	0.007912	-1.273242
51	6	0	3.377276	-0.420647	-2.728895

52	6	0	3.503779	1.500651	-1.196544
53	1	0	4.436865	-0.304176	-2.977324
54	1	0	3.093444	-1.460023	-2.916101
55	1	0	2.802035	0.203587	-3.422732
56	6	0	3.431308	2.238320	0.145024
57	6	0	3.655508	3.728090	0.001871
58	8	0	3.582766	4.371055	-1.031285
59	8	0	3.930399	4.295503	1.197043
60	6	0	4.110826	5.728109	1.205111
61	6	0	5.148589	-1.527001	-0.612517
62	1	0	4.060755	-0.231096	0.643250
63	6	0	6.303705	-0.898094	-0.341573
64	6	0	5.152897	-2.876969	-1.296361
65	1	0	7.262001	-1.316290	-0.638510
66	1	0	6.328135	0.053566	0.183313
67	1	0	6.168612	-3.159144	-1.586574
68	1	0	4.768546	-3.660523	-0.631965
69	1	0	4.527674	-2.893800	-2.194408
70	1	0	4.163293	1.864941	0.867445
71	1	0	2.458108	2.102266	0.627658
72	1	0	2.900222	2.054417	-1.923179
73	1	0	4.535611	1.567894	-1.564723
74	8	0	2.960702	-3.714139	1.910308
75	1	0	3.858990	-1.952717	2.281297
76	6	0	1.453342	-0.261652	2.759693
77	1	0	2.376134	-0.687360	3.172838
78	1	0	1.242849	0.674073	3.283352
79	1	0	0.629043	-0.960948	2.930338
80	6	0	-4.581222	-3.030811	0.194662
81	1	0	-5.579035	-2.795865	0.577293
82	1	0	-4.707974	-3.484120	-0.794971
83	1	0	-4.162901	-3.789957	0.860043
84	1	0	4.314159	5.987828	2.243134
85	1	0	4.952011	6.008654	0.567534
86	1	0	3.204652	6.227341	0.854978

48

Center Number	Atomic Number	Atomic Type	Coordinates (Angstroms) X	Y	Z
1	6	0	-0.812626	0.147667	-1.064949
2	6	0	0.550431	0.834719	-0.828151
3	1	0	-0.774803	-0.294383	-2.064417
4	1	0	-1.597091	0.912399	-1.092279
5	1	0	0.537548	1.442209	0.082117
6	1	0	0.735520	1.511534	-1.669795
7	6	0	3.823842	-1.076144	0.031227
8	6	0	2.684357	-2.098176	0.296485
9	6	0	1.424498	-1.171694	0.454377
10	6	0	1.648518	-0.228580	-0.750074
11	6	0	3.046858	-3.032029	1.426278
12	1	0	2.487868	-2.728007	-0.576634
13	8	0	1.513574	-0.338207	1.630644
14	6	0	-1.748338	-3.317861	0.314046
15	6	0	-2.223563	-2.067967	-0.498436
16	6	0	-1.173519	-0.940731	-0.012342
17	6	0	0.058270	-1.809008	0.335682
18	6	0	-0.278329	-3.089100	0.526739
19	1	0	-2.265158	-3.387998	1.283307
20	1	0	-1.956084	-4.256288	-0.212868
21	6	0	-2.028759	-2.403595	-1.995181
22	6	0	-1.658406	-0.228929	1.278045
23	1	0	0.407135	-3.879565	0.814123
24	1	0	-2.333284	-1.590301	-2.658974
25	1	0	-0.981118	-2.634829	-2.211598
26	1	0	-2.617043	-3.285626	-2.265599
27	1	0	-0.878646	0.433063	1.659741
28	1	0	-2.547905	0.379203	1.091417
29	1	0	-1.901606	-0.944251	2.069754
30	6	0	-3.731802	-1.761667	-0.197707
31	6	0	-4.283906	-0.552435	-0.989744
32	6	0	-5.659031	-0.048244	-0.547872
33	6	0	-6.131988	1.227199	-1.274619
34	6	0	-5.705721	2.367393	-0.337074
35	6	0	-5.657065	1.680144	1.023699
36	8	0	-5.625718	0.335938	0.869848
37	1	0	-6.403895	-0.843270	-0.608540
38	1	0	-7.222681	1.219215	-1.376768
39	1	0	-5.700978	1.309849	-2.275426
40	1	0	-4.668302	2.660801	-0.552848
41	6	0	-6.583703	3.619671	-0.352355
42	1	0	-3.797640	-1.512370	0.866484
43	1	0	-4.379731	-0.817273	-2.050747
44	1	0	-3.583348	0.287305	-0.944384
45	8	0	-5.632492	2.196601	2.121953
46	1	0	-6.244490	4.339138	0.397758
47	1	0	-6.542571	4.099968	-1.334649
48	1	0	-7.627884	3.368500	-0.138022
49	1	0	1.497129	-0.875901	-1.626348
50	6	0	3.168541	0.138801	-0.781902
51	6	0	3.634983	0.220793	-2.247501

52	6	0	3.577930	1.436875	-0.026032
53	1	0	4.703939	0.446719	-2.320363
54	1	0	3.448802	-0.713492	-2.783377
55	1	0	3.088018	1.004673	-2.781933
56	6	0	3.284910	2.782882	-0.701070
57	6	0	3.796999	3.959239	0.104613
58	8	0	4.561890	3.903052	1.051066
59	8	0	3.308369	5.124154	-0.374553
60	6	0	3.756723	6.330118	0.281464
61	6	0	5.109613	-1.679355	-0.519963
62	1	0	4.080882	-0.659196	1.010512
63	6	0	6.297815	-1.251367	-0.063480
64	6	0	5.037720	-2.792398	-1.543174
65	1	0	7.232081	-1.653302	-0.447188
66	1	0	6.373685	-0.488352	0.706816
67	1	0	6.034164	-3.033940	-1.922974
68	1	0	4.621723	-3.706516	-1.101587
69	1	0	4.401173	-2.541344	-2.397297
70	1	0	2.219366	2.945757	-0.879214
71	1	0	3.768310	2.854824	-1.683210
72	1	0	4.660723	1.386889	0.137877
73	1	0	3.115200	1.423082	0.964231
74	8	0	2.779822	-4.222720	1.451763
75	1	0	3.635442	-2.588276	2.253940
76	6	0	1.273564	-0.928528	2.905582
77	1	0	1.013914	-0.110442	3.581953
78	1	0	0.447941	-1.646646	2.879030
79	1	0	2.169828	-1.426105	3.296453
80	6	0	-4.637448	-2.993639	-0.416047
81	1	0	-5.647627	-2.804975	-0.040474
82	1	0	-4.725994	-3.255696	-1.476391
83	1	0	-4.265049	-3.871933	0.116785
84	1	0	4.841626	6.426107	0.199368
85	1	0	3.259526	7.145654	-0.241924
86	1	0	3.469614	6.318514	1.335021

49

Center Number	Atomic Number	Atomic Type	Coordinates (Angstroms) X	Y	Z
1	6	0	-0.969899	-0.050773	-1.173262
2	6	0	0.401830	0.657211	-1.199901
3	1	0	-1.021032	-0.673192	-2.070543
4	1	0	-1.762358	0.699712	-1.269552
5	1	0	0.460833	1.439551	-0.438096
6	1	0	0.520264	1.146979	-2.173695
7	6	0	3.775178	-1.008086	-0.274963
8	6	0	2.704012	-1.923544	0.366847
9	6	0	1.411553	-1.030308	0.408088
10	6	0	1.496923	-0.388342	-0.993616
11	6	0	3.150203	-2.565164	1.660567
12	1	0	2.447382	-2.764799	-0.292448
13	8	0	1.550036	0.042717	1.362793
14	6	0	-1.733241	-3.173282	0.948760
15	6	0	-2.275627	-2.129132	-0.081009
16	6	0	-1.222155	-0.917506	0.097401
17	6	0	0.052095	-1.689193	0.531696
18	6	0	-0.256835	-2.898987	1.010361
19	1	0	-2.187714	-3.035386	1.941695
20	1	0	-1.956942	-4.203529	0.649189
21	6	0	-2.153065	-2.773733	-1.481282
22	6	0	-1.636607	0.029955	1.253698
23	1	0	0.446503	-3.618828	1.417384
24	1	0	-2.513064	-2.125858	-2.284402
25	1	0	-1.112677	-3.030472	-1.703582
26	1	0	-2.732950	-3.700664	-1.523222
27	1	0	-0.854100	0.769453	1.435976
28	1	0	-2.555902	0.572042	1.016594
29	1	0	-1.800870	-0.516251	2.187663
30	6	0	-3.771439	-1.776445	0.231693
31	6	0	-4.390679	-0.779945	-0.777342
32	6	0	-5.737553	-0.180234	-0.368902
33	6	0	-6.283465	0.880955	-1.346194
34	6	0	-5.809646	2.214809	-0.748104
35	6	0	-5.645158	1.878727	0.730508
36	8	0	-5.604734	0.537247	0.905924
37	1	0	-6.471649	-0.964663	-0.177983
38	1	0	-7.378354	0.849627	-1.361915
39	1	0	-5.927333	0.717069	-2.366322
40	1	0	-4.797695	2.447905	-1.108673
41	6	0	-6.709976	3.426029	-0.996948
42	1	0	-3.784715	-1.297150	1.216092
43	1	0	-4.555855	-1.281968	-1.739348
44	1	0	-3.698683	0.044110	-0.976286
45	8	0	-5.543812	2.646491	1.665151
46	1	0	-6.328869	4.305632	-0.471151
47	1	0	-6.752112	3.654486	-2.066197
48	1	0	-7.730006	3.233732	-0.647895
49	1	0	1.237250	-1.218736	-1.665614
50	6	0	3.000224	-0.079831	-1.313635
51	6	0	3.244629	-0.502835	-2.780409

52	6	0	3.408137	1.419013	-1.238223
53	1	0	4.276399	-0.320307	-3.095990
54	1	0	3.035833	-1.565366	-2.933647
55	1	0	2.587068	0.070102	-3.445205
56	6	0	3.403584	2.135853	0.115445
57	6	0	3.698054	3.614345	-0.009885
58	8	0	3.723935	4.259565	-1.044146
59	8	0	3.920334	4.169704	1.201972
60	6	0	4.175729	5.590299	1.223613
61	6	0	5.010697	-1.717917	-0.813526
62	1	0	4.128462	-0.363536	0.535265
63	6	0	5.020005	-2.982967	-1.256654
64	6	0	6.276496	-0.891867	-0.814232
65	1	0	5.933087	-3.436467	-1.633070
66	1	0	4.136559	-3.614342	-1.260467
67	1	0	7.122990	-1.451738	-1.221428
68	1	0	6.159848	0.024286	-1.406132
69	1	0	6.528830	-0.577227	0.206379
70	1	0	4.136505	1.715621	0.811521
71	1	0	2.439786	2.033332	0.623097
72	1	0	2.757109	1.974235	-1.922098
73	1	0	4.414016	1.512530	-1.664514
74	8	0	4.212652	-2.347881	2.216926
75	1	0	2.449480	-3.303161	2.098562
76	6	0	1.431969	-0.257375	2.752835
77	1	0	2.350990	-0.697213	3.153066
78	1	0	1.257145	0.696664	3.256191
79	1	0	0.589430	-0.926051	2.957245
80	6	0	-4.661918	-3.032951	0.347238
81	1	0	-5.660487	-2.769429	0.708853
82	1	0	-4.786571	-3.539225	-0.616582
83	1	0	-4.254655	-3.757368	1.056910
84	1	0	4.324876	5.841234	2.272956
85	1	0	5.070258	5.824688	0.642318
86	1	0	3.322068	6.136508	0.816157

50

Center Number	Atomic Number	Atomic Type	Coordinates (Angstroms) X	Y	Z
1	6	0	-1.088419	0.139022	-1.140020
2	6	0	0.305630	0.796688	-1.063215
3	1	0	-1.111045	-0.452566	-2.059396
4	1	0	-1.848335	0.920223	-1.253910
5	1	0	0.355152	1.533236	-0.256250
6	1	0	0.490206	1.335216	-2.000696
7	6	0	3.563512	-1.171384	-0.204937
8	6	0	2.406797	-1.979648	0.452179
9	6	0	1.176147	-1.006411	0.497814
10	6	0	1.359055	-0.292201	-0.860266
11	6	0	2.800336	-2.674975	1.734662
12	1	0	2.079727	-2.795946	-0.206542
13	8	0	1.331133	0.004462	1.516806
14	6	0	-2.082854	-3.018826	0.823028
15	6	0	-2.536482	-1.909264	-0.181655
16	6	0	-1.436438	-0.757840	0.084866
17	6	0	-0.217487	-1.603612	0.534935
18	6	0	-0.599366	-2.815385	0.952301
19	1	0	-2.571108	-2.899666	1.802319
20	1	0	-2.339637	-4.024755	0.471829
21	6	0	-2.392823	-2.502883	-1.602062
22	6	0	-1.847777	0.163855	1.263172
23	1	0	0.054432	-3.581653	1.357200
24	1	0	-2.693017	-1.807261	-2.389826
25	1	0	-1.358547	-2.801271	-1.799333
26	1	0	-3.014524	-3.397758	-1.701393
27	1	0	-1.031871	0.845872	1.510527
28	1	0	-2.722368	0.770680	1.013204
29	1	0	-2.084279	-0.410185	2.164301
30	6	0	-4.024586	-1.499329	0.093940
31	6	0	-4.553425	-0.412888	-0.871641
32	6	0	-5.920600	0.175998	-0.517415
33	6	0	-6.345033	1.366836	-1.401324
34	6	0	-5.907782	2.600332	-0.595930
35	6	0	-5.901574	2.079209	0.837277
36	8	0	-5.899498	0.725587	0.844353
37	1	0	-6.685774	-0.601728	-0.497937
38	1	0	-7.433090	1.372624	-1.528337
39	1	0	-5.890556	1.319509	-2.394066
40	1	0	-4.859729	2.842567	-0.822732
41	6	0	-6.752791	3.862276	-0.777421
42	1	0	-4.053915	-1.079710	1.104680
43	1	0	-4.653137	-0.829606	-1.882264
44	1	0	-3.837942	0.411382	-0.949840
45	8	0	-5.886636	2.721607	1.866971
46	1	0	-6.405086	4.658614	-0.113860
47	1	0	-6.685503	4.219375	-1.809524
48	1	0	-7.806051	3.664585	-0.551854
49	1	0	1.137386	-1.073940	-1.602683
50	6	0	2.877189	0.013909	-1.035046
51	6	0	3.223496	0.027667	-2.538080

52	6	0	3.247830	1.396331	-0.426001
53	1	0	4.301769	0.066235	-2.711885
54	1	0	2.840802	-0.855195	-3.057483
55	1	0	2.775853	0.906456	-3.016696
56	6	0	4.720296	1.805125	-0.554720
57	6	0	5.001692	3.177517	0.019696
58	8	0	4.171760	4.035218	0.265213
59	8	0	6.325765	3.363480	0.212815
60	6	0	6.728897	4.656744	0.712824
61	6	0	4.558671	-2.052022	-0.956468
62	1	0	4.122694	-0.709956	0.613005
63	6	0	5.879094	-1.853953	-0.830438
64	6	0	4.043610	-3.184900	-1.820673
65	1	0	6.598774	-2.463850	-1.370712
66	1	0	6.285604	-1.088075	-0.176052
67	1	0	4.837157	-3.572891	-2.465090
68	1	0	3.687284	-4.020621	-1.204749
69	1	0	3.205496	-2.884980	-2.458089
70	1	0	5.039422	1.843831	-1.603949
71	1	0	5.393334	1.095316	-0.065380
72	1	0	2.965418	1.407787	0.628586
73	1	0	2.640010	2.163922	-0.916784
74	8	0	3.891580	-2.585567	2.270858
75	1	0	2.030895	-3.337611	2.178247
76	6	0	1.141200	-0.366202	2.880157
77	1	0	2.011856	-0.893933	3.284654
78	1	0	1.017672	0.567429	3.434222
79	1	0	0.247055	-0.982890	3.018385
80	6	0	-4.979892	-2.713127	0.098884
81	1	0	-5.966041	-2.428453	0.477668
82	1	0	-5.121575	-3.129306	-0.905009
83	1	0	-4.616347	-3.515401	0.745703
84	1	0	6.430790	5.443344	0.016085
85	1	0	6.277267	4.843531	1.689438
86	1	0	7.813858	4.609223	0.795592

51

Center Number	Atomic Number	Atomic Type	Coordinates (Angstroms) X	Y	Z
1	6	0	0.891370	0.191293	1.141609
2	6	0	-0.496099	0.854591	1.006103
3	1	0	0.895601	-0.349685	2.091785
4	1	0	1.653332	0.974449	1.224138
5	1	0	-0.523626	1.543627	0.157010
6	1	0	-0.688396	1.448744	1.908007
7	6	0	-3.747385	-1.103049	0.138538
8	6	0	-2.577296	-2.015020	-0.335855
9	6	0	-1.354743	-1.039752	-0.450953
10	6	0	-1.558480	-0.234213	0.851397
11	6	0	-2.983120	-2.830688	-1.542231
12	1	0	-2.295035	-2.734948	0.435980
13	8	0	-1.512480	-0.103995	-1.538800
14	6	0	1.888807	-3.077527	-0.628767
15	6	0	2.344009	-1.912459	0.310493
16	6	0	1.251084	-0.772002	-0.028108
17	6	0	0.035602	-1.638548	-0.438472
18	6	0	0.406785	-2.877020	-0.780376
19	1	0	2.382183	-3.018455	-1.611196
20	1	0	2.141611	-4.062061	-0.218475
21	6	0	2.192678	-2.416801	1.764227
22	6	0	1.680603	0.086171	-1.247061
23	1	0	-0.262162	-3.652266	-1.139333
24	1	0	2.491814	-1.673648	2.508060
25	1	0	1.156881	-2.700562	1.974509
26	1	0	2.810642	-3.305619	1.922528
27	1	0	0.866242	0.749914	-1.544750
28	1	0	2.550627	0.707211	-1.015580
29	1	0	1.930886	-0.535484	-2.112573
30	6	0	3.834793	-1.524190	0.021193
31	6	0	4.371629	-0.413158	0.956045
32	6	0	5.641592	0.284089	0.486190
33	6	0	6.201863	1.337405	1.457346
34	6	0	6.822627	2.397038	0.531579
35	6	0	6.027400	2.216406	-0.758417
36	8	0	5.359532	1.039594	-0.741860
37	1	0	6.415010	-0.442741	0.217321
38	1	0	6.920821	0.899797	2.155224
39	1	0	5.383326	1.771837	2.041831
40	1	0	6.651210	3.414858	0.893727
41	6	0	8.329347	2.213372	0.265603
42	1	0	3.868647	-1.138533	-1.003079
43	1	0	4.592923	-0.836935	1.944768
44	1	0	3.618170	0.363368	1.115797
45	8	0	5.968798	2.970011	-1.708749
46	1	0	8.681026	2.932909	-0.479112
47	1	0	8.890509	2.373006	1.191199
48	1	0	8.555403	1.205576	-0.099218
49	1	0	-1.365463	-0.963151	1.651738
50	6	0	-3.081141	0.101618	0.960491
51	6	0	-3.478217	0.124858	2.448404

52	6	0	-3.372634	1.483555	0.310260
53	1	0	-4.543785	0.318367	2.599139
54	1	0	-3.249978	-0.826903	2.936274
55	1	0	-2.923416	0.913575	2.969866
56	6	0	-4.849525	1.936877	0.273739
57	6	0	-4.963093	3.286676	-0.396664
58	8	0	-5.060483	3.464358	-1.599342
59	8	0	-4.912794	4.299512	0.493642
60	6	0	-4.962271	5.637943	-0.047879
61	6	0	-4.882332	-1.873150	0.814272
62	1	0	-4.175934	-0.655906	-0.768307
63	6	0	-4.706728	-3.024045	1.481836
64	6	0	-6.271979	-1.291232	0.677259
65	1	0	-5.550476	-3.534139	1.938735
66	1	0	-3.739979	-3.500463	1.606120
67	1	0	-6.523780	-1.118834	-0.376966
68	1	0	-7.024073	-1.961135	1.102545
69	1	0	-6.361737	-0.324668	1.185265
70	1	0	-5.268226	2.004924	1.280623
71	1	0	-5.450552	1.241050	-0.315175
72	1	0	-2.986627	1.475380	-0.711233
73	1	0	-2.803808	2.240481	0.863057
74	8	0	-2.606636	-3.968595	-1.768744
75	1	0	-3.705186	-2.346489	-2.231625
76	6	0	-1.303708	-0.569482	-2.868582
77	1	0	-1.201344	0.322117	-3.491663
78	1	0	-2.158824	-1.150390	-3.236914
79	1	0	-0.396821	-1.176065	-2.955837
80	6	0	4.780794	-2.744240	0.060598
81	1	0	5.794332	-2.460407	-0.239912
82	1	0	4.848845	-3.177924	1.064376
83	1	0	4.459121	-3.532108	-0.625176
84	1	0	-5.897311	5.791911	-0.590655
85	1	0	-4.905820	6.300783	0.814397
86	1	0	-4.117695	5.806661	-0.719329

52

Center Number	Atomic Number	Atomic Type	Coordinates (Angstroms) X	Y	Z
1	6	0	-0.936633	0.149331	-1.129585
2	6	0	0.449769	0.819043	-1.022032
3	1	0	-0.947261	-0.413544	-2.066971
4	1	0	-1.702271	0.927451	-1.223237
5	1	0	0.482941	1.530297	-0.191694
6	1	0	0.633455	1.389384	-1.940726
7	6	0	3.719873	-1.139307	-0.196655
8	6	0	2.565118	-1.985050	0.407913
9	6	0	1.327425	-1.029567	0.482256
10	6	0	1.515023	-0.262938	-0.845957
11	6	0	2.965403	-2.741024	1.653494
12	1	0	2.259499	-2.776586	-0.290155
13	8	0	1.466887	-0.053770	1.538357
14	6	0	-1.916580	-3.075287	0.729530
15	6	0	-2.375683	-1.938291	-0.240577
16	6	0	-1.284275	-0.787376	0.065083
17	6	0	-0.061406	-1.638301	0.491418
18	6	0	-0.434436	-2.866557	0.866801
19	1	0	-2.405011	-2.987402	1.712123
20	1	0	-2.169200	-4.071512	0.348761
21	6	0	-2.225915	-2.482341	-1.680072
22	6	0	-1.709742	0.096916	1.266699
23	1	0	0.224544	-3.641085	1.246624
24	1	0	-2.531756	-1.762027	-2.443182
25	1	0	-1.189161	-2.765362	-1.886448
26	1	0	-2.839456	-3.378574	-1.811622
27	1	0	-0.898182	0.773979	1.540486
28	1	0	-2.586741	0.704576	1.026822
29	1	0	-1.948020	-0.505248	2.149214
30	6	0	-3.866882	-1.545994	0.041648
31	6	0	-4.407607	-0.460788	-0.920601
32	6	0	-5.684363	0.238569	-0.472921
33	6	0	-6.244451	1.266675	-1.470869
34	6	0	-6.876119	2.343461	-0.572724
35	6	0	-6.087708	2.196190	0.725641
36	8	0	-5.413814	1.022471	0.739631
37	1	0	-6.455465	-0.486500	-0.192984
38	1	0	-6.957081	0.809391	-2.162577
39	1	0	-5.424962	1.692246	-2.060461
40	1	0	-6.707917	3.353785	-0.956709
41	6	0	-8.383306	2.157493	-0.311356
42	1	0	-3.899525	-1.134092	1.055708
43	1	0	-4.621335	-0.908445	-1.900368
44	1	0	-3.658995	0.317265	-1.094406
45	8	0	-6.038584	2.971099	1.659084
46	1	0	-8.743233	2.891570	0.414973
47	1	0	-8.939775	2.293145	-1.243575
48	1	0	-8.605835	1.156855	0.074669
49	1	0	1.315339	-1.018604	-1.620549
50	6	0	3.034081	0.070281	-0.989420
51	6	0	3.390157	0.125422	-2.487655

52	6	0	3.360972	1.439606	-0.328483
53	1	0	4.459620	0.273854	-2.661027
54	1	0	3.106442	-0.796457	-3.001739
55	1	0	2.859981	0.957347	-2.965772
56	6	0	4.842939	1.878869	-0.367167
57	6	0	5.007889	3.215076	0.319289
58	8	0	5.153376	3.373671	1.519591
59	8	0	4.943719	4.242986	-0.554004
60	6	0	5.039580	5.571261	0.005243
61	6	0	4.743022	-1.974839	-0.966672
62	1	0	4.256168	-0.706176	0.653247
63	6	0	4.420129	-3.019067	-1.743940
64	6	0	6.187686	-1.562815	-0.806285
65	1	0	5.186017	-3.578509	-2.274415
66	1	0	3.397834	-3.355075	-1.892313
67	1	0	6.859326	-2.235402	-1.346883
68	1	0	6.359618	-0.545821	-1.178699
69	1	0	6.473020	-1.564336	0.252949
70	1	0	5.205621	1.961969	-1.394361
71	1	0	5.468737	1.165106	0.173913
72	1	0	3.032186	1.415144	0.712144
73	1	0	2.768105	2.210423	-0.835401
74	8	0	4.081563	-2.729931	2.143957
75	1	0	2.179389	-3.375866	2.107120
76	6	0	1.300497	-0.483879	2.886444
77	1	0	0.412659	-1.113054	3.010909
78	1	0	2.181679	-1.021422	3.255232
79	1	0	1.179742	0.423802	3.482495
80	6	0	-4.809925	-2.769024	0.036075
81	1	0	-5.822049	-2.480653	0.336688
82	1	0	-4.883598	-3.225759	-0.957032
83	1	0	-4.482659	-3.540430	0.737802
84	1	0	4.965919	6.248665	-0.844403
85	1	0	4.223076	5.746473	0.709073
86	1	0	5.996211	5.698518	0.516448

53

Center Number	Atomic Number	Atomic Type	Coordinates (Angstroms) X	Y	Z
1	6	0	-1.126052	-0.247861	1.234811
2	6	0	0.224585	-0.995115	1.276918
3	1	0	-1.149969	0.416585	2.102799
4	1	0	-1.937950	-0.970871	1.372572
5	1	0	0.258585	-1.794780	0.531693
6	1	0	0.332000	-1.466986	2.261424
7	6	0	3.631587	0.644638	0.349030
8	6	0	2.547325	1.535243	-0.335208
9	6	0	1.272738	0.618936	-0.376171
10	6	0	1.356568	0.005452	1.039471
11	6	0	3.066893	2.120009	-1.626907
12	1	0	2.275703	2.385451	0.296833
13	8	0	1.419460	-0.475311	-1.303480
14	6	0	-1.816951	2.795920	-1.022117
15	6	0	-2.391577	1.805632	0.043628
16	6	0	-1.362966	0.564982	-0.073065
17	6	0	-0.074332	1.291848	-0.529860
18	6	0	-0.346077	2.488652	-1.060939
19	1	0	-2.266330	2.627840	-2.012751
20	1	0	-2.022540	3.841878	-0.766376
21	6	0	-2.270276	2.502291	1.418780
22	6	0	-1.793076	-0.427047	-1.185660
23	1	0	0.388583	3.164985	-1.486002
24	1	0	-2.650132	1.892304	2.242468
25	1	0	-1.227202	2.748275	1.640685
26	1	0	-2.831871	3.441453	1.419672
27	1	0	-1.019619	-1.182946	-1.338474
28	1	0	-2.719761	-0.944997	-0.923376
29	1	0	-1.950076	0.080956	-2.142489
30	6	0	-3.890698	1.470638	-0.268268
31	6	0	-4.545589	0.550759	0.790651
32	6	0	-5.835780	-0.132971	0.357938
33	6	0	-6.514223	-0.988160	1.441535
34	6	0	-7.165381	-2.133870	0.648794
35	6	0	-6.295629	-2.205224	-0.603149
36	8	0	-5.546619	-1.084716	-0.723579
37	1	0	-6.543855	0.588767	-0.062325
38	1	0	-7.231933	-0.404766	2.024606
39	1	0	-5.757227	-1.380290	2.129776
40	1	0	-7.088880	-3.093381	1.168269
41	6	0	-8.638860	-1.892171	0.267753
42	1	0	-3.903619	0.934623	-1.223128
43	1	0	-4.784318	1.133974	1.690062
44	1	0	-3.853962	-0.234969	1.107760
45	8	0	-6.243603	-3.099151	-1.423338
46	1	0	-9.006509	-2.690932	-0.382465
47	1	0	-9.255867	-1.871085	1.171061
48	1	0	-8.769412	-0.938524	-0.254923
49	1	0	1.158203	0.854012	1.710220
50	6	0	2.846307	-0.380625	1.296253
51	6	0	3.155338	-0.227368	2.799462

52	6	0	3.126004	-1.854021	0.879325
53	1	0	4.220094	-0.343904	3.020530
54	1	0	2.845704	0.746755	3.187125
55	1	0	2.612634	-0.990688	3.369094
56	6	0	4.578069	-2.356723	1.014697
57	6	0	5.358899	-2.437489	-0.282765
58	8	0	4.915507	-2.281348	-1.406439
59	8	0	6.652599	-2.744446	-0.043966
60	6	0	7.502917	-2.908024	-1.199926
61	6	0	4.766216	1.461121	0.962393
62	1	0	4.081331	0.038663	-0.442921
63	6	0	6.044784	1.105537	0.760583
64	6	0	4.444708	2.714201	1.750075
65	1	0	6.866394	1.672809	1.190418
66	1	0	6.314413	0.242832	0.158372
67	1	0	5.330912	3.078154	2.277022
68	1	0	4.103880	3.518327	1.086208
69	1	0	3.651173	2.558343	2.486817
70	1	0	4.587189	-3.377124	1.418764
71	1	0	5.172190	-1.769128	1.720757
72	1	0	2.796518	-2.003460	-0.150158
73	1	0	2.494052	-2.490402	1.508062
74	8	0	2.834205	3.254183	-2.014124
75	1	0	3.739111	1.467868	-2.219506
76	6	0	1.303781	-0.207384	-2.697441
77	1	0	0.463743	0.457755	-2.921629
78	1	0	2.224397	0.227112	-3.106068
79	1	0	1.137663	-1.172448	-3.181889
80	6	0	-4.750840	2.737321	-0.466979
81	1	0	-5.768114	2.471960	-0.771772
82	1	0	-4.831597	3.328232	0.452151
83	1	0	-4.347881	3.385321	-1.249446
84	1	0	8.485512	-3.157939	-0.802123
85	1	0	7.129158	-3.713831	-1.835337
86	1	0	7.546615	-1.980232	-1.774513

54

Center Number	Atomic Number	Atomic Type	Coordinates (Angstroms) X	Y	Z
1	6	0	-0.939266	0.536055	-0.799997
2	6	0	0.397776	1.123826	-0.301541
3	1	0	-0.824202	0.342838	-1.869950
4	1	0	-1.723111	1.295840	-0.703603
5	1	0	0.311737	1.489085	0.726040
6	1	0	0.655830	1.986387	-0.928194
7	6	0	3.624030	-0.981261	0.238097
8	6	0	2.454215	-2.015068	0.249383
9	6	0	1.184005	-1.140249	0.536763
10	6	0	1.482530	0.050828	-0.401621
11	6	0	2.768119	-3.172226	1.167911
12	1	0	2.297764	-2.445609	-0.743869
13	8	0	1.188990	-0.613867	1.881676
14	6	0	-1.965311	-3.145095	-0.339828
15	6	0	-2.375072	-1.735106	-0.880457
16	6	0	-1.374375	-0.765788	-0.062958
17	6	0	-0.169729	-1.711098	0.172662
18	6	0	-0.518180	-2.995030	0.037342
19	1	0	-2.559756	-3.427713	0.542558
20	1	0	-2.126514	-3.935176	-1.082375
21	6	0	-2.054397	-1.720865	-2.392535
22	6	0	-1.965612	-0.369120	1.315485
23	1	0	0.143366	-3.841836	0.189303
24	1	0	-2.311304	-0.774235	-2.874928
25	1	0	-0.990499	-1.907348	-2.568342
26	1	0	-2.611105	-2.511215	-2.904743
27	1	0	-1.221266	0.169721	1.905213
28	1	0	-2.837098	0.282046	1.205422
29	1	0	-2.273456	-1.245842	1.893449
30	6	0	-3.904413	-1.481612	-0.641905
31	6	0	-4.392046	-0.111703	-1.170598
32	6	0	-5.790936	0.308898	-0.715523
33	6	0	-6.201935	1.728079	-1.158756
34	6	0	-5.841232	2.612056	0.045236
35	6	0	-5.892611	1.628349	1.209778
36	8	0	-5.854396	0.355623	0.751143
37	1	0	-6.535351	-0.433341	-1.007967
38	1	0	-7.281792	1.768459	-1.338243
39	1	0	-5.696114	2.029226	-2.079467
40	1	0	-4.790245	2.927433	-0.023532
41	6	0	-6.712368	3.850782	0.259385
42	1	0	-4.059259	-1.489200	0.441884
43	1	0	-4.417551	-0.125251	-2.267889
44	1	0	-3.690996	0.680436	-0.890213
45	8	0	-5.945342	1.876424	2.396872
46	1	0	-6.423050	4.374526	1.174495
47	1	0	-6.603411	4.541639	-0.582180
48	1	0	-7.769383	3.576432	0.341558
49	1	0	1.392665	-0.377599	-1.410324
50	6	0	2.993952	0.410122	-0.243250
51	6	0	3.539831	0.925093	-1.589070

52	6	0	3.195332	1.502935	0.845421
53	1	0	4.629464	1.019702	-1.583117
54	1	0	3.272131	0.264867	-2.418273
55	1	0	3.116685	1.910943	-1.810422
56	6	0	4.640400	1.981097	1.106199
57	6	0	5.232613	2.993557	0.147319
58	8	0	6.377913	2.966615	-0.272045
59	8	0	4.370450	3.986786	-0.152960
60	6	0	4.868183	5.034660	-1.012093
61	6	0	4.865489	-1.512668	-0.473628
62	1	0	3.914278	-0.831568	1.284139
63	6	0	6.071842	-1.425192	0.108017
64	6	0	4.716282	-2.215875	-1.806828
65	1	0	6.966232	-1.802981	-0.380306
66	1	0	6.207827	-0.977482	1.088778
67	1	0	5.693499	-2.380512	-2.268571
68	1	0	4.243002	-3.197656	-1.680948
69	1	0	4.096336	-1.656807	-2.513144
70	1	0	5.346788	1.151309	1.161110
71	1	0	4.658725	2.473384	2.088077
72	1	0	2.795409	1.119600	1.786524
73	1	0	2.590165	2.376505	0.584198
74	8	0	2.487816	-4.336563	0.933866
75	1	0	3.331536	-2.920995	2.089243
76	6	0	0.841514	-1.486145	2.953492
77	1	0	0.627397	-0.845393	3.812266
78	1	0	-0.042824	-2.088875	2.725359
79	1	0	1.668993	-2.155287	3.220496
80	6	0	-4.785126	-2.612295	-1.217751
81	1	0	-5.828175	-2.487735	-0.911025
82	1	0	-4.765736	-2.629549	-2.313334
83	1	0	-4.469582	-3.595821	-0.860764
84	1	0	4.035456	5.725388	-1.137957
85	1	0	5.717240	5.537535	-0.544113
86	1	0	5.174787	4.621938	-1.975641

55

Center Number	Atomic Number	Atomic Type	Coordinates (Angstroms) X	Y	Z
1	6	0	-0.908920	0.609335	-0.809492
2	6	0	0.440705	1.182538	-0.328681
3	1	0	-0.803327	0.393305	-1.876135
4	1	0	-1.677840	1.385207	-0.722706
5	1	0	0.365442	1.566322	0.692879
6	1	0	0.709721	2.030423	-0.970210
7	6	0	3.637853	-0.982136	0.186276
8	6	0	2.443893	-1.982509	0.246972
9	6	0	1.194981	-1.082246	0.540349
10	6	0	1.507415	0.090183	-0.416101
11	6	0	2.753610	-3.135652	1.173849
12	1	0	2.249509	-2.426466	-0.732210
13	8	0	1.221964	-0.543115	1.879962
14	6	0	-1.998383	-3.043028	-0.273481
15	6	0	-2.389126	-1.635576	-0.832866
16	6	0	-1.362101	-0.669559	-0.043499
17	6	0	-0.173082	-1.632737	0.196691
18	6	0	-0.544916	-2.912485	0.085250
19	1	0	-2.587284	-3.300426	0.620342
20	1	0	-2.183143	-3.842184	-1.000867
21	6	0	-2.086861	-1.651104	-2.348746
22	6	0	-1.933450	-0.232766	1.331171
23	1	0	0.104127	-3.767443	0.244649
24	1	0	-2.335007	-0.708157	-2.842873
25	1	0	-1.028111	-1.856758	-2.533841
26	1	0	-2.661553	-2.440905	-2.841659
27	1	0	-1.172224	0.301514	1.903436
28	1	0	-2.793140	0.433004	1.214694
29	1	0	-2.252485	-1.091914	1.929707
30	6	0	-3.910216	-1.350188	-0.582946
31	6	0	-4.381805	0.008568	-1.155572
32	6	0	-5.711764	0.521979	-0.619871
33	6	0	-6.194770	1.843595	-1.241955
34	6	0	-6.933072	2.544808	-0.089434
35	6	0	-6.276813	1.934363	1.145622
36	8	0	-5.580585	0.823044	0.812170
37	1	0	-6.489933	-0.244676	-0.692410
38	1	0	-6.828067	1.668863	-2.115878
39	1	0	-5.332084	2.437986	-1.562704
40	1	0	-6.745108	3.622271	-0.072403
41	6	0	-8.455439	2.307687	-0.064491
42	1	0	-4.051286	-1.317839	0.502492
43	1	0	-4.491370	-0.067657	-2.245513
44	1	0	-3.637759	0.789294	-0.974510
45	8	0	-6.339707	2.327482	2.292869
46	1	0	-8.900451	2.743511	0.834447
47	1	0	-8.916929	2.775706	-0.939237
48	1	0	-8.698614	1.239771	-0.083018
49	1	0	1.408447	-0.351238	-1.418472
50	6	0	3.025418	0.430801	-0.266177
51	6	0	3.560194	0.918479	-1.624751

52	6	0	3.225498	1.522831	0.823198
53	1	0	4.633596	1.128400	-1.603042
54	1	0	3.396795	0.171165	-2.406323
55	1	0	3.049030	1.840749	-1.920645
56	6	0	4.660645	1.993020	1.124072
57	6	0	5.205981	3.036842	0.171745
58	8	0	4.570286	3.970393	-0.285769
59	8	0	6.519372	2.849623	-0.081120
60	6	0	7.163117	3.836215	-0.917773
61	6	0	4.833557	-1.525325	-0.598776
62	1	0	3.984909	-0.847704	1.219537
63	6	0	4.721515	-2.380446	-1.626949
64	6	0	6.204876	-1.075205	-0.145858
65	1	0	5.604614	-2.742298	-2.146630
66	1	0	3.769640	-2.747806	-1.995750
67	1	0	6.993052	-1.612708	-0.680138
68	1	0	6.361034	-0.004052	-0.315796
69	1	0	6.340436	-1.248617	0.929439
70	1	0	5.368996	1.167345	1.206305
71	1	0	4.655670	2.486539	2.105552
72	1	0	2.791285	1.151287	1.752946
73	1	0	2.646279	2.407485	0.538911
74	8	0	2.392994	-4.286993	0.994094
75	1	0	3.389024	-2.893448	2.050925
76	6	0	0.881780	-1.404987	2.961362
77	1	0	0.717105	-0.758599	3.826765
78	1	0	-0.028672	-1.978601	2.761980
79	1	0	1.695175	-2.101635	3.201215
80	6	0	-4.817647	-2.483472	-1.109396
81	1	0	-5.861176	-2.307386	-0.829776
82	1	0	-4.782187	-2.562465	-2.201671
83	1	0	-4.540487	-3.455111	-0.692711
84	1	0	7.094489	4.824843	-0.458859
85	1	0	8.202274	3.518597	-0.991221
86	1	0	6.696951	3.858093	-1.905105

56

Center Number	Atomic Number	Atomic Type	Coordinates (Angstroms) X	Y	Z
1	6	0	-0.888804	0.066209	-1.159500
2	6	0	0.470162	0.799072	-1.099000
3	1	0	-0.901237	-0.499941	-2.094799
4	1	0	-1.692713	0.806978	-1.232889
5	1	0	0.492197	1.525326	-0.281979
6	1	0	0.605837	1.359886	-2.031732
7	6	0	3.832542	-0.881241	-0.178951
8	6	0	2.754263	-1.889639	0.314429
9	6	0	1.465980	-0.989654	0.407636
10	6	0	1.578955	-0.241327	-0.939195
11	6	0	3.213700	-2.621358	1.552427
12	1	0	2.538793	-2.656208	-0.436993
13	8	0	1.591069	0.006187	1.442495
14	6	0	-1.650975	-3.195828	0.743773
15	6	0	-2.189679	-2.095205	-0.229000
16	6	0	-1.159025	-0.882903	0.047341
17	6	0	0.113963	-1.665328	0.458277
18	6	0	-0.179849	-2.908220	0.854333
19	1	0	-2.128185	-3.127015	1.733504
20	1	0	-1.854173	-4.207881	0.375535
21	6	0	-2.030698	-2.647254	-1.664426
22	6	0	-1.611002	-0.014758	1.251275
23	1	0	0.538464	-3.632847	1.224089
24	1	0	-2.382427	-1.953511	-2.432193
25	1	0	-0.983442	-2.879583	-1.880775
26	1	0	-2.599249	-3.575179	-1.776925
27	1	0	-0.840911	0.719484	1.496766
28	1	0	-2.530732	0.533107	1.028788
29	1	0	-1.789083	-0.619778	2.145507
30	6	0	-3.697062	-1.785644	0.074979
31	6	0	-4.308488	-0.722564	-0.868525
32	6	0	-5.694711	-0.208524	-0.475528
33	6	0	-6.228133	0.927108	-1.372538
34	6	0	-5.837330	2.208913	-0.620829
35	6	0	-5.735171	1.732107	0.824334
36	8	0	-5.653462	0.381903	0.868951
37	1	0	-6.408504	-1.030459	-0.403089
38	1	0	-7.318662	0.862075	-1.452976
39	1	0	-5.812977	0.878201	-2.382216
40	1	0	-4.818318	2.511814	-0.901115
41	6	0	-6.770870	3.407558	-0.798083
42	1	0	-3.738823	-1.383769	1.092577
43	1	0	-4.411176	-1.139501	-1.878734
44	1	0	-3.640386	0.139671	-0.959247
45	8	0	-5.710833	2.405798	1.833853
46	1	0	-6.447707	4.245161	-0.174122
47	1	0	-6.772924	3.735835	-1.841920
48	1	0	-7.797787	3.149010	-0.518933
49	1	0	1.350322	-1.020495	-1.679915
50	6	0	3.085839	0.100317	-1.183896
51	6	0	3.403287	-0.191491	-2.668579

52	6	0	3.516806	1.579729	-0.969919
53	1	0	4.464594	-0.045551	-2.891206
54	1	0	3.130692	-1.211484	-2.952776
55	1	0	2.830371	0.487452	-3.310794
56	6	0	3.343959	2.241890	0.414286
57	6	0	3.933936	3.633854	0.394340
58	8	0	5.126636	3.886740	0.343851
59	8	0	2.983829	4.592444	0.420275
60	6	0	3.447153	5.960117	0.378987
61	6	0	5.137552	-1.506494	-0.658697
62	1	0	4.092933	-0.278106	0.695698
63	6	0	6.306291	-0.910525	-0.372836
64	6	0	5.106754	-2.811508	-1.424189
65	1	0	7.252095	-1.322214	-0.715513
66	1	0	6.355087	0.006764	0.208284
67	1	0	6.108210	-3.079735	-1.771513
68	1	0	4.743793	-3.632853	-0.794208
69	1	0	4.445546	-2.769525	-2.295205
70	1	0	3.884423	1.682675	1.182873
71	1	0	2.297886	2.285563	0.710559
72	1	0	2.979520	2.189080	-1.707753
73	1	0	4.578266	1.645883	-1.238888
74	8	0	2.971722	-3.793311	1.792453
75	1	0	3.849813	-2.042034	2.251323
76	6	0	1.424288	-0.394047	2.799740
77	1	0	0.577634	-1.075284	2.928492
78	1	0	2.328793	-0.869526	3.199111
79	1	0	1.238026	0.520076	3.368779
80	6	0	-4.571307	-3.059189	0.073434
81	1	0	-5.574625	-2.842234	0.452229
82	1	0	-4.684239	-3.478722	-0.932594
83	1	0	-4.157017	-3.838788	0.717334
84	1	0	4.085614	6.169710	1.239807
85	1	0	4.004298	6.143778	-0.542251
86	1	0	2.546481	6.571557	0.411571

57

Center Number	Atomic Number	Atomic Type	Coordinates (Angstroms) X	Y	Z
1	6	0	-0.891719	0.616218	-0.786071
2	6	0	0.461246	1.177546	-0.300004
3	1	0	-0.788556	0.415710	-1.855964
4	1	0	-1.657699	1.393481	-0.686754
5	1	0	0.390835	1.549032	0.726466
6	1	0	0.733375	2.031649	-0.931910
7	6	0	3.644620	-0.991680	0.233919
8	6	0	2.454141	-2.001563	0.252899
9	6	0	1.205146	-1.097540	0.545630
10	6	0	1.521974	0.081184	-0.402174
11	6	0	2.749131	-3.161812	1.173002
12	1	0	2.283254	-2.430624	-0.738654
13	8	0	1.234847	-0.564094	1.887235
14	6	0	-1.989382	-3.042229	-0.296991
15	6	0	-2.374635	-1.627766	-0.843036
16	6	0	-1.349684	-0.672350	-0.038246
17	6	0	-0.163339	-1.640787	0.195800
18	6	0	-0.537217	-2.918484	0.069445
19	1	0	-2.582872	-3.307778	0.591352
20	1	0	-2.172403	-3.833139	-1.033727
21	6	0	-2.065197	-1.627093	-2.357441
22	6	0	-1.926815	-0.250744	1.338816
23	1	0	0.108810	-3.777196	0.221386
24	1	0	-2.310603	-0.678603	-2.842181
25	1	0	-1.005734	-1.831475	-2.539883
26	1	0	-2.638095	-2.411008	-2.861695
27	1	0	-1.168776	0.279204	1.919317
28	1	0	-2.786660	0.415248	1.225607
29	1	0	-2.247628	-1.116040	1.927423
30	6	0	-3.896310	-1.342609	-0.596878
31	6	0	-4.363081	0.023809	-1.155030
32	6	0	-5.693926	0.532939	-0.617389
33	6	0	-6.173310	1.862557	-1.225114
34	6	0	-6.914408	2.550698	-0.066504
35	6	0	-6.262354	1.924990	1.163095
36	8	0	-5.566539	0.816795	0.818461
37	1	0	-6.472934	-0.231710	-0.701369
38	1	0	-6.804055	1.699161	-2.103064
39	1	0	-5.308854	2.459620	-1.535991
40	1	0	-6.725649	3.627718	-0.036143
41	6	0	-8.437043	2.314501	-0.048914
42	1	0	-4.042247	-1.323209	0.488208
43	1	0	-4.469188	-0.039402	-2.246131
44	1	0	-3.618159	0.800877	-0.962162
45	8	0	-6.327950	2.304501	2.314745
46	1	0	-8.884333	2.739676	0.853987
47	1	0	-8.895611	2.793558	-0.919208
48	1	0	-8.681004	1.247086	-0.081186
49	1	0	1.415159	-0.351297	-1.407635
50	6	0	3.041294	0.408998	-0.256223
51	6	0	3.587977	0.901870	-1.609937

52	6	0	3.280953	1.505037	0.821093
53	1	0	4.678949	0.990427	-1.606488
54	1	0	3.315516	0.231601	-2.429459
55	1	0	3.173194	1.888049	-1.842105
56	6	0	4.739143	1.960439	1.021525
57	6	0	5.181241	3.106935	0.134708
58	8	0	4.463021	3.995486	-0.289433
59	8	0	6.511090	3.068471	-0.094841
60	6	0	7.065991	4.160073	-0.860646
61	6	0	4.876762	-1.554545	-0.469486
62	1	0	3.936386	-0.839378	1.279002
63	6	0	6.081377	-1.493558	0.120125
64	6	0	4.719528	-2.261623	-1.799559
65	1	0	6.969325	-1.897147	-0.359486
66	1	0	6.220146	-1.046488	1.100990
67	1	0	5.695734	-2.459654	-2.250354
68	1	0	4.215137	-3.227414	-1.671524
69	1	0	4.123795	-1.688887	-2.515531
70	1	0	5.455247	1.142525	0.920351
71	1	0	4.855326	2.339667	2.046210
72	1	0	2.895925	1.136139	1.773592
73	1	0	2.688526	2.389449	0.565285
74	8	0	2.454850	-4.322803	0.938611
75	1	0	3.312552	-2.917614	2.096203
76	6	0	0.867535	-1.418598	2.966253
77	1	0	-0.045485	-1.982999	2.753961
78	1	0	1.669066	-2.122796	3.222243
79	1	0	0.695171	-0.767162	3.826378
80	6	0	-4.803088	-2.467798	-1.141443
81	1	0	-5.847427	-2.293908	-0.863568
82	1	0	-4.763481	-2.532493	-2.234529
83	1	0	-4.528876	-3.445163	-0.736356
84	1	0	6.883195	5.110695	-0.354878
85	1	0	8.134283	3.956746	-0.920771
86	1	0	6.623572	4.186312	-1.858822

58

Center Number	Atomic Number	Atomic Type	Coordinates (Angstroms) X	Y	Z
1	6	0	-0.856796	0.081493	-1.083501
2	6	0	0.508287	0.770536	-0.875917
3	1	0	-0.827663	-0.390046	-2.069513
4	1	0	-1.640582	0.846061	-1.126925
5	1	0	0.501924	1.400653	0.018577
6	1	0	0.691380	1.425548	-1.735185
7	6	0	3.801633	-1.156642	-0.075221
8	6	0	2.658690	-2.112316	0.340545
9	6	0	1.394135	-1.194344	0.462698
10	6	0	1.603900	-0.292560	-0.773868
11	6	0	3.010580	-2.992484	1.518566
12	1	0	2.413592	-2.819253	-0.464614
13	8	0	1.483735	-0.317629	1.608591
14	6	0	-1.797659	-3.336122	0.400370
15	6	0	-2.269764	-2.108582	-0.445358
16	6	0	-1.211402	-0.973647	0.003504
17	6	0	0.022204	-1.834361	0.371362
18	6	0	-0.326156	-3.105619	0.598318
19	1	0	-2.309905	-3.376405	1.373658
20	1	0	-2.010533	-4.288658	-0.098323
21	6	0	-2.084739	-2.489517	-1.932605
22	6	0	-1.685245	-0.225206	1.276933
23	1	0	0.343866	-3.902176	0.905814
24	1	0	-2.388272	-1.694654	-2.618592
25	1	0	-1.039838	-2.733455	-2.148465
26	1	0	-2.680237	-3.375296	-2.172969
27	1	0	-0.898547	0.439896	1.637766
28	1	0	-2.570927	0.384310	1.077570
29	1	0	-1.930339	-0.917895	2.087993
30	6	0	-3.775371	-1.786594	-0.146479
31	6	0	-4.325579	-0.596734	-0.968686
32	6	0	-5.699705	-0.078758	-0.539474
33	6	0	-6.165877	1.183659	-1.292993
34	6	0	-5.738788	2.340796	-0.376778
35	6	0	-5.696144	1.680588	0.997409
36	8	0	-5.667621	0.333339	0.870232
37	1	0	-6.447535	-0.871961	-0.586109
38	1	0	-7.256184	1.177259	-1.399015
39	1	0	-5.730903	1.244800	-2.293619
40	1	0	-4.699779	2.626498	-0.594959
41	6	0	-6.612347	3.595516	-0.419347
42	1	0	-3.835371	-1.507907	0.910635
43	1	0	-4.422115	-0.887557	-2.022817
44	1	0	-3.623804	0.242820	-0.944547
45	8	0	-5.673560	2.218306	2.085372
46	1	0	-6.272143	4.328732	0.316893
47	1	0	-6.567312	4.055784	-1.411003
48	1	0	-7.657894	3.352485	-0.202358
49	1	0	1.445191	-0.970033	-1.626639
50	6	0	3.121518	0.077738	-0.841338
51	6	0	3.529579	0.178337	-2.323467

52	6	0	3.545169	1.369225	-0.081135
53	1	0	4.571012	0.495684	-2.444185
54	1	0	3.416707	-0.782314	-2.832473
55	1	0	2.898687	0.904973	-2.846865
56	6	0	3.193830	2.724547	-0.714606
57	6	0	3.845953	3.883431	0.011308
58	8	0	4.898568	3.839498	0.623723
59	8	0	3.126261	5.018103	-0.126156
60	6	0	3.681410	6.209603	0.472918
61	6	0	4.977065	-1.821176	-0.781277
62	1	0	4.203975	-0.752689	0.859496
63	6	0	4.869850	-2.899599	-1.570910
64	6	0	6.323725	-1.183061	-0.528856
65	1	0	5.744846	-3.328582	-2.052019
66	1	0	3.924795	-3.396059	-1.770969
67	1	0	6.565755	-1.214455	0.540948
68	1	0	7.120766	-1.691081	-1.078953
69	1	0	6.330155	-0.125562	-0.819815
70	1	0	2.119789	2.910704	-0.754222
71	1	0	3.557093	2.782212	-1.749111
72	1	0	4.634499	1.340130	0.028022
73	1	0	3.131958	1.328080	0.930336
74	8	0	4.083986	-2.985823	2.096187
75	1	0	2.228630	-3.708807	1.836829
76	6	0	1.285213	-0.874235	2.906112
77	1	0	2.174306	-1.407029	3.261294
78	1	0	1.100668	-0.030301	3.575289
79	1	0	0.421774	-1.547410	2.936904
80	6	0	-4.687354	-3.020062	-0.327040
81	1	0	-5.694823	-2.816596	0.047921
82	1	0	-4.781831	-3.310607	-1.379389
83	1	0	-4.316529	-3.885271	0.227956
84	1	0	4.653778	6.438775	0.031357
85	1	0	2.965966	7.001802	0.256691
86	1	0	3.792123	6.075415	1.550981
